# NIBV Induces Incomplete Autophagy via AMPK‐TFEB, Causing Kidney Injury in Chicks

**DOI:** 10.1002/advs.202514993

**Published:** 2026-04-09

**Authors:** Cheng Huang, Yizhou Zeng, Yunfeng Chen, Zhengqing Li, Salma Mbarouk Omar, Shengwei Zhong, Ping Liu, Zhanhong Zheng, Gaofeng Cai, Xiaona Gao, Xiaoquan Guo

**Affiliations:** ^1^ Jiangxi Provincial Key Laboratory for Animal Health College of Animal Science and Technology Jiangxi Agricultural University Nanchang Jiangxi China

**Keywords:** AMPK, autophagolysosome, chicken, kidney, NIBV

## Abstract

Nephropathogenic infectious bronchitis virus (NIBV) infection leads to urate deposition in chick kidneys, resulting in severe kidney injury and affecting chicken performance, but its pathogenesis has not been fully elucidated. In this study, we observed a large number of autophagosomes in the kidney tissues and cells of NIBV‐infected chicks under electron microscopy, and the proteomics results revealed that AMPK signaling was altered. In the early and late stages of viral infection, the expression of autophagolysosome‐related proteins was significantly upregulated, and autophagic flux was unimpeded. However, at the peak of viral infection, the expression of lysosome‐related proteins was significantly downregulated in chick kidneys and tubular epithelial cells. Blocked autophagic flux was accompanied by the downregulation of AMPK expression. Therefore, we overexpressed AMPK and found that increased AMPK phosphorylation activated TFEB and promoted its nuclear translocation. Autophagic flux was restored, and NIBV replication was significantly reduced. Overall, this study reveals that NIBV can inhibit the nuclear translocation of TFEB by suppressing the expression of AMPK, leading to the blockade of autophagolysosomal functions, in turn increasing NIBV replication and triggering severe kidney injury in chicks.

## Graphical abstract

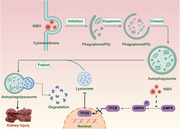

## 1 Introduction

Nephropathogenic infectious bronchitis virus (NIBV) is the most common and clinically significant type of virus that affects poultry productivity. Comprehensive studies have shown that chicks are highly susceptible to NIBV, and there is a wide range of genotypes and serotypes of NIBV, which poses a great challenge for the prevention and control of the virus [1, 2]. NIBV can colonize several organs in the chick, including the kidneys, lungs, and liver [3]. Chicks infected with NIBV exhibit enlarged, pale‐colored kidneys, thickened ureters, white urine deposits on the surface of the kidneys, and death [4, 5]. However, knowledge of how NIBV infection affects the normal function of renal cells remains limited.

As a fundamental cellular process, autophagy is important for both hosts and viruses and can either eliminate viruses to inhibit viral replication or promote viral replication by either evading or disrupting degradation by the autophagy–lysosome pathway or hijacking autophagy [6]. The autophagy–lysosomal pathway is involved in autophagy genesis and is the first line of defense in innate immunity to clear pathogens. Lysosomes are considered the degradation centers of most eukaryotic cells. The acidification of lysosomes, lysosomal transport, the fusion of lysosomes with late endosomes or autophagosomes, and the maturation of lysosomal proteases are essential for the maintenance of normal lysosomal physiology [7]. Mechanistically, autophagy‐lysosome biogenesis is regulated by the transcription factor EB (TFEB), which affects the production of lysosome‐related proteins [8, 9]. When the translocation of TFEB to the nucleus is blocked, it disrupts the process of lysosomal synthesis and hinders autophagy. Recent findings indicate that CoV hinders the process of autophagic flux by controlling lysosomal degradation through the regulation of lysosomal acidity or by impeding the fusion of autophagosomes and lysosomes [10, 11, 12]. The formation of double‐membrane vesicles (DMVs), a protective structure during the infection cycle of coronaviruses, can provide sites for viral replication and protect their genetic material from lysosomal clearance. It has been reported that the production of coronavirus particles is positively correlated with the number of DMVs [13]. Because autophagosomes share some similarities with DMVs, some viruses (e.g., coronaviruses) can hijack autophagy initiation to form unique DMVs, and autophagosomes that do not fuse with lysosomes can also rupture and release viral particles [14]. However, the relationship between NIBV and the occurrence of cellular autophagy in chick kidneys remains unclear.

AMP‐activated protein kinase (AMPK) is a highly conserved serine/threonine kinase and is among the core regulators of eukaryotic cellular and organismal metabolism [15]. Autophagy, as a cellular self‐protection mechanism under stress, is also regulated by AMPK, which promotes autophagy by regulating autophagy‐related factors that play different roles in the regulation of autophagy at different levels [16]. For example, AMPK regulates the activity of autophagy‐related proteins by mediating the phosphorylation of ULK1 and TFEB [17, 18]. Moreover, AMPK plays a role in viral infection, and different viruses may activate or inhibit AMPK to optimize their replication environment [19]. AMPK activation inhibits HHV‐6A replication by downregulating glycolysis and blocking viral‐induced metabolic reprogramming [20]. The ARV p17 protein hijacks AMPK–ULK1 signaling and triggers autophagy to increase self‐replication [21]. Nevertheless, the role of AMPK in kidney injury in chicks due to NIBV infection remains unknown.

In this study, we established a chicken model of kidney injury due to NIBV infection and performed proteomic screening of cellular autophagy, lysosomal, oxidative stress, and AMPK signaling pathways. Dynamic changes in viral load, cellular autophagy, and lysosomal function in chicken kidney and primary renal tubular epithelial cells during NIBV infection were continuously monitored. We also utilized the overexpression technique and the AMPK inhibitor Compound C in chicken primary renal tubular epithelial cells and reported that viral replication, cellular autophagy, and lysosomal function were closely related to changes in AMPK expression. Ultimately, our study demonstrated that the activation of AMPK could regulate lysosomal function through TFEB and alleviate autophagy flux blockade, thus reducing NIBV replication and ultimately ameliorating renal injury caused by NIBV in chickens with gout.

## 2 Results

### 2.1 NIBV Infection Causes Severe Kidney Damage in Chicks

**FIGURE 1:**
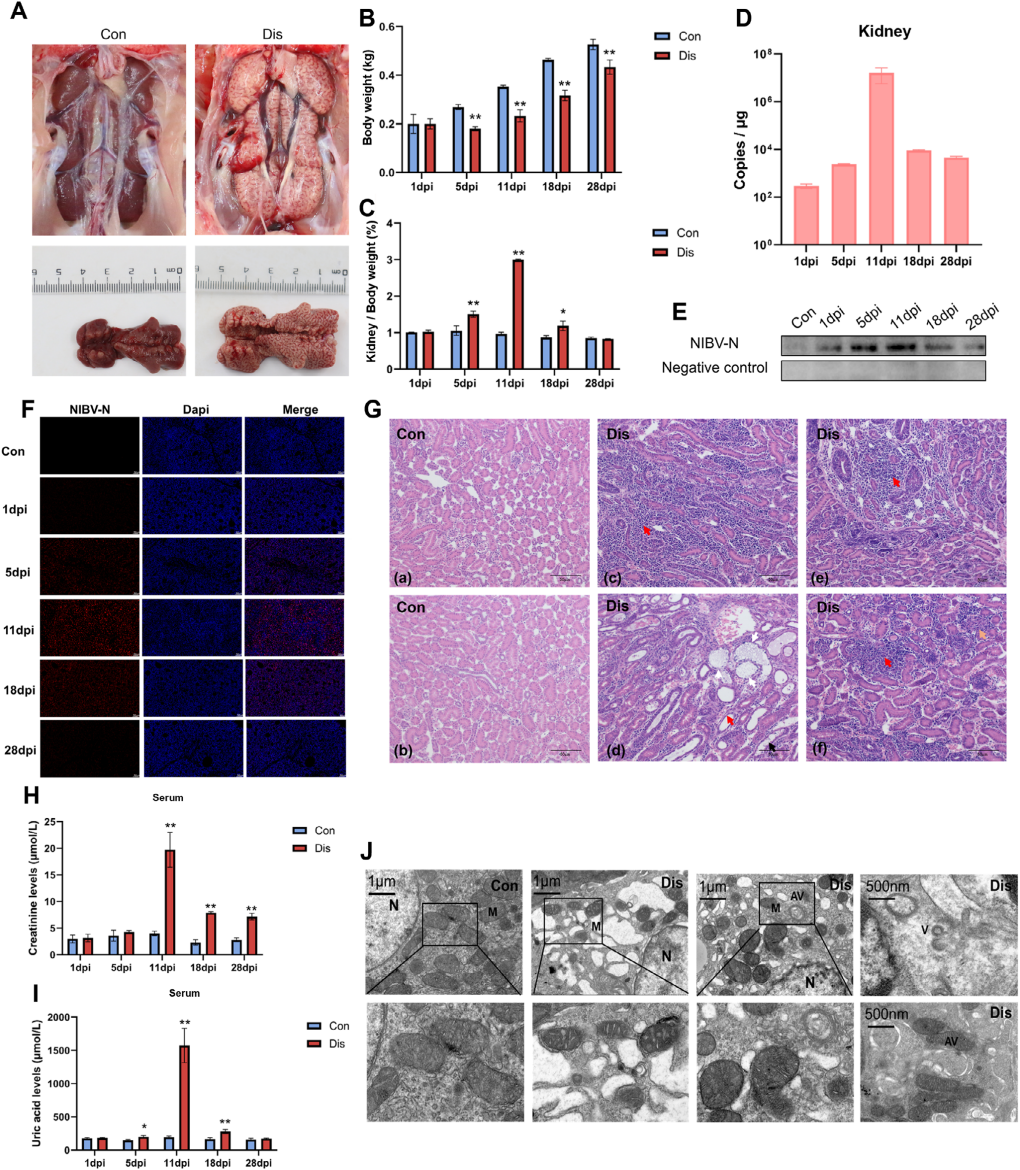
NIBV infection leads to severe kidney damage in chicks. (A) Anatomical drawings of NIBV‐infected chicks with normal (Con) and NIBV‐infected (Dis) kidneys. (B) Chick weight changes. (C) Chick kidney index results. (D) Changes of NIBV viral load in kidney tissues. (E) Western blot explores the expression levels of NIBV‐N protein. (F) Immunofluorescence of NIBV‐N protein. (G) Renal histology of chickens by H&E staining. Massive inflammatory cell infiltration of the renal interstitium (red arrows); detachment of renal epithelial cells with edema (white arrows); flattening and detachment of epithelial cells, with the appearance of exposed tubular basement membranes (black arrows); and glomerular cleavage (orange arrows). (H) Changes in serum creatinine levels. (I) Changes in serum uric acid content. (J) Ultrastructural observation of kidney tissue. N: nucleus, M: mitochondria, E: endoplasmic reticulum, V: virus particles, AV: autophagic vacuoles. data are shown as the mean ± SD (n = 6).

In this study, a model of NIBV‐infected chicks was established. Dissections were performed, and it was found that compared with chicks in the Con group (normal group), chicks in the Dis group (pathology group) had enlarged kidneys with white urate deposits, significant weight loss, and increased renal indices (Figure 1A–C). Absolute quantitative PCR, WB for the NIBV‐N protein, and immunofluorescence staining were used to assess the presence of NIBV in kidney tissues at different times of infection, which revealed that the titer of NIBV gradually increased in the early stage of infection, reached the highest level at 11 dpi, and then decreased (Figure 1D–F). HE staining of renal pathology sections and serum creatinine and uric acid levels revealed that the chicks in the Dis group exhibited severe kidney damage (Figure 1G–I). In comparison with those in the Con group, the kidney cells in the Dis group were found to have abnormal structures, with crumpled nuclei, broken mitochondria with missing cristae junctions, and viral particles distributed in the cytoplasm, in addition to the presence of a large number of autophagic vesicles (Figure 1J). These results reveal that NIBV infection leads to autophagy in chick kidney tissues.

### 2.2 Renal Proteomic Analysis After NIBV Infection

**FIGURE 2:**
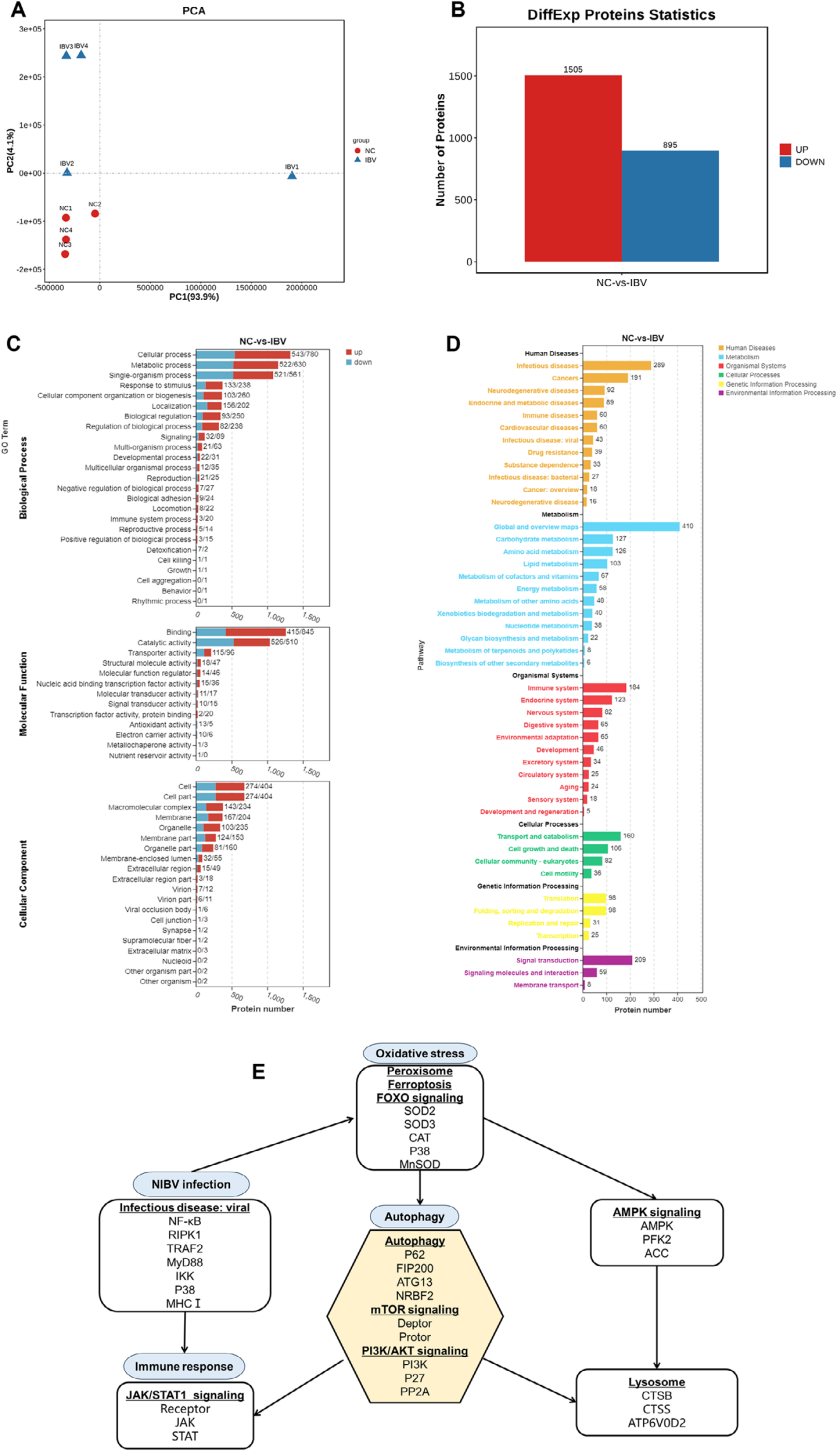
11dpi proteomic analysis of normal (NC) and NIBV‐infection (IBV) groups. (A) Principal Component Analysis of Samples (PCA). The horizontal coordinate is the first principal component, and the vertical coordinate is the second principal component. (B) Bar chart of differential protein numbers. (C) Enrichment analysis of differential protein GO: The horizontal coordinate is the number of differential proteins in GO term (the basic unit of GO), the vertical coordinate represents GO term. (D) Enrichment analysis of differential protein KEGG: The horizontal coordinate represents the number of differential proteins in the KEGG pathway, and the vertical coordinate represents the KEGG pathway. (E) Enrichment pattern of autophagy related items after NIBV infection. Data are shown as the mean ± SD (n = 4).

Because a large number of autophagic vesicles were detected in renal cells by electron microscopy, we collected renal tissues from the Con and Dis groups at 11 dpi. Proteomics analysis was subsequently used to screen for key differentially expressed proteins (Figure 2A). By differentially expressed proteins (DEPs) analysis, the expression of 1505 proteins was upregulated, and the expression of 895 proteins was downregulated (Figure 2B). GO annotation, and Kyoto Encyclopedia of Genes and Genomes (KEGG) enrichment tests revealed that the DEPs in the NIBV infection group were enriched in biological processes related to cellular metabolism, cellular activity, and DNA replication. KEGG analysis revealed enrichment of the DEPs in the autophagy pathway, AMPK signaling pathway, and lysosomal pathways, suggesting that autophagy was activated after virus infection (Figure 2C–E). Therefore, we hypothesized that after NIBV infection, the large number of autophagic vesicles in renal tissues was caused by abnormal lysosomal function. This may be related to alterations in the AMPK signaling pathway.

### 2.3 NIBV Infection Causes Cellular Autophagy in the Chick Kidney

**FIGURE 3:**
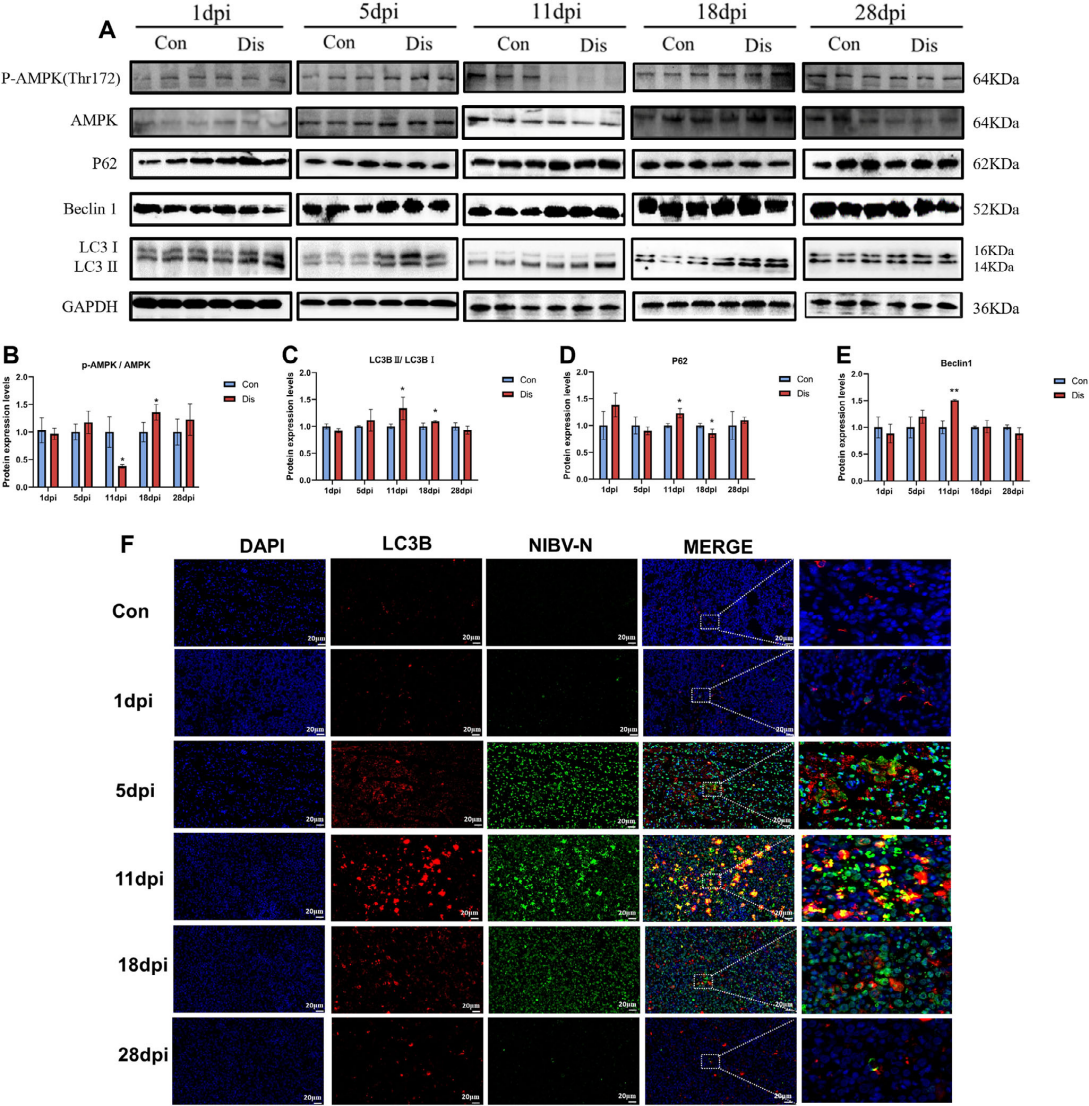
NIBV Infection Causes Cellular Autophagy in the Chick Kidney. (A‐E) WB and quantitative analysis of AMPKα2, mTOM and autophagy‐related proteins. Data are shown as the mean ± SD (n = 3). (F) Effect of NIBV infection on the co‐localization of LC3B and NIBV‐N fluorescence in chick kidney. Blue fluorescence: Nuclear staining (DAPI), red fluorescence: LC3B staining, green fluorescence NIBV‐N staining, scale: 20 µm.

In the kidneys of chicks infected with the NIBV virus, the levels of LC3B‐II and Beclin1 significantly increased, whereas the level of p62 significantly decreased at 5 dpi but sharply increased at 11 dpi. In addition, our study revealed that the expression of AMPK increased significantly to 5 dpi and 18 dpi, with a dramatic decrease at 11 dpi (Figure 3A–E). Furthermore, fluorescence colocalization analysis of LC3B and NIBV‐N showed that the colocalization (yellow) of LC3B (red) and NIBV‐N (green) in NIBV‐infected kidneys increased but then decreased, with the most pronounced colocalization occurring at 11 dpi, compared to that in the Con group (Figure 3F). In summary, at 11 dpi of NIBV infection, renal autophagic vesicles accumulated; during the remaining period, cellular autophagy occurred in the kidney, with an unimpeded autophagic flux, and may have been associated with alterations in AMPK activity.

### 2.4 NIBV Infection Leads to Impaired Autophagic Lysosomal Function

**FIGURE 4:**
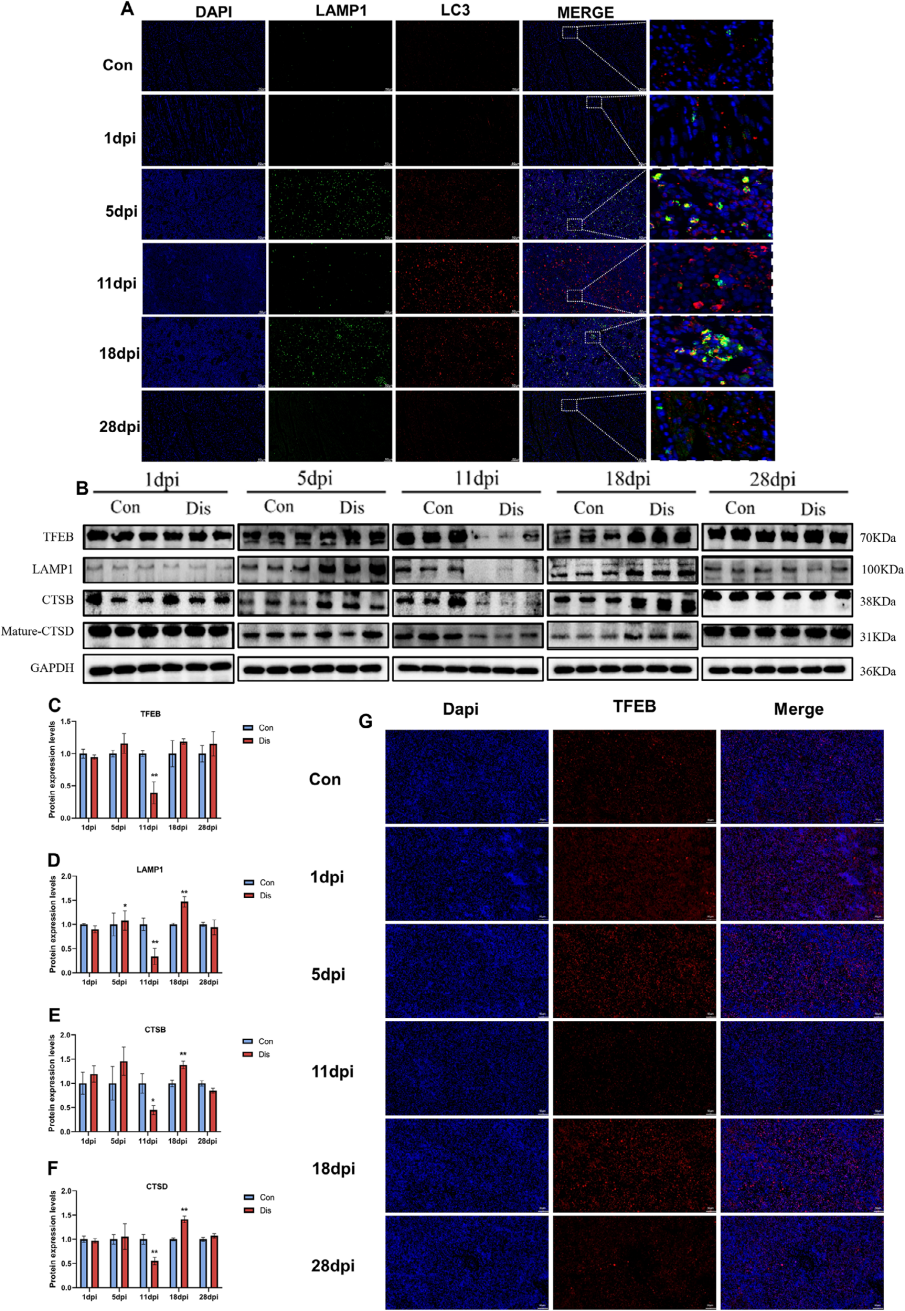
NIBV infection leads to impaired autophagic lysosomal function. (A) Effects of NIBV infection on renal co‐localization of LC3B and LAMP1 in chicks. Blue fluorescence: Nuclear staining (DAPI), red fluorescence: LC3B staining, green fluorescence: LAMP1 staining, scale: 50 µm. (B–F) WB of lysosome‐associated protein in chick kidney and its quantitative analysis. Data are shown as the mean ± SD (n = 3). (G) Immunofluorescence localization and expression results of TFEB in chick kidney. Blue fluorescence: Nuclear staining (DAPI), red fluorescence: TFEB staining, scale: 50 µm.

To understand the cause of autophagy blockade in NIBV infection, we first investigated the fusion mechanism between autophagosomes and lysosomes. The colocalization of LC3B and LAMP1 increased significantly at 5 dpi and 18 dpi but decreased dramatically at 11 dpi, suggesting that the number of autophagic lysosomes decreased during peak viral replication (Figure 4A). The expression of LAMP1 in the kidney significantly decreased at the peak of viral infection (Figure 4B,D). Thus, NIBV infection at 11 dpi results in reduced autophagosome and lysosome binding and autophagolysosome production. Moreover, the activities of CTSB and CTSD decreased during the peak of NIBV infection. However, the activities of CTSB and CTSD significantly increased during the early and late stages of viral infection (Figure 4B,E,F). These findings suggest that NIBV inhibits lysosomal function by affecting the maturation of protein hydrolases in lysosomes at the peak of infection.

TFEB, a core regulator of autophagolysosomes, enters the nucleus of renal cells in the early stages of NIBV infection in significantly increased amounts. However, during the peak of viral infection, TFEB expression in chick kidneys sharply declined, followed by a gradual recovery of TFEB function. (Figure 4B,C,G). These data suggest that NIBV infection enhances TFEB expression during both the early and late phases, but TFEB expression is suppressed during the peak of viral infection. These findings indicate that NIBV infection leads to a reduction in autophagic lysosomal function. The blockade of autophagic flux in chick kidneys at the peak of NIBV infection is correlated with altered lysosomal function.

### 2.5 NIBV Infection Leads to Autophagy in Renal Tubular Epithelial Cells

**FIGURE 5:**
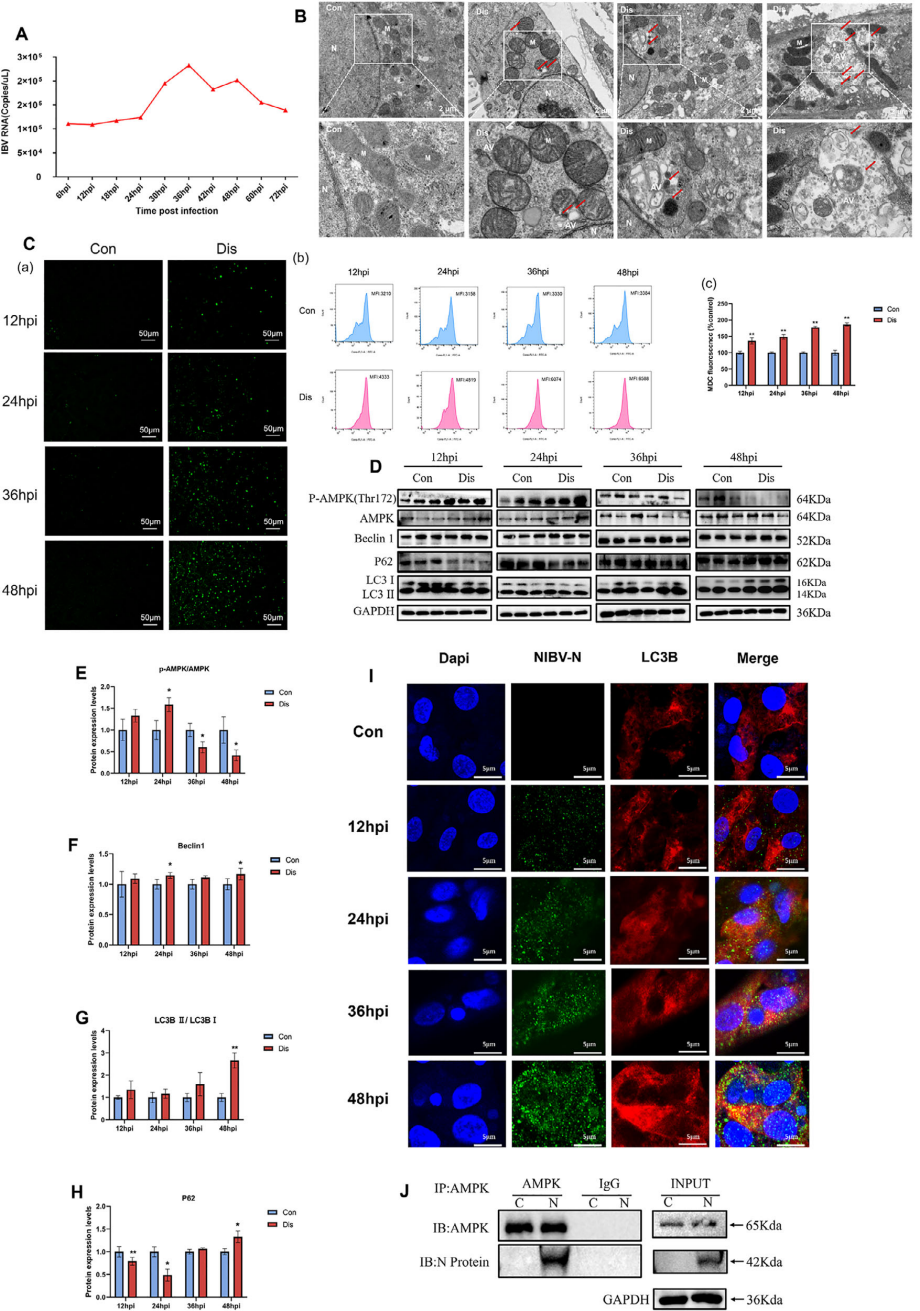
NIBV Infection Leads to Autophagy in Renal Tubular Epithelial Cells. (A) Viral load changes. (B) ultrastructural observation. N: nucleus, M: mitochondria, AV: autophagic vacuoles, red arrows: autophagic vesicles and autophagic lysosomes. (C) Cellular MDC levels, (a) MDC fluorogram, (b) MDC flow graph, (c) MDC flow analysis results scale: 50 µm. (D–H) WB and quantitative analysis of AMPKα2, mTOM and autophagy‐related proteins. Data are shown as the mean ± SD (n = 3). (I) Effect of NIBV infection on the co‐localization of LC3B and NIBV‐N fluorescence in cells. Blue fluorescence: Nuclear staining (DAPI), red fluorescence: LC3B staining, green fluorescence NIBV‐N staining, scale: 5 µm. (J) Immunoprecipitation to detect AMPK‐NIBV N Protein interactions.

NIBV infection of chicken primary renal tubular epithelial cells leads to cell death and the formation of typical cytopathic effects (CPEs) (Figure S1A,B). Absolute fluorescence quantification was performed to determine the change in viral load within 72 h in NIBV‐infected cells, which revealed that viral replication peaked at 36 hpi (Figure 5A). Moreover, electron microscopy revealed a large number of autophagosomes in the NIBV‐infected group of cells (Figure 5B).

The effect of NIBV infection on the number of autophagic vesicles in cells was subsequently examined using an eosinophilic MDC fluorescent probe. Compared with that in the normal group, the level of MDC in the cells at 12, 24, 36, and 48 hpi after NIBV infection significantly increased, and the level of MDC gradually increased with increasing infection time (Figure 5C). Moreover, NIBV regulated the expression of LC3A, LC3B, and Beclin1. p62 was downregulated at 12 hpi and 24 hpi and upregulated at 36 hpi and 48 hpi. However, AMPK expression was upregulated at 12 hpi and 24 hpi but downregulated at 36 hpi and 48 hpi (Figure 5D–H). The colocalization of LC3B and NIBV‐N in renal tubular epithelial cells also increased progressively over time during NIBV infection (Figure 5I). Furthermore, immunoprecipitation analysis revealed an interaction between AMPK and NIBV‐N (Figure 5J). These results indicate that at 12 hpi and 24 hpi, autophagy occurs with an unimpeded autophagic flux. However, at 36 hpi and 48 hpi, autophagic flux is disrupted, and viral replication increases, which is potentially linked to changes in AMPK activity.

### 2.6 NIBV Infection Causes Lysosomal Dysfunction in Renal Tubular Epithelial Cells

**FIGURE 6:**
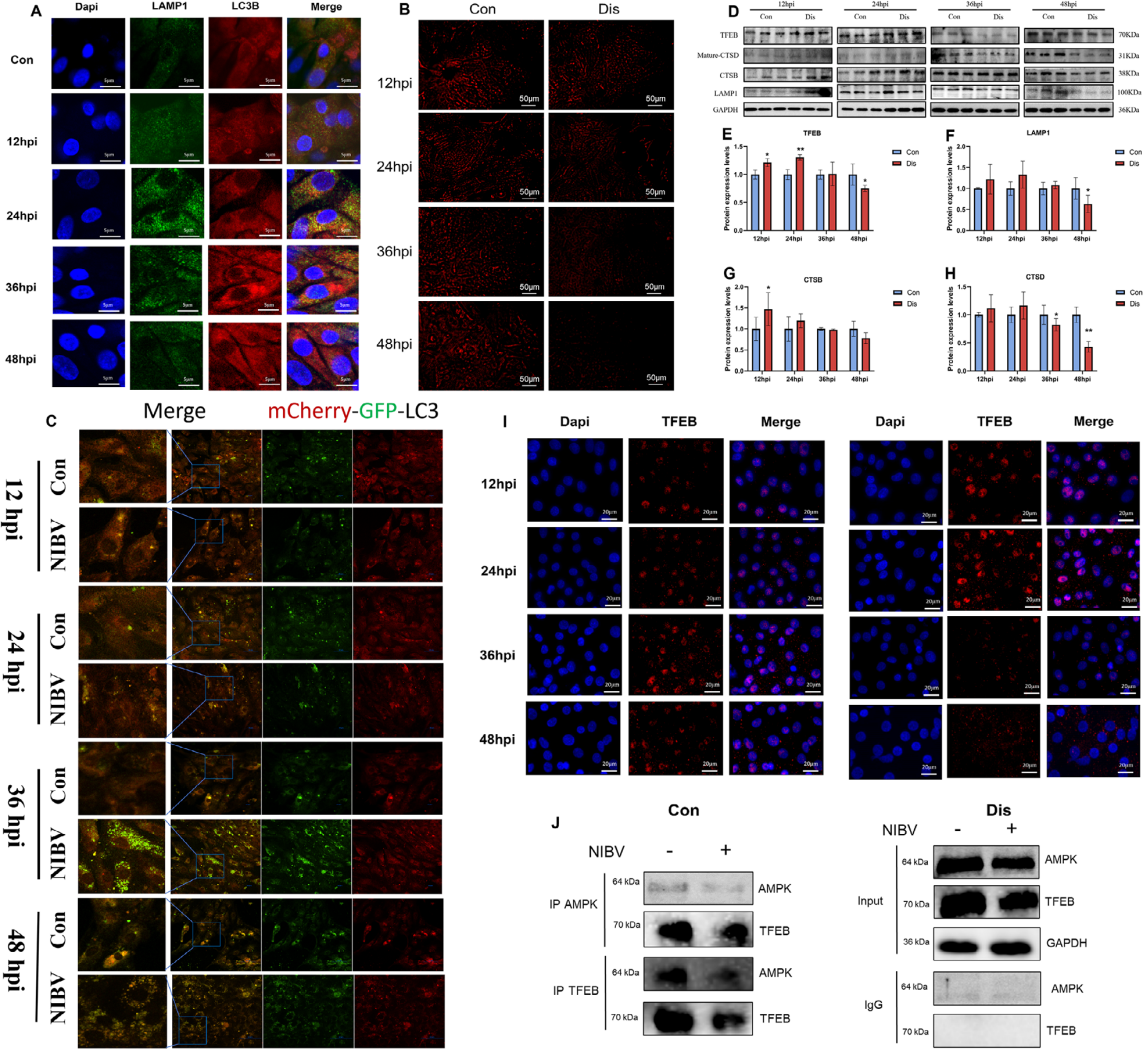
NIBV infection causes lysosomal dysfunction in renal tubular epithelial cells. (A) Effect of NIBV infection on the co‐localization of LC3B and LAMP1 in cells. Blue fluorescence: Nuclear staining (DAPI), red fluorescence: LC3B staining, green fluorescence: LAMP1 staining, scale: 5 µm. (B) Results of changes in the pH value of cytosolic lysosomes, scale: 50 µm. (C) Effects of NIBV infection on the fusion of autophagosomes and lysosomes in cells transfected with the mCherry‐GFP‐LC3 dual‐fluorescent plasmid. GFP: green fluorescence; mCherry: red fluorescence. (D‐H) WB and quantitative analysis of lysosome‐associated proteins. Data are shown as the mean ± SD (n = 3). (I) TFEB immunofluorescence localization and expression results. Blue fluorescence: Nuclear staining (DAPI), red fluorescence: TFEB staining, scale: 20 µm. (J) Immunoprecipitation to detect AMPK‐TFEB interactions.

Unimpeded autophagic flux is closely related to the fusion of autophagosomes and lysosomes. Compared with that in the normal group, the fluorescence colocalization of intracellular LC3B and LAMP1 was obvious at 12 hpi and 24 hpi. At 36 hpi and 48 hpi, the colocalization was attenuated (Figure 6A). Furthermore, the cellular lysosomal pH was assayed after NIBV infection using the Lysosomal probe LysoTracker Red. The intensity of red fluorescence in NIBV‐infected cells gradually decreased as time progressed, resulting in increased lysosomal pH and lysosomal alkalinization in renal tubular epithelial cells (Figure 6B). Therefore, by transfecting mCherry‐GFP‐LC3 dual‐fluorescent plasmids and using laser confocal microscopy to detect the fusion of autophagosomes with lysosomes, we observed that autophagy flowed smoothly at 12hpi and 24hpi, with autophagosomes being degraded by lysosomes. However, at 36hpi and 48hpi, impaired lysosomes failed to degrade autophagosomes, resulting in blocked autophagy (Figure 6C).

In terms of the effect of NIBV infection on cellular lysosomal function, NIBV upregulated the expression of LAMP1, TFEB, CTSB, and CTSD at 12 and 24 hpi but downregulated their expression at 36 hpi and 48 hpi (Figure 6D–H). These findings indicated that at 12 hpi and 24 hpi, cellular lysosomal function was increased; at 36 hpi and 48 hpi, cellular lysosomal function was impaired, and the fusion of autophagic vesicles with lysosomes was blocked. Furthermore, compared with those in the normal group, cellular TFEB immunofluorescence clustering and nucleation significantly increased at 12 hpi and 24 hpi, whereas they significantly decreased at 36 hpi and 48 hpi (Figure 6I). Given the important role of TFEB in regulating lysosomal function and the fact that changes in AMPK expression are consistent with the role of TFEB, immunoprecipitation followed by WB was conducted, which revealed an interaction between AMPK and TFEB (Figure 6J). These results suggest that NIBV infection causes lysosomal dysfunction in renal tubular epithelial cells, which may be related to the regulation of TFEB by AMPK.

### 2.7 AMPK Affects NIBV Replication Through the Regulation of Autophagic Flux

To investigate the relationship between AMPK in autophagic flux and viral replication, an AMPKα2 overexpression plasmid was constructed (Figure S2A,B). At 48 h after plasmid transfection, green fluorescence was observed, and the expression of AMPKα2 was extremely significantly increased at both the transcriptional and translational levels (Figure S2C–E).

**FIGURE 7:**
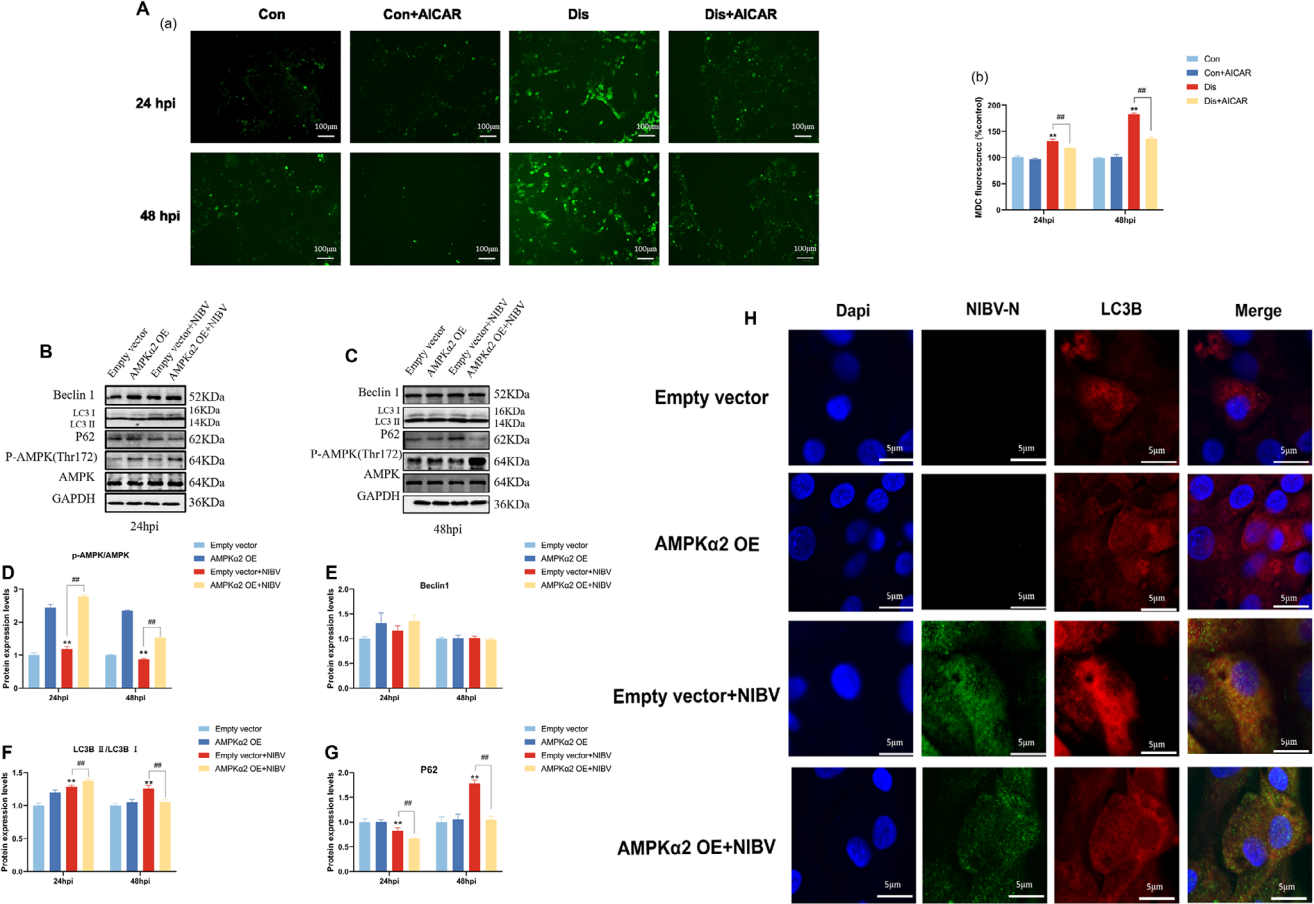
Activation of AMPK alleviates autophagic flux blockage, which reduces viral replication. (A) Effect of AMPK activation on cellular MDC levels. (a) Representative MDC fluorograms; (b) Representative MDC flow‐through analysis results, scale: 100 µm. (B‐G) Expression of AMPKα2 and cellular autophagy‐related proteins. Data are shown as the mean ± SD (n = 3). (H) Effect of overexpression of AMPKα2 on fluorescence co‐localization of LC3B and NIBV‐N in cells (48hpi). Blue fluorescence: Nuclear staining (DAPI), red fluorescence: LC3B staining, green fluorescence NIBV‐N staining, scale: 5 µm.

To avoid interference with GFP fluorescence in the plasmid, the AMPK activator AICAR was used to increase AMPK activity (Figure S3A). Examination of cellular MDC levels revealed that activation of AMPK significantly reduced the increase in MDC levels due to NIBV infection (Figure 7A). The overexpression of AMPKα2 differentially reduced the expression of autophagy vesicle generation‐related genes and proteins Beclin1 and LC3, and decreased the abnormal increase in P62. It alleviated the blockade of autophagic flux in renal tubular epithelial cells caused by NIBV infection and reduced the accumulation of autophagosomes (Figure 7B–G). Furthermore, compared with that in the empty vector+NIBV group, the fluorescence colocalization of LC3B and NIBV‐N was reduced in the AMPKα2 OE+NIBV group (Figure 7H). Moreover, overexpression of AMPKα reduced the replication of NIBV (Figure S3B).

Subsequently, AMPK expression was knocked down using siAMPK and the AMPK inhibitor Compound C to observe its effects on autophagy flux and viral replication, further revealing AMPK's regulatory role. We found that AMPK regulates autophagy flux, and when AMPK activity was downregulated, autophagy flux blockage became more pronounced (Figure S4A,B). Assessment of the viral copy number of NIBV at each time point revealed that inhibition of AMPK activity, compared with that in the normal group, significantly increased viral replication in the cells (Figure S4C,D).

### 2.8 Overexpression of AMPKα2 Enhances Lysosomal Function and Increases Lysosomal Fusion With Autophagic Vesicles by Regulating TFEB

**FIGURE 8:**
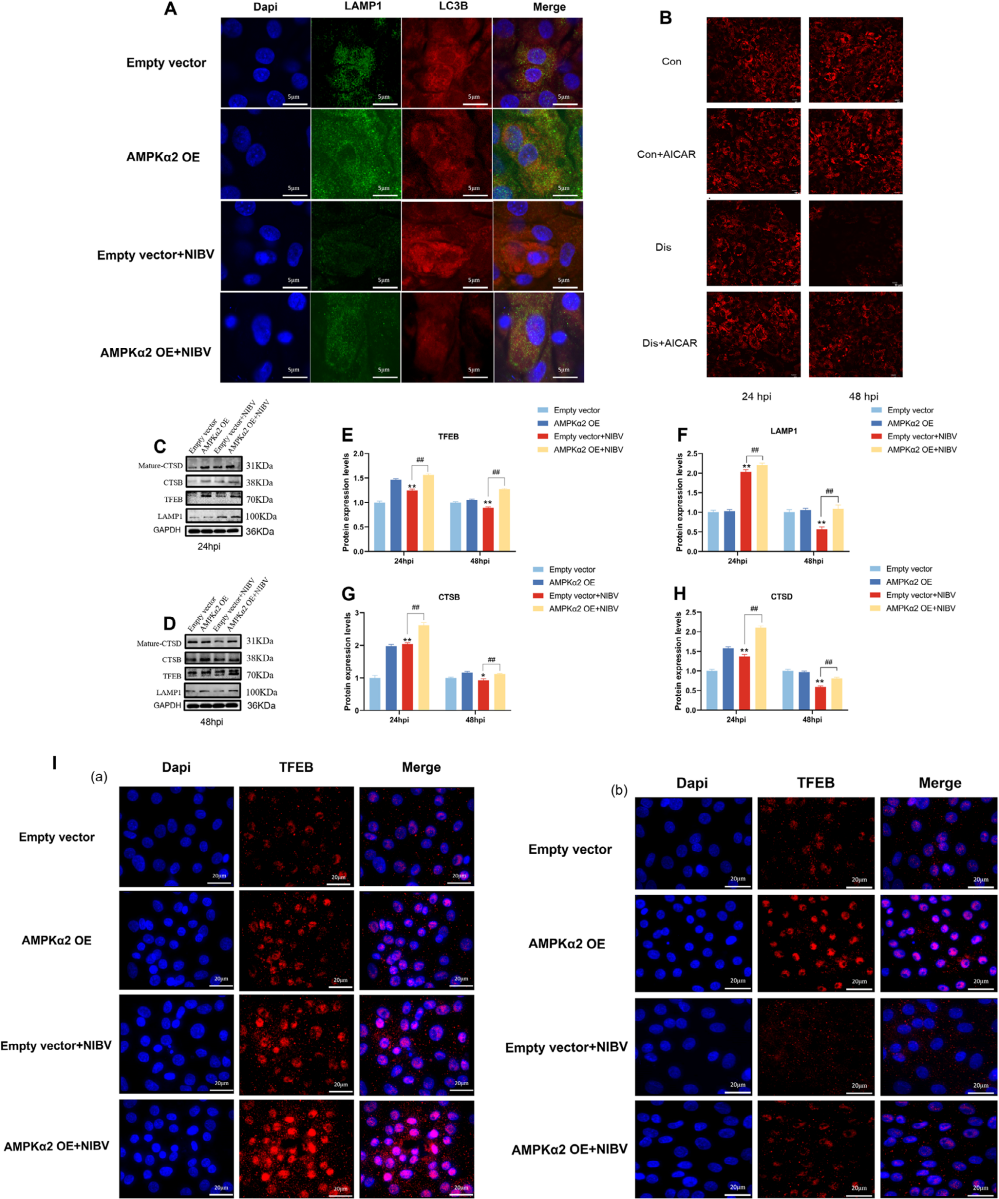
Activation of AMPK enhances lysosomal function and increases lysosomal fusion with autophagic vesicles by regulating TFEB. (A) Cellular LC3B and LAMP1 fluorescence co‐localization (48hpi). Blue fluorescence: nuclear staining (DAPI), red fluorescence: LC3B staining, green fluorescence: LAMP1 staining, scale: 5 µm. (B) Effect of AMPK activation on cellular lysosomal pH, scale: 10 µm. (C‐H) Cellular lysosome‐related protein expression results. Data are shown as the mean ± SD (n = 3). (I) Localized expression results of cellular TFEB immunofluorescence. (a): 24 hpi TFEB immunofluorescence localization results. (b): 48 hpi TFEB immunofluorescence localization results. Blue fluorescence: nuclear staining (DAPI), red fluorescence: TFEB staining, scale: 20 µm.

After the overexpression of AMPKα2, the fluorescence intensity of LAMP1 was restored, and the fluorescence colocalization of LC3B and LAMP1 increased (Figure 8A). Additionally, the activation of AMPK ameliorated the lysosomal alkalinization of renal tubular epithelial cells caused by NIBV infection (Figure 8B). The overexpression of AMPKα2 alleviated the suppression of lysosome function‐related proteins caused by NIBV infection (Figure 8C–H). In addition, overexpression of AMPKα2 significantly increased and promoted the transcription of TFEB at 24 hpi and 48 hpi (Figure 8I). These results suggest that overexpression of AMPKα2 can enhance lysosomal function and increase the fusion of lysosomes with autophagic vesicles by regulating TFEB.

## 3 Discussion

Autophagy plays multiple roles in various viral infections, with either proviral or antiviral functions [6]. Increasing evidence suggests that coronavirus infection subverts and hijacks host autophagy flux at different steps to evade elimination [22]. In this study, we revealed that NIBV infection induced a blockade of autophagic flux in chick kidneys, which resulted in an increase in self‐replication accompanied by the downregulation of AMPK expression. However, overexpression of AMPK improved lysosomal function, reduced autophagosome accumulation, inhibited viral replication, and ultimately ameliorated NIBV‐induced kidney injury in chicks by upregulating TFEB expression.

In recent years, the assessment of autophagy levels has changed from being based on the generation of autophagic vesicles to being based on autophagic flux [23, 24]. The induction of autophagy begins with phagocytosis formation, which is regulated by autophagy‐associated (ATG) proteins. Among them, ATG5 is essential for the formation of autophagic vesicles and is a core gene in both classical and nonclassical autophagy [25]. ATG5 can promote the formation of autophagosome membranes by participating in the LC3–PE binding pathway. LC3 is dominated by the LC3B subunit, and its elevated expression may be due to the increased formation of autophagosomes as autophagy occurs or decreased degradation of autophagosomes, leading to the accumulation of autophagosomes [26, 27]. P62 binds to LC3 to form autophagosomes, which are degraded to autophagic lysosomes, triggering autophagic flux and accelerating the removal of protein aggregates [28]. P62 protein is depleted along with the degradation of autophagic vesicles, thus P62 expression is often regarded as one of the indicators that can reflect autophagic flux [29, 30]. Certain types of coronaviruses (e.g., SARS‐CoV‐2) can alter autophagic processes to enhance their ability to replicate [31]. Our study revealed that NIBV infection of the kidney resulted in elevated LC3B expression. At the beginning of viral infection, P62 levels decreased, indicating smooth autophagic flux, but at the peak of viral infection, P62 levels increased significantly, indicating blocked autophagic flux. MDC staining revealed that more autophagic vesicles aggregated in renal tubular epithelial cells at 36 hpi and 48 hpi. Immunofluorescence staining revealed that the NIBV‐N protein colocalized with LC3B and had the highest fluorescence intensity at the peak viral load, suggesting that autophagic vesicle production is associated with viral replication. The interaction between AMPK and the NIBV‐N protein indicated that the NIBV‐N protein could directly or indirectly regulate AMPK expression, leading to alterations in autophagic flux.

Lysosomes serve as the primary sites for cellular breakdown. Autophagic vesicles combine to produce autolysosomes to control cellular breakdown and maintain homeostasis inside an organism [32]. Blocked autophagic flux is associated with impaired lysosomal biogenesis or lysosomal membrane permeability [33, 34]. Research has demonstrated that influenza A virus and African swine fever virus (ASFV) cause lysosomal dysfunction, leading to impaired autophagy [35, 36]. Pancreatic LC3–LAMP1 colocalization is reduced in type 1 diabetic mice [37]. Similarly, we found that LC3B‒LAMP1 colocalization increased at the initiation of NIBV infection. At the peak of viral infection, LC3B‒LAMP1 colocalization decreased, suggesting that the fusion of autophagic vesicles with lysosomes was blocked at this time. Furthermore, this phenomenon was corroborated by tracking the dynamics of individual autophagosomes using laser confocal microscopy after transfection with the mCherry‐GFP‐LC3 dual‐fluorescent plasmid. The lysosomal lumen contains a large number of enzymes that function optimally in the pH range of 4.5–5. This acidic pH is maintained primarily by the enzyme v‐ATPase [38]. It has been suggested that the acidic environment of the lysosomal lumen activates lysosomal endohydrolases, such as CTSB and CTSD, which digest macromolecules including proteins, nucleic acids, lipids, and carbohydrates [39]. Hepatitis B virus causes reduced lysosomal degradation and accumulation of immature lysosomes by inhibiting lysosomal acidification [40]. During the early stages of NIBV infection, the activity and content of both CTSB and CTSD increased, which contributed to the degradation capacity of the lysosomal material. At the peak of NIBV infection, the activities and levels of CTSB and CTSD peaked, which affected the maturation process of protein hydrolases and thus hindered lysosomal function. Disruption of the acidic lysosomal environment was further illustrated by the lysosomal pH assay with the lysosomal probe LysoTracker Red in NIBV‐infected cells. TFEB, as a major transcription factor required for lysosome‐associated genes, activates lysosomal gene expression and promotes lysosomal biogenesis. During periods of stress, TFEB moves from the cytoplasm to the nucleus and influences the activity of specific genes that are involved in the generation of lysosomes [41]. At the peak of NIBV infection, TFEB expression and activity were significantly reduced, and TFEB translocation into the nucleus was reduced. Recent data indicate that β‐coronavirus infection can result in a decreased immune response as a result of altered TFEB expression and aberrant biosynthesis [42]. Thus, at the peak of NIBV infection, the blockade of renal autophagic flux in chicks was correlated with altered lysosomal function.

Viruses can stimulate AMPK, leading to increased intracellular ATP levels, triggering the development of lysosomal proteases that promote autophagic catabolism and inhibit viral replication [43, 44]. In addition to initiating autophagy, AMPK plays an important role in the later stages of autophagy and increases the expression of genes that promote lysosomal biogenesis [17]. At the peak of NIBV infection, the expression of p‐AMPK/AMPK decreased significantly, which is consistent with the trend of NIBV‐induced blockade of autophagic flux. Because changes in AMPK activity are closely related to autophagic flux [45], we chose to overexpress AMPK to clarify the role of AMPK in the mechanism through which NIBV induces cellular autophagy. Activation of AMPK expression was found to inhibit viral replication in a model of herpes simplex virus type 1 (HSV‐1) infection of endothelial cells [46]. Similarly, with increased levels of AMPK phosphorylation, autophagic flux was restored, and viral replication of NIBV was significantly reduced. Owing to the protein interactions between AMPK and TFEB, overexpression of AMPK could activate TFEB and promote its nuclear translocation, which in turn increased the activity of lysosome‐related genes and proteins, restoring lysosomal function. Recent investigations have shown that the AMPK inhibitor Compound C and AMPK shRNA effectively reduced the effect on mTOR signaling and even reversed the effects of TFEB nuclear translocation [47]. Our study revealed that NIBV can increase viral replication by inhibiting AMPK expression and thus autophagic lysosome production.

In conclusion, we found that the activation of AMPK could enhance lysosomal function, reduce the accumulation of autophagosomes, and inhibit the production of NIBV by modulating TFEB, alleviating renal injury caused by NIBV in chickens with gout. In addition, the NIBV‐N protein, which is a key component for viral replication, could interact with AMPK to suppress its expression, thereby inhibiting TFEB and preventing the degradation of autophagolysosomes. Moreover, the N protein colocalized and interacted with LC3B during NIBV replication, suggesting that autophagic vesicles or other autophagic vacuoles may provide a physical scaffold for NIBV replication. These findings provide a new therapeutic target for kidney injury caused by NIBV infection.

## 4 Methods

### 4.1 Antibodies and Reagents

The primary antibodies used in this experiment were as follows: Antisera against the NIBV‐N, LC3B, P62, and AMPKα proteins were obtained from rabbits immunized with bacterially expressed fusion proteins in our laboratory; antibodies against phospho‐AMPK alpha‐2 (Thr172) (Bioss, bs‐4002R, Beijing, China), Beclin1 (Omnimabs, OM186318, CA, USA), LAMP1 (Omnimabs, OM252100, CA, USA), CTSB (CST, #2357, MA, USA), CTSD (CST, #11818, MA, USA), mTOR (CST, #12252, MA, USA) and TFEB (CST, #5832, MA, USA) were obtained from the indicated sources.

### 4.2 Viral Strains and Viral Load Determination

The virulent strain of NIBV (SX9, accession number: MN707951.1) used in this study was isolated and preserved at the College of Animal Science and Technology, Jiangxi Agricultural University. The absence of additional pathogens was verified through the use of reverse transcription PCR (RT‒PCR) and bacterial culture. Highly pathogenic NIBV strains were cultured in 10‐day‐old SPF embryo eggs. The infected embryos were then analyzed for particular pathologies associated with IBV, such as developmental delay or coiling. The Reed–Muench method was used to compute the embryo infective dose (EID50). The pMD18‐T‐N‐positive plasmid was subsequently constructed using the pMD18‐T vector (Takara Bio, Dalian, China), a standard curve was constructed, and the viral loads in the kidney and tubular epithelial cells of chicks at different times of infection were determined by absolute quantitative PCR.

### 4.3 Animal Experiments and Sample Collection with Preservation

Three hundred 1‐day‐old chicks were randomly selected and immunized according to the immunization procedure for laying hens (with the exception of the infectious bronchitis virus vaccine) and fed until 21 days of age. One hundred chicks were randomly selected as the normal group (Con group), 200 chicks were randomly selected as the NIBV model group (Dis group), and the chicks were grouped into separate rooms for rearing. After 7 days of acclimatization, the chicks in the Con group were inoculated with 0.2 mL of sterile saline by eye and nose drops, while the chicks in the Dis model group were inoculated with 0.2 mL of 10^5^ ELD50 virus solution by eye and nose drops. The experiment lasted for 28 days, during which time both groups of chicks were provided with sufficient feed and water for free feeding. This experiment was carried out in strict accordance with the national regulations on experimental animal ethics, the principles of experimental animal welfare and the safety of animal experiments and was approved by the Animal Management and Ethics Committee of Jiangxi Agricultural University (JXAU) (No. JXAULL‐2021‐12).

On the first, fifth, 11th, 18th, and 28th dpi of the experimental period, 8 chicks were randomly selected from the normal group and the infected group. They were fasted and dehydrated for 12 h in advance, followed by weighing, and after euthanasia, the kidneys were weighed, and kidney tissue and blood samples were collected. Some of the kidney samples were frozen in liquid nitrogen and stored at ‐80°C for viral load and proteomics determination. Some of the kidney samples were sampled and preserved in 4% paraformaldehyde fixative and electron microscopy fixative for sectioning and transmission electron microscopy observation. Blood samples were incubated at 37°C for 2 h and centrifuged at 3000 r/min for 10 min, and the serum was separated and stored in portions at ‐80°C for the detection of uric acid (Meikang Bio, Ningbo, China) and creatinine (Meikang Bio, Ningbo, China) levels in serum.

### 4.4 Isolation and Culture of Primary Chicken Renal Tubular Epithelial Cells

After 12 h of fasting and water fasting, 1–7‐day‐old Hyland Brown chicks were euthanized, and the kidneys were removed. Kidneys were placed in low‐sugar DMEM containing 5% penicillin and streptomycin (Solarbio, Beijing, China), and connective tissues and the fascia were removed, after which the kidneys were repeatedly fragmented into 1–2 mm^3^ pieces. Prewarmed 1% type I collagenase (Solarbio, Beijing, China) solution was added, and the samples were allowed to digest in a constant‐temperature water bath at 37°C for 6 min for 30 s. The digestion was terminated by adding an equal volume of 10% FBS (Kitai Ikosai Bio, Shanghai, China) cell culture solution. A pipette gun was used to gently blow repeatedly until smooth. The cell suspension was filtered using a 200‐mesh mesh sieve and dispensed into 10 mL EP tubes at 1000 r/min for 6 min, and the centrifugation was repeated three times, with the second centrifugation using erythrocyte lysate (Solarbio, Beijing, China) for lysis of erythrocytes in the cell sediment. At the end of centrifugation, a 10% FBS cell culture solution was used to resuspend the cell precipitates, and the cells were spread on plates at a density of 1 × 10^6^ cells/mL. The cell culture plates were incubated in a cell culture incubator at 37°C with 5% CO_2_.

### 4.5 AMPK Activation and Inhibition and Construction of the Eukaryotic Expression Plasmid pcDNA3.1‐AMPK‐P2A‐eGFP

The cell density was adjusted to 4–5 × 10^5^ cells/mL, the cells were spread in 96‐well plates at 100 µl/well, and the cell culture plates were incubated in a cell culture incubator at 37°C with 5% CO_2_. When the density of the cultured cells was 70–80%, the supernatant was discarded, the cells were washed with PBS, and the diluted AMPK activator (AICAR) (MCE, NJ, USA) and inhibitor (Compound C) (Sigma‒Aldrich Biology Chemistry St. Louis, Missouri, USA) were added, with 8 replicate wells for each dilution concentration. At the end of the incubation period, 10 µL of CCK‐8 solution was added to each well and incubated at 37°C in the dark for 2 h. The absorbance value at 450 nm was measured using an enzyme marker (Biotech, Vermont, USA), and the optimal concentrations of the AMPK activator and inhibitor were determined according to the analysis results.

The plasmid sequence was designed on the basis of the gene sequence of AMPKα2 published by NCBI (NM_001039605.2), and plasmid synthesis was performed by Nanjing Kingsley Biotechnology Co. Finally, fluorescence microscopy, qRT‒PCR, and WB were used to determine the overexpression efficiency of AMPKα2.

### 4.6 NIBV Infection of Primary Chicken Renal Tubular Epithelial Cells and In Vitro Experimental Groups

The primary chicken renal tubular epithelial cell suspension was adjusted to a seeding density of 1 × 10^6^ cells/mL for plating. The cell culture plates were placed in a cell culture incubator at 37°C and 5% CO_2_ for culture. After 24 h, the medium was changed, and the cells were cultured until they reached 70–80% confluence. The supernatant was discarded, and the cells were washed with PBS. Viral hyaluronic acid solution was added at an MOI of 1, and the cells were incubated at 37°C for 2 h, with shaking every 30 min. The viral solution was discarded, and 2% FBS cell culture medium was added. The cells were cultured at 37°C in a 5% CO_2_ incubator. In accordance with the experimental objectives, the cells were grouped as follows:

1. Control group (Con group) and NIBV infection group (Dis group), with sampling times of 12, 24, 36, and 48 h postinfection (hpi).
2. pcDNA3.1‐eGFP plasmid group (empty vector group), pcDNA3.1‐AMPK‐P2A‐eGFP plasmid group (AMPKα2 OE group), pcDNA3.1‐eGFP plasmid + NIBV group (empty vector + NIBV group), and pcDNA3.1‐AMPK‐P2A‐eGFP plasmid + NIBV group (AMPKα2 OE + NIBV group), with sampling times at 24 and 48 hpi.
3. Control group (Con group), control + activator group (Con+AICAR group), NIBV infection group (Dis group), and NIBV infection + activator group (Dis+AICAR group); sample collection times were 24 and 48 hpi.
4. Control group (Con group), control + inhibitor group (Con+Compound C group), NIBV infection group (Dis group), and NIBV infection + inhibitor group (Dis+Compound C group); the sample collection times were 12, 24, 36, and 48 hpi.

### 4.7 Proteomics Analysis

Kidney samples from the normal and NIBV groups were analyzed by mass spectrometry at 11 dpi by Guangdong KDO Biotechnology. The samples were analyzed after being transported on dry ice: first, the proteins in the samples were measured with a BCA kit, and then the protein quality was assessed by SDS‒PAGE. Each 100 µg sample of protein was digested with protease to obtain peptides, and the concentration was subsequently measured with a Pierce Peptide Concentration Kit (Thermo Fisher Scientific, MA, USA). Next, the peptides were analyzed using LC‒MS/MS (Orbitrap Fusion Lumos) (Thermo Fisher Scientific, MA, USA), and the data were processed using Spectronaut software (Biognosys AG, Zurich, Switzerland). Differentially expressed proteins (|fold change| ≥ 1.5, *p* < 0.05) were annotated using the GO and KEGG databases.

### 4.8 Histopathological Observations, HE Staining, and LysoTracker Red Staining

Kidney tissues were preserved in 4% paraformaldehyde, dried with xylene, and then embedded in paraffin. The tissues were sliced into sections with a thickness of 5 µm and then subjected to staining using hematoxylin and eosin (H&E) (Servicebio, Wuhan, China) following established histological protocols. The slides were evaluated using a light microscope (Nikon, Tokyo, Japan).

After chicken primary renal tubular epithelial cells were infected with NIBV, the cells were washed with PBS buffer, and 1 mL of medium containing 50 nM LysoTracker Red probe (Beyotime, Shanghai, China) was added to each well and incubated for 30 min at 37°C in the dark. After the cells were incubated, the cell culture medium was added, and the intensity of lysosomal red fluorescence of the cells was assessed using a laser confocal imaging camera (Olympus, Tokyo, Japan) to determine the intensity of lysosomal red fluorescence.

### 4.9 TEM Imaging

After the chicken primary renal tubular epithelial cells were treated, the cells were washed three times with PBS, and an electron microscopy fixative was added for 15 min, and the cells were resuspended and prepared as cellular electron microscopy samples. Renal tissue and cellular electron microscopy samples were fixed with 1% osmium acid for 2 h at room temperature, protected from light, rinsed sequentially with PBS, dehydrated with an alcohol gradient, and then dehydrated with acetone. After dehydration, the samples were osmotically embedded in acetone and an embedding agent. After embedding, the samples were stained with 2% uranyl acetate solution and 2.6% lead citrate solution, and the images were observed under a transmission electron microscope (JEOL, Tokyo, Japan).

### 4.10 RNA Extraction and Quantitative Real‐Time PCR (qRT‒PCR) Analysis

Total RNA was isolated from kidney tissues and cell samples using TRIzol reagent (Vazyme, Nanjing, China). The original quality and abundance of the whole RNA samples were evaluated using a Thermo NanoDrop 2000 spectrophotometer (Thermo, Waltham, USA). cDNA was reverse transcribed into cDNA using a HiScript II first Strand cDNA Synthesis Kit (Vazyme, Nanjing, China). cDNA was detected using the CFX384 Touch Real‐Time PCR Detection System (Bio‐Rad Laboratories, CA, USA) with a ChamMax RNA Analyzer (Bio‐Rad Laboratories, Waltham, MA, USA). qRT‒PCR was performed using a CFX384 Touch Real‐Time PCR Detection System (Bio‐Rad Laboratories, CA, USA) according to the instructions of ChamQ SYBR qPCR Master Mix (Vazyme, Nanjing, China). The absolute quantification method was used to determine the precise quantity of copies of NIBV RNA. The mRNA expression of the target gene was normalized to that of GAPDH, and the qRT‒PCR results were analyzed using the comparative 2^−ΔΔCt^ method. The primers used in the experiments are listed in Table S1.

### 4.11 Total Protein Extraction and Western Blot Analysis

Total kidney and cell sample proteins were extracted on ice with a mixture of RIPA lysis buffer (Solarbio, Beijing, China), phosphatase inhibitor (100X; CWBIO, Taizhou, China), and protease inhibitor (100X; Solarbio, Beijing, China). A BCA protein assay kit (Solarbio, Beijing, China) was used to determine the protein concentration. Subsequently, 10 µg of protein was subjected to 10% SDS‒PAGE and subsequently transferred to a 0.45 µm PVDF (Millipore Corp., MA, USA) membrane. The membrane was blocked using a 5% solution of skim milk for a duration of 2 h at ambient temperature. Next, the PVDF membranes were incubated with primary antibodies overnight at 4°C. Following the washing process, the membrane was incubated with the appropriate secondary antibody for a duration of 1 h. Finally, a ChemiDoc imaging system (Bio‐Rad Laboratories, CA, USA) and enhanced chemiluminescence reagent (Bio‐Rad Laboratories, CA, USA) were used to detect the bands. Bands were analyzed using ImageJ software (NIH, Bethesda, USA).

### 4.12 Immunoprecipitation of AMPK with the Viral N Protein and TFEB

After NIBV infection of chicken primary renal tubular epithelial cells, the supernatant was discarded, and the cells were subsequently washed with PBS. The cells in the six‐well plates were lysed by the addition of RIPA lysis solution to each well on ice for 5 min, scraped repeatedly until the cells were dislodged, and shaken horizontally at low speed for 30 min at 4°C to fully lyse the samples. The samples were placed in a high‐speed cryo‐centrifuge (Thermo, Waltham, USA) and centrifuged at 12 000 g and 4°C for 15 min, after which the supernatant was removed. Protein A agarose was washed with PBS, adjusted to a 50% concentration, added to the cell samples (1 mL of total protein: 100 µL of 50% Protein A agarose beads), and oscillated at a low speed for 10 min at 4°C. The supernatant was centrifuged for 15 min at 12 000 g at 4°C. The concentration of the protein sample was determined using a BCA protein concentration assay kit, and the sample concentration was adjusted to 1 µg/µL. The corresponding antibody was added to the sample, and the sample was incubated on a shaker at 4°C overnight. After incubation, 100 µL of Protein A agarose beads was added, and the mixture was shaken horizontally at low speed for 1 h at 4°C. The mixture was centrifuged at 12 000 × g for 5 min at 4°C, after which the supernatant was discarded; the pellet was washed three times with RIPA lysis buffer, followed by addition of protein sampling buffer, gentle mixing, incubation in a water bath for 15 min at 100°C, and storage at ‐20°C.

### 4.13 Immunofluorescence Staining and MDC Detection

The prepared paraffin sections of kidney tissue were deparaffinized. Cells were inoculaterminated usings and grown to 50–60% adherence; the infection was terminated using precooled 4% paraformaldehyde fixative and incubation in a membrane‐breaking working solution after fixation. After antigen repair, 3% BSA was added dropwise, and the membrane was closed for 30 min. The corresponding primary antibody was added dropwise, and the membrane was incubated at 4°C overnight; the corresponding fluorescence‐labeled secondary antibody was added, and the membrane was incubated at room temperature for 50 min in the dark. The nuclei of the cells were restained with DAPI, and the membrane was incubated at room temperature for 10 min in the dark; an auto fluorescent quencher was added for 5 min, and the membrane was washed for 10 min, followed by sealing using anti‐fluorescence‐quenching sealer (Solarbio, Beijing, China). Observations were subsequently performed using an orthogonal fluorescence microscope (Nikon Corporation, Tokyo, Japan).

At the end of NIBV infection of the chicken primary renal tubular epithelial cells, the cells were washed with PBS buffer and digested by adding 0.25% trypsin (Solarbio, Beijing, China) at 37°C for 5 min. The digestion was terminated by adding an equal amount of 10% FBS cell culture solution at 1000 r/min for 5 min. Next, 1 mL of medium containing 50 µM MDC (Beyotime, Shanghai, China) was added, mixed well by blowing, and incubated at 37°C for 30 min in the dark, with the top and bottom turned up and down every 5 min. At the end of the incubation period, the cells were centrifuged at 1000 r/min for 5 min, the supernatant was discarded, and the precipitate was resuspended by the addition of an appropriate amount of PBS, avoiding light during the entire process. The MDC content of the cells was detected by flow cytometry (BD Medical Equipment Co., Shanghai, China), and the fluorescence intensity of MDC was detected by a fluorescence inverted microscope (Caikon Optical Instruments Co., Shanghai, China).

### 4.14 Statistical Analysis

With the exception of the proteomics data, the remaining data in this study were statistically analyzed by SPSS 25.0 software. Independent samples t tests were used to compare the differences between the two groups, and one‐way analysis of variance (ANOVA) was used to compare the differences between multiple groups. The data are displayed as the mean±SD and were plotted by GraphPad Prism 8.0 software. *P > 0.05* indicated that the data were not significantly different, *P < 0.05* (*) indicated that the results were significantly different, and *P < 0.01* (**) indicated that the results were highly significantly different.

## Author Contributions

C.H. contributed to conceptualization, methodology, formal analysis, data curation, visualization, and writing of the original draft. Y.Z. was responsible for validation, formal analysis, data curation, visualization, and writing of the original draft. Z.L. contributed to validation, data curation, and formal analysis, while S.M.O. was involved in validation and formal analysis. Y.C. contributed to validation and data curation, and S.Z. provided resources, validation, and visualization. P.L., Z.Z., and G.C. contributed to methodology, supervision, and writing – review and editing. X.G. and X.G. served as corresponding authors and were responsible for funding acquisition, project administration, supervision, resources, and conceptualization. All authors contributed to the article and approved the submitted version, and the support sources had no restrictions on publication.

## Conflicts of Interest

The authors declare no conflicts of interest.

## Supporting information




**Supporting File**: advs75104‐sup‐0001‐SuppMat.docx.

## Data Availability

The data that support the findings of this study are available from the corresponding author upon reasonable request.
