# Supplementary material for: NIBV Induces Incomplete Autophagy via AMPK‐TFEB, Causing Kidney Injury in Chicks
Source: Adv Sci (Weinh). 2026 Apr 9;13(34):e14993. doi: 10.1002/advs.202514993 (PMC13285144; doi:10.1002/advs.202514993)
Supplement: Supplementary file 1 — Supporting File: advs75104‐sup‐0001‐SuppMat.docx. [file ADVS-13-e14993-s001.docx]

| 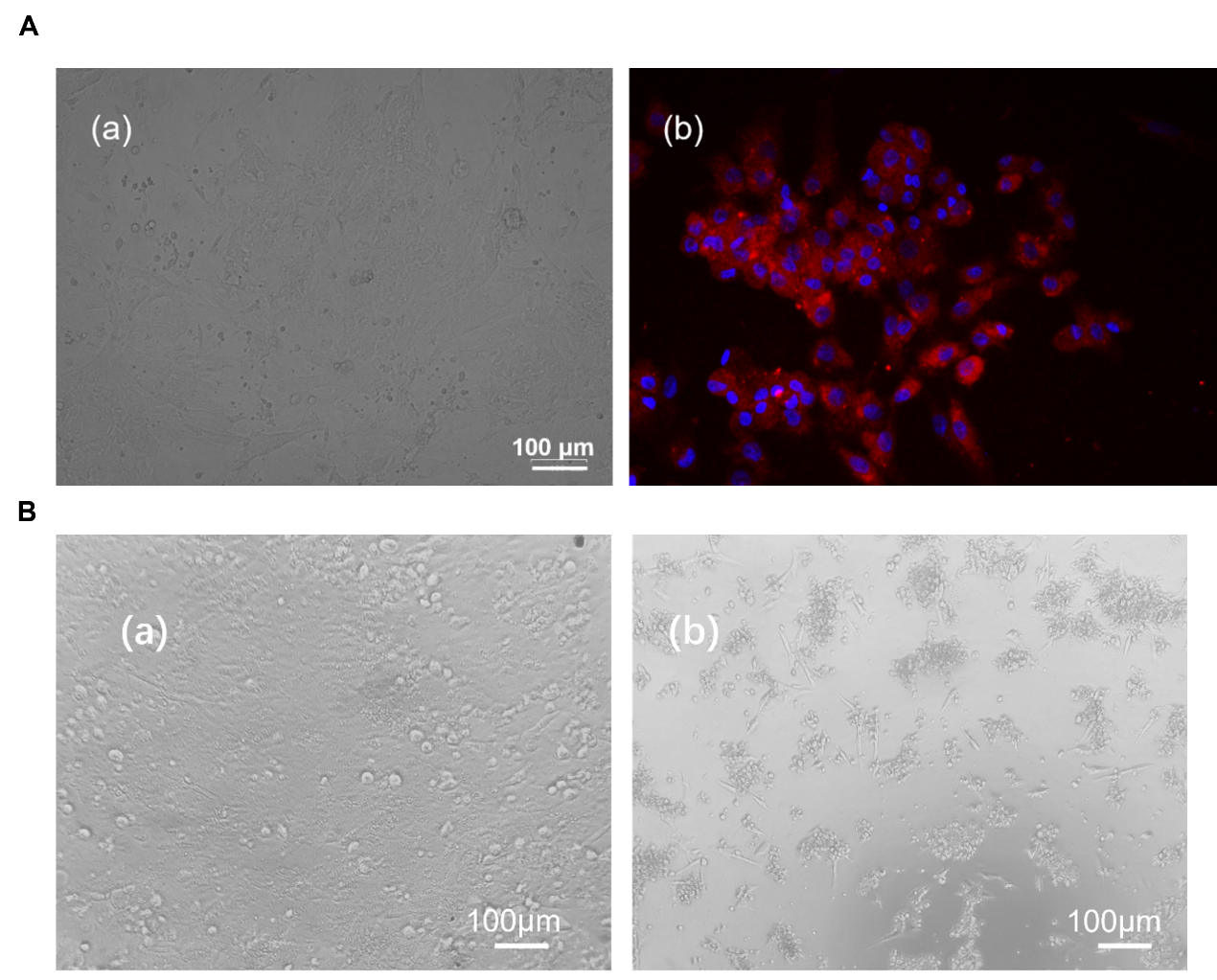 |
| --- |
| **Figure.S1** (A) Identification results of chicken primary renal tubular epithelial cells. (a) Chicken primary renal tubular epithelial cells, scal:100 μm; (b) results of CK18 immunofluorescence staining of chicken primary renal tubular epithelial cells, 400×. (B) NIBV-infected chicken primary renal tubular epithelial cells. (a) Normal chicken primary renal tubular epithelial cells, scale:100 μm.(b) 10^-3^ of NIBV allantoic fluid infected chicken primary renal tubular epithelial cells, scale:100 μm. |
| **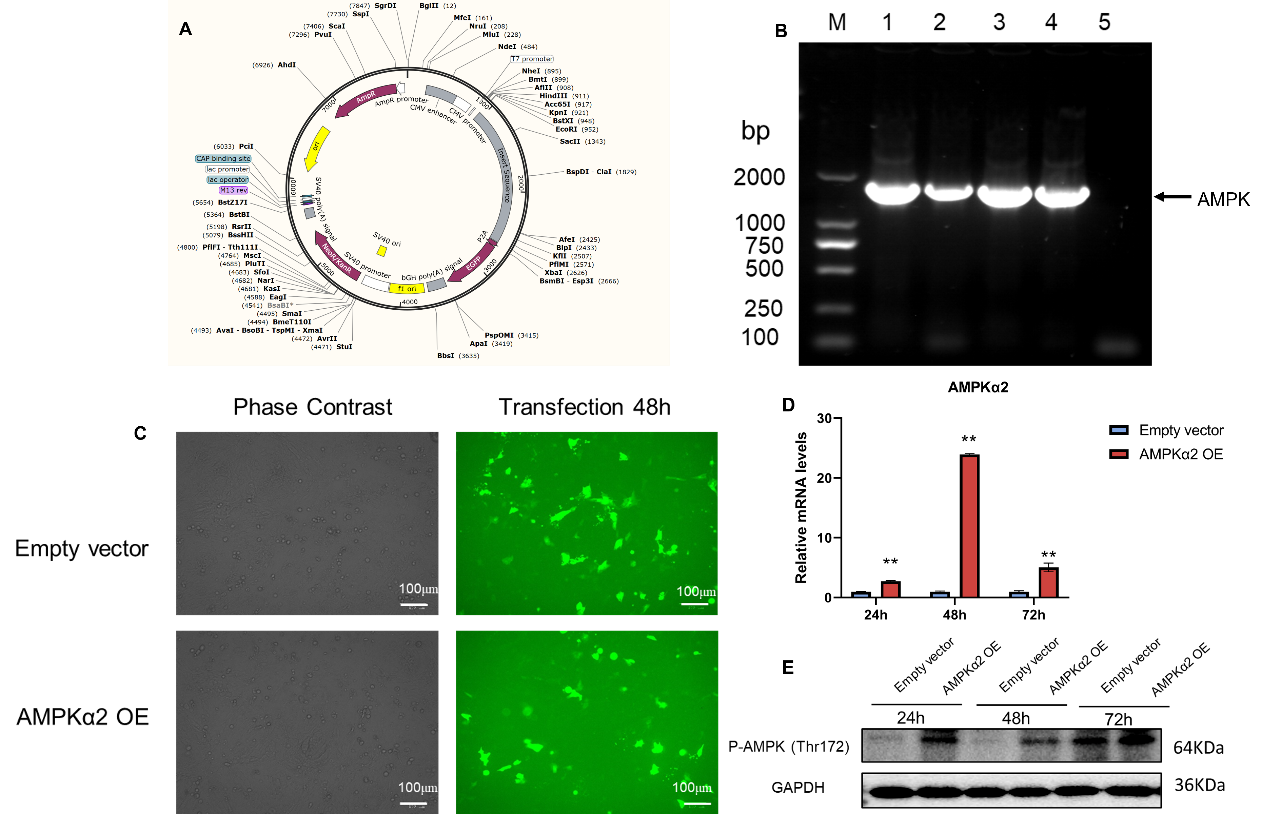** |
| **Figure.S2** (A) Plasmid map of pcDNA3.1-AMPK-P2A-eGFP. (B) Results of pcDNA3.1-AMPK-P2A-eGFP nucleic acid electrophoresis. Lanes 1-4: bacteriophage PCR products of eukaryotic expression plasmid pcDNA3.1-AMPK-P2A-eGFP; lane 5: negative control; lane M: DNA Marker. (C) Transient expression of AMPKα2 overexpression plasmid, scale:100μm. (D) Efficiency of AMPKα2 overexpression detected by qRT-PCR. (E) Western Blot Detection of AMPKα2 Overexpression Efficiency. |
| 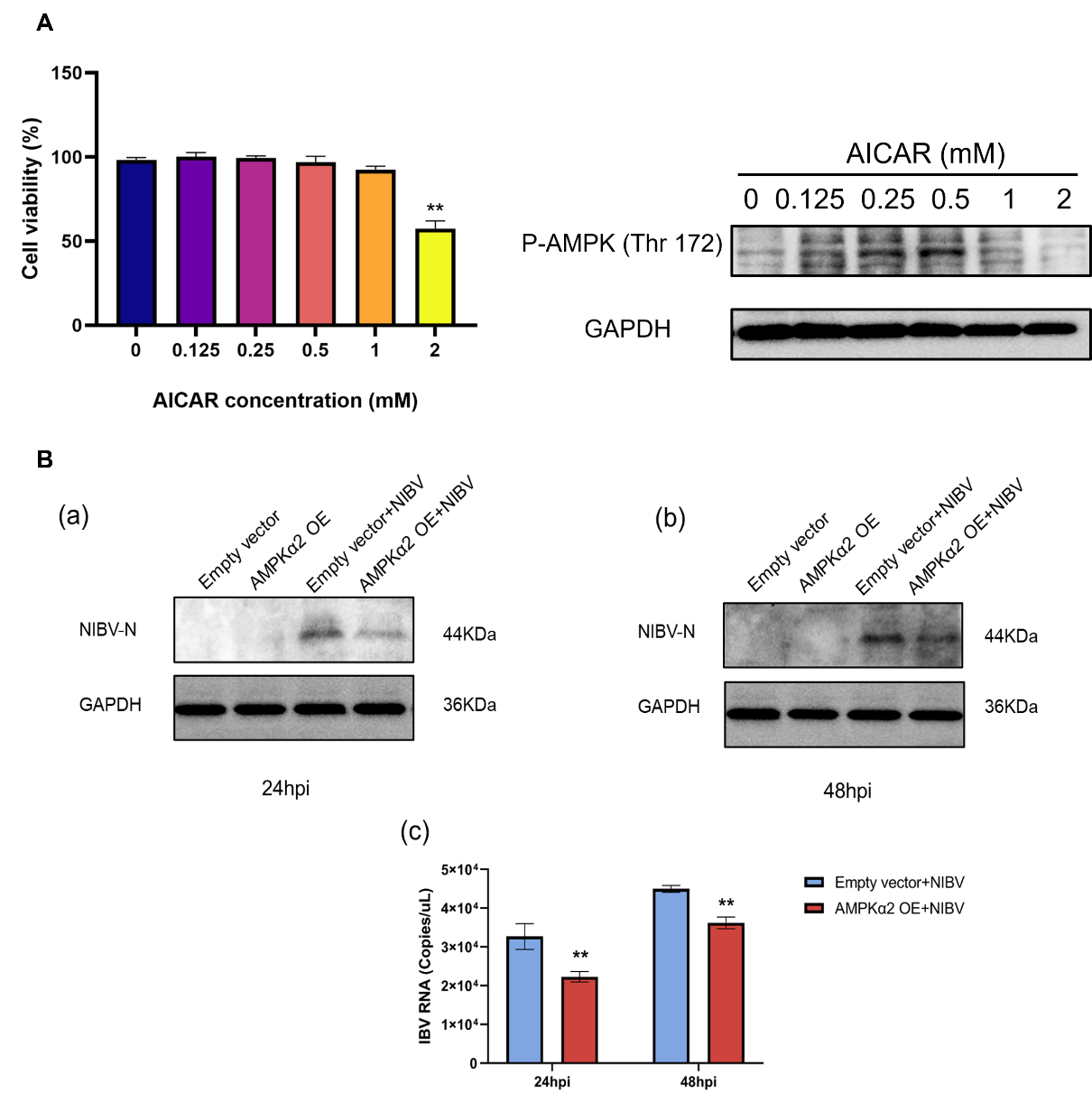 |
| **Figure.S3** (A) Determination of the optimal intervention concentration of the AMPK activator AICAR. (B)Effect of overexpression of AMPKα2 on NIBV virus replication. (a): 24hpi, effect of overexpression of AMPKα2 on NIBV-N protein expression. (b): 48hpi, effect of overexpression of AMPKα2 on NIBV-N protein expression. (c): Effect of overexpression of AMPKα2 on NIBV-N gene expression. |
| **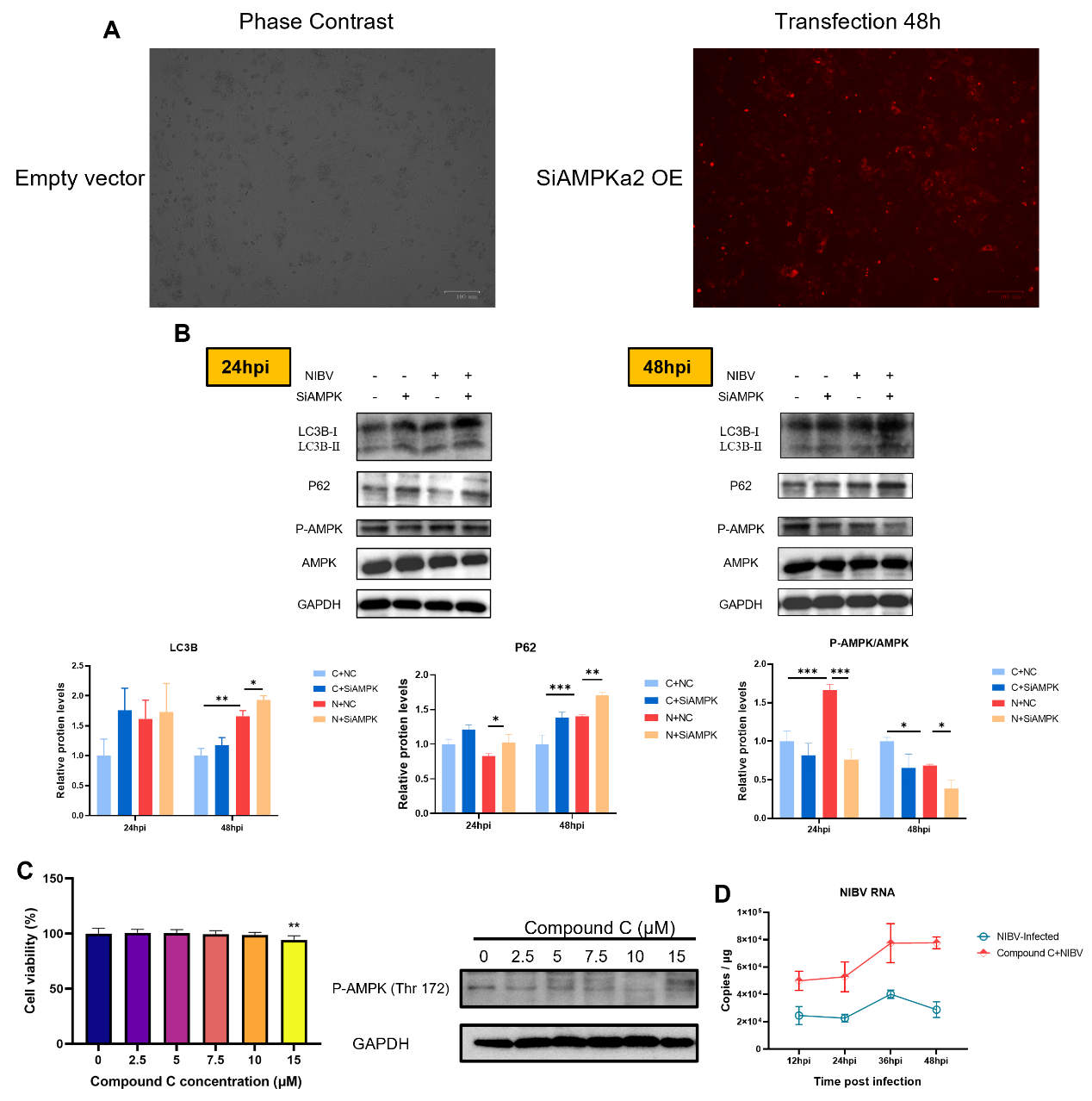** |
| **Figure.S4** (A) Transient expression of the siAMPKα2 plasmid. (B) Effects of altered AMPK activity on autophagic flow-associated proteins. (C) Determination of the optimal intervention concentration of the AMPK inhibitor Compound C. (D) Effect of inhibition of AMPK activity on NIBV viral load. |

| NIBV-N | MN707951.1 | F: 5'-GGTAGYGGYGTTCCTGATAA-3'  R: 5'-TCATCTTGTCRTCACCAAAA-3' |
| --- | --- | --- |
| Beclin1 | NC_052558.1 | F:5’- CGACTGGAGCAGGAAGAAG-3’  R:5’- TCTGAGCATAACGCATCTGG -3’ |
| LC3A | XM_040688401.2 | F:5’-TCCTTGTCCCAGACCATGTC-3’  R:5’-GCCATCCTCATCCTTCTCCT-3’ |
| LC3B | NM_001031461.1 | F:5’-GTACGAGAGCGAGAAGGACG-3’  R:5’-GGGTGATGGCCAAGGGTAAA-3’ |
| P62 | XM_003642061.6 | F:5’- CTCTCGCTGGACTCTCTCTG-3’  R:5’- ATGCTTGTGTCGTGGGTAGA -3’ |
| AMPKα2 | NM_204305 | F:5’-TGTCTTACTGCAGATGCCCC-3’  R:5’- GCCAGAGGGGGAGATACCAT-3’ |
| mTOR | XM_040688401.2 | F:5’- AGCGTGCCTACCTTCTTCTT-3’  R:5’- CGGTCATCACGGTTCATTCC -3’ |
| ATG5 | NM_001006409.2 | F:5’-CTGCCATTCTAAGGATGTGA-3’  R:5’-AGTTTTCGATTTATAGCCCAA-3’ |
| TFEB | NM_001030922.1 | F:5’-GTTGCTTCGGTGTCCTGGAG-3’  R:5’-CAGCACGCACCCAAAGAATC-3’ |
| ATP6V1A | NM_001389289.2 | F:5’-GAGCGCCGCTTCGTTGAA-3’  R:5’-AGTTCACTGTGGCCTACTCG-3’ |
| ATP6V1B | NM_001293241.2 | F:5’-GGCTCTAACAACGGCAGAGT-3’  R:5’-TAACCTGGGAAACCACGACG-3’ |
| LAMP1 | NM_001397639.1 | F:5’-ACGTGACTGGTGCAAATGGA-3’  R:5’-AGCCAGATTCAAGAAGGCAGA-3’ |
| LAMP2 | NM_001001749.1 | F:5’-TACCGTGTGGCTTTTGGAGA-3’  R:5’-TCACACGGACAGACGTAACC-3’ |
| CTSB | NM_205371.3 | F:5’-CCCCTCCTGCCTGTGTTAAG-3’  R:5’-AGGCCCTTCCCTAGGATCAA-3’ |
| CTSD | NM_205177.2 | F:5’-ACTCGGTGGATGTTGCCAAT-3’  R:5’-GTGACAACAGGCAGAGACGA-3’ |
| GAPDH | NM_204305.1 | F:5′-TGGCATCCAAGGAGTGAGC-3′  R:5′-GGGGAGACAGAAGGGAACAG-3′ |
| **Table. S1** Primer Sequences. | | |
| **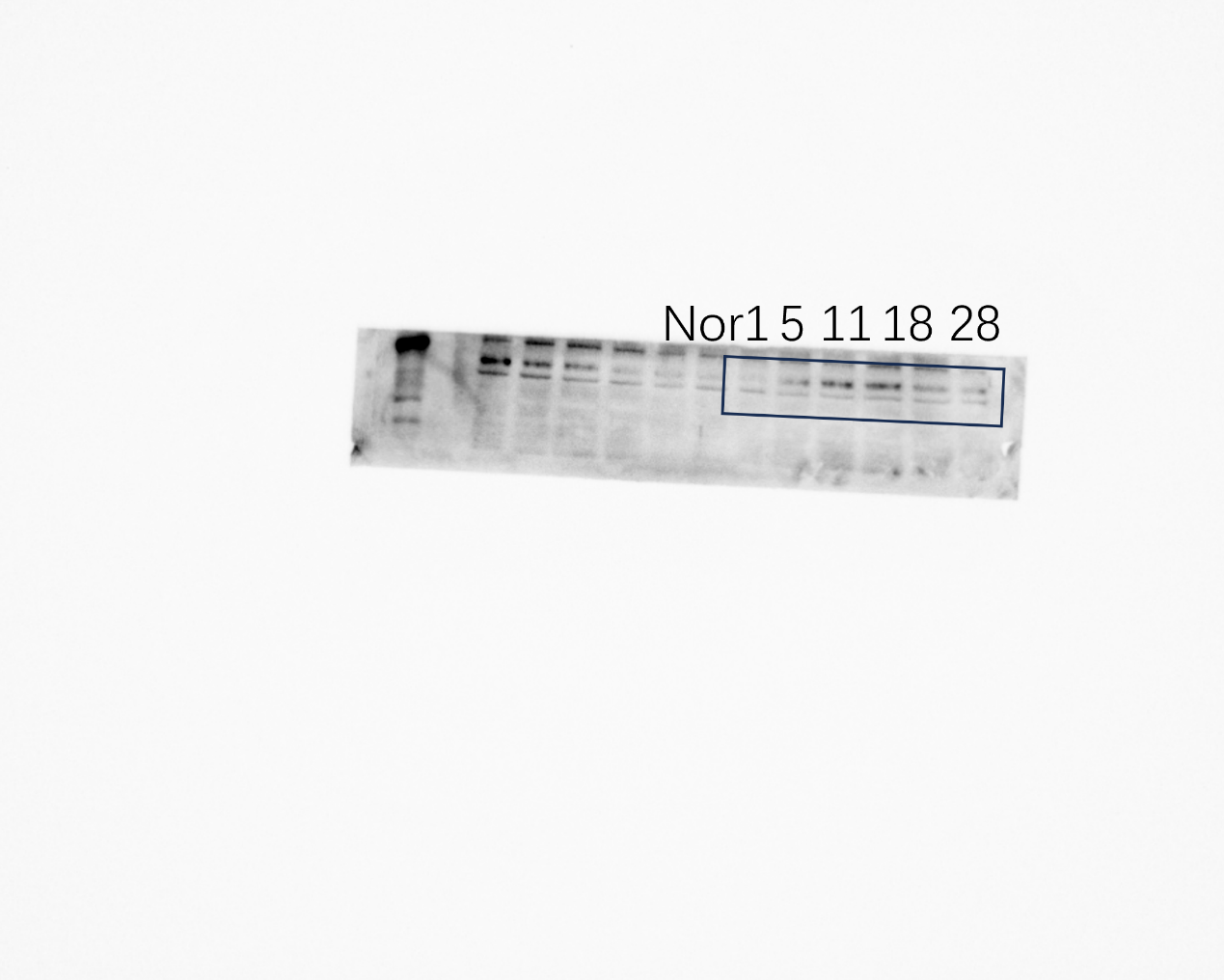** | | |
| **Figure.1 (E)** | | |
| **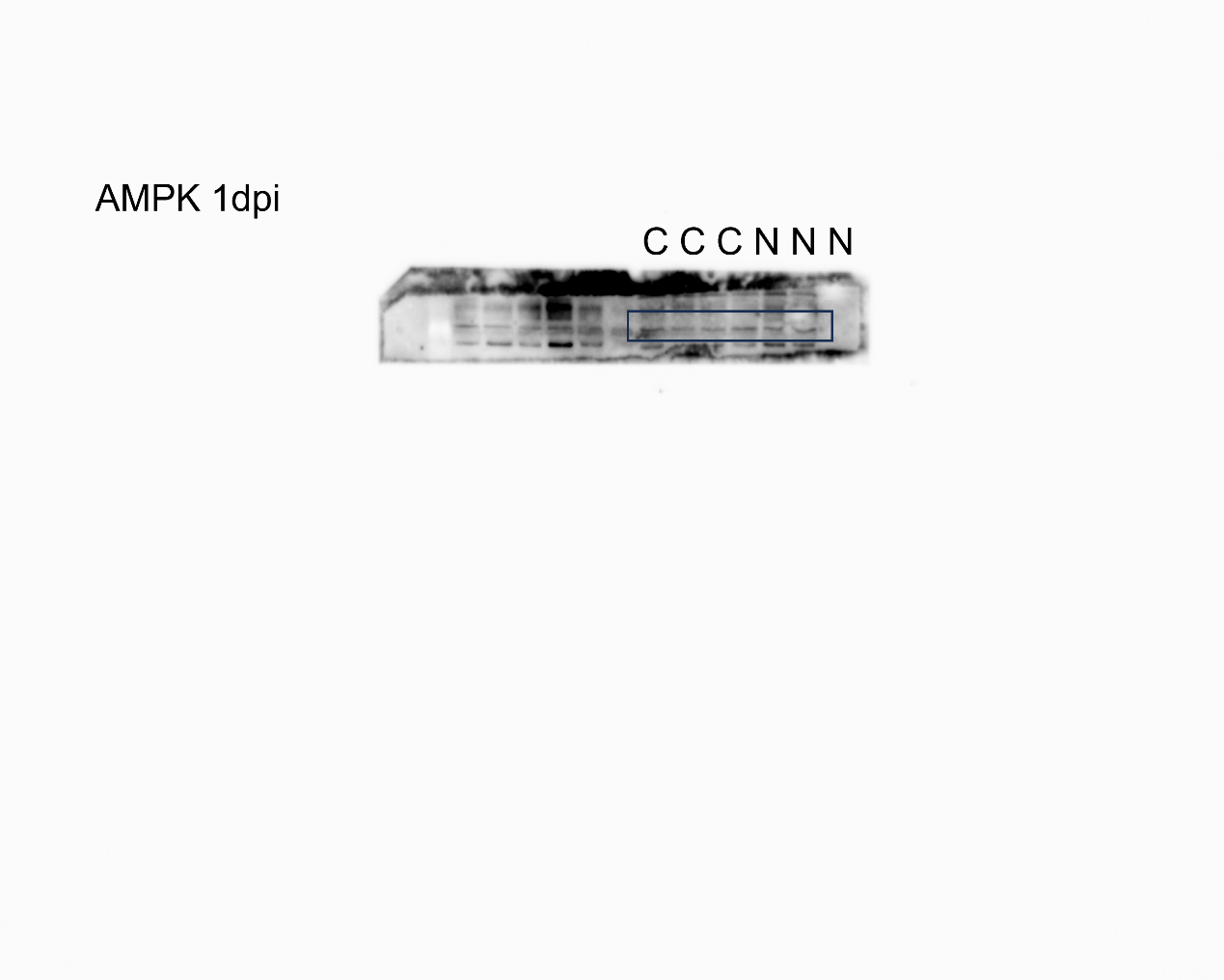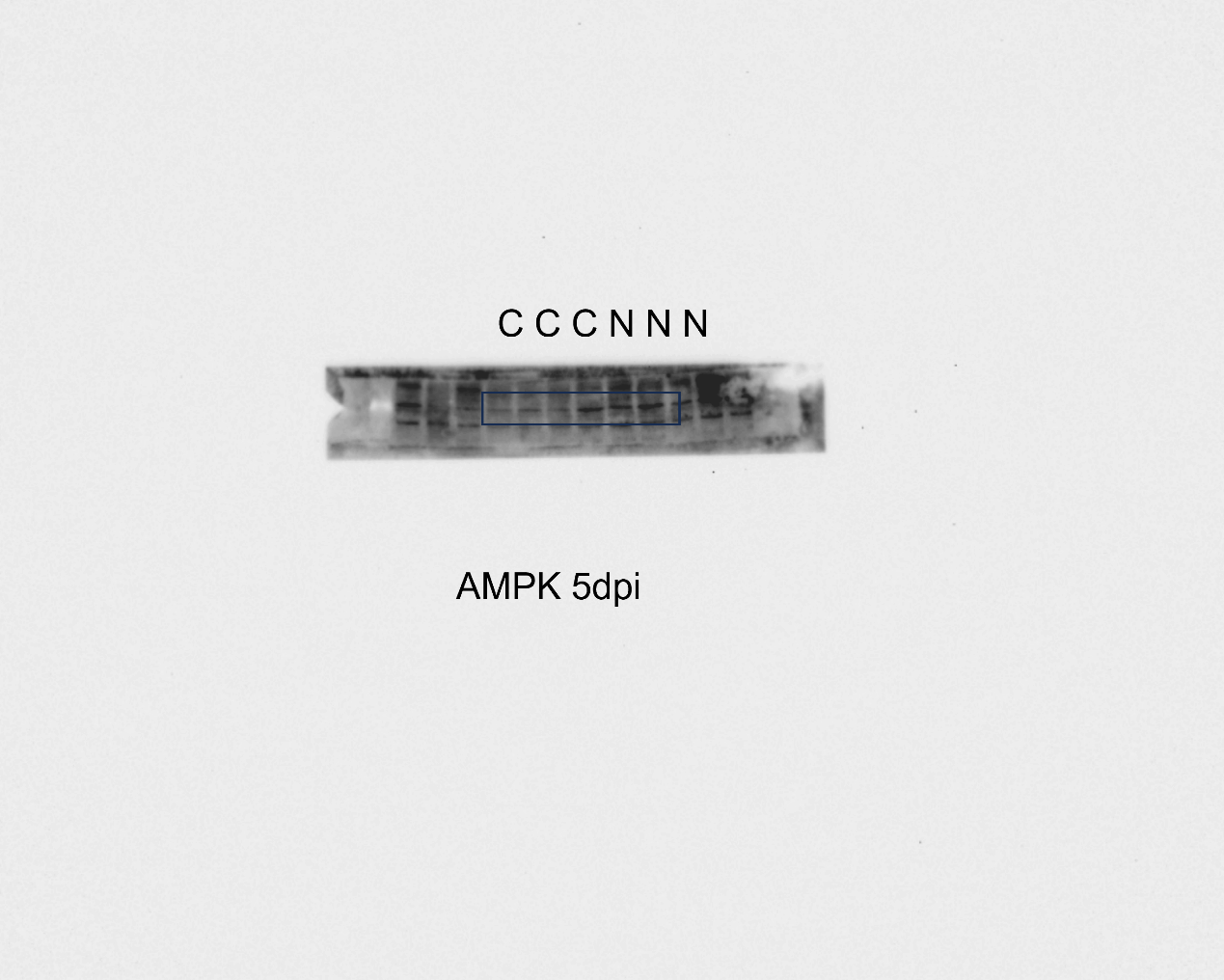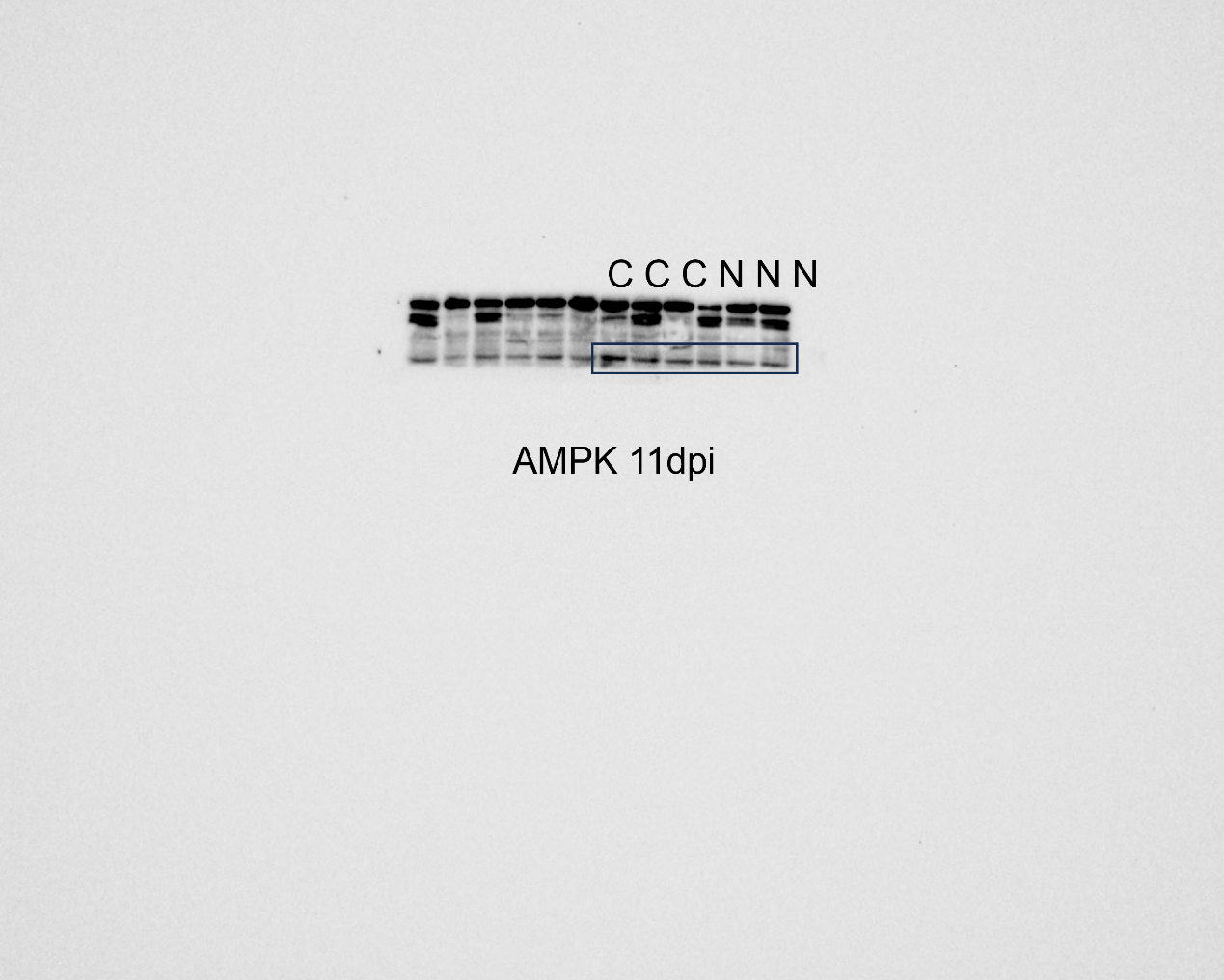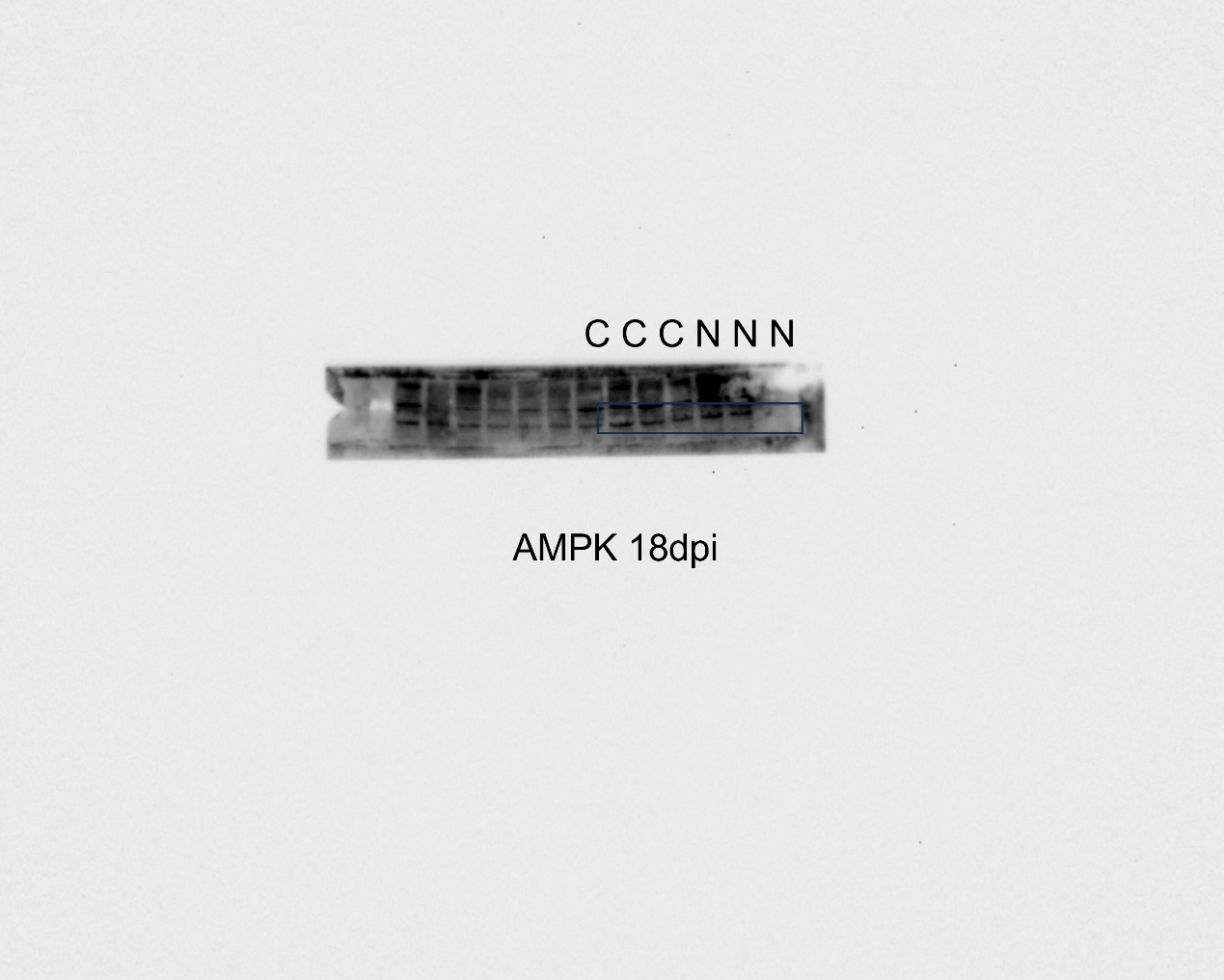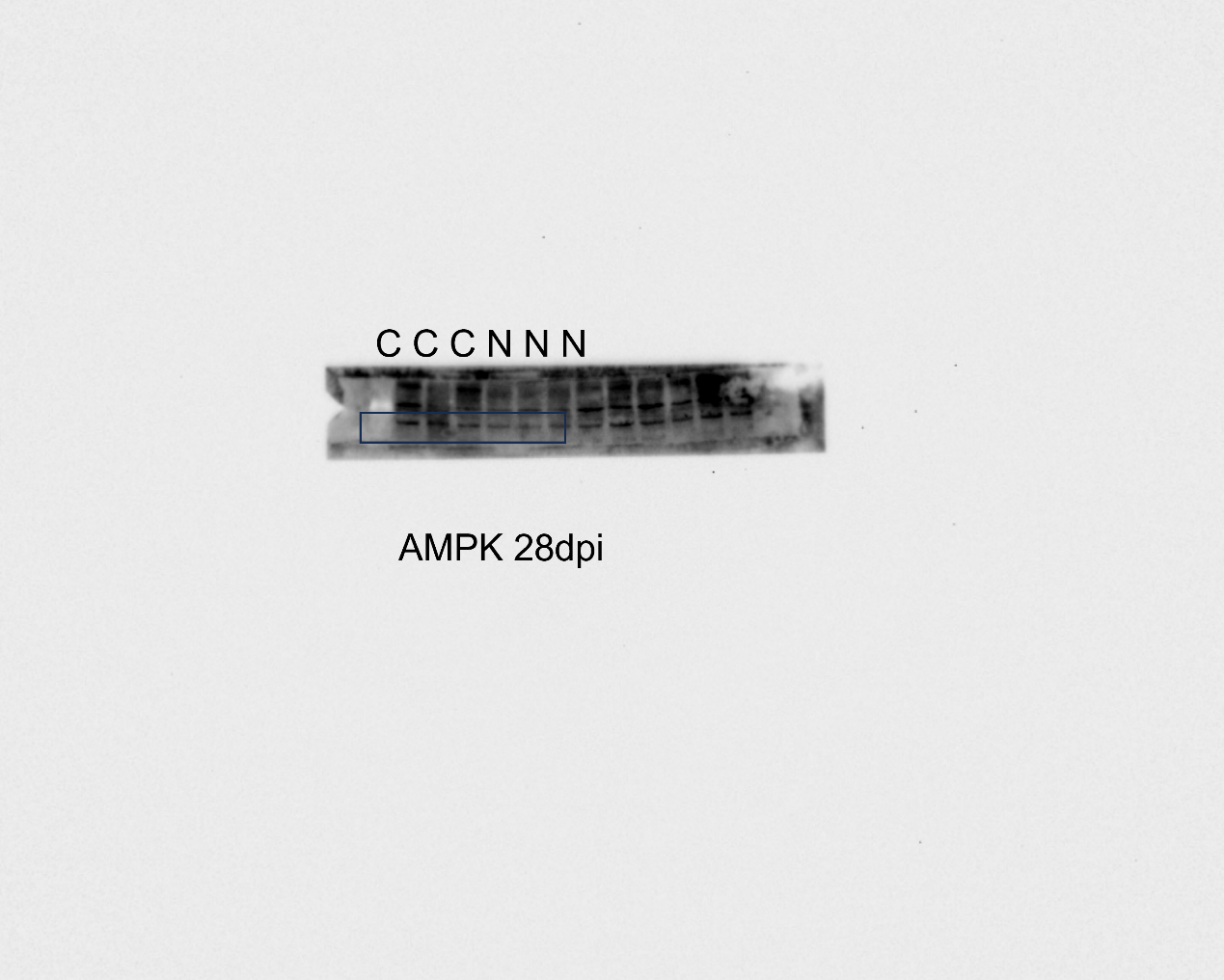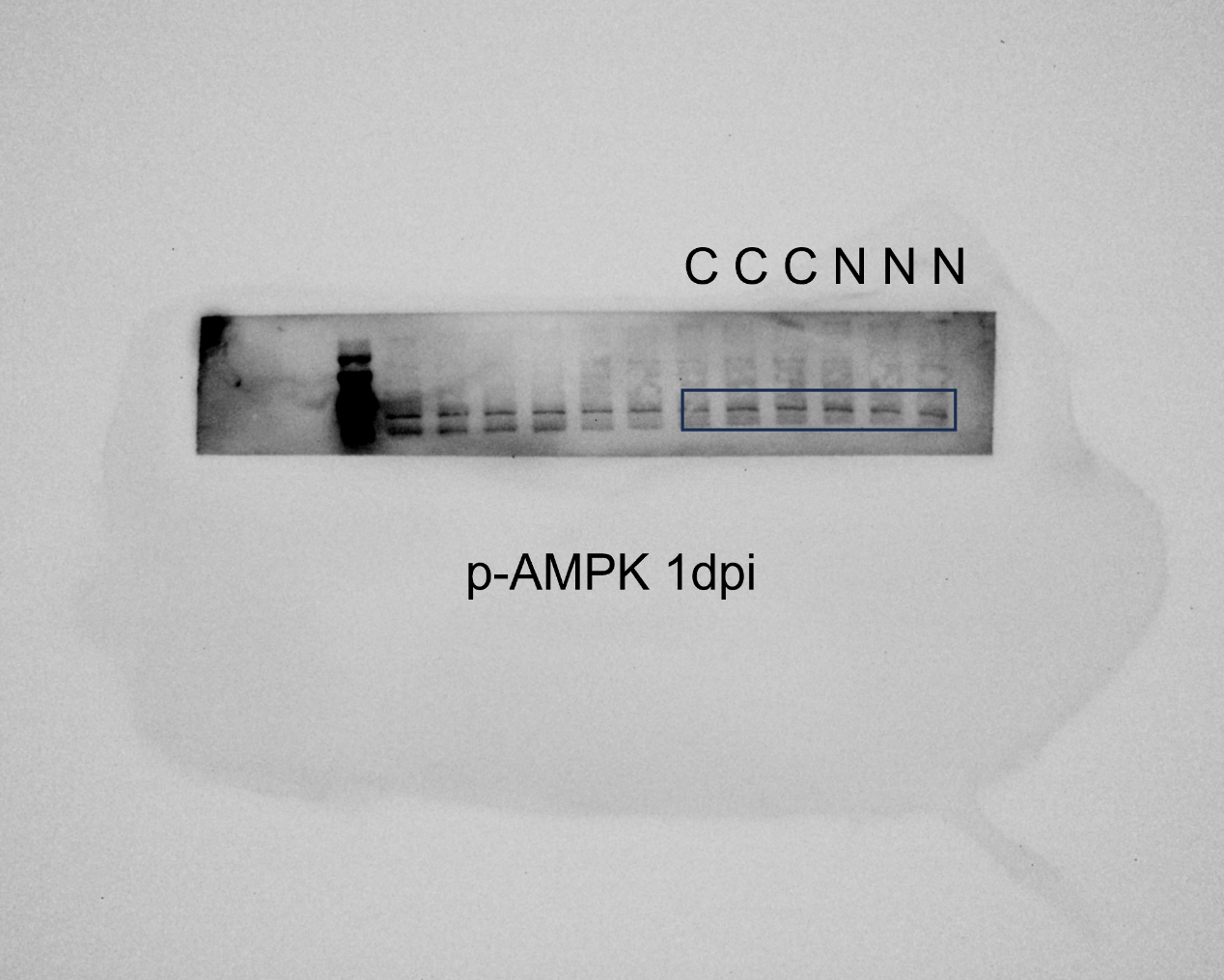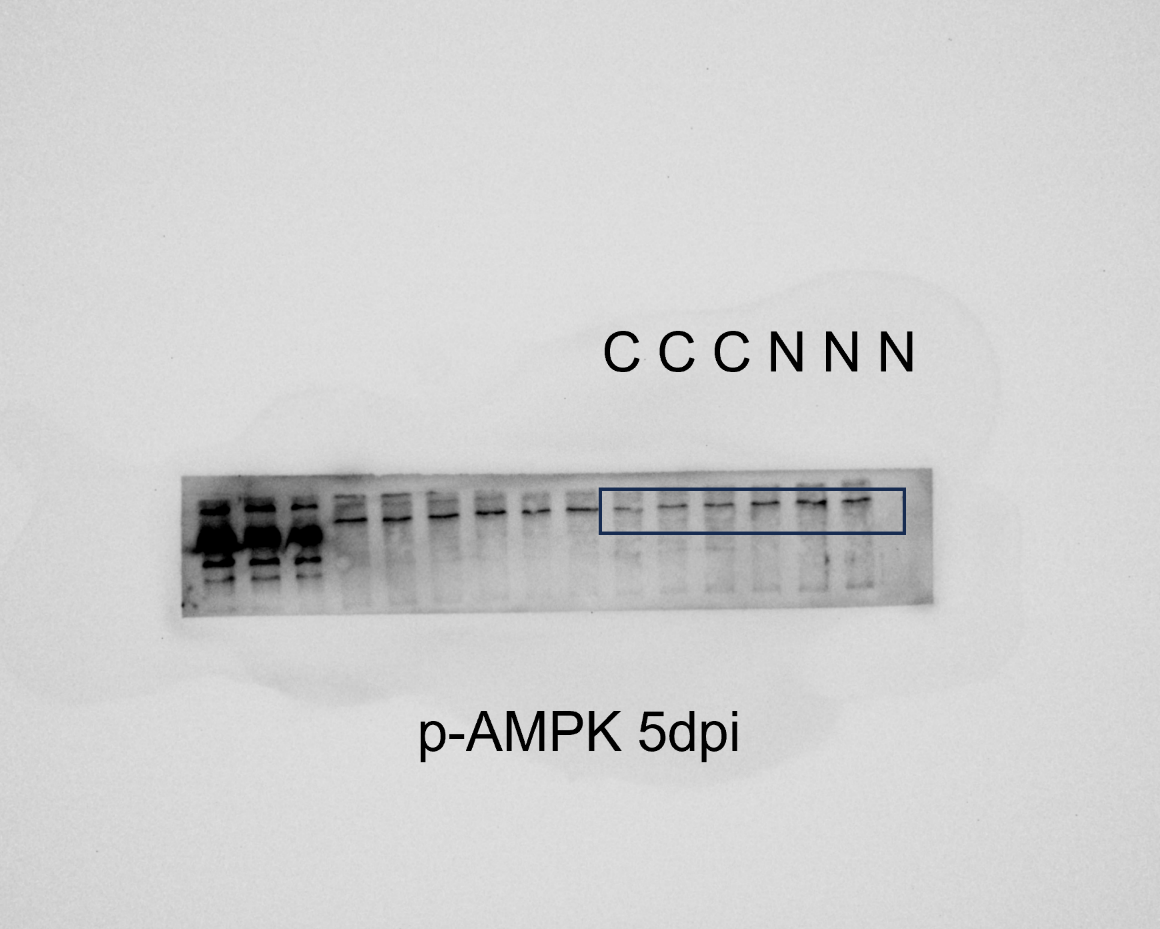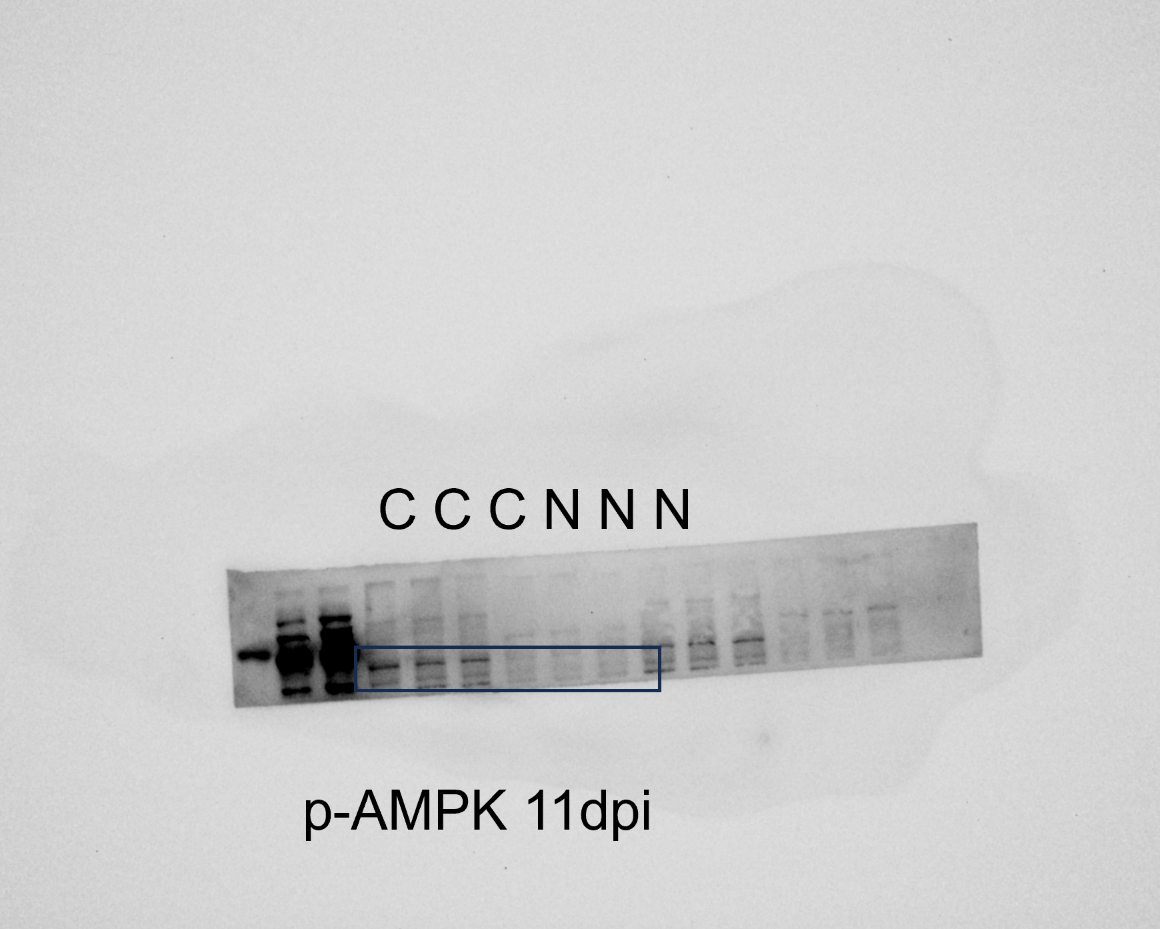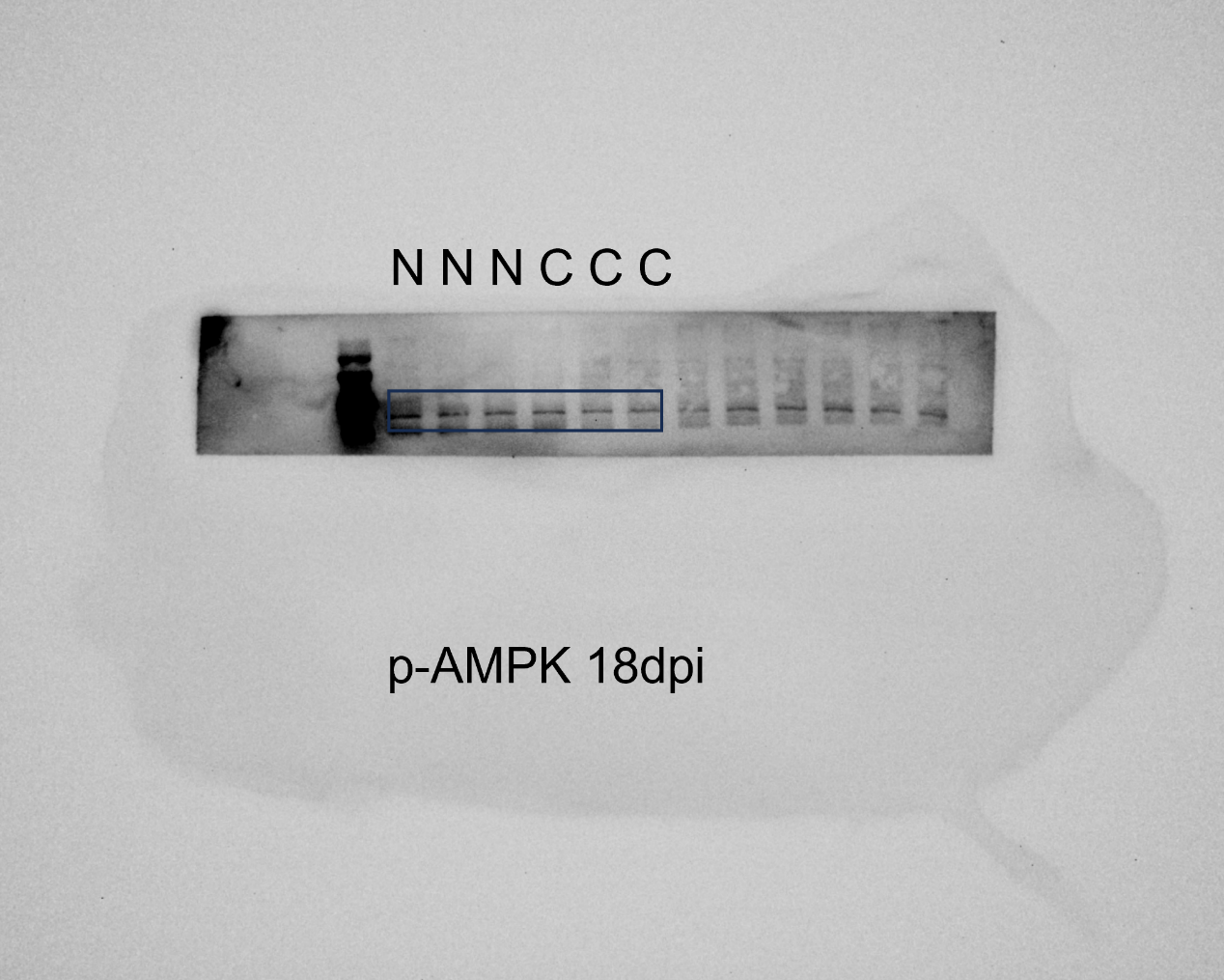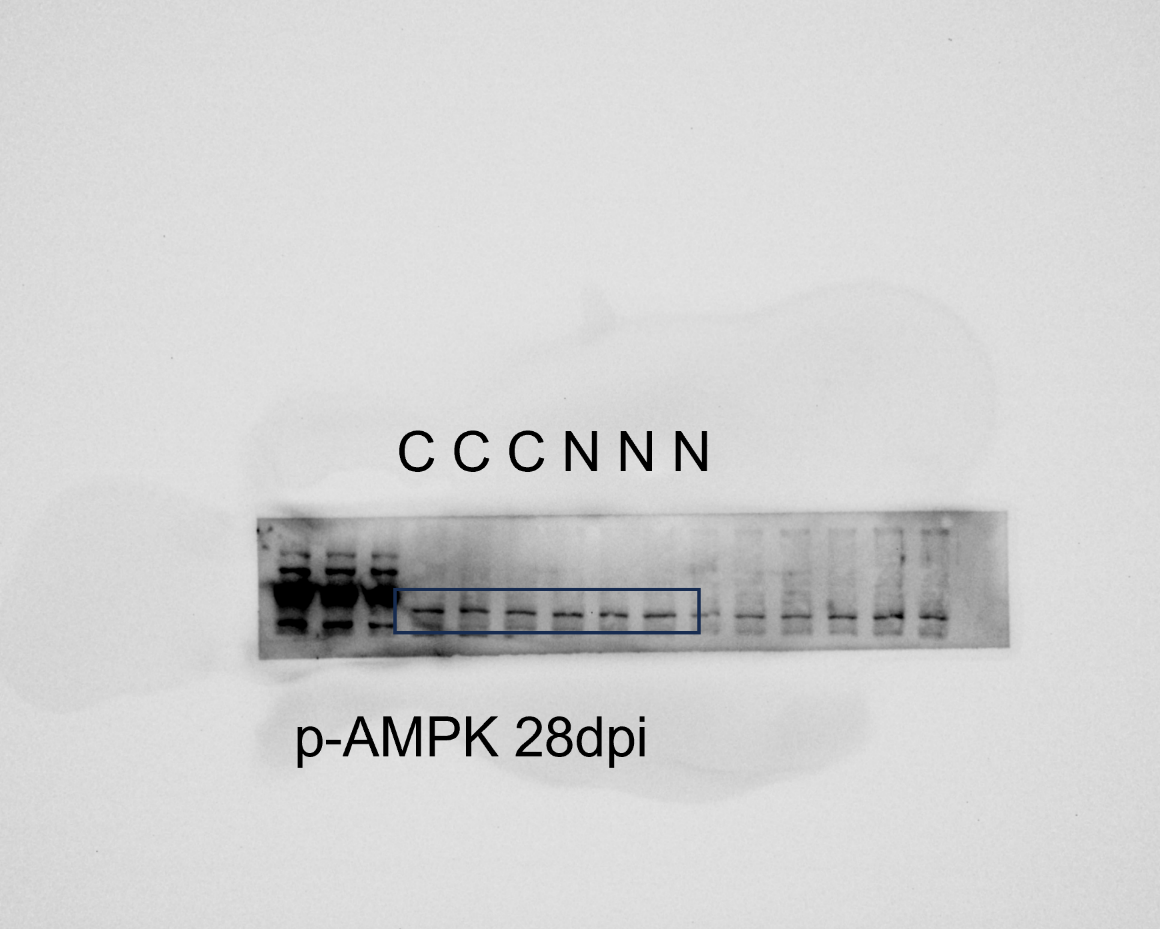** | | |
| **Figure.3 (A)** | | |
| **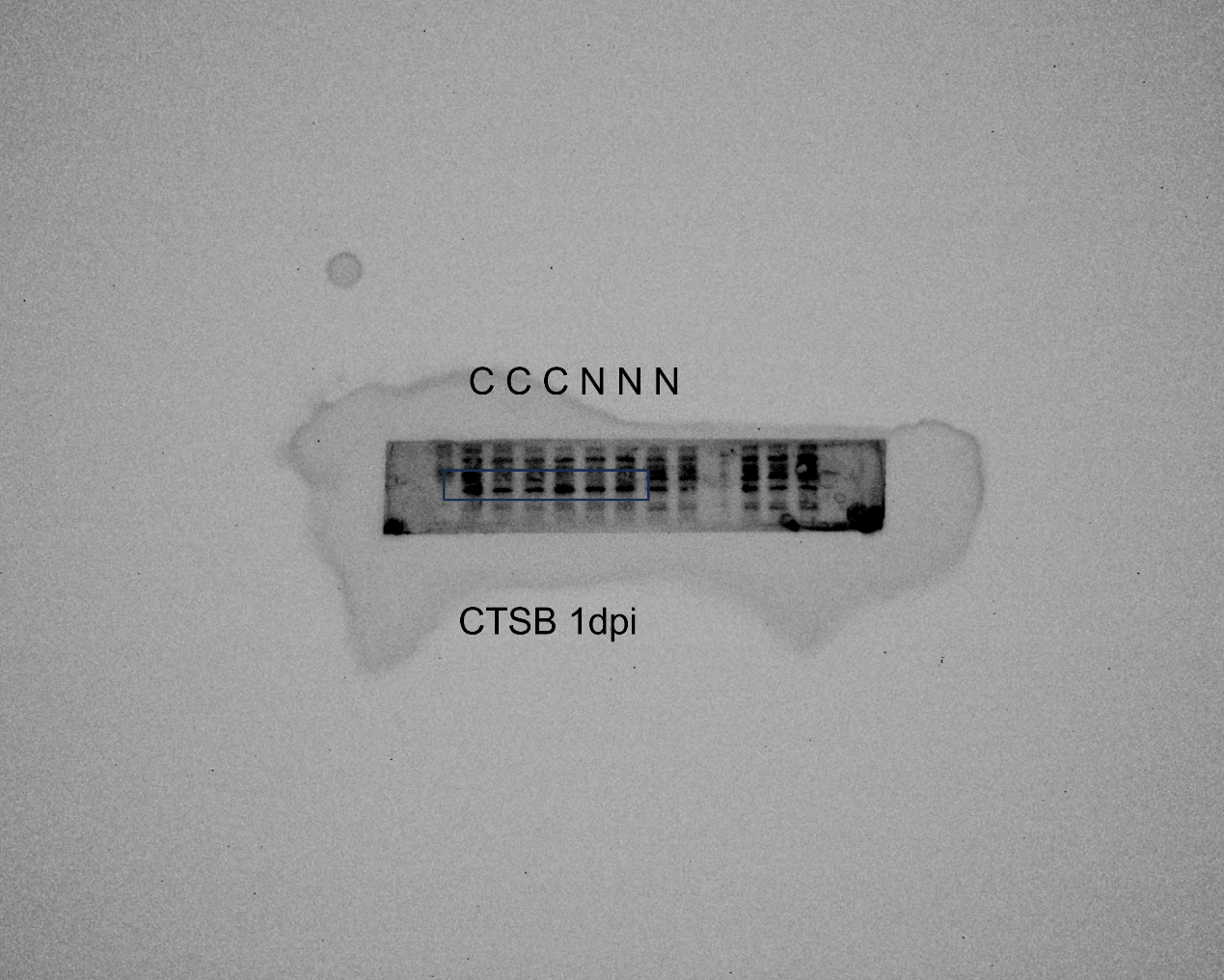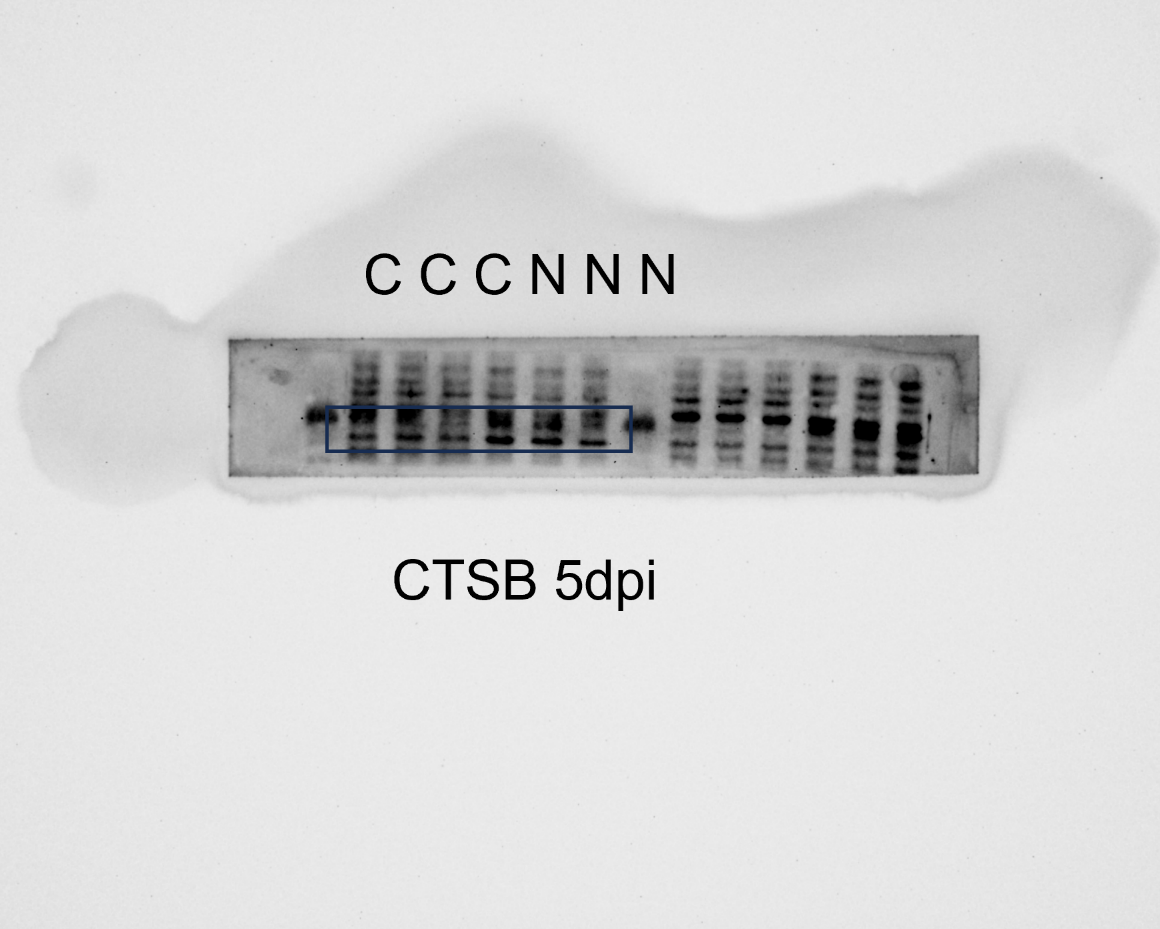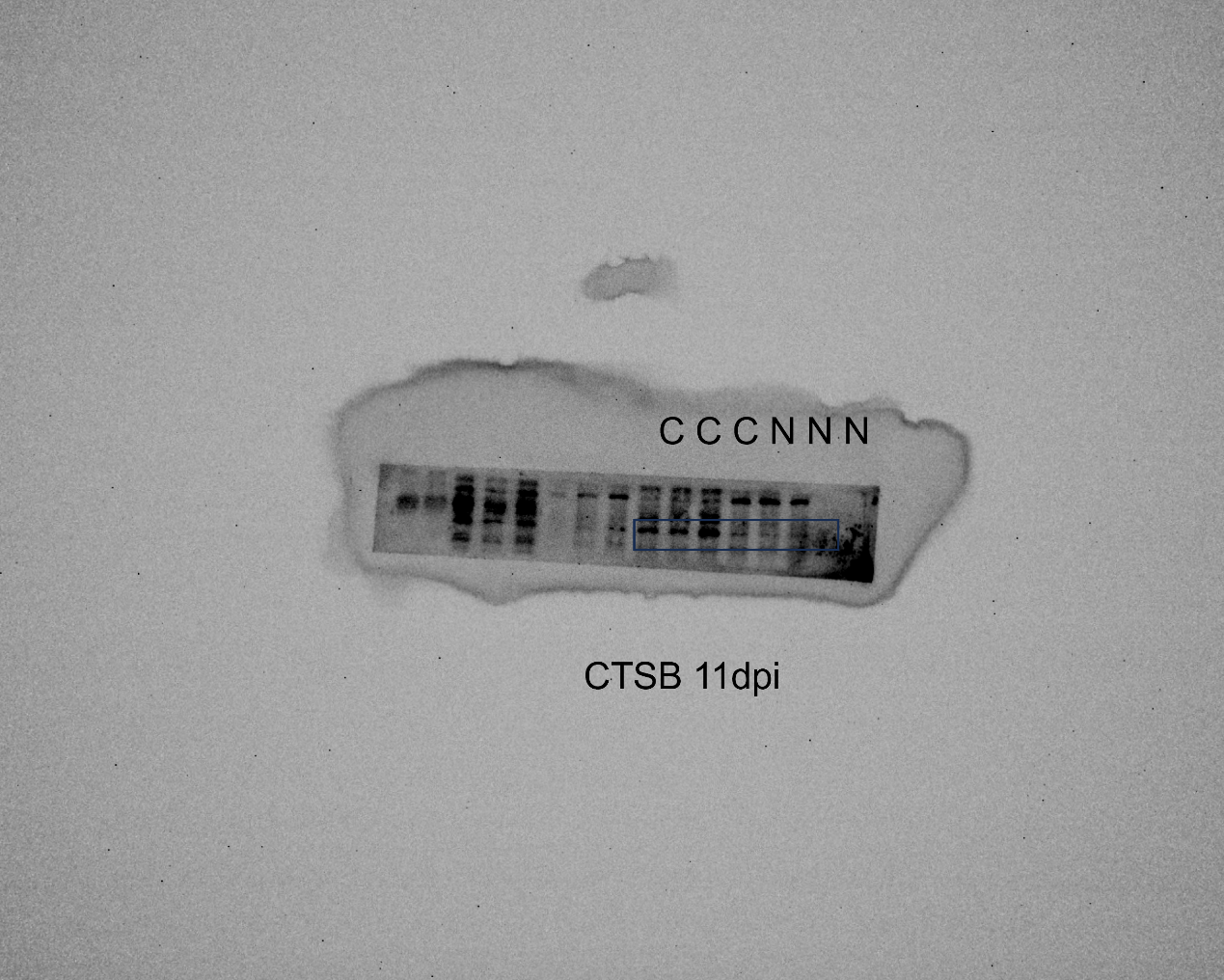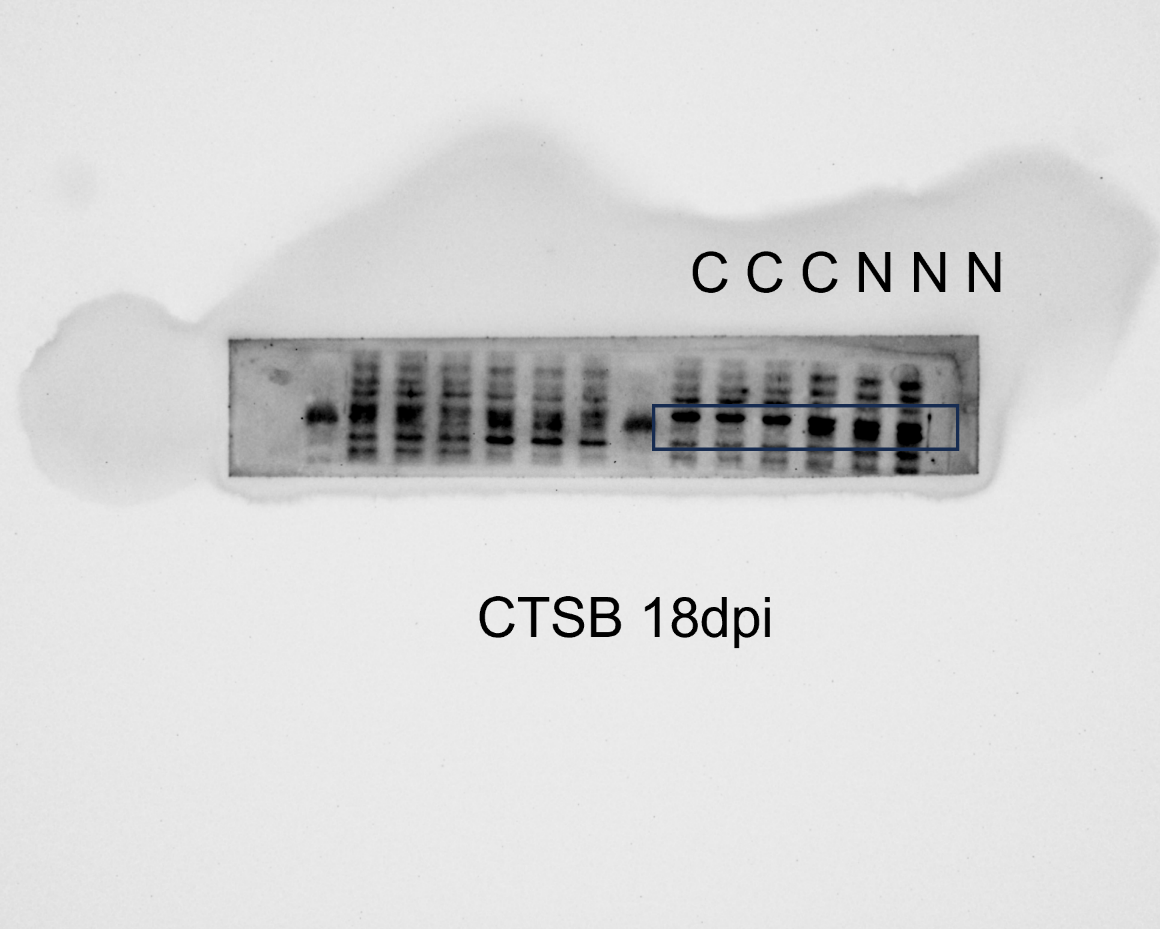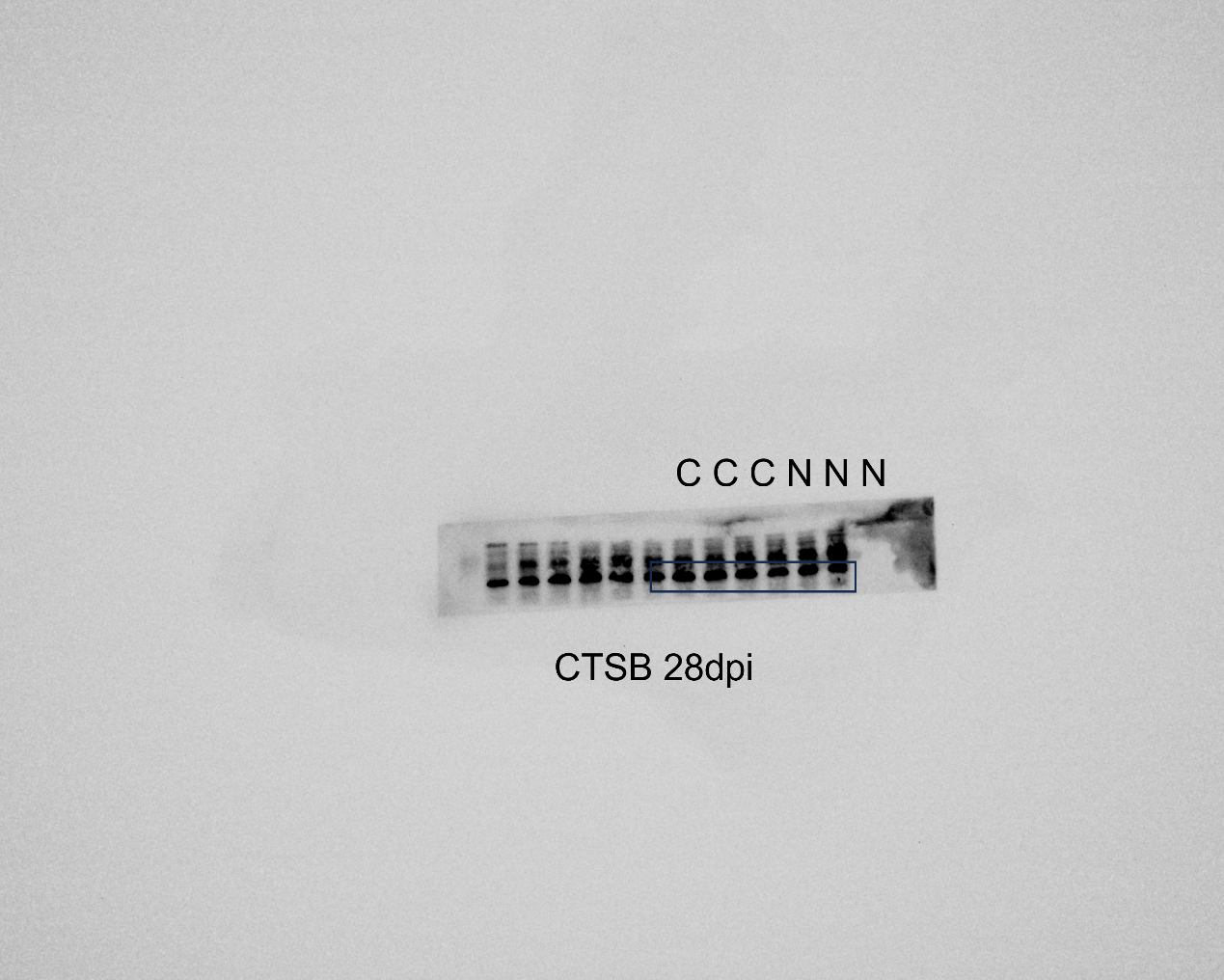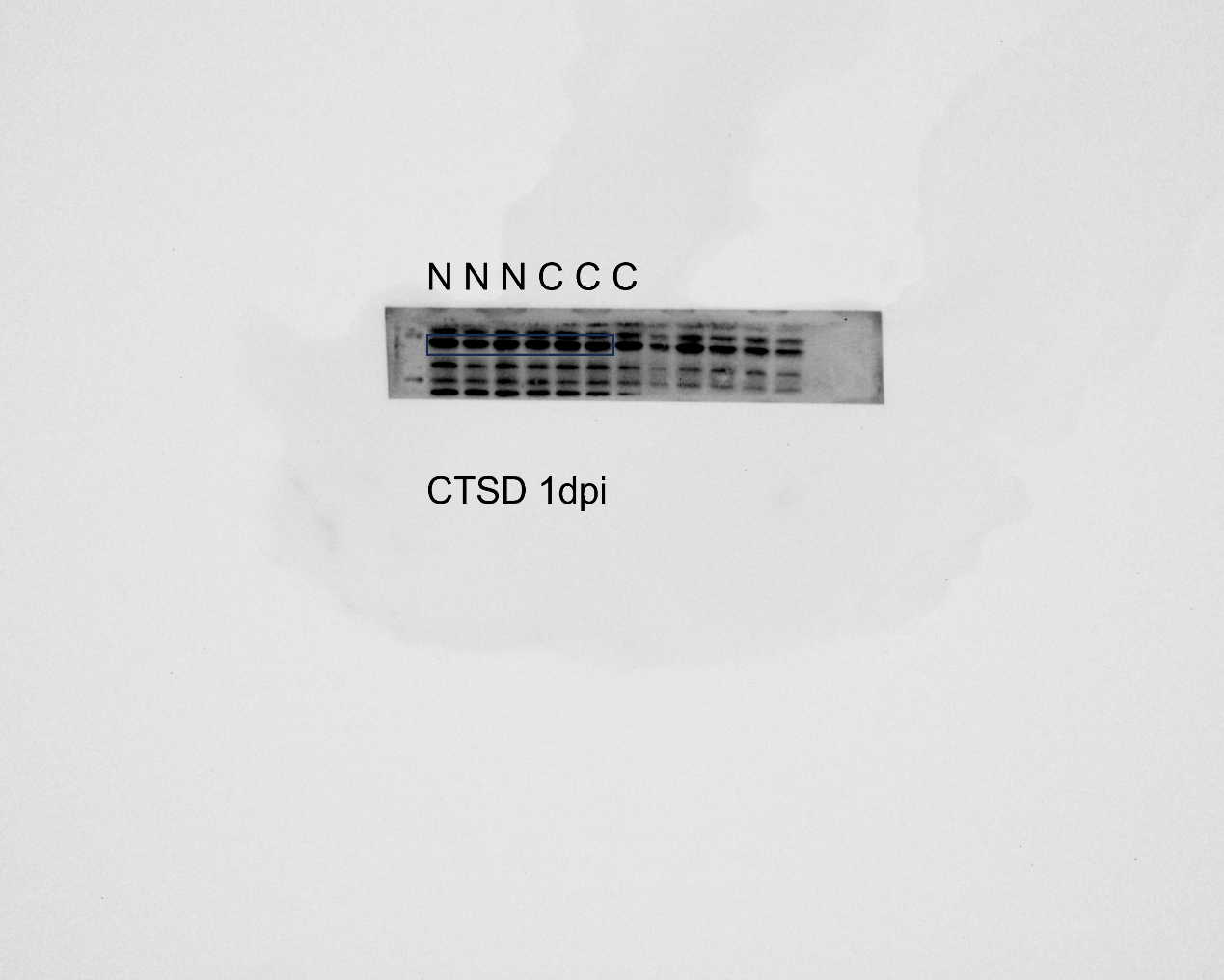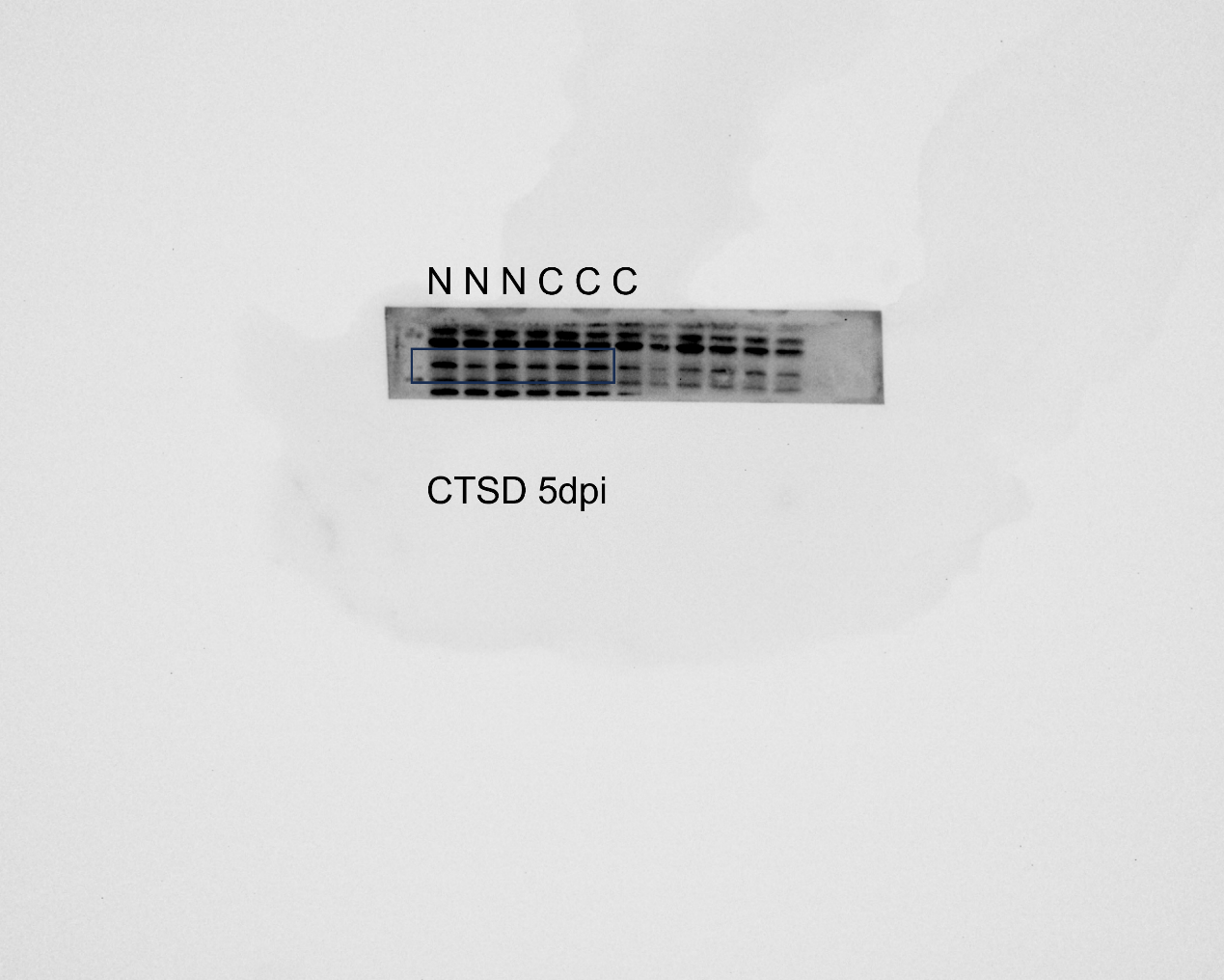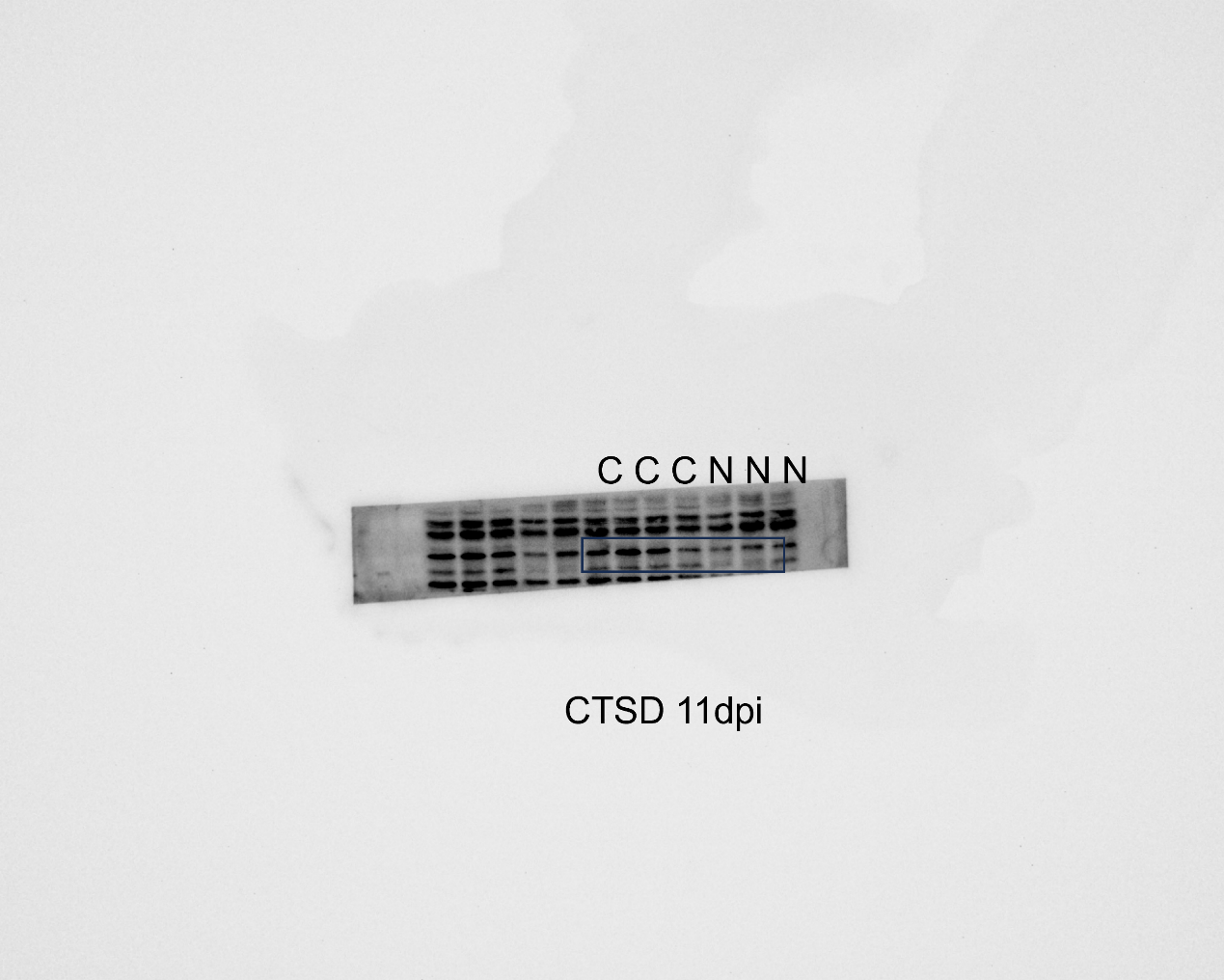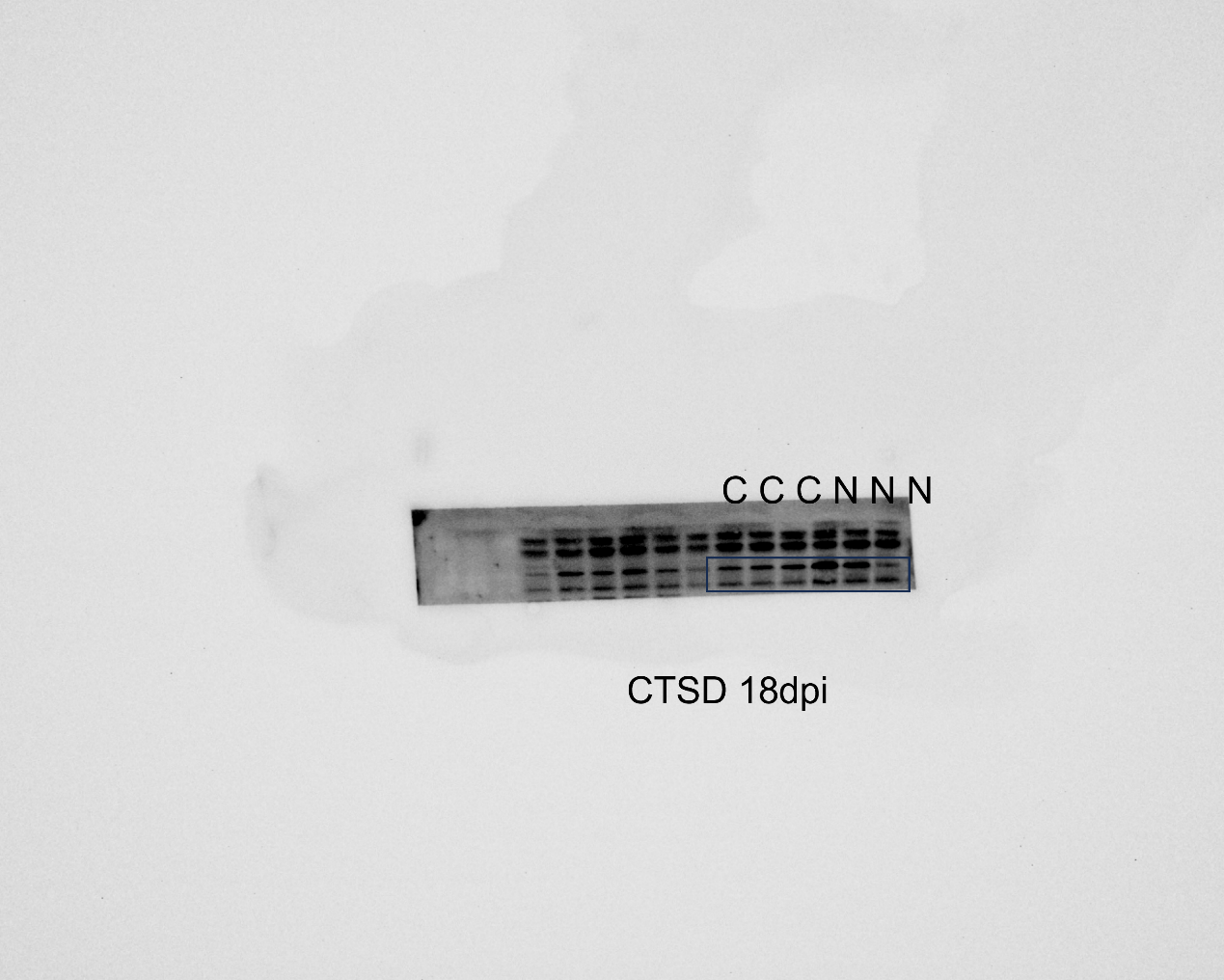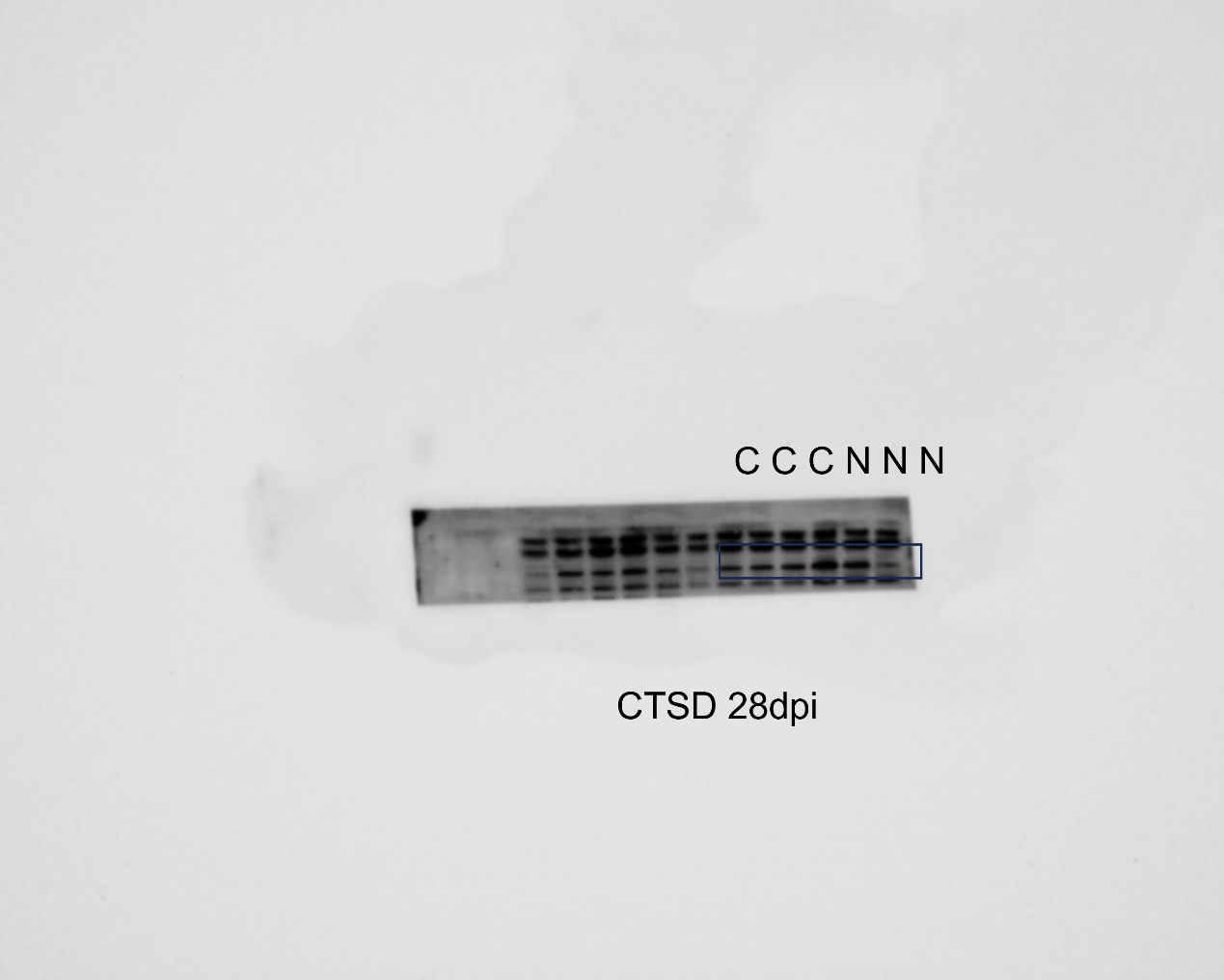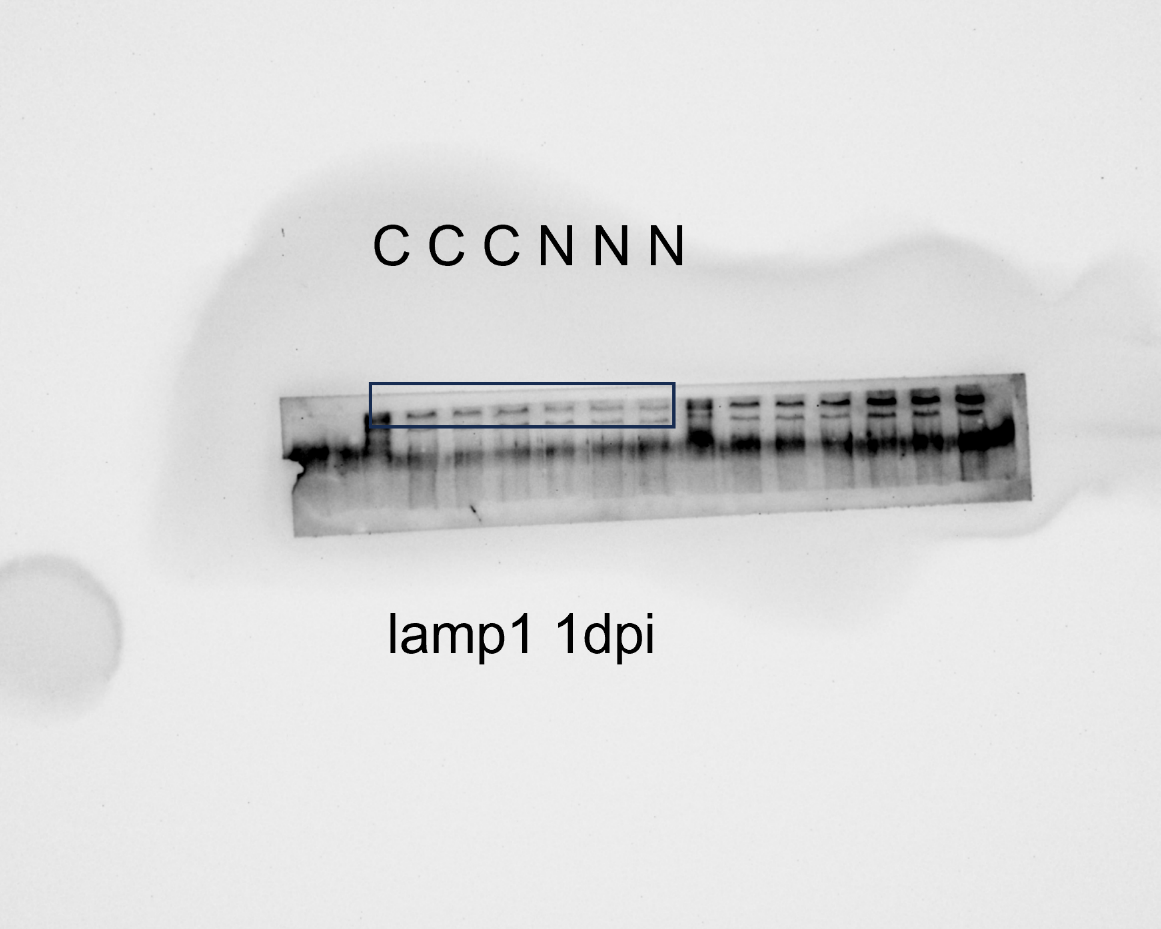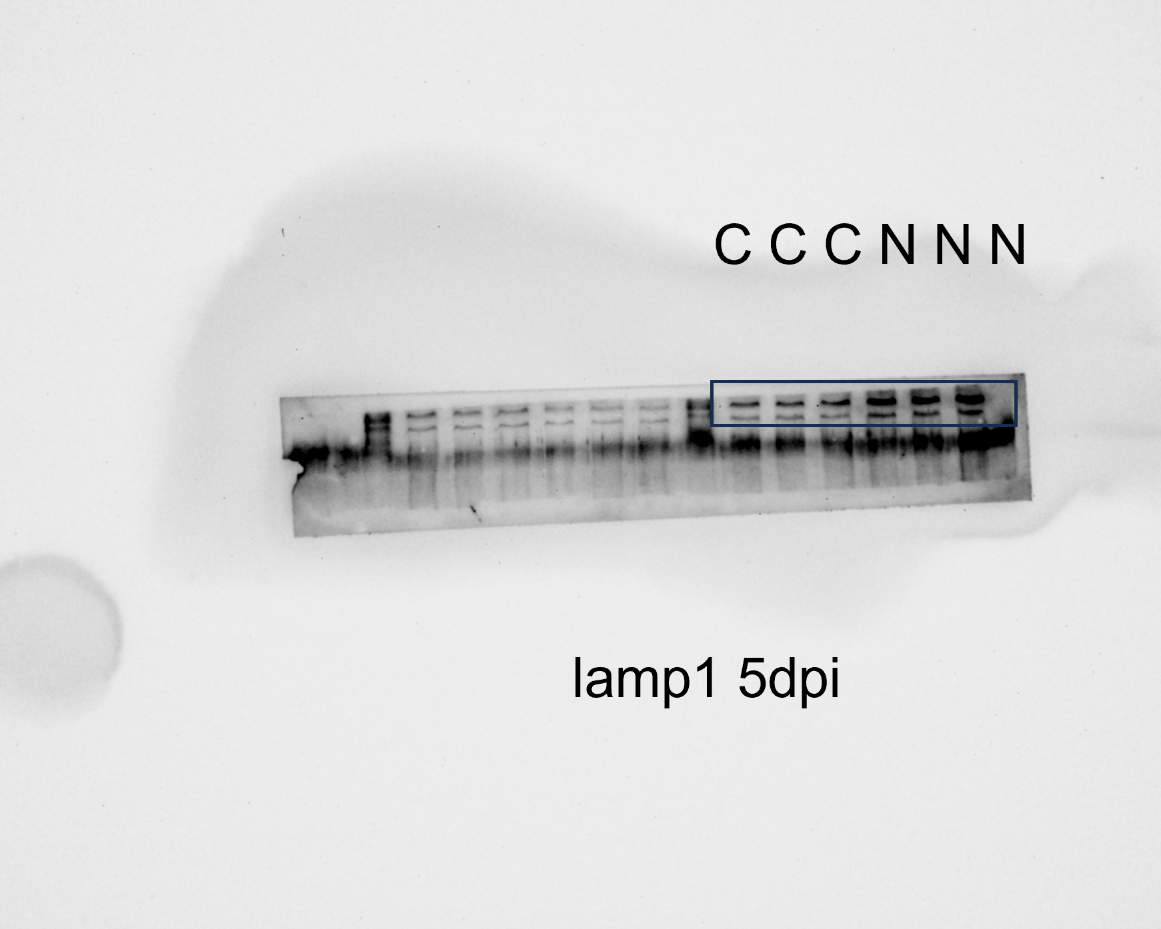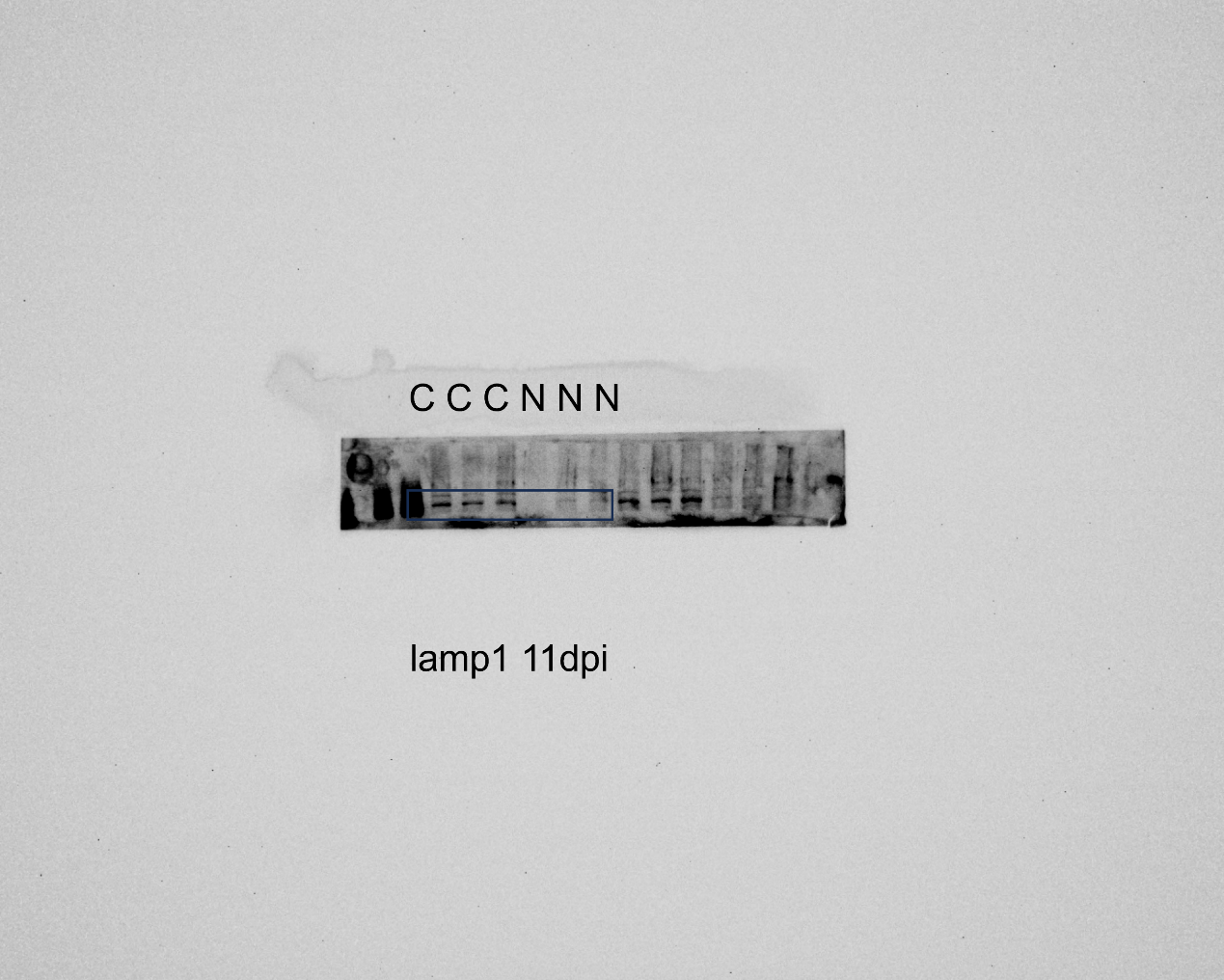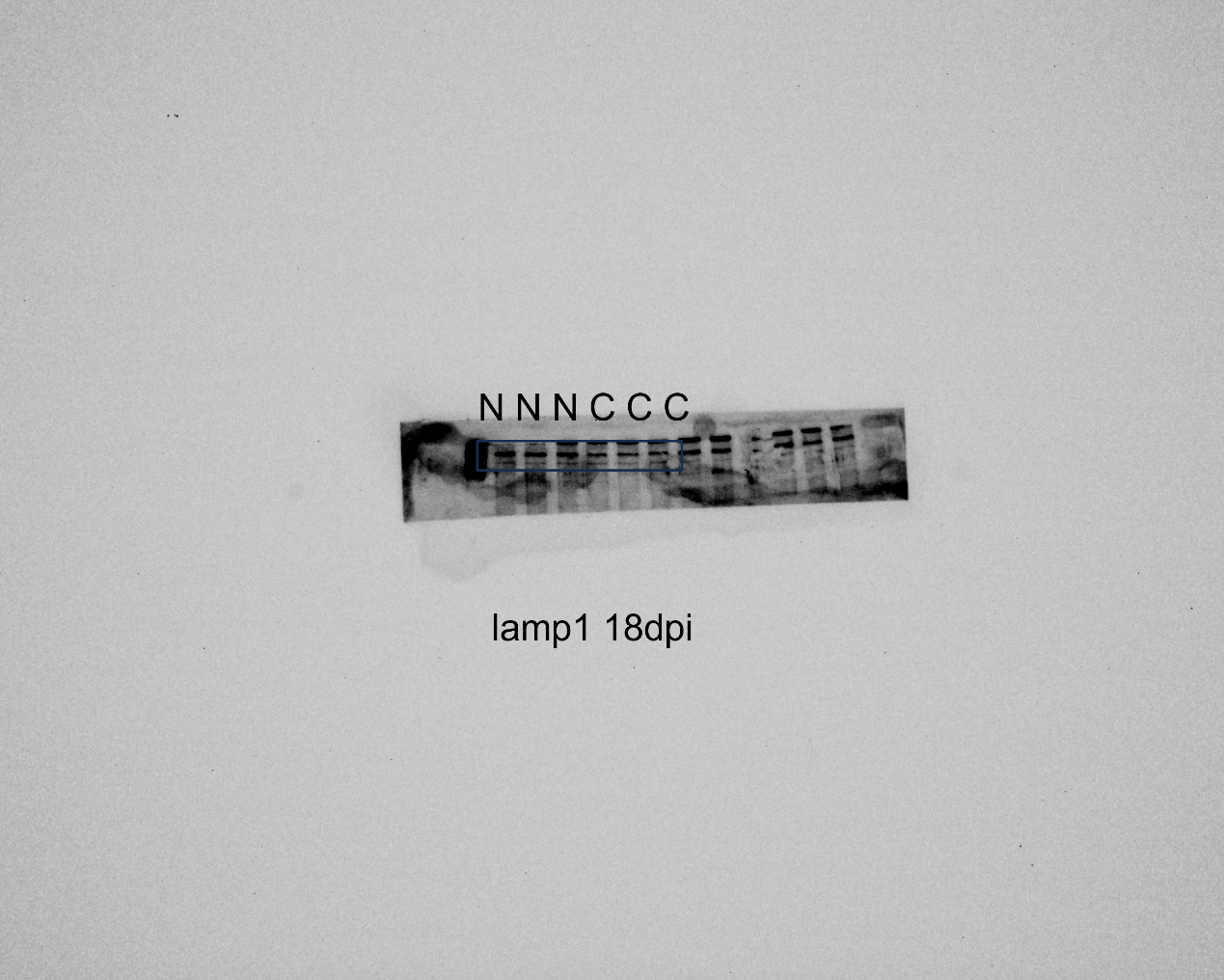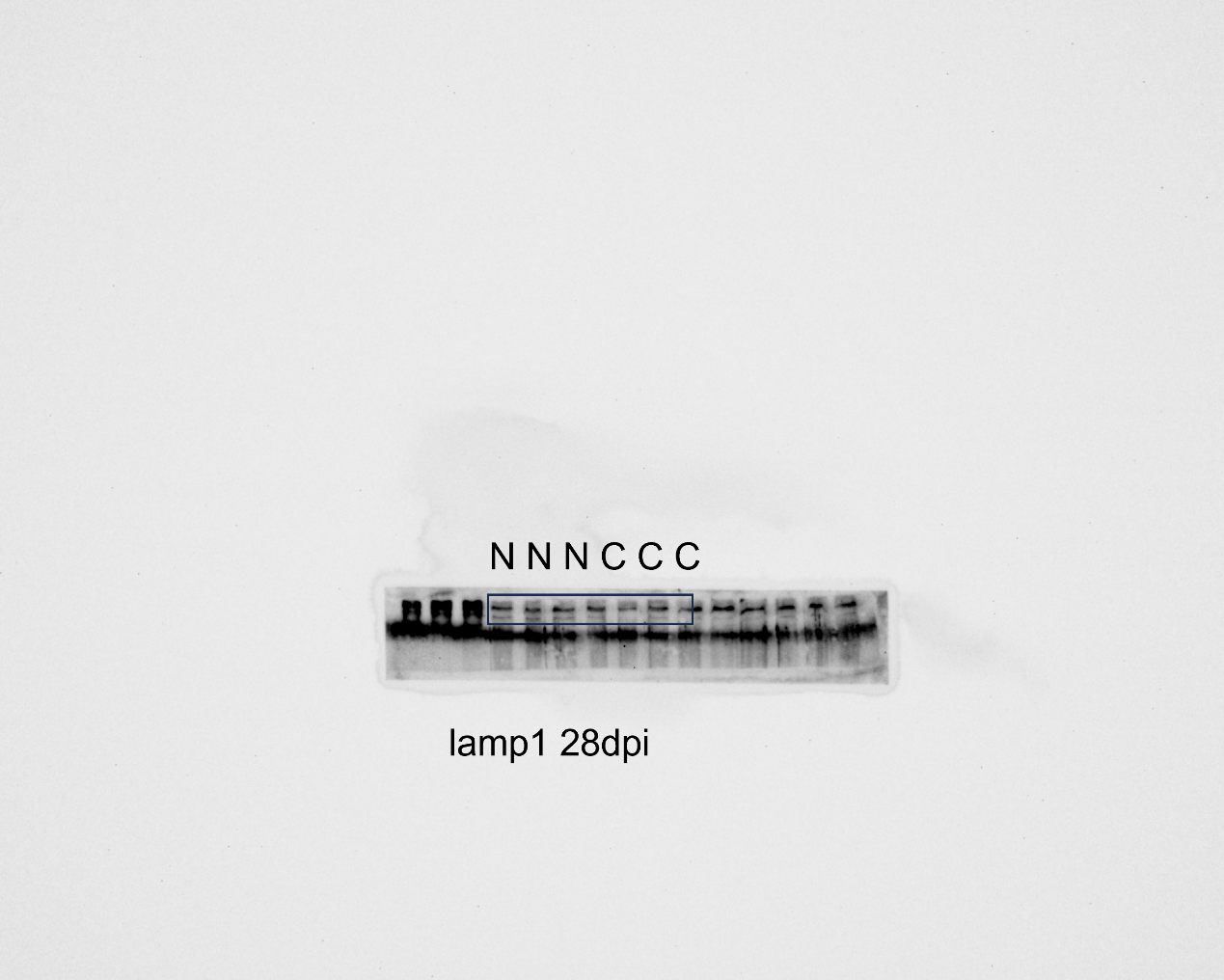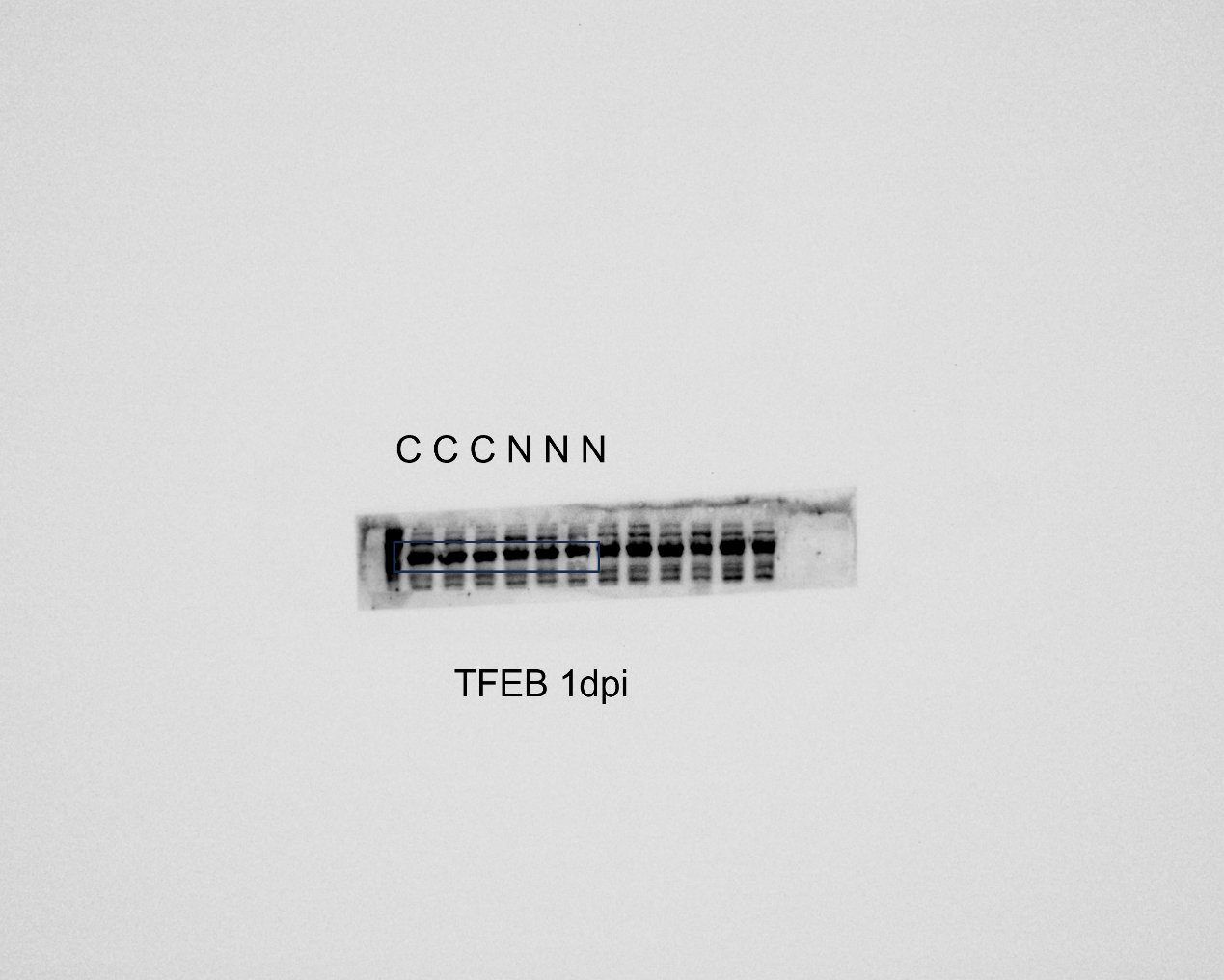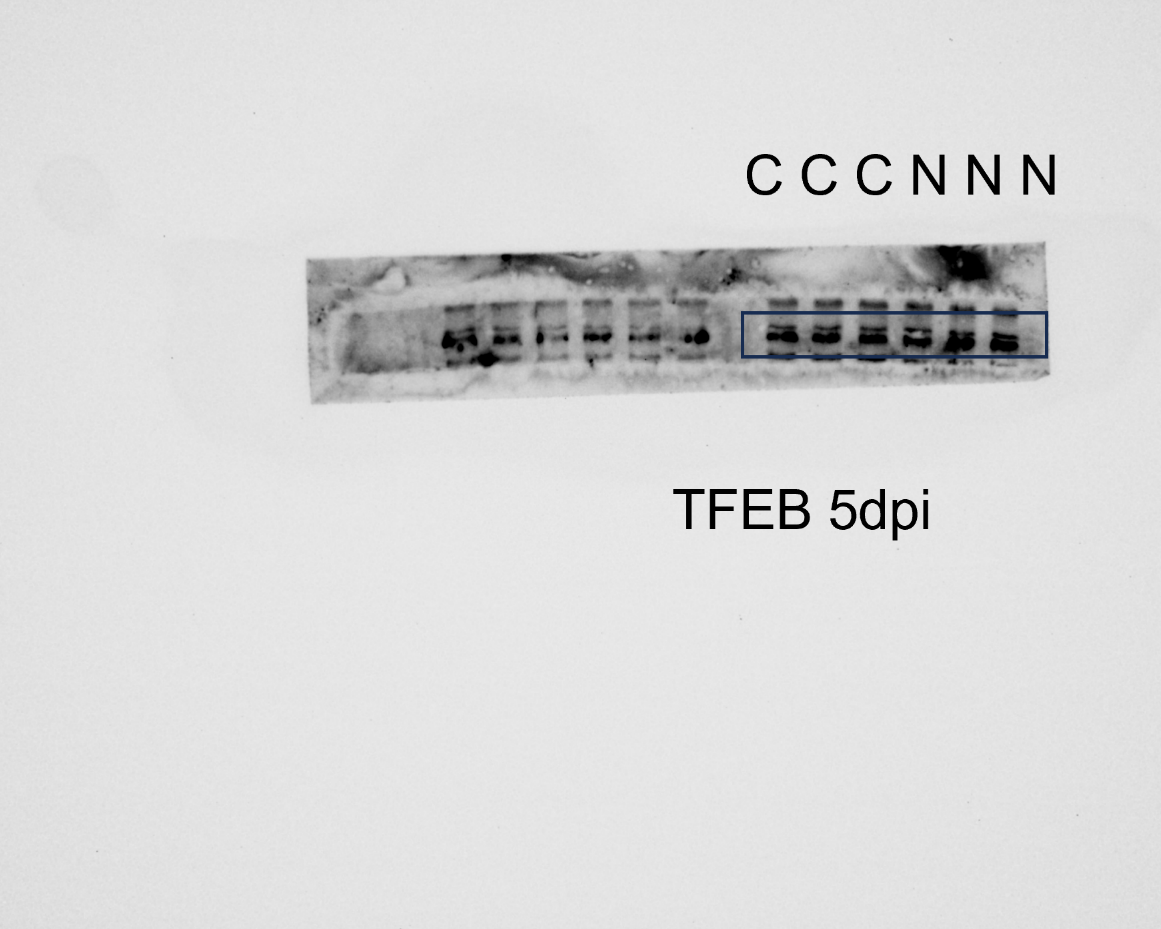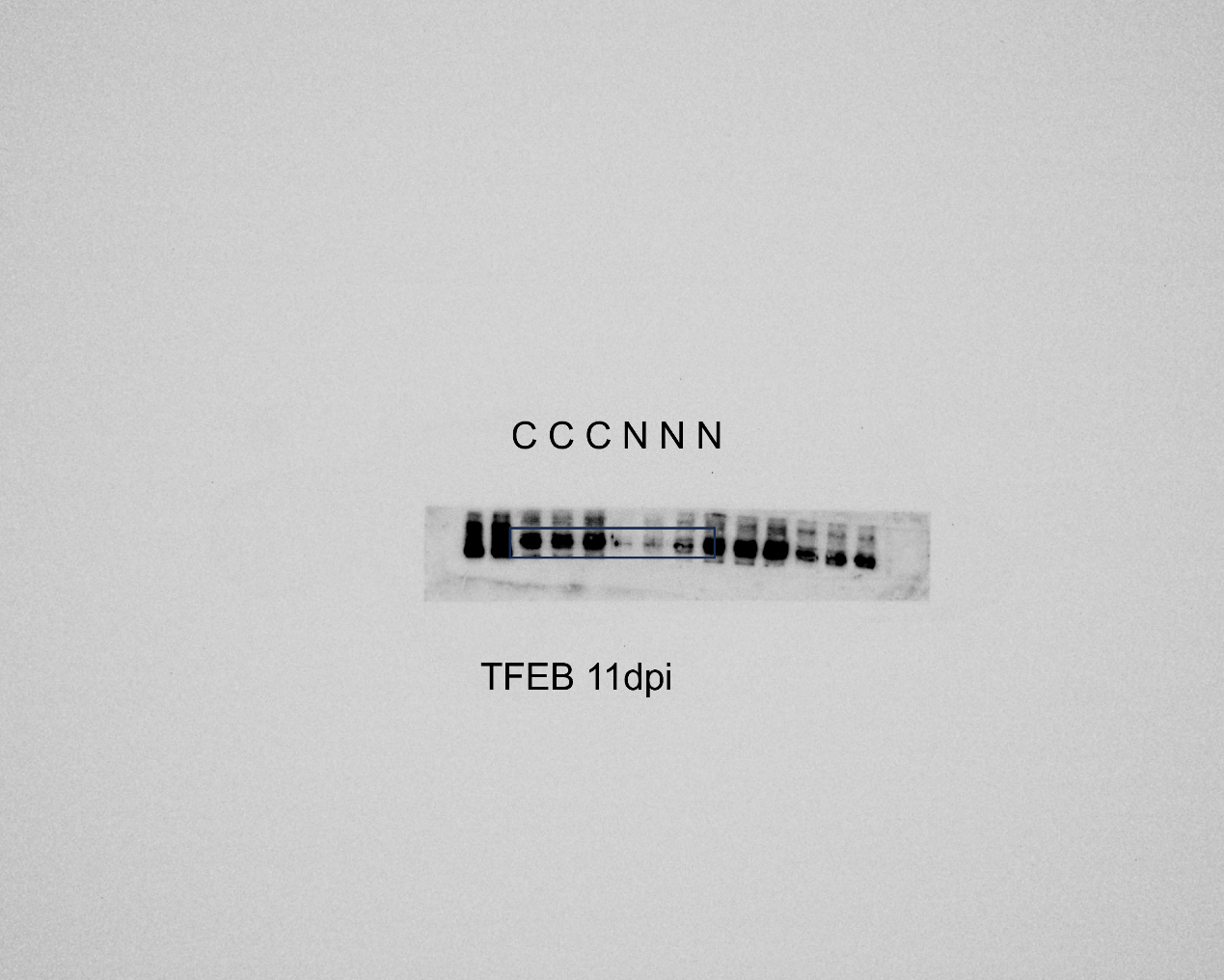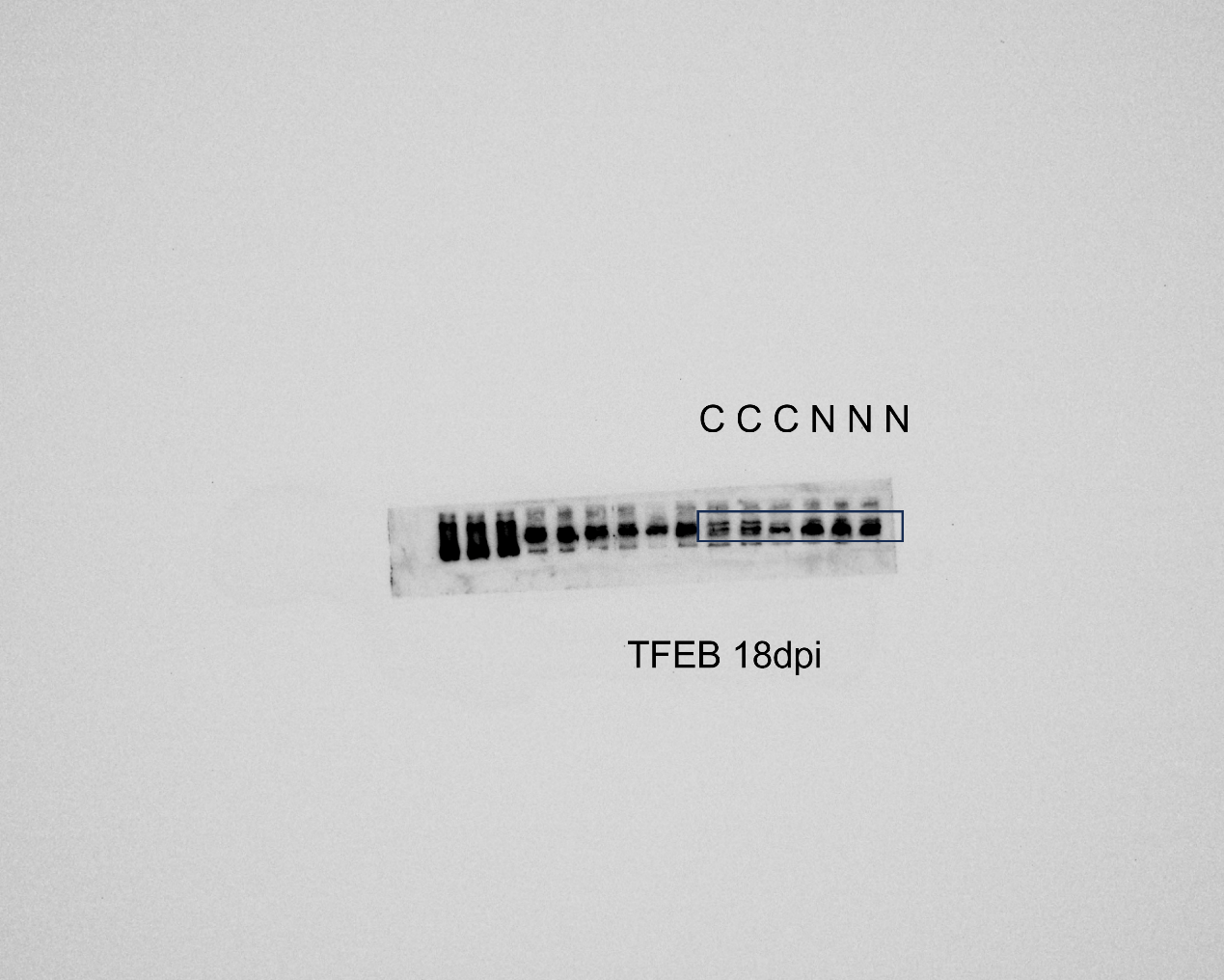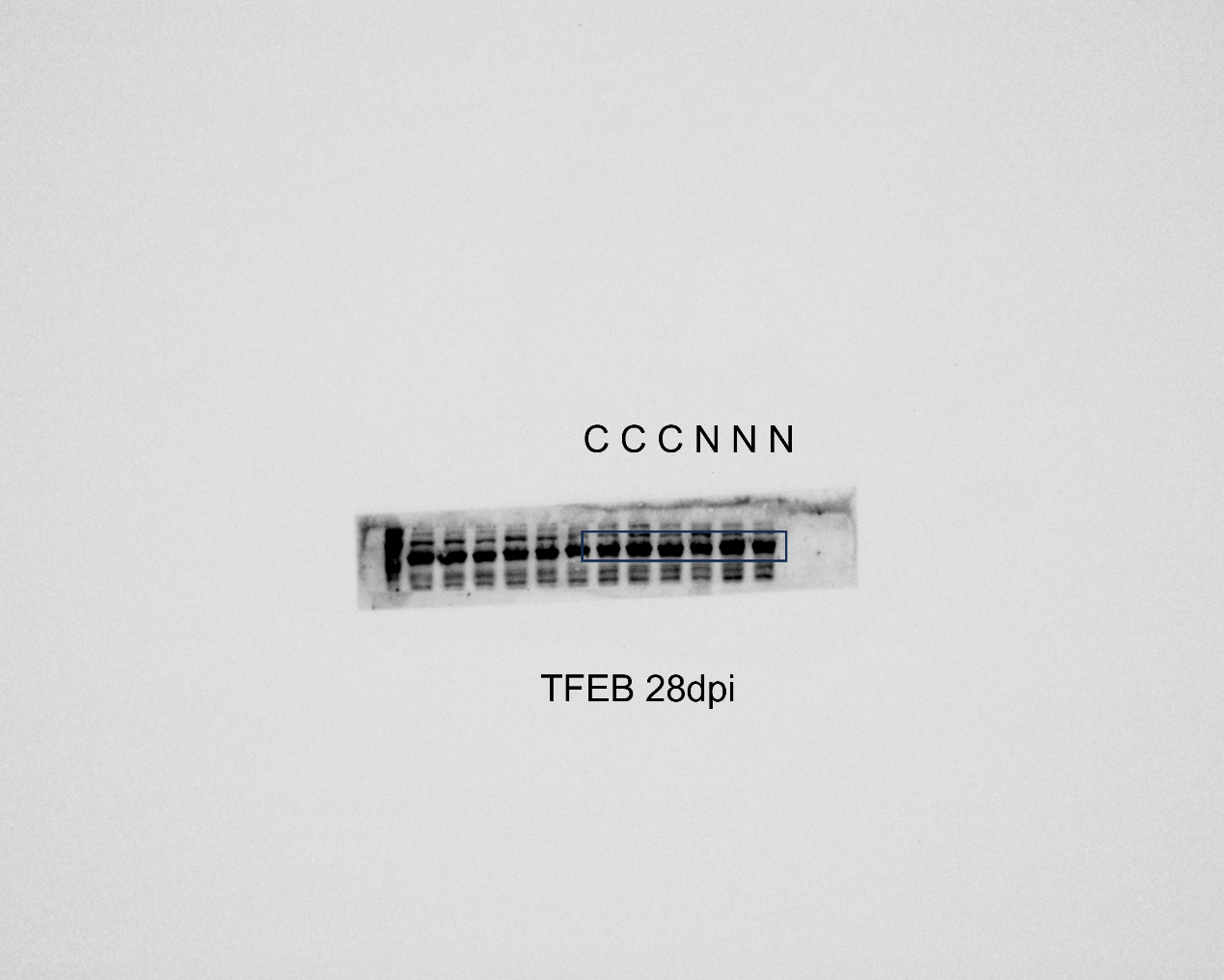** | | |
| **Figure.4 (B)** | | |
| **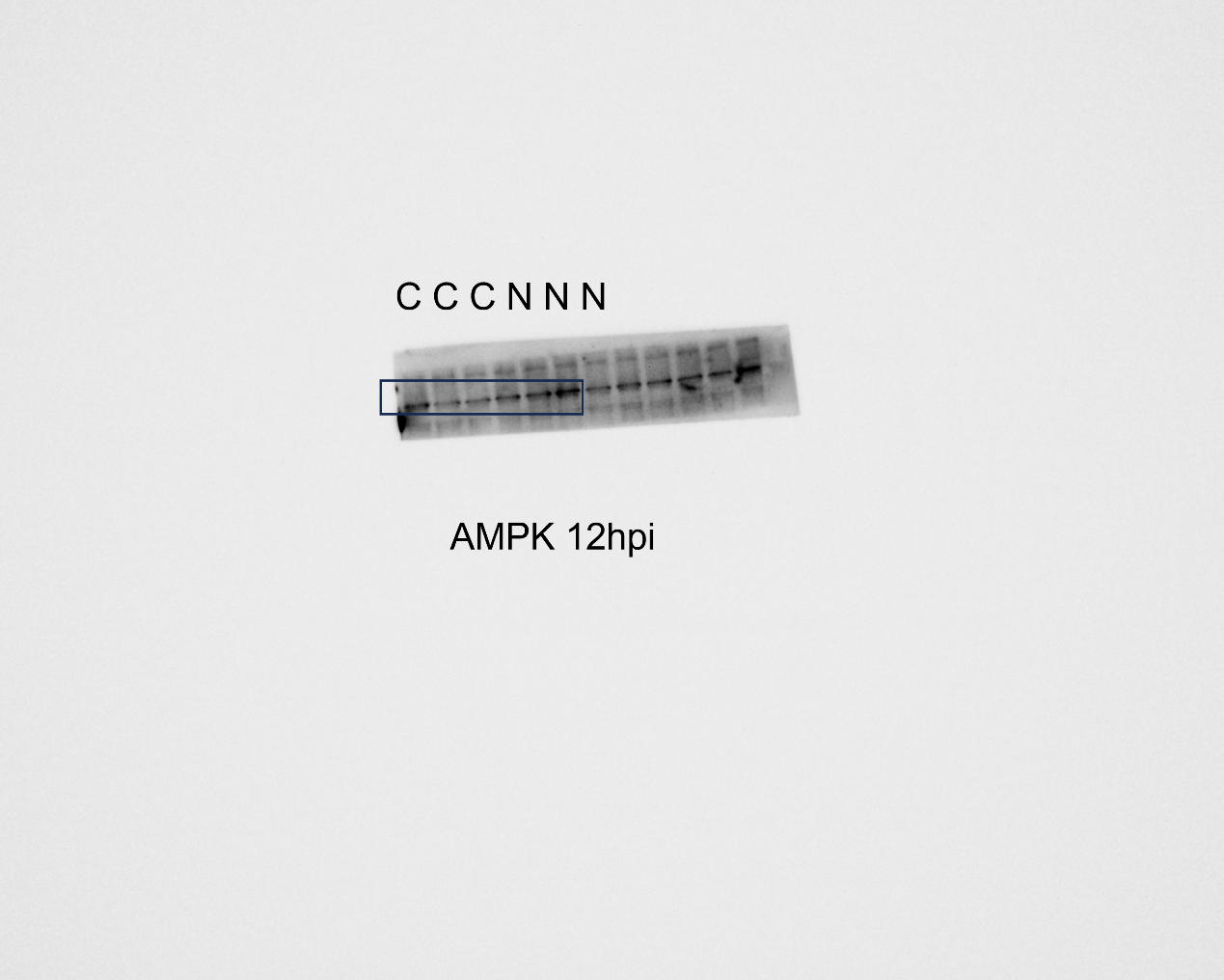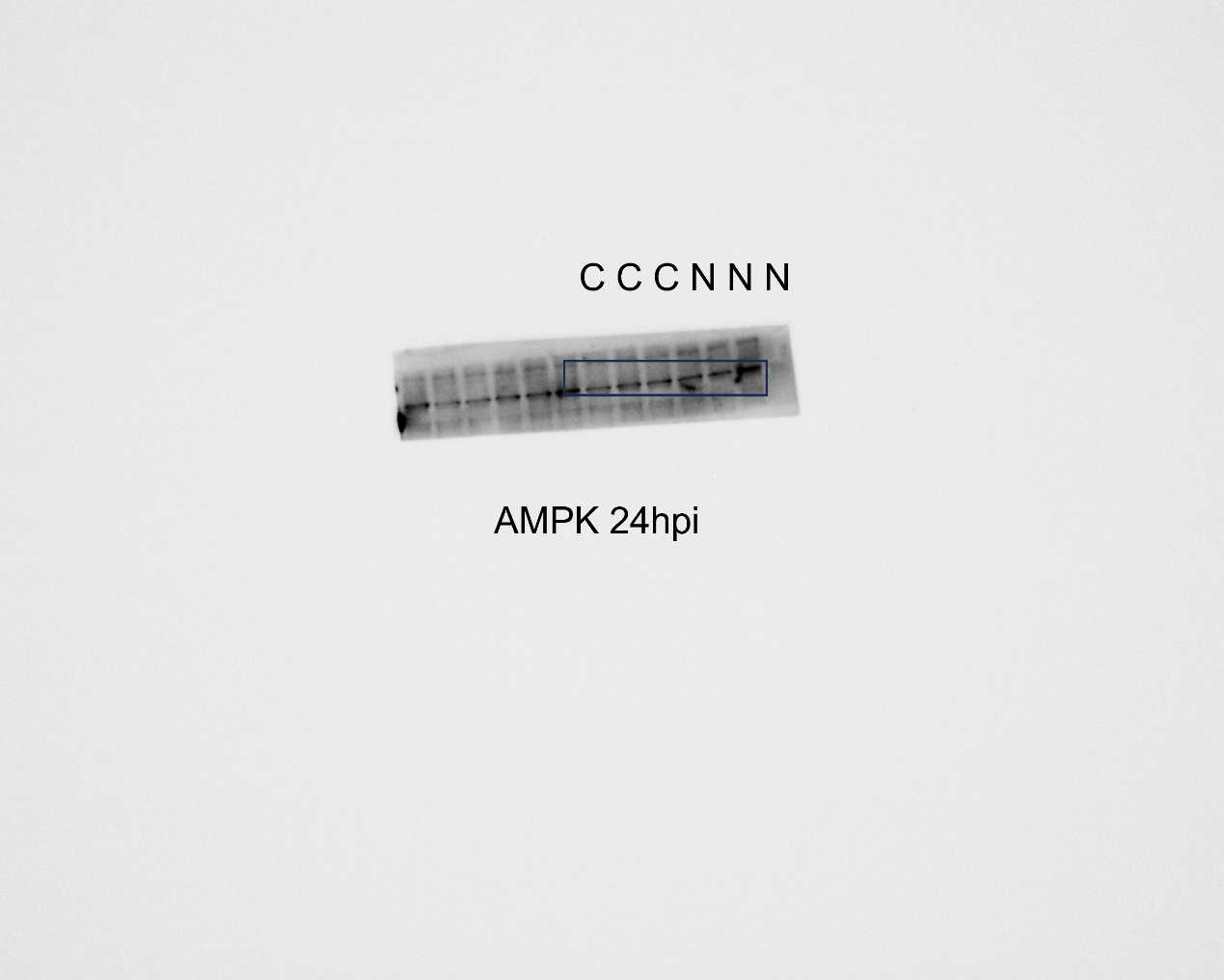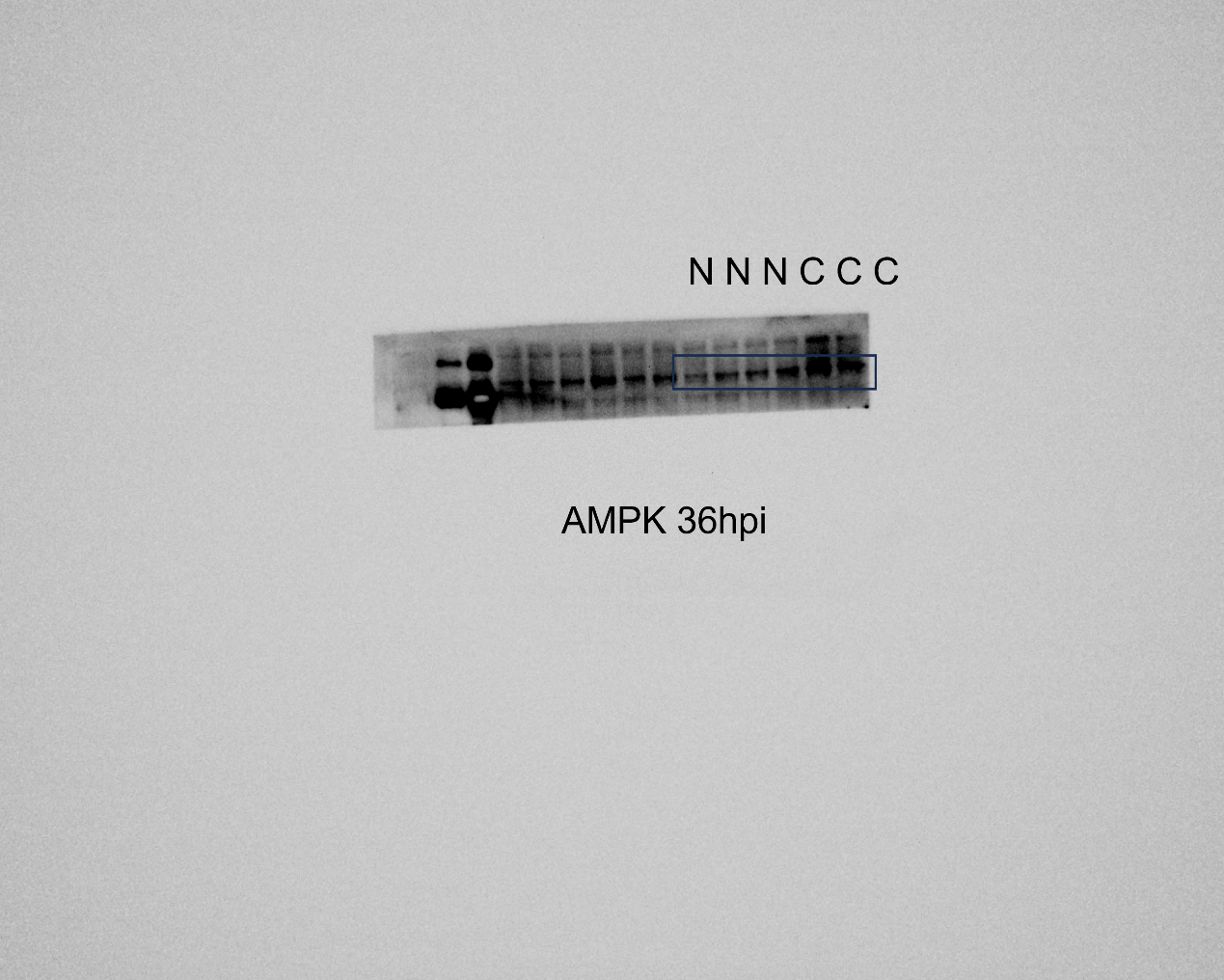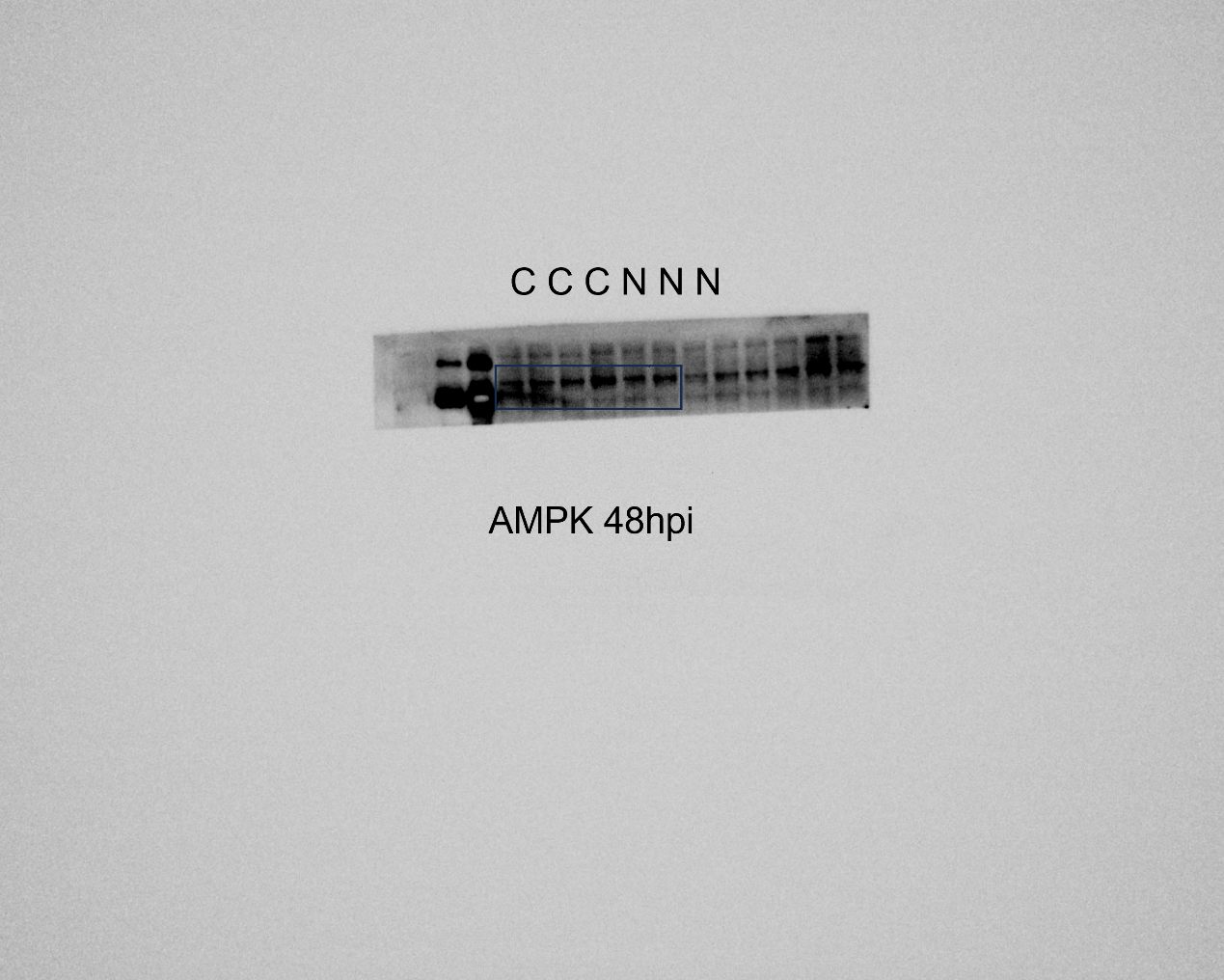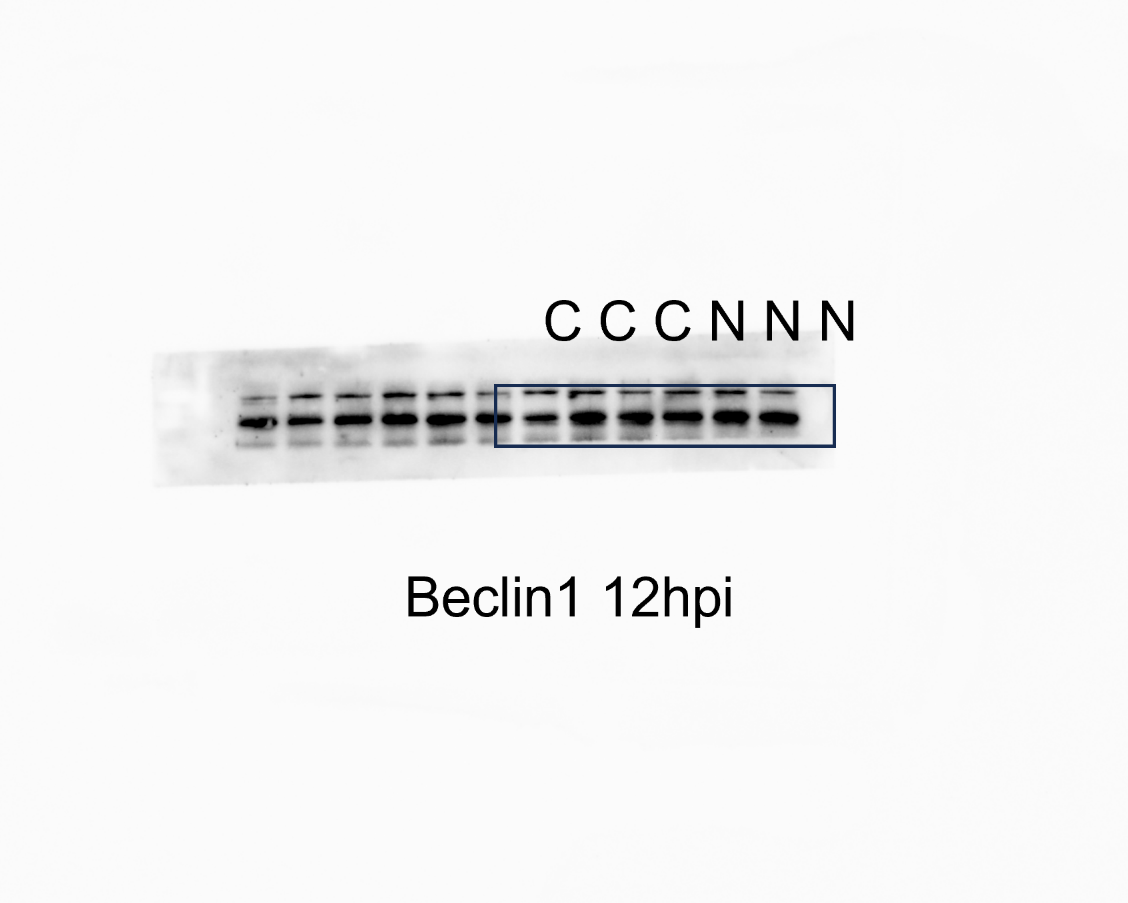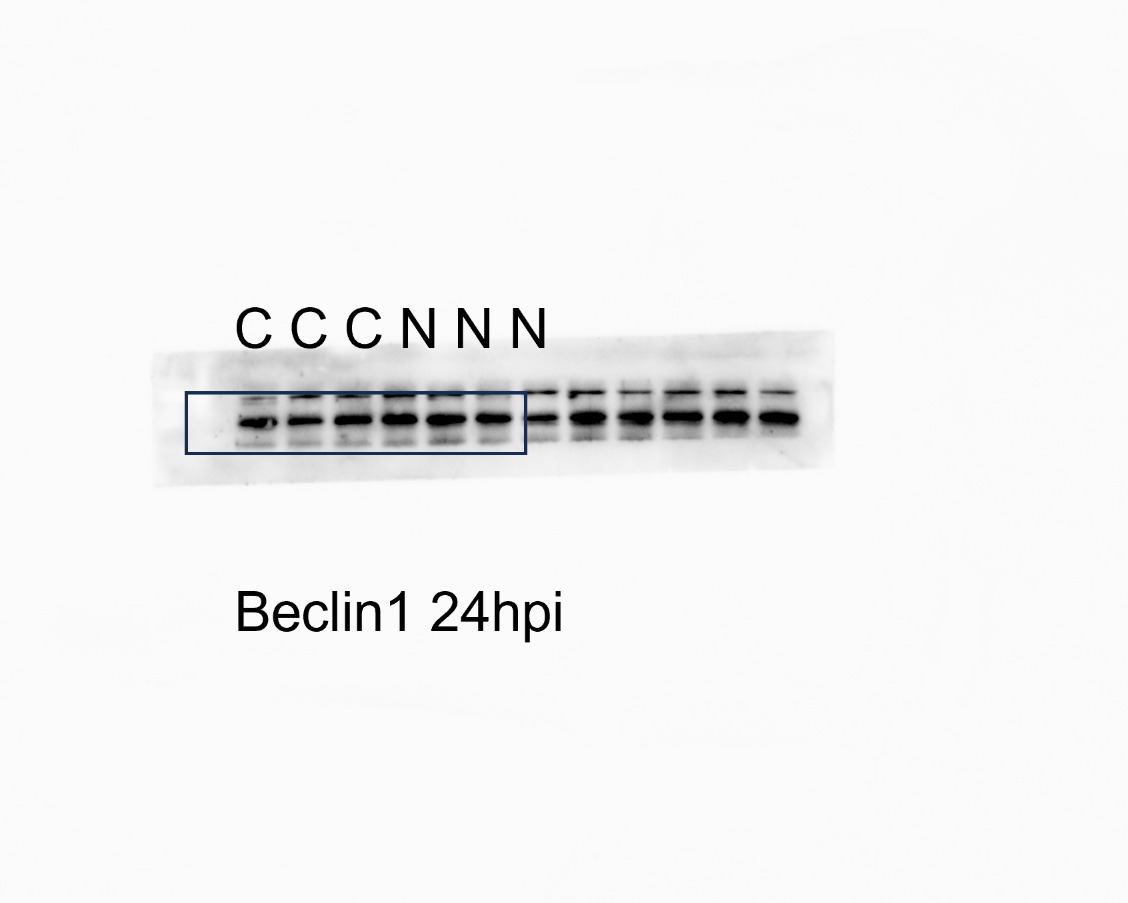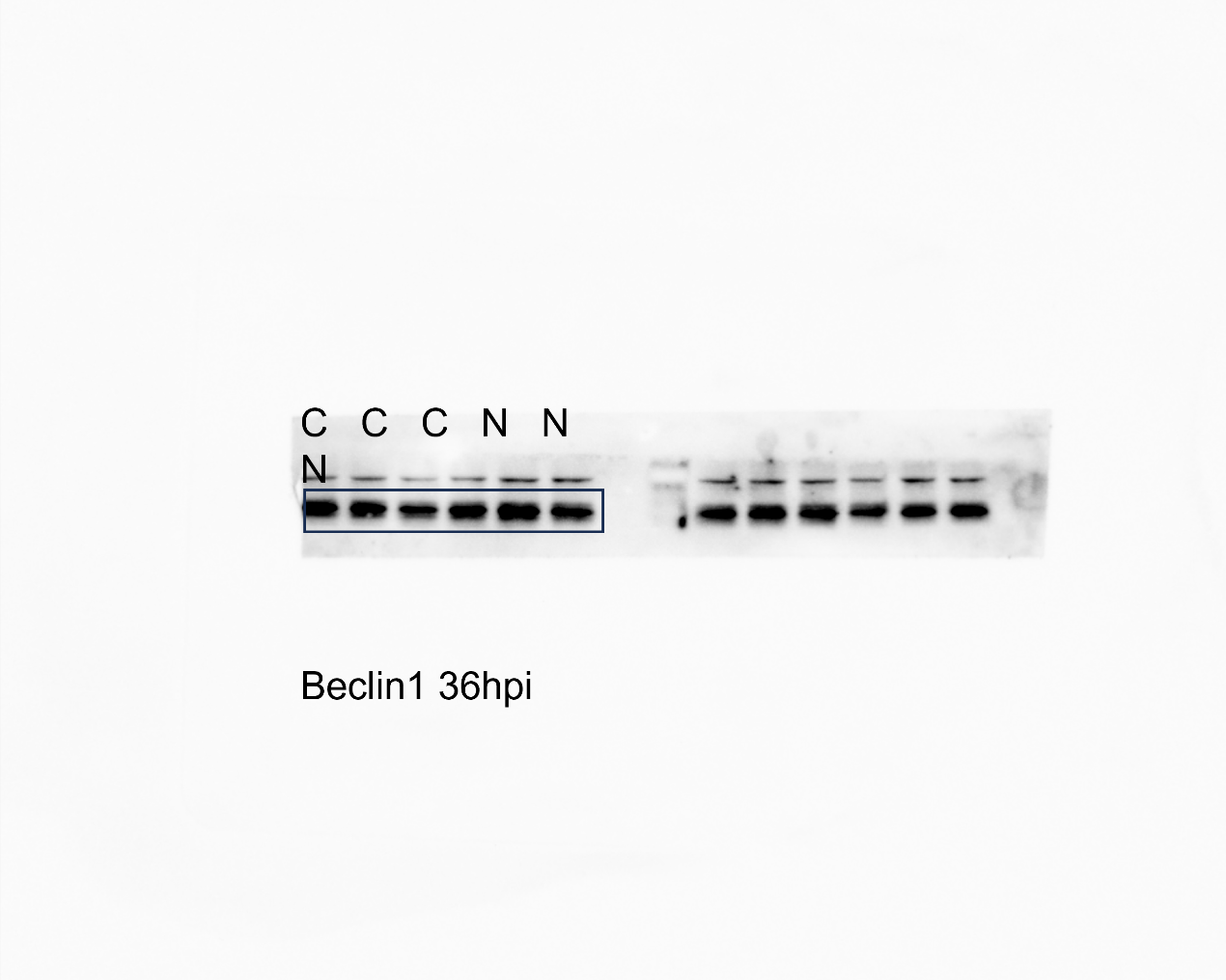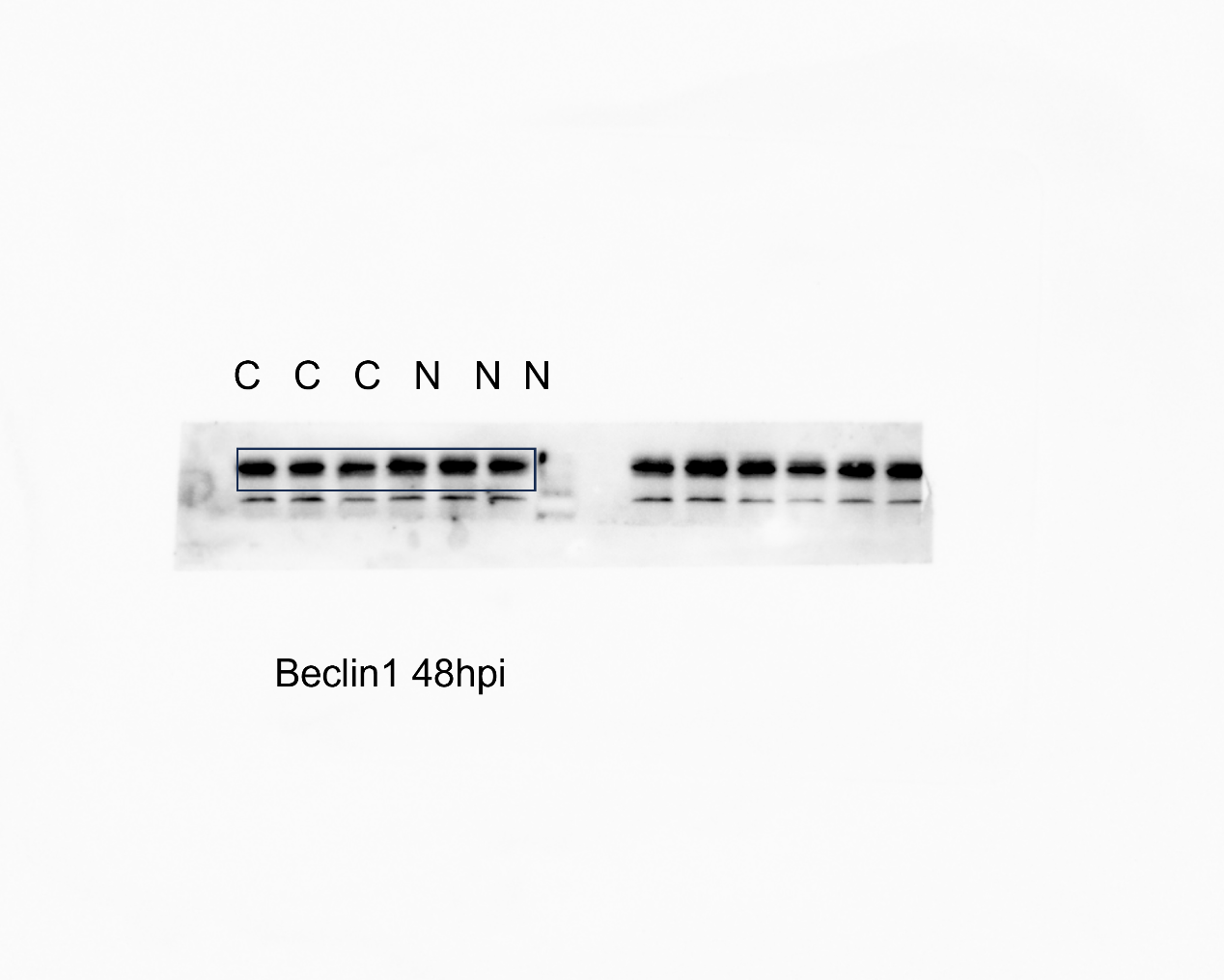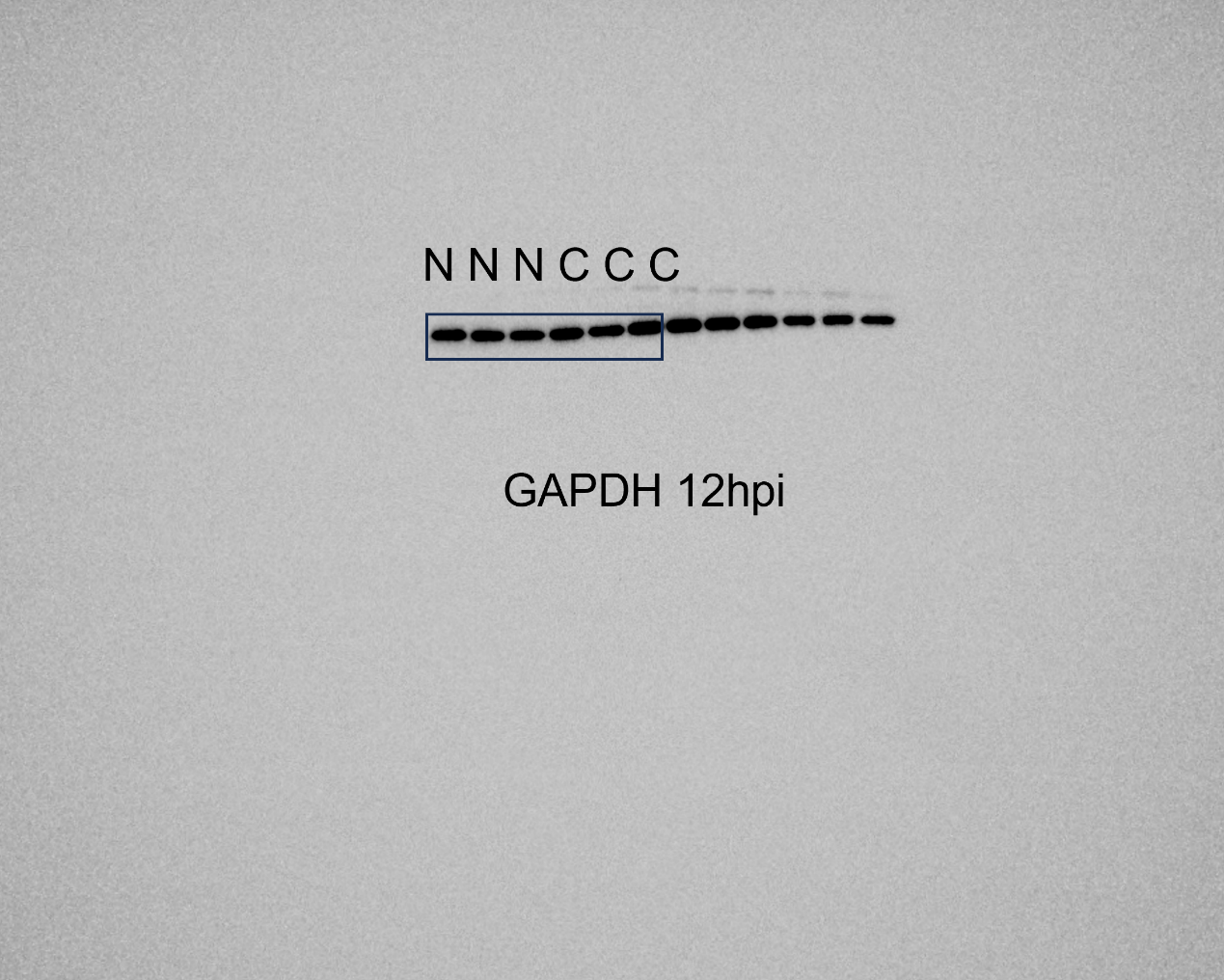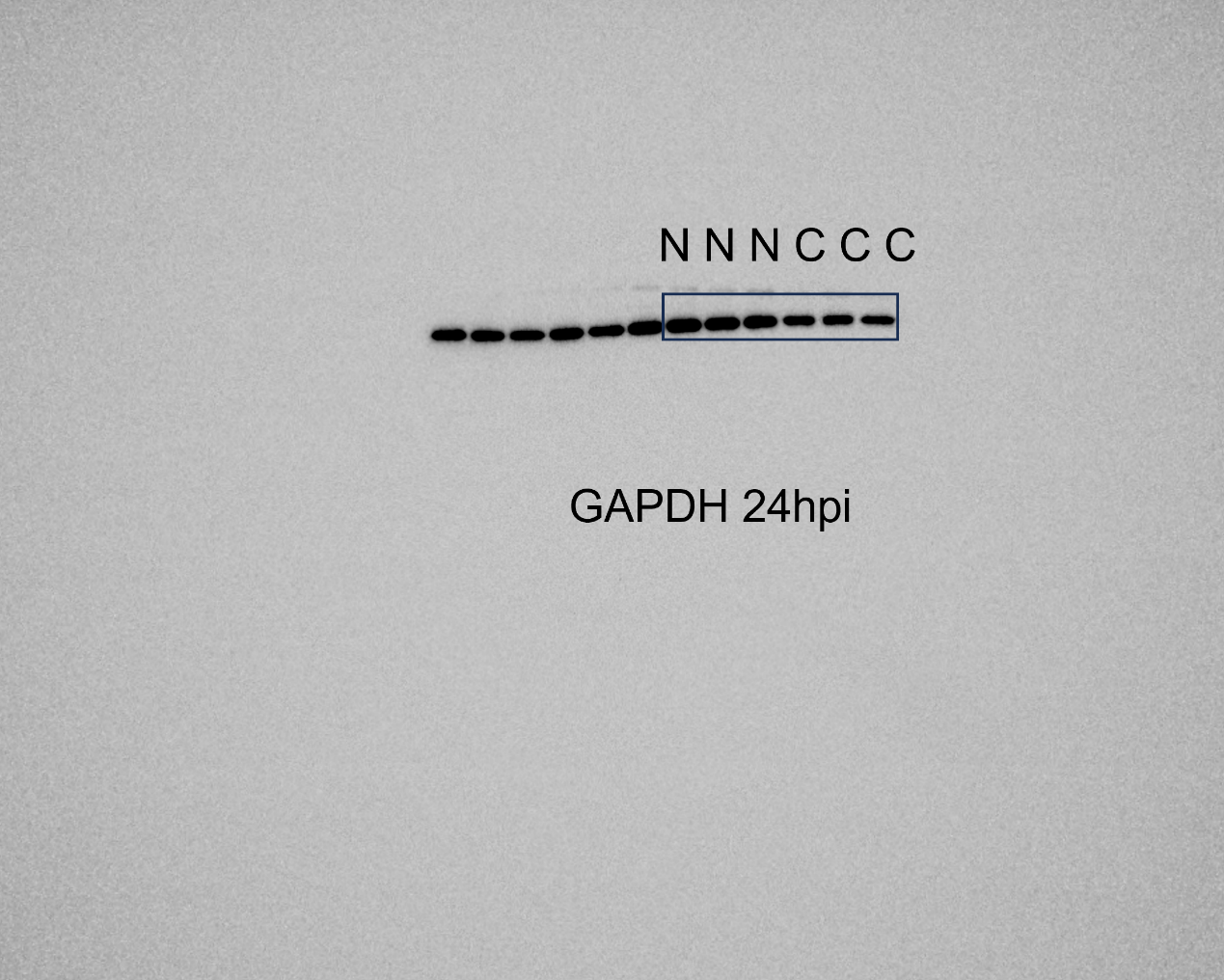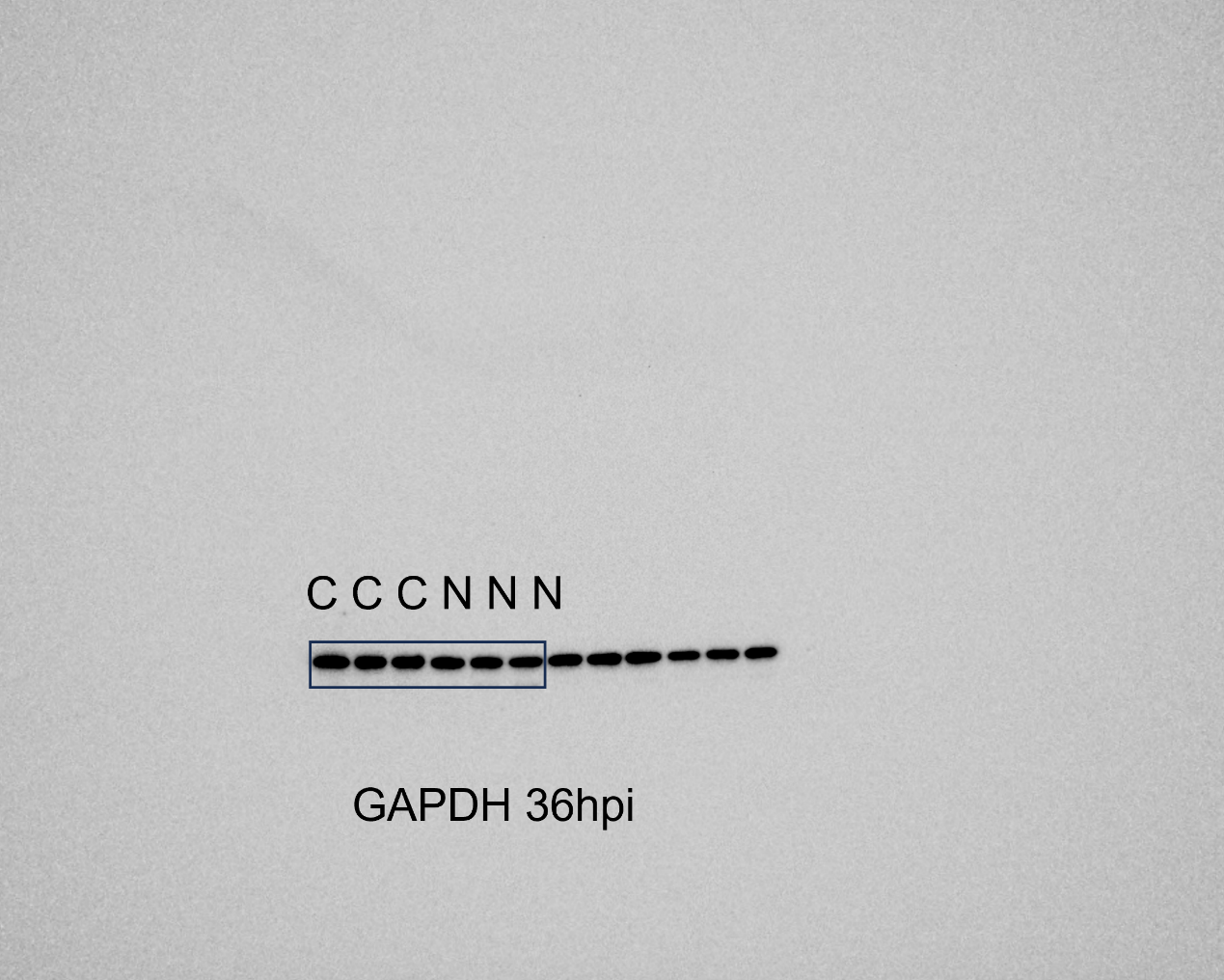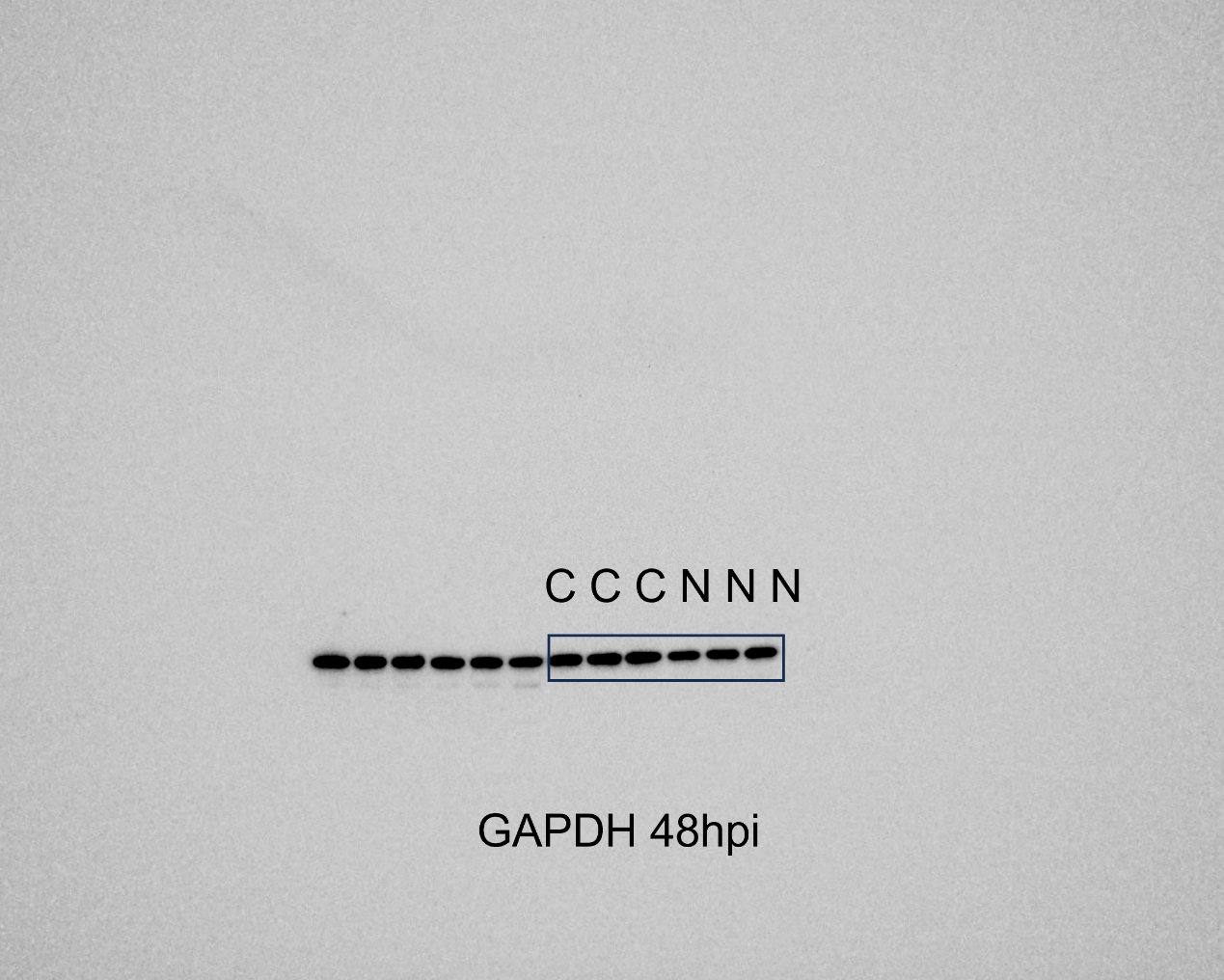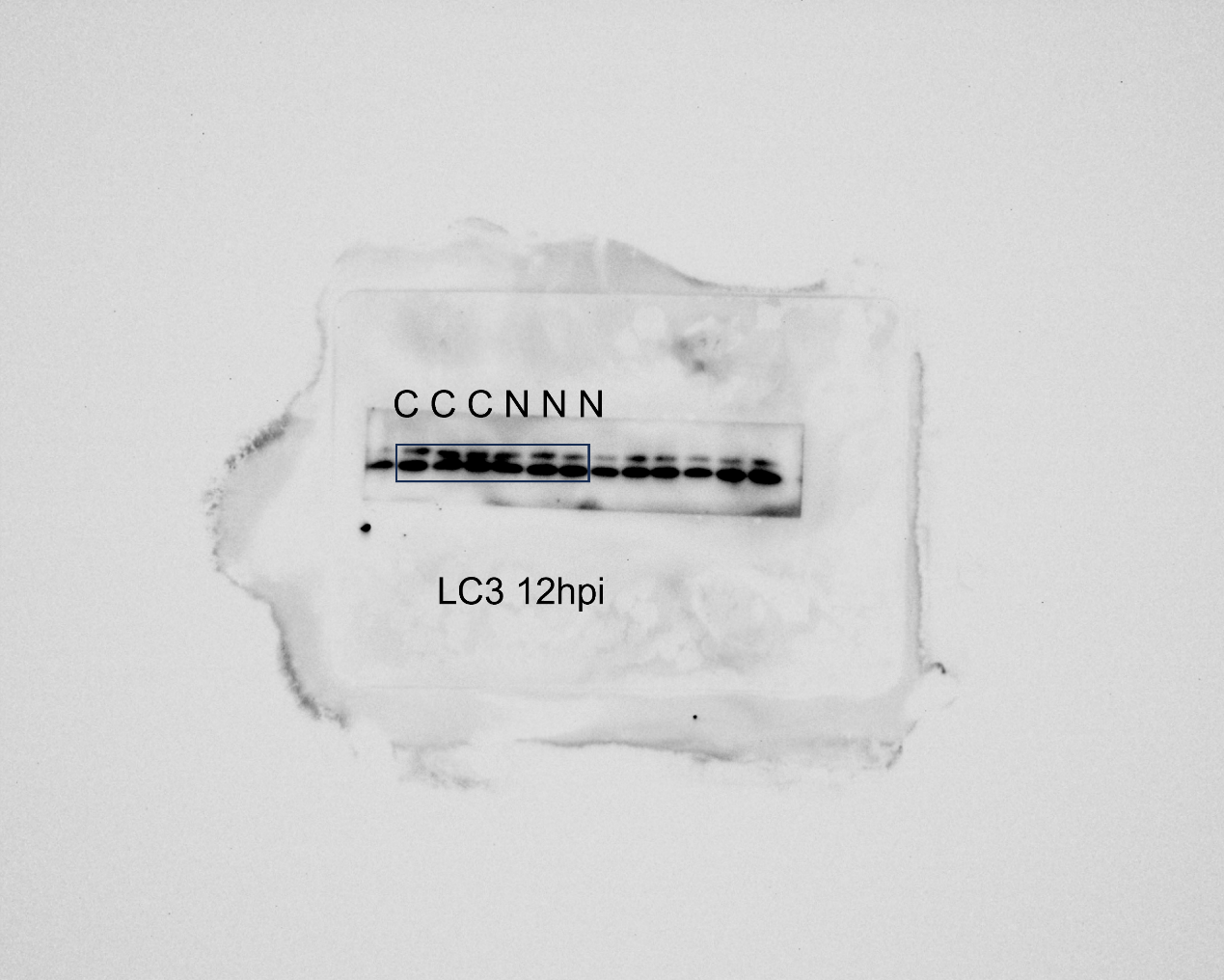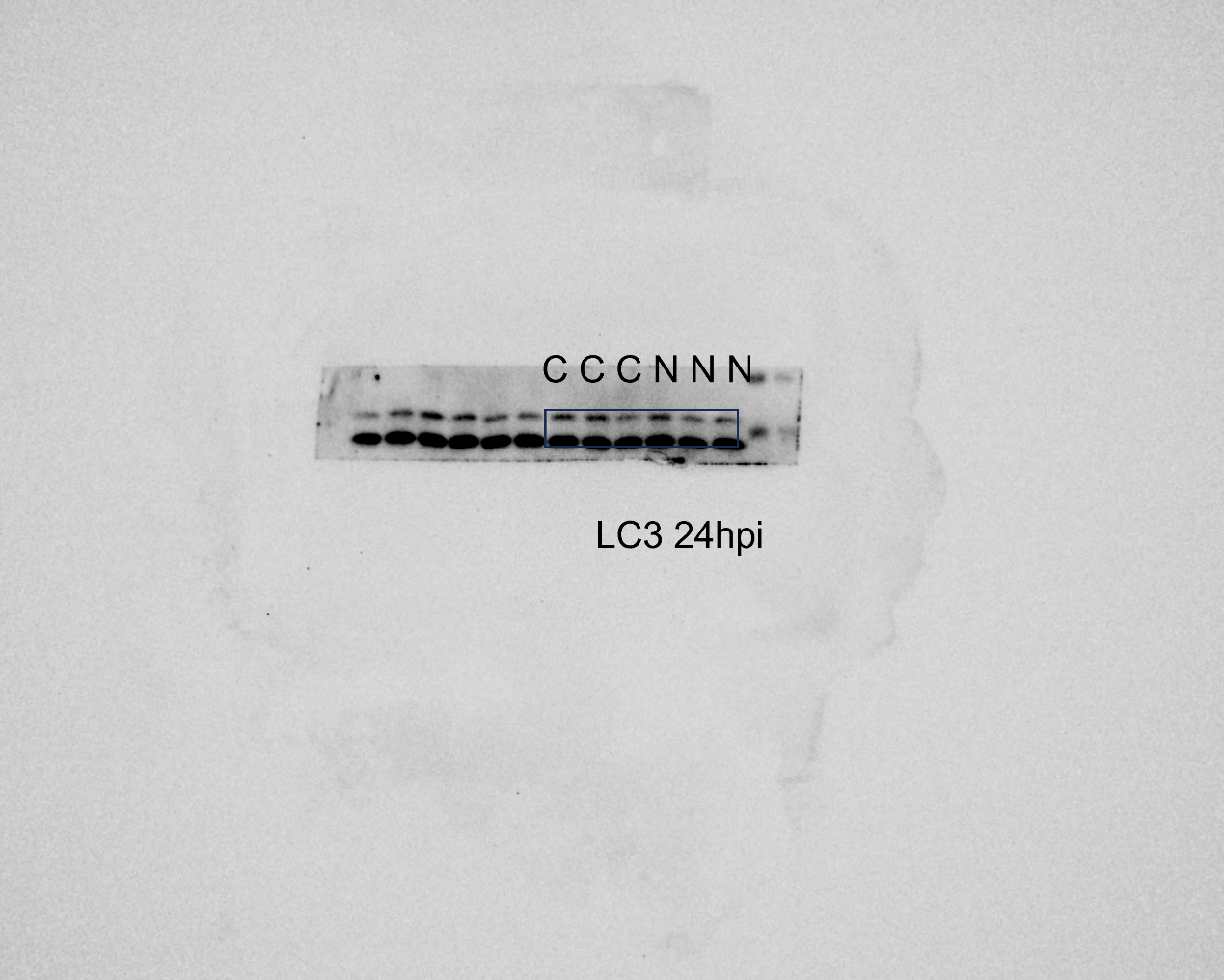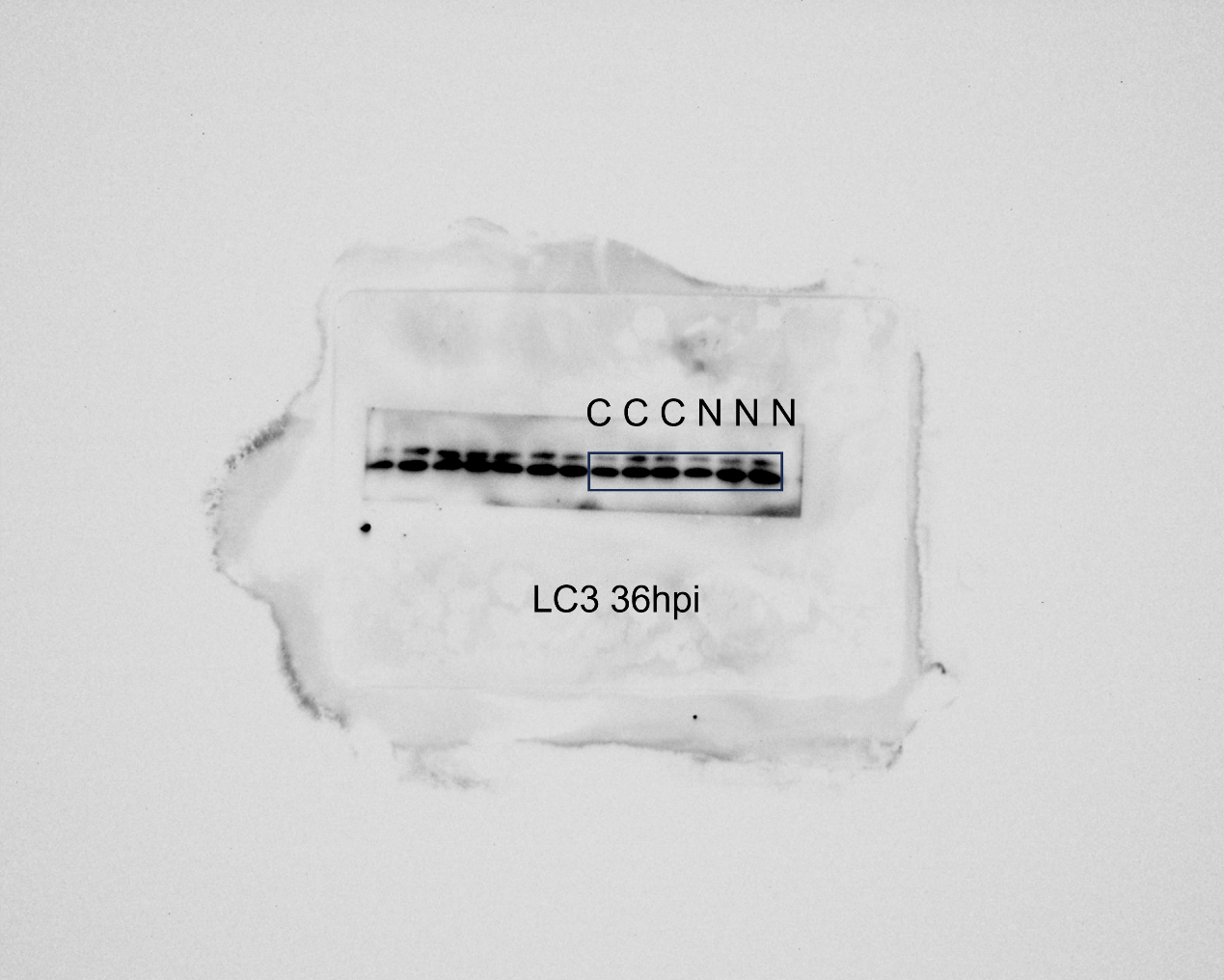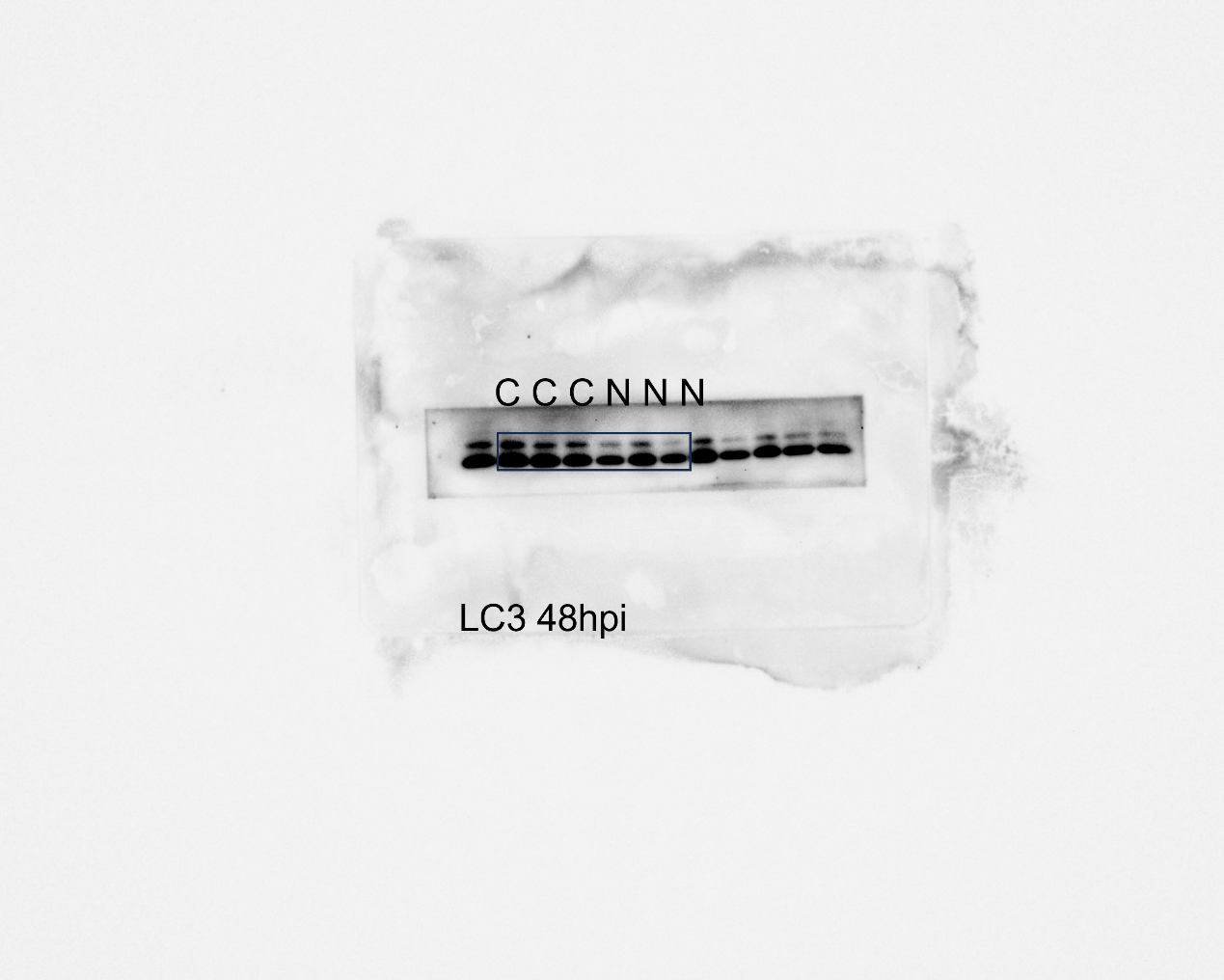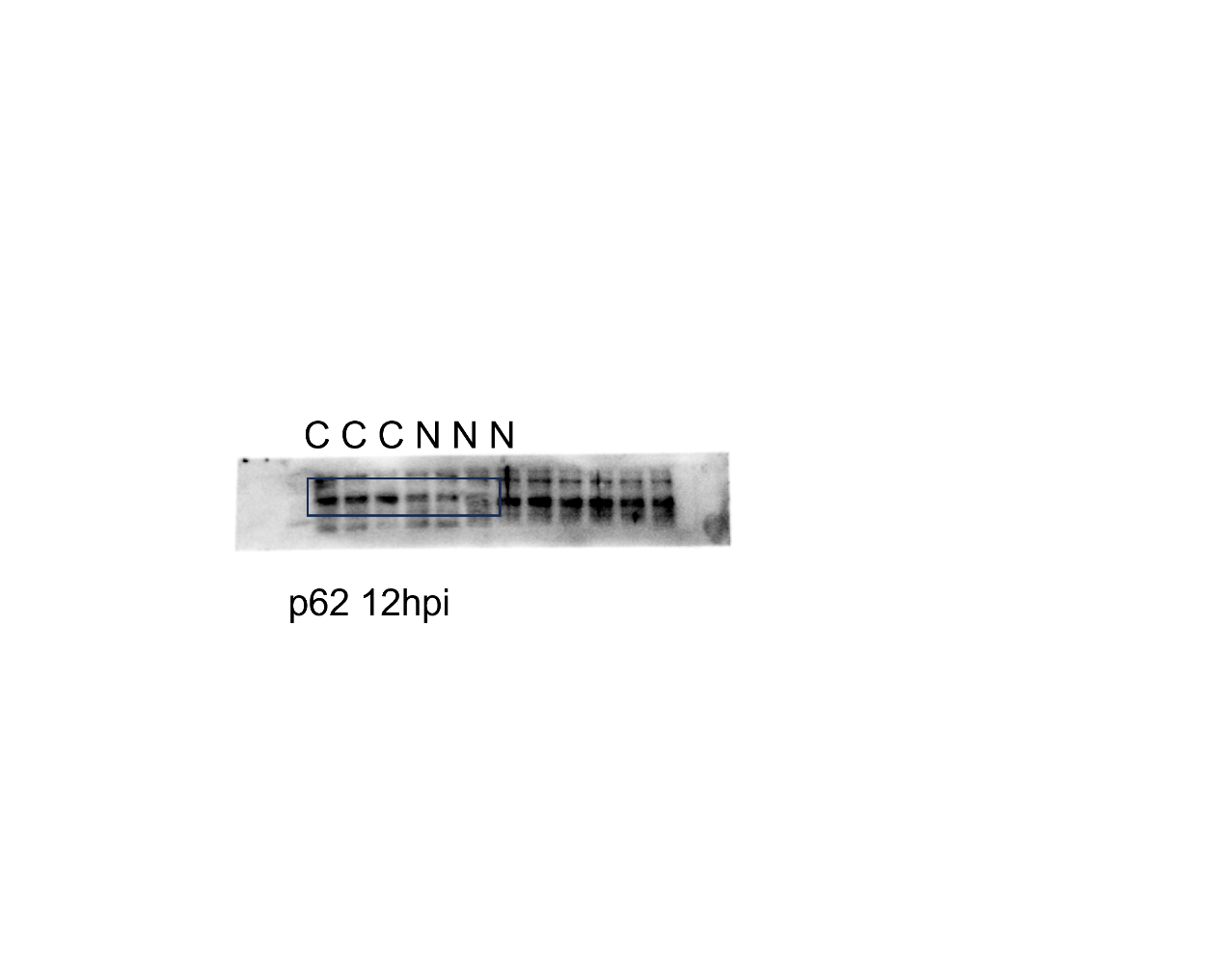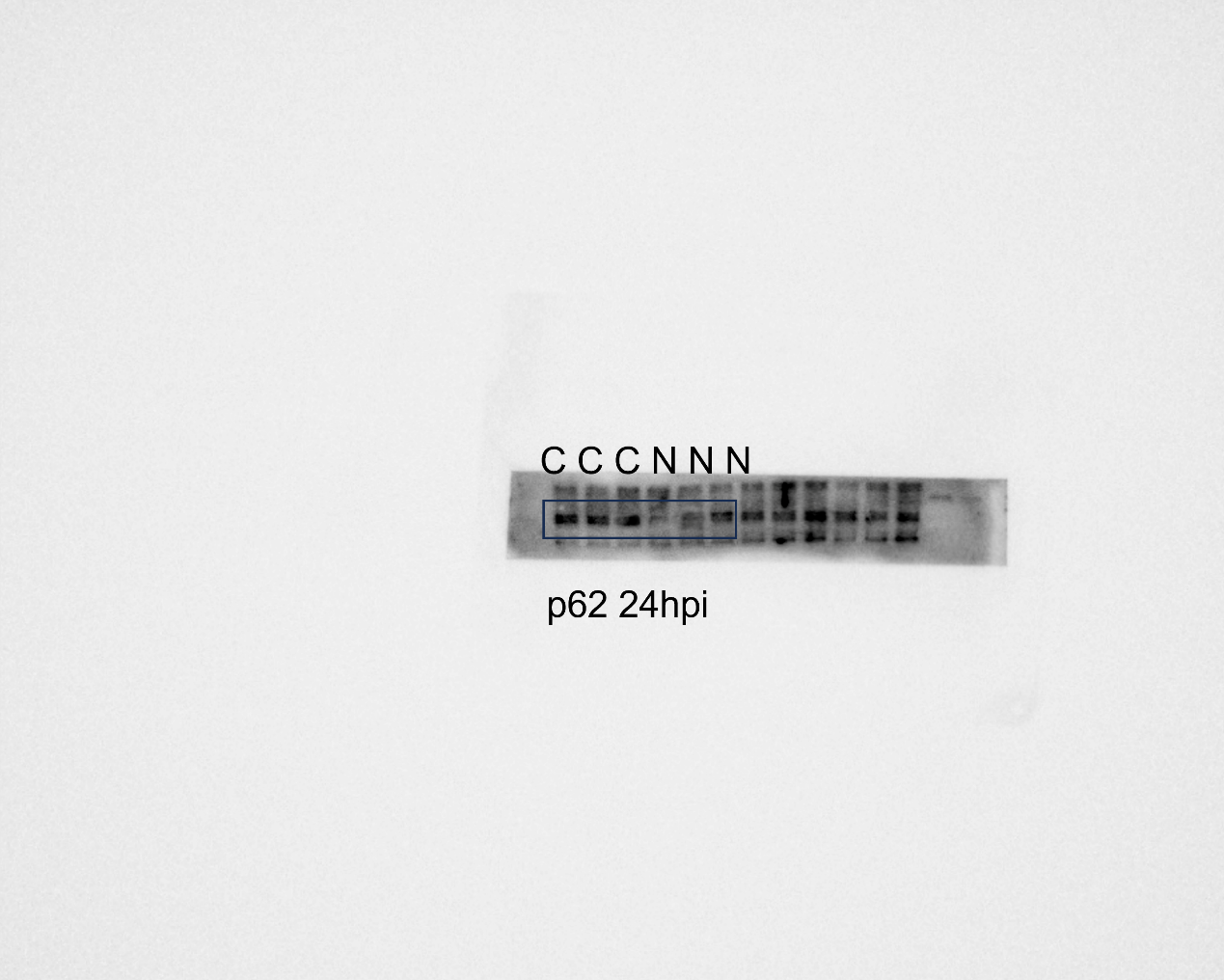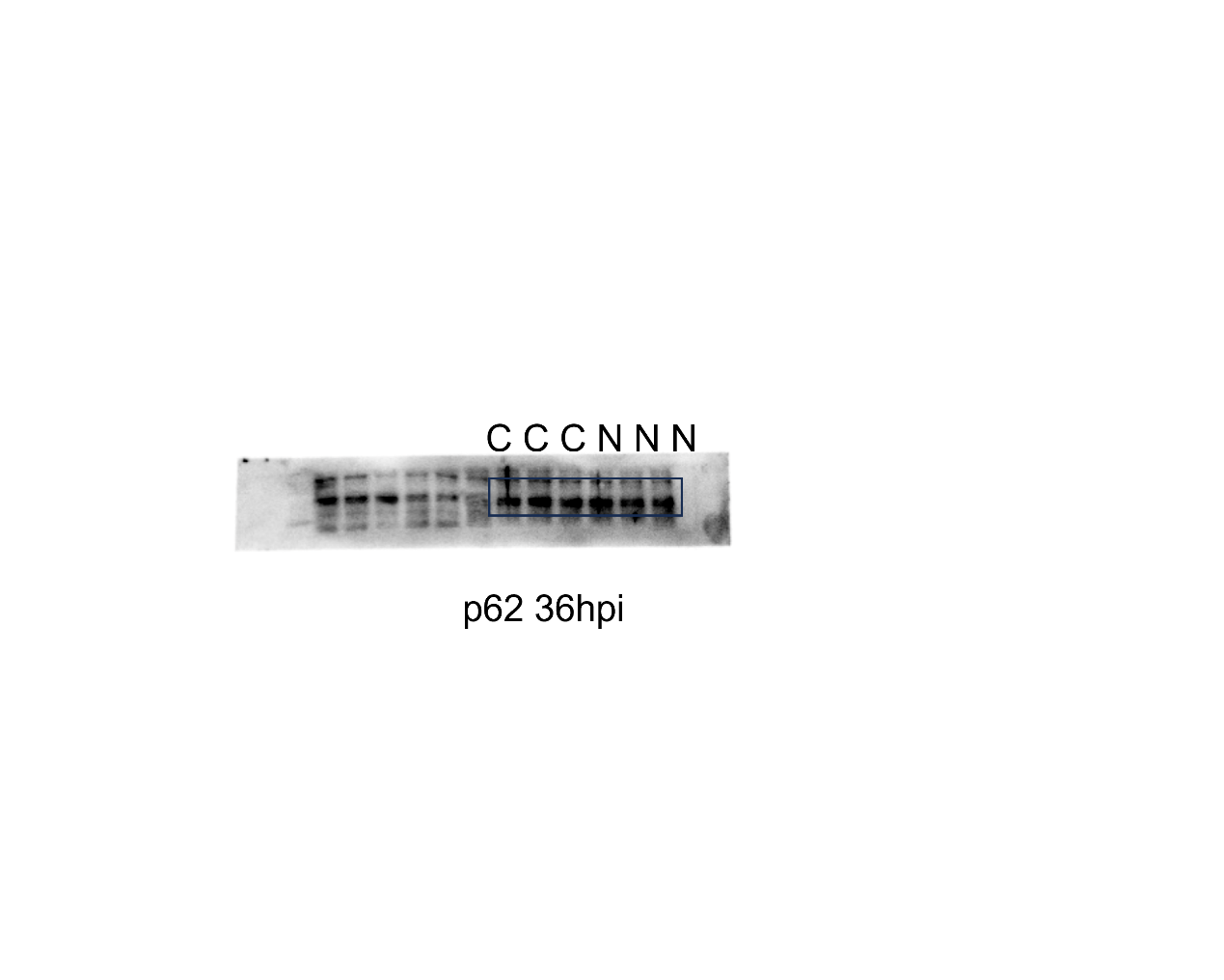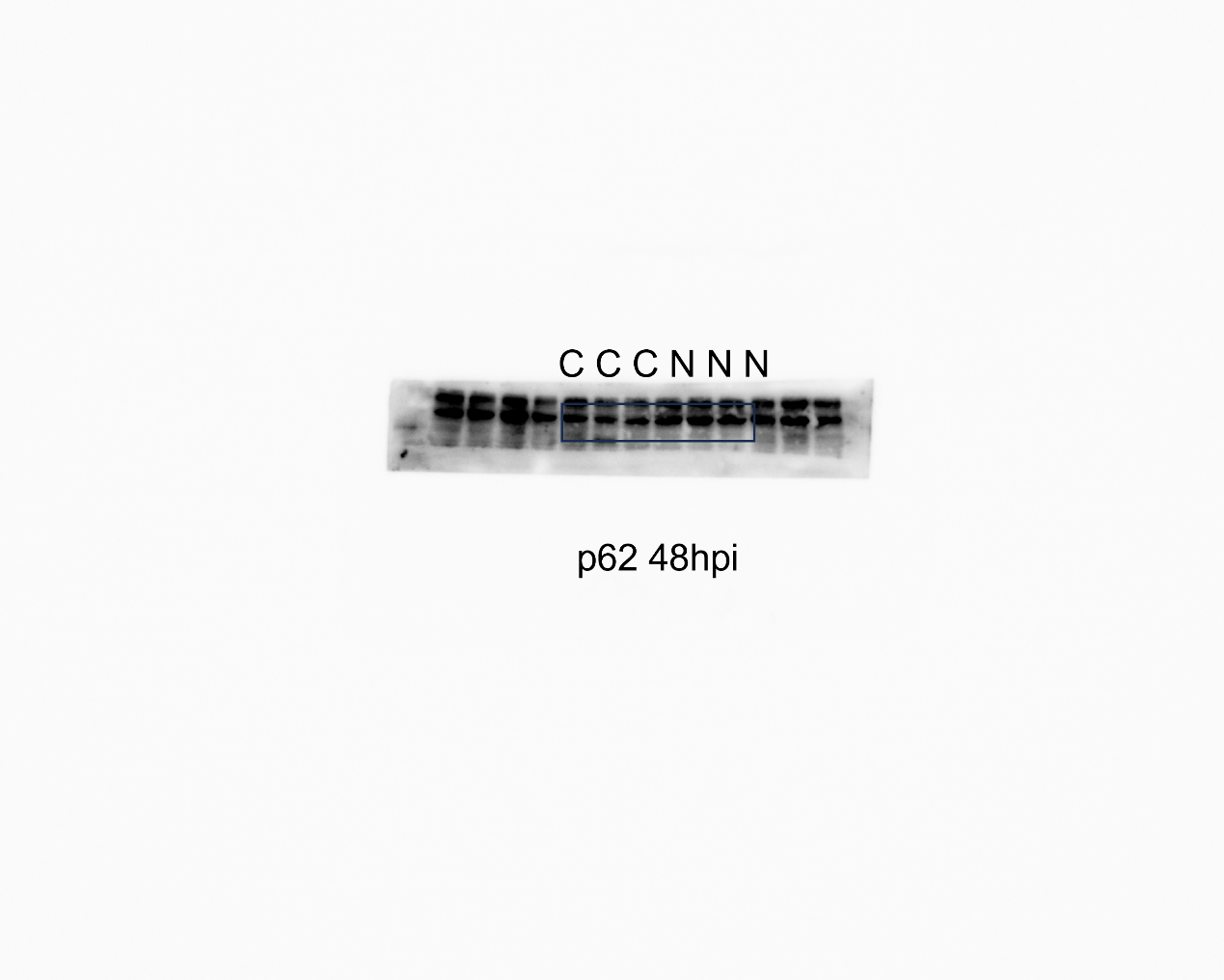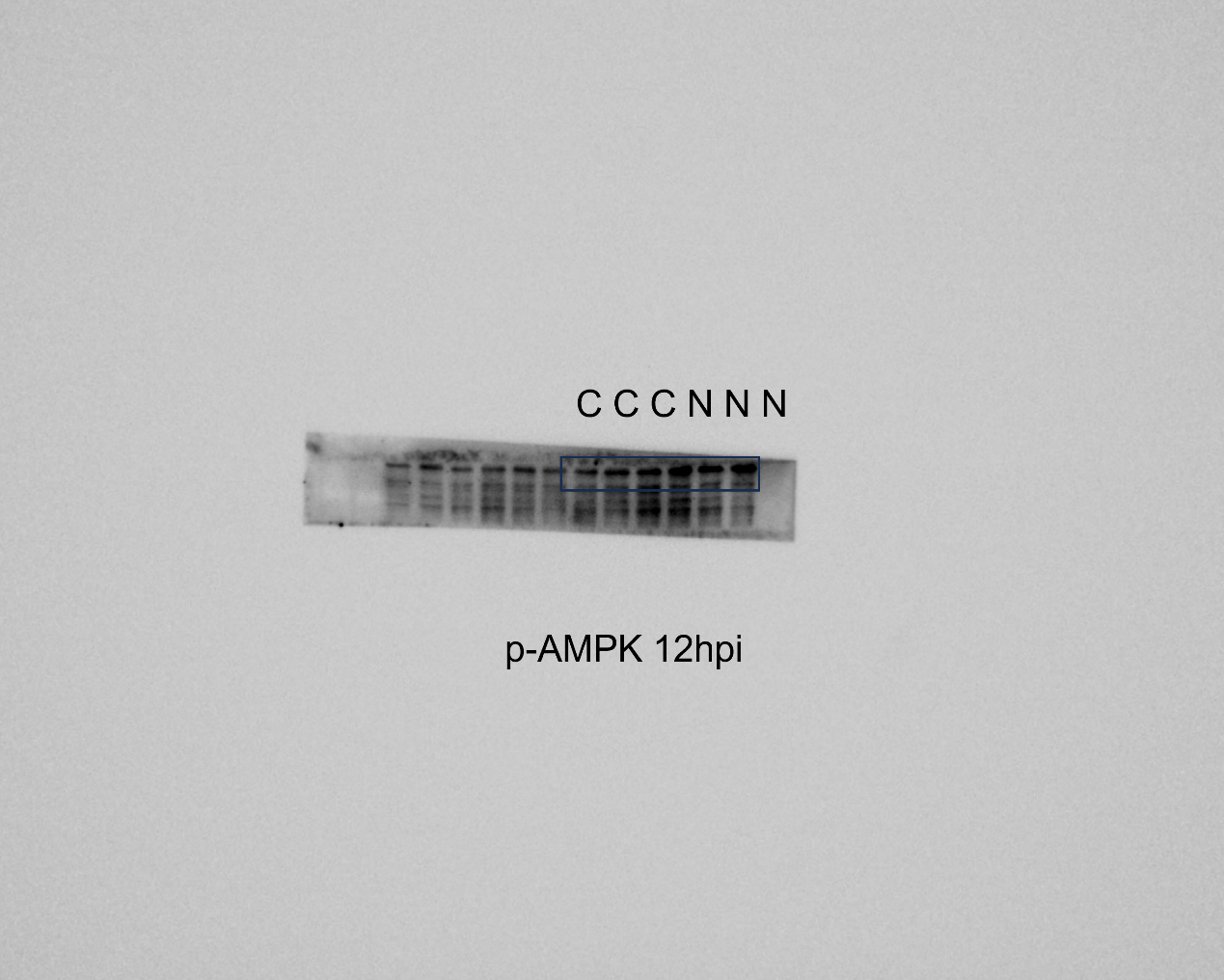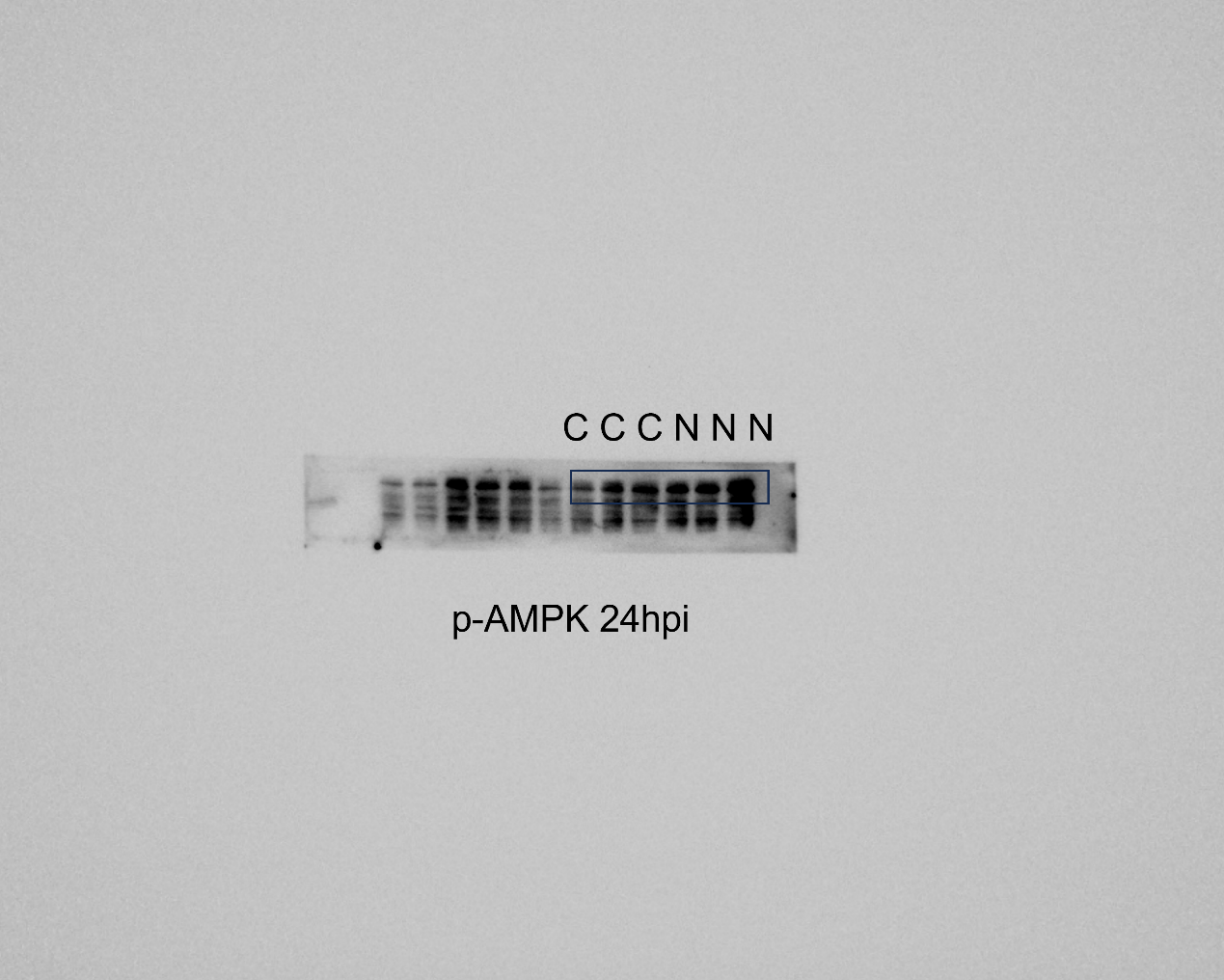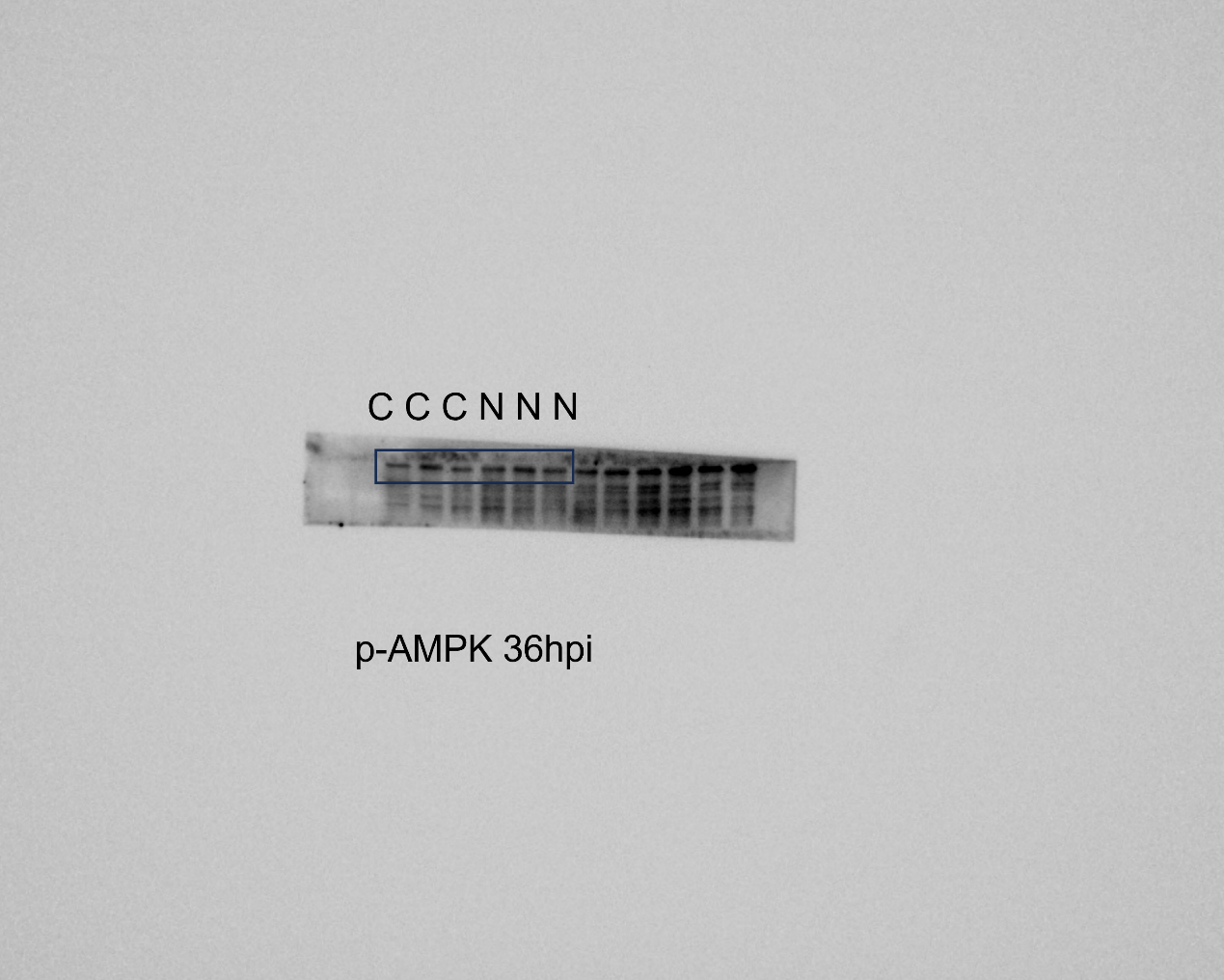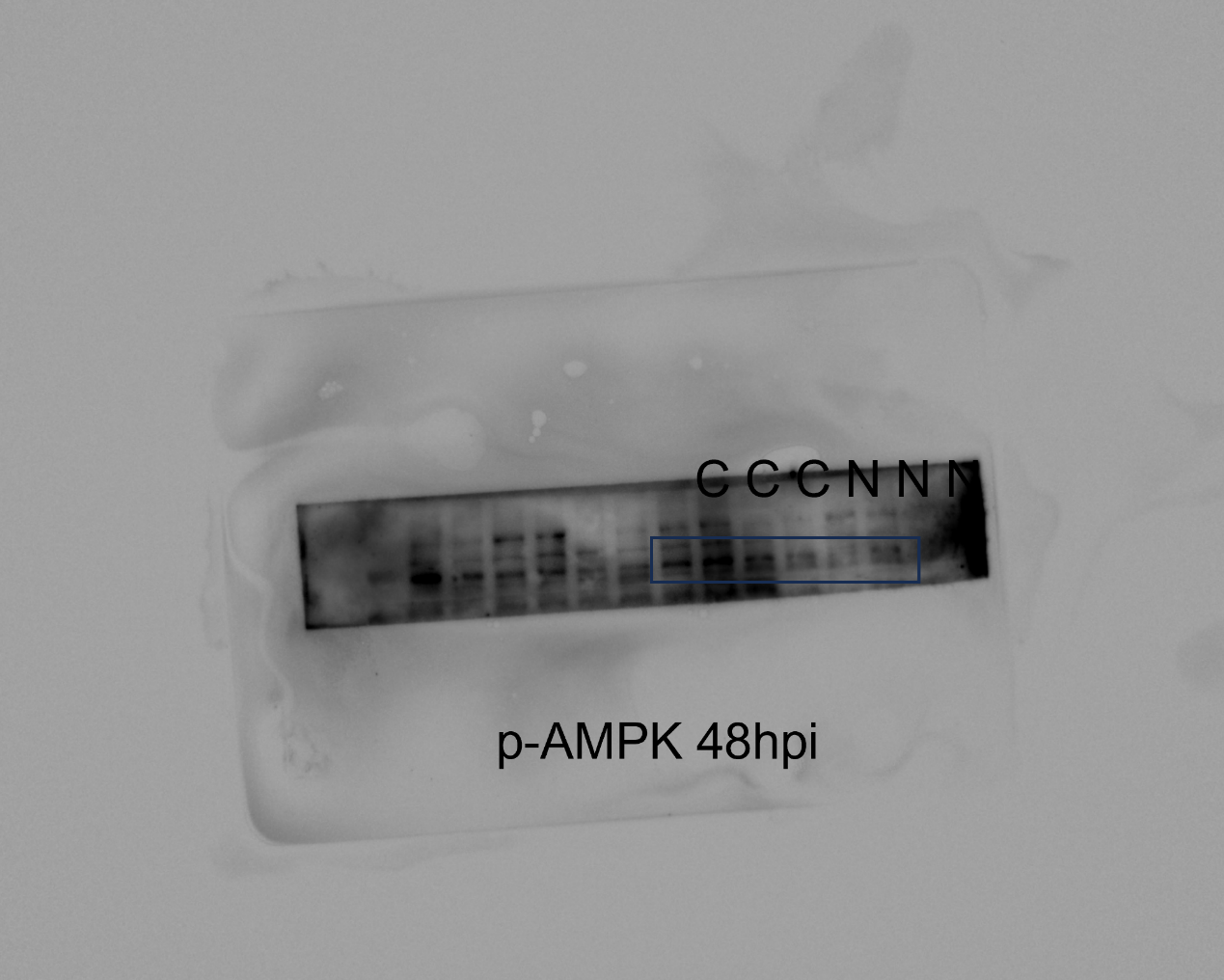** | | |
| **Figure.5 (D)** | | |
| **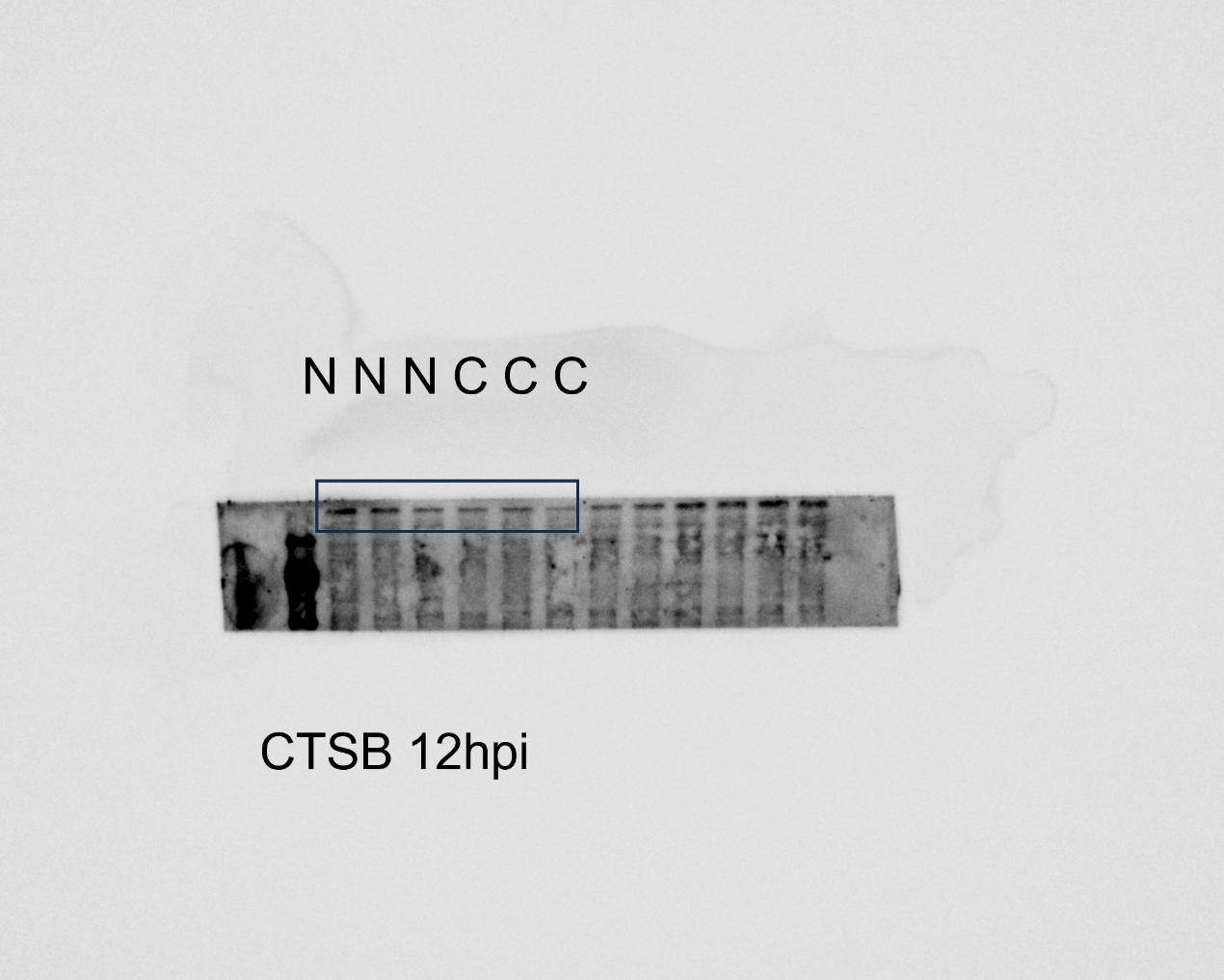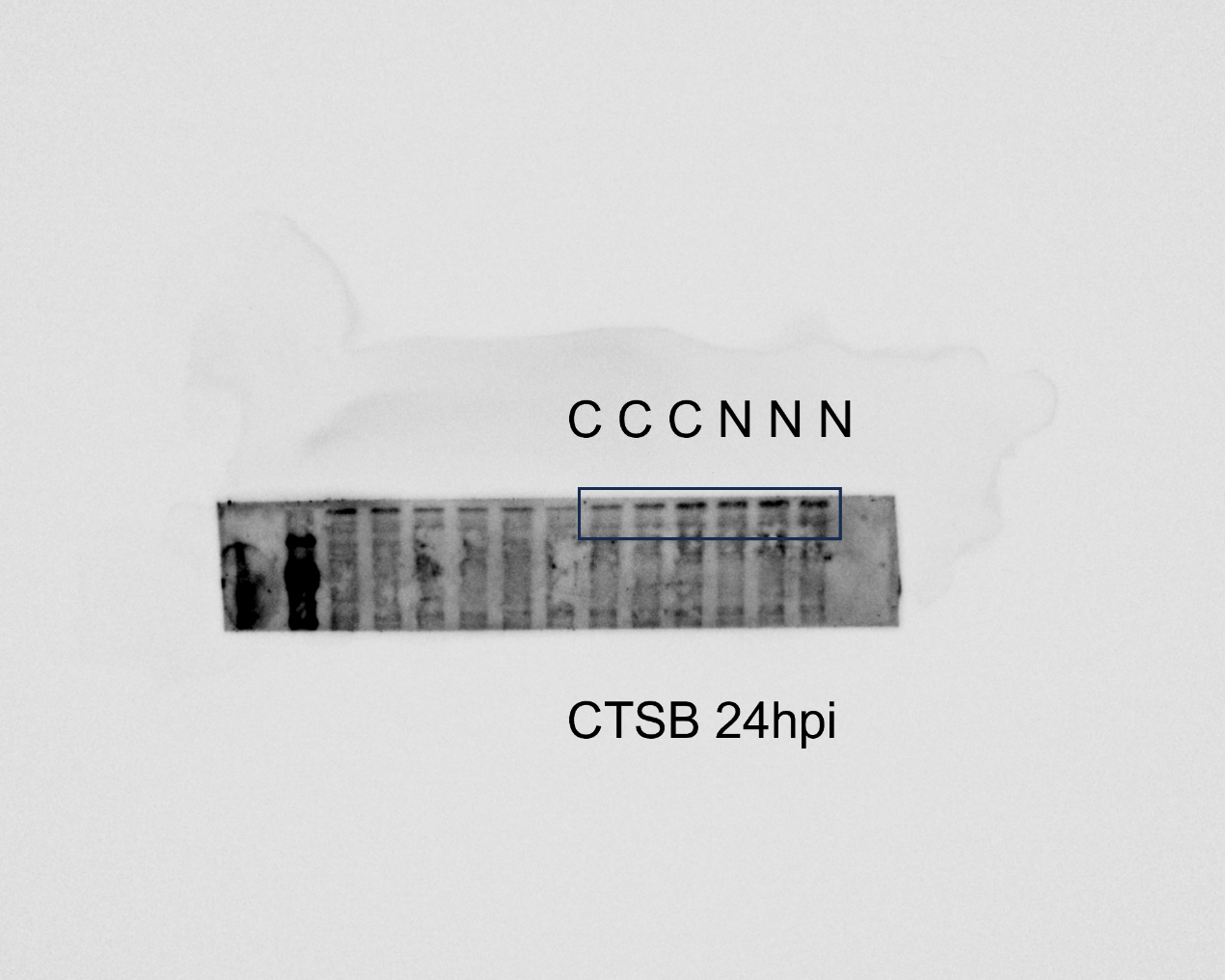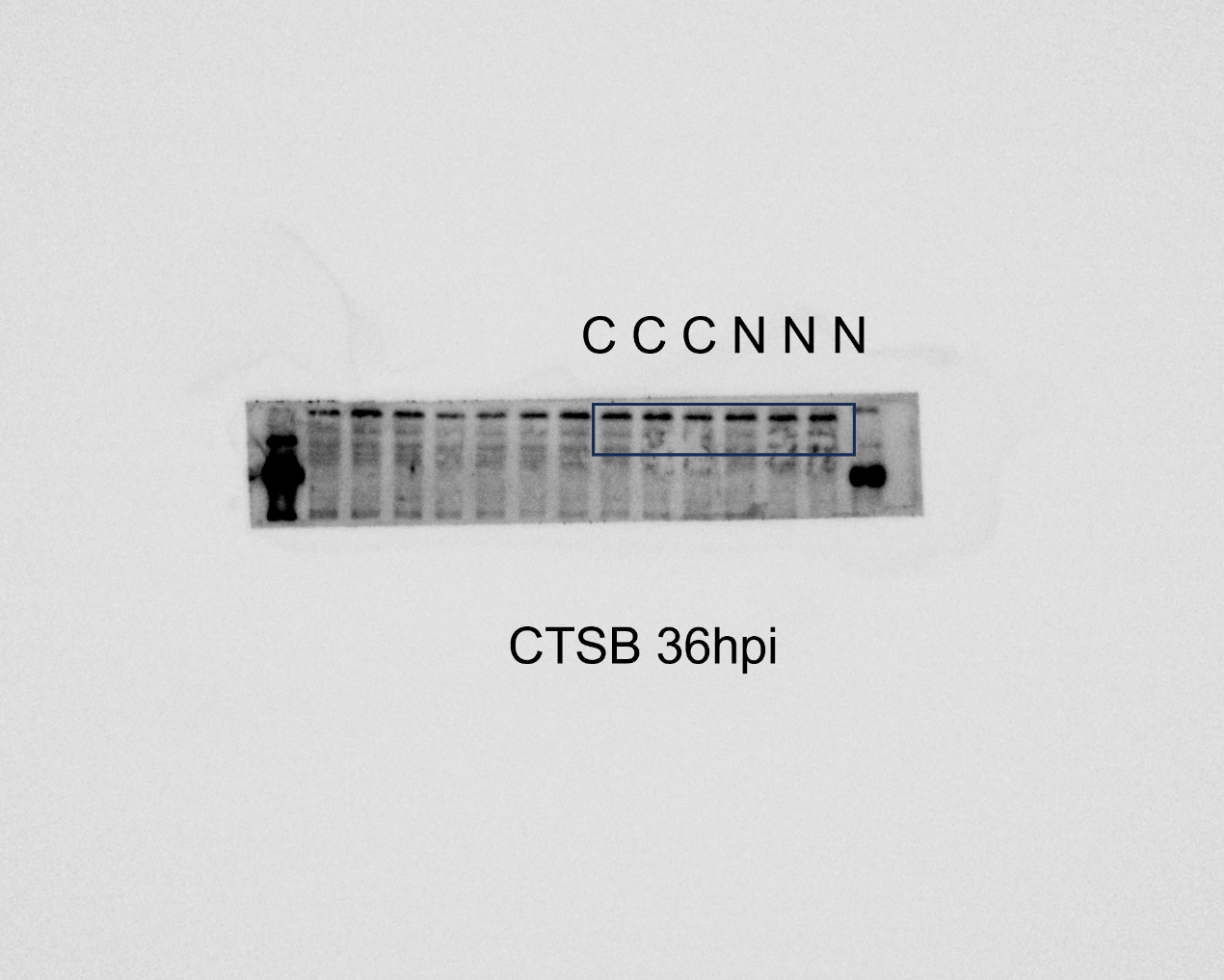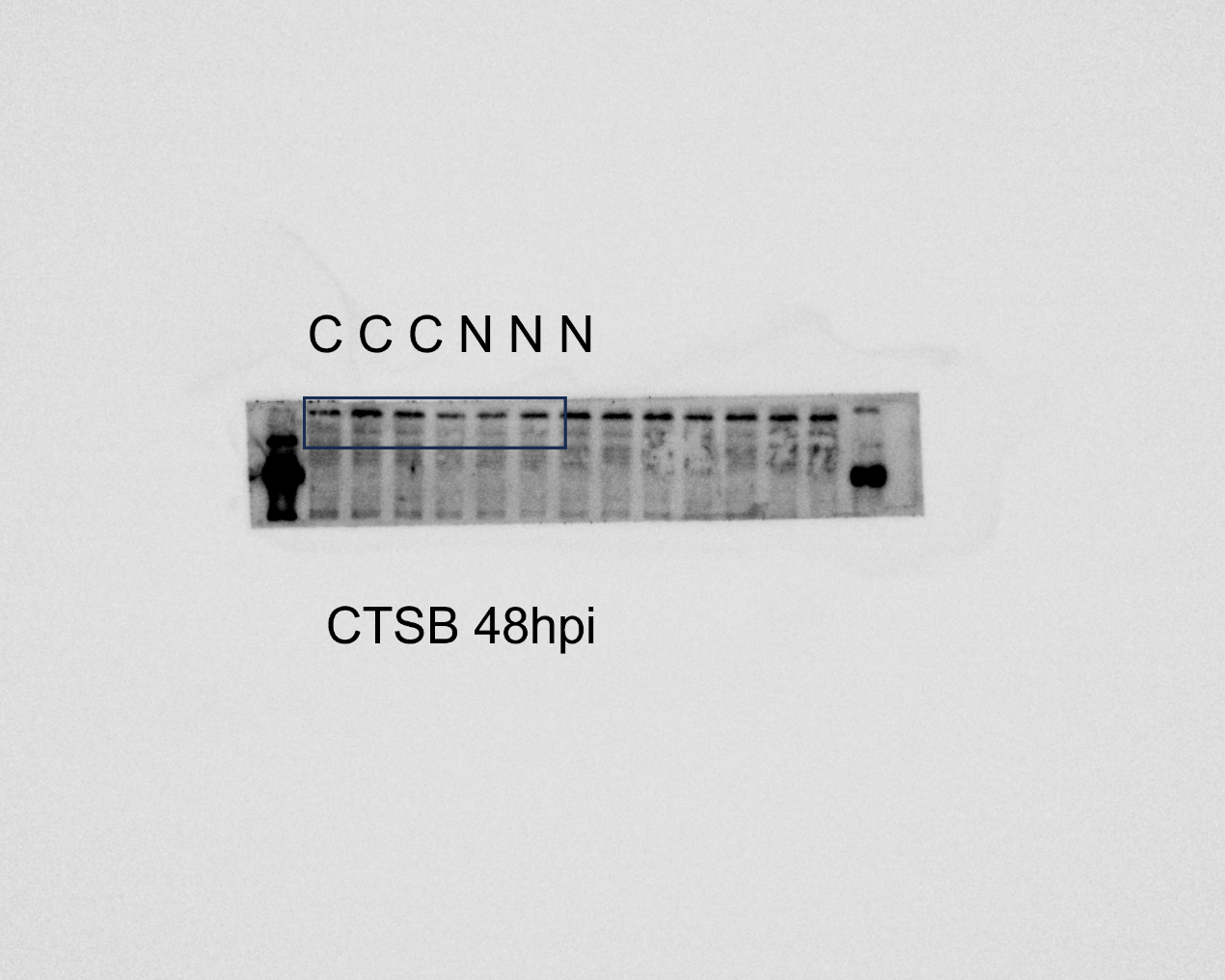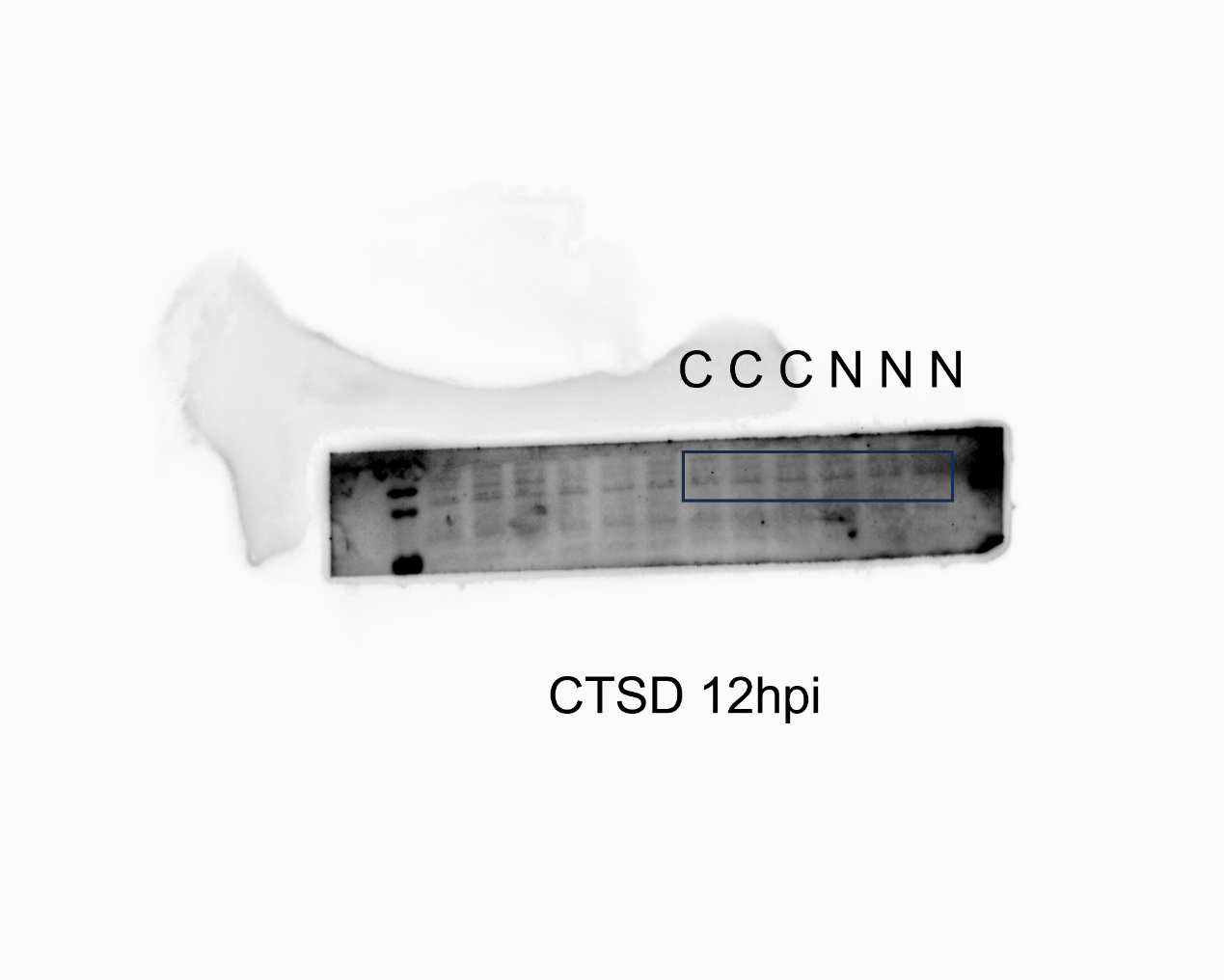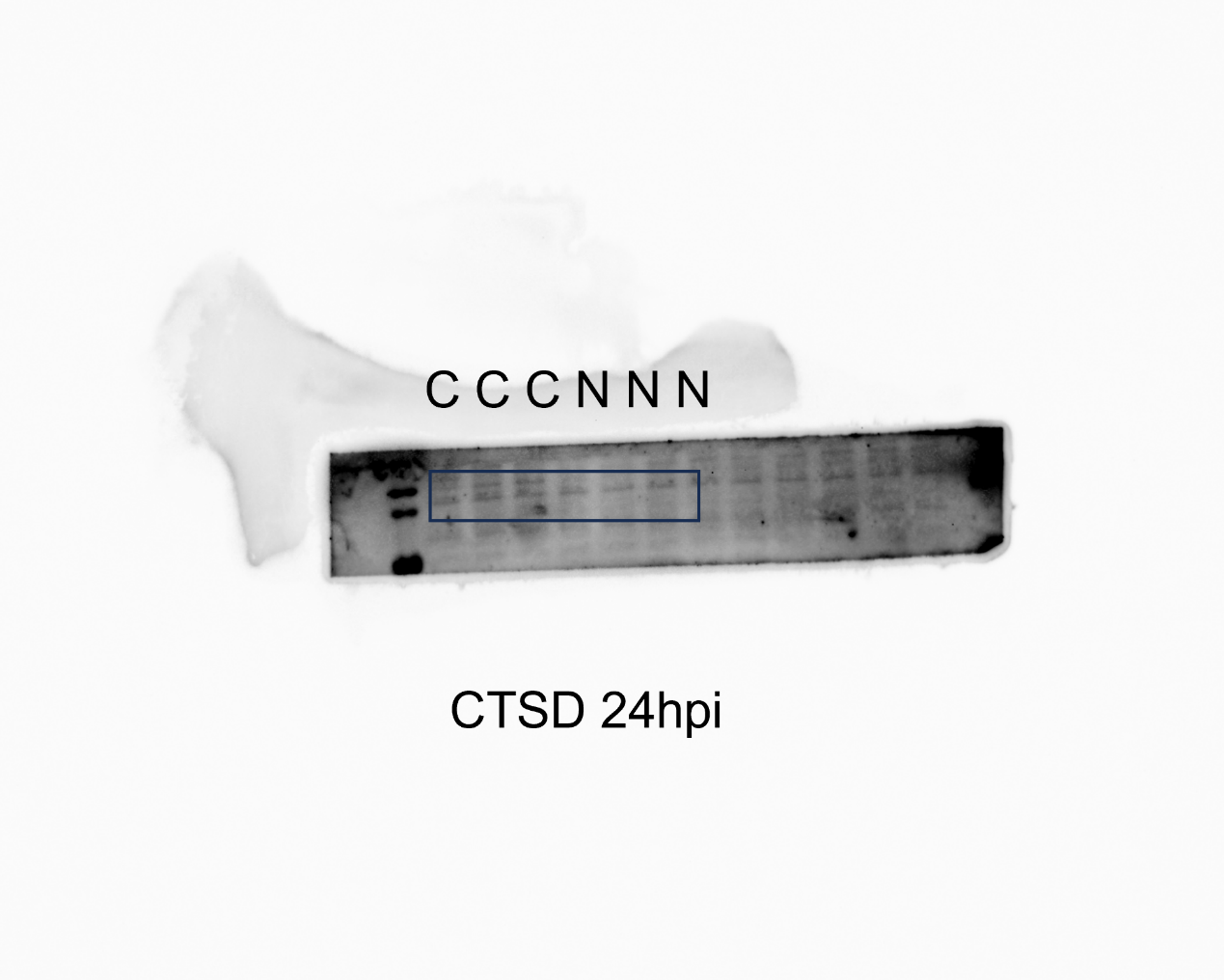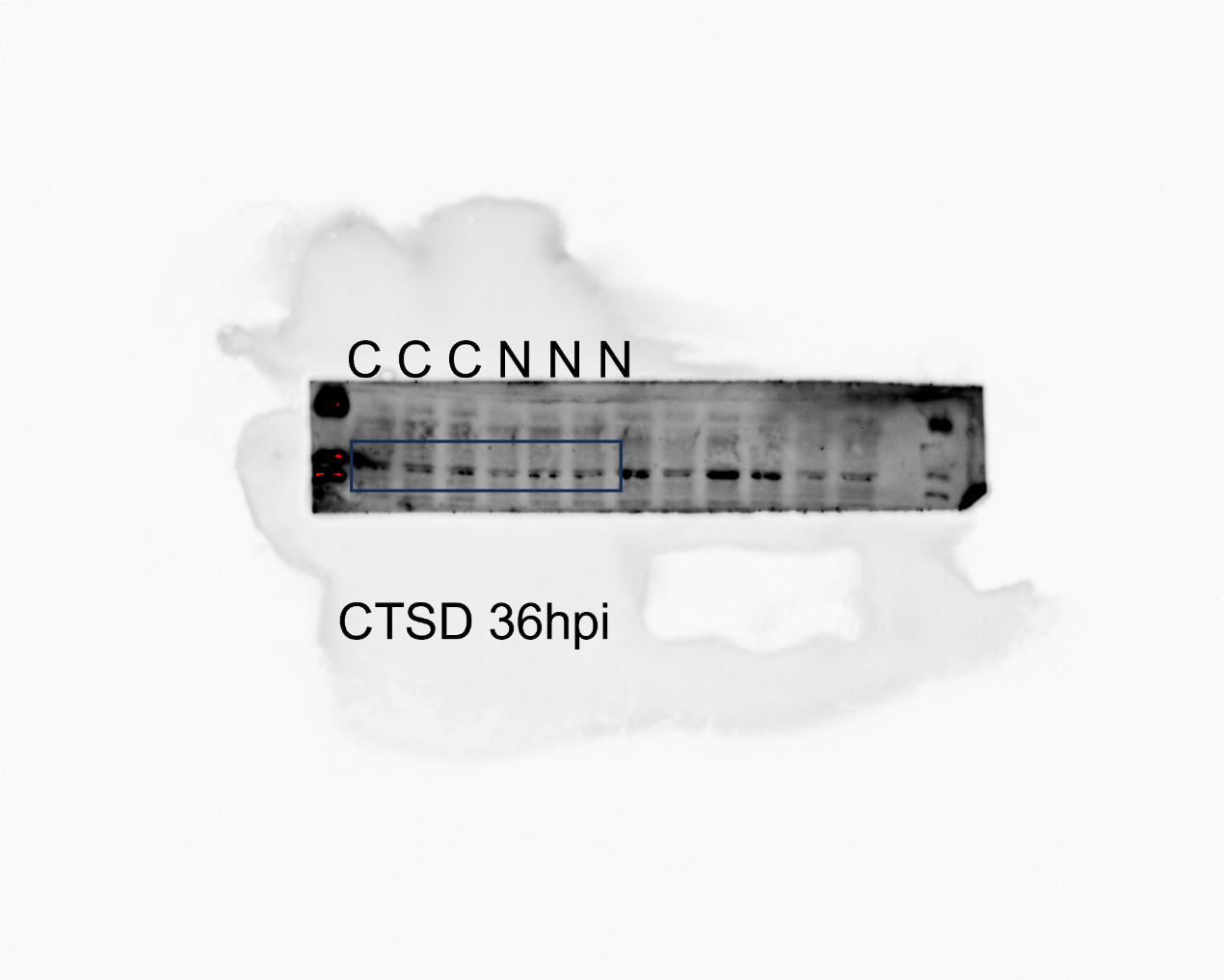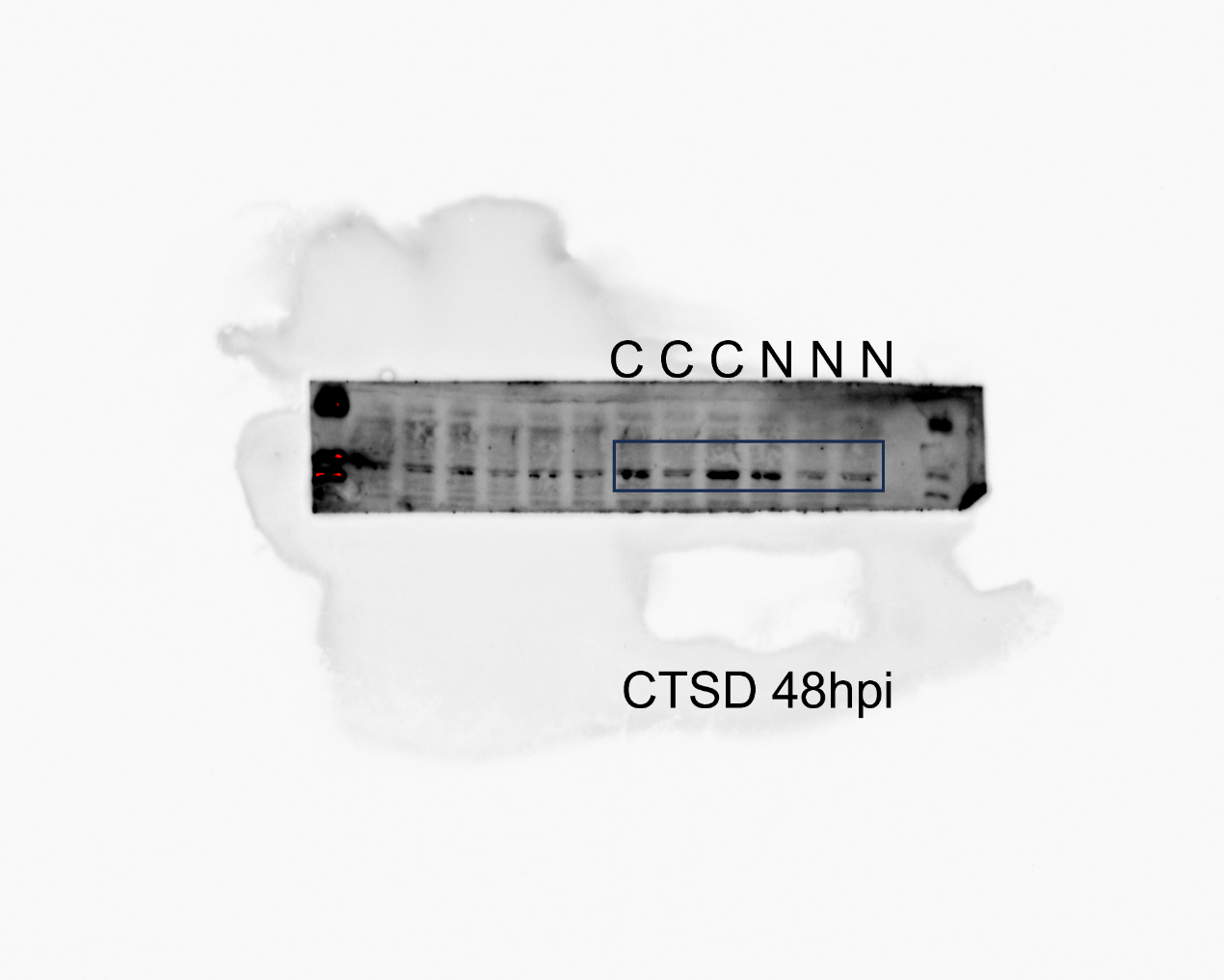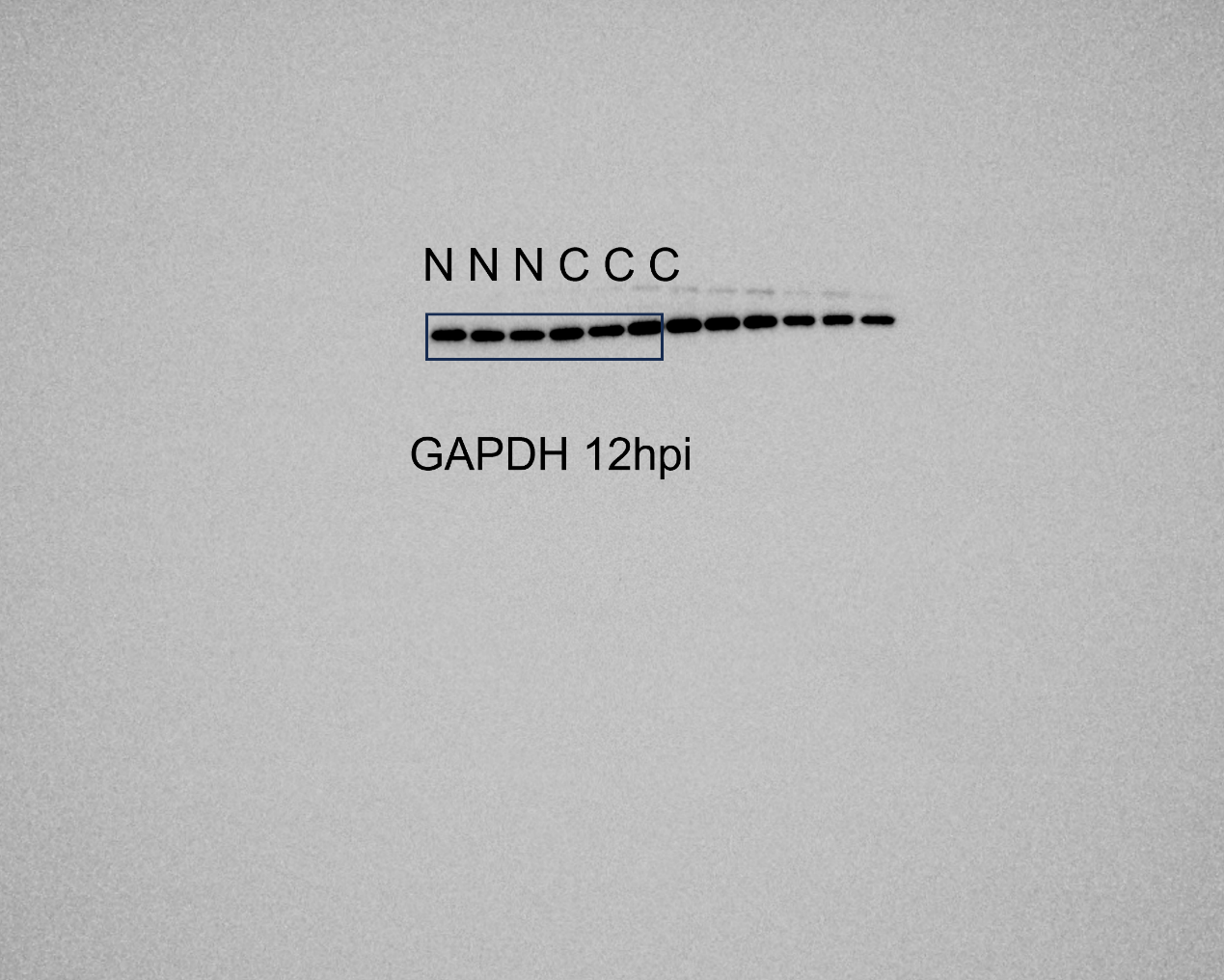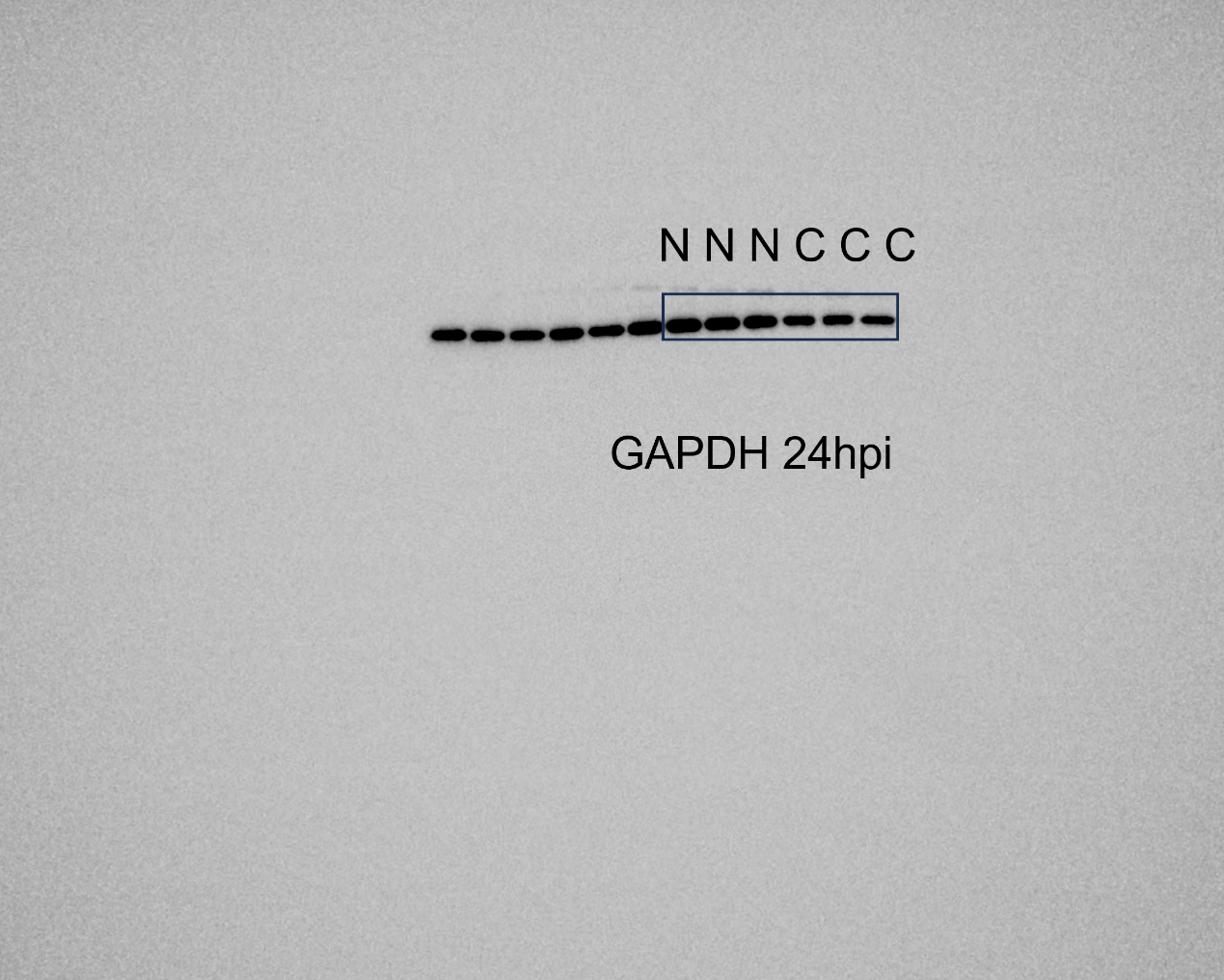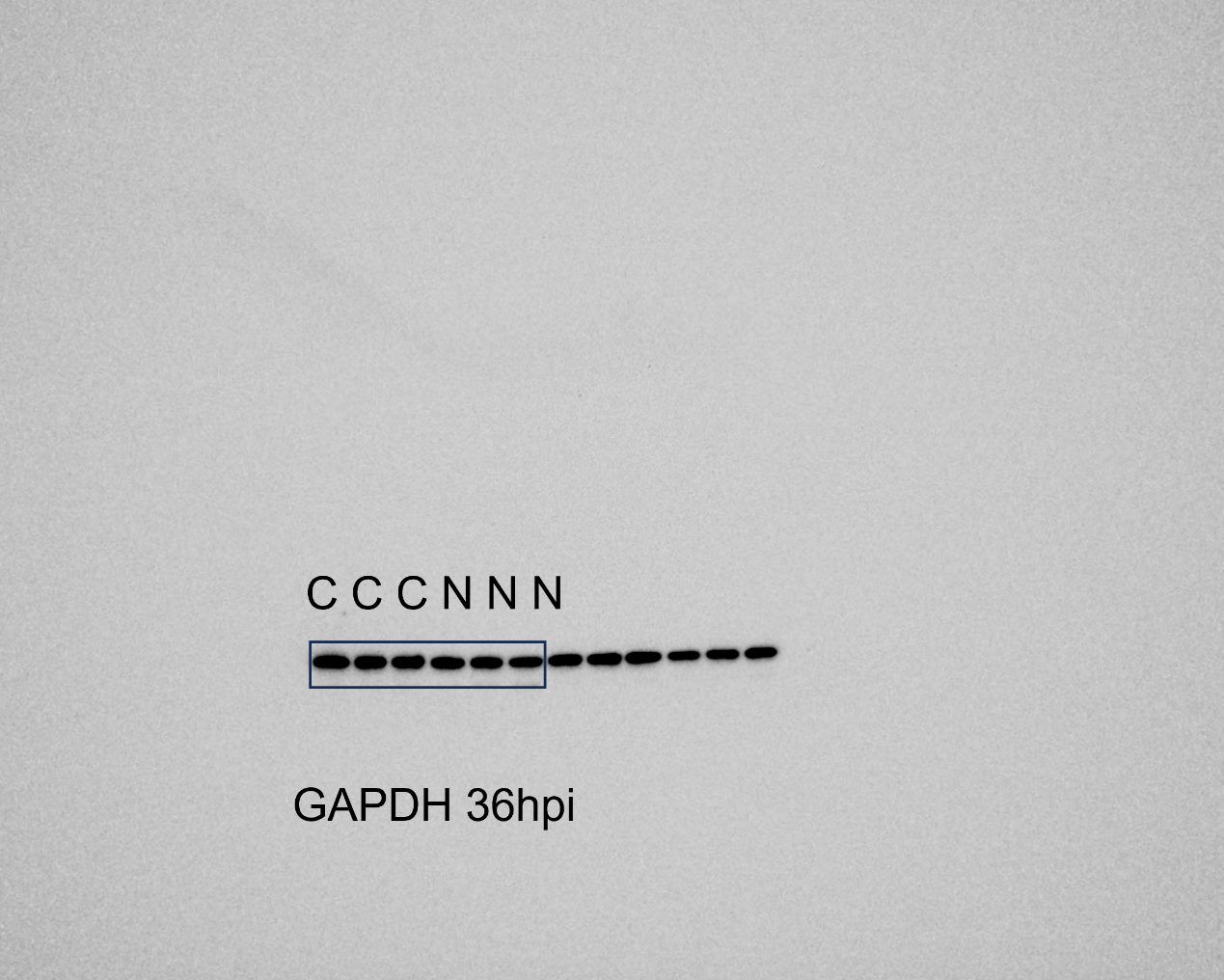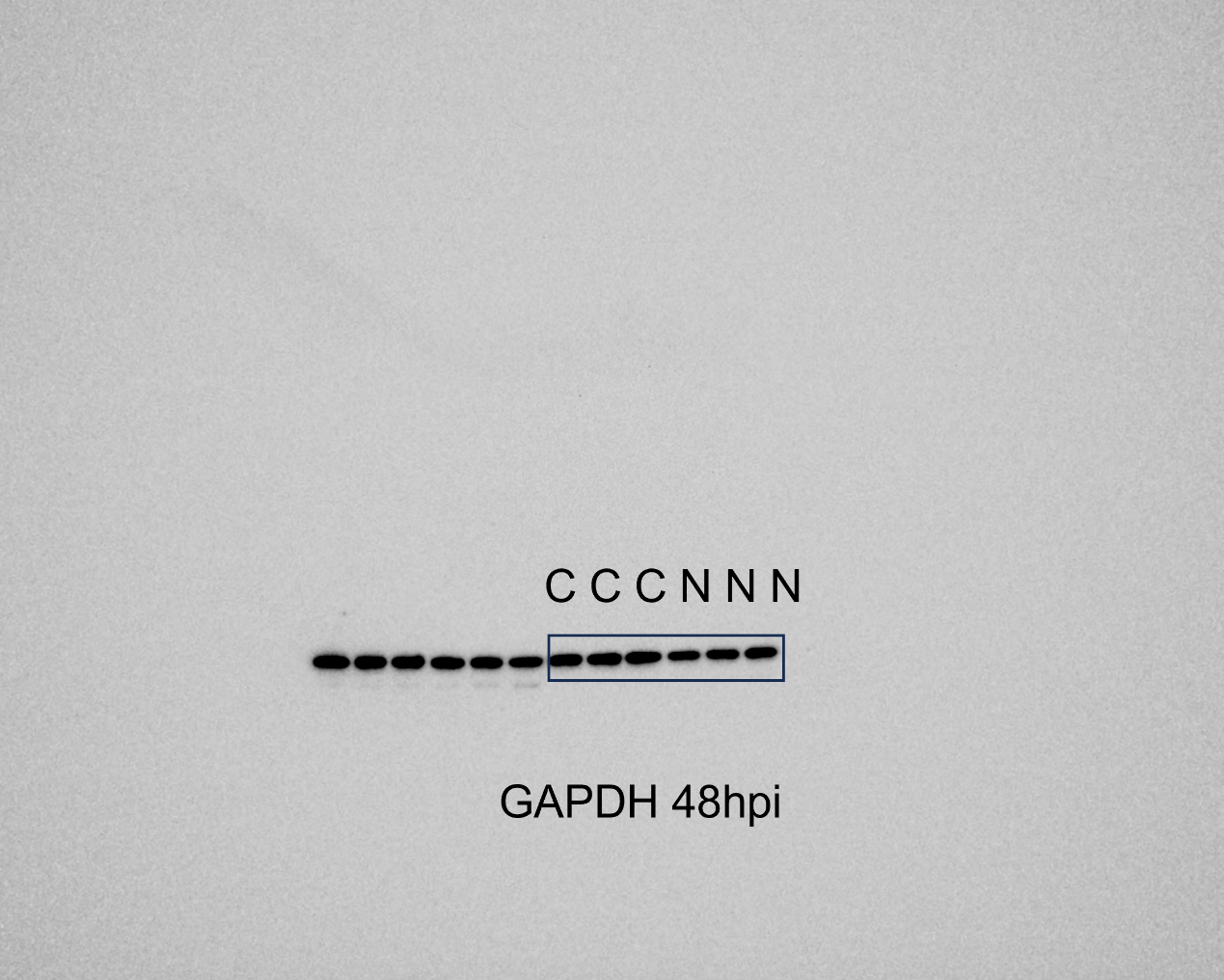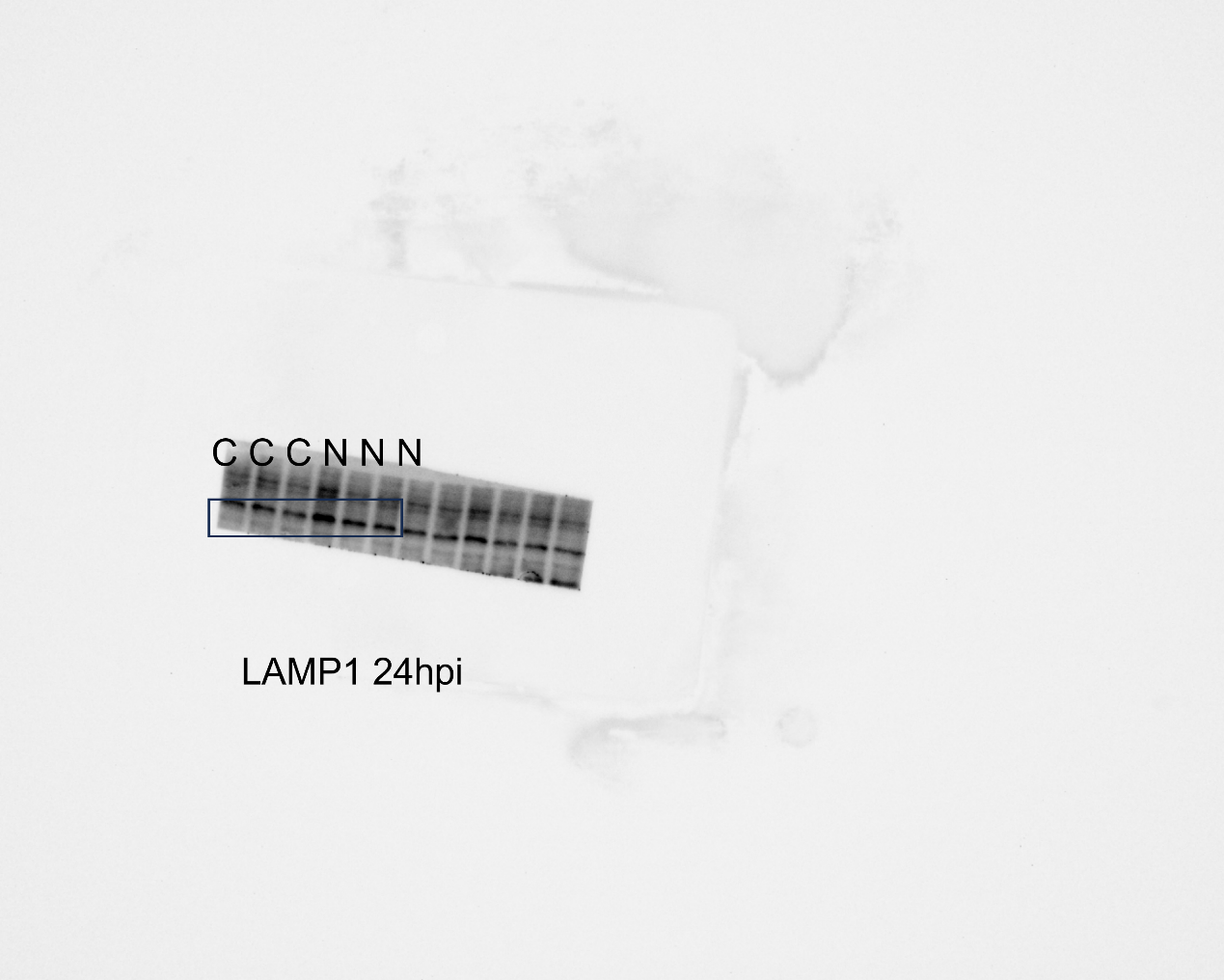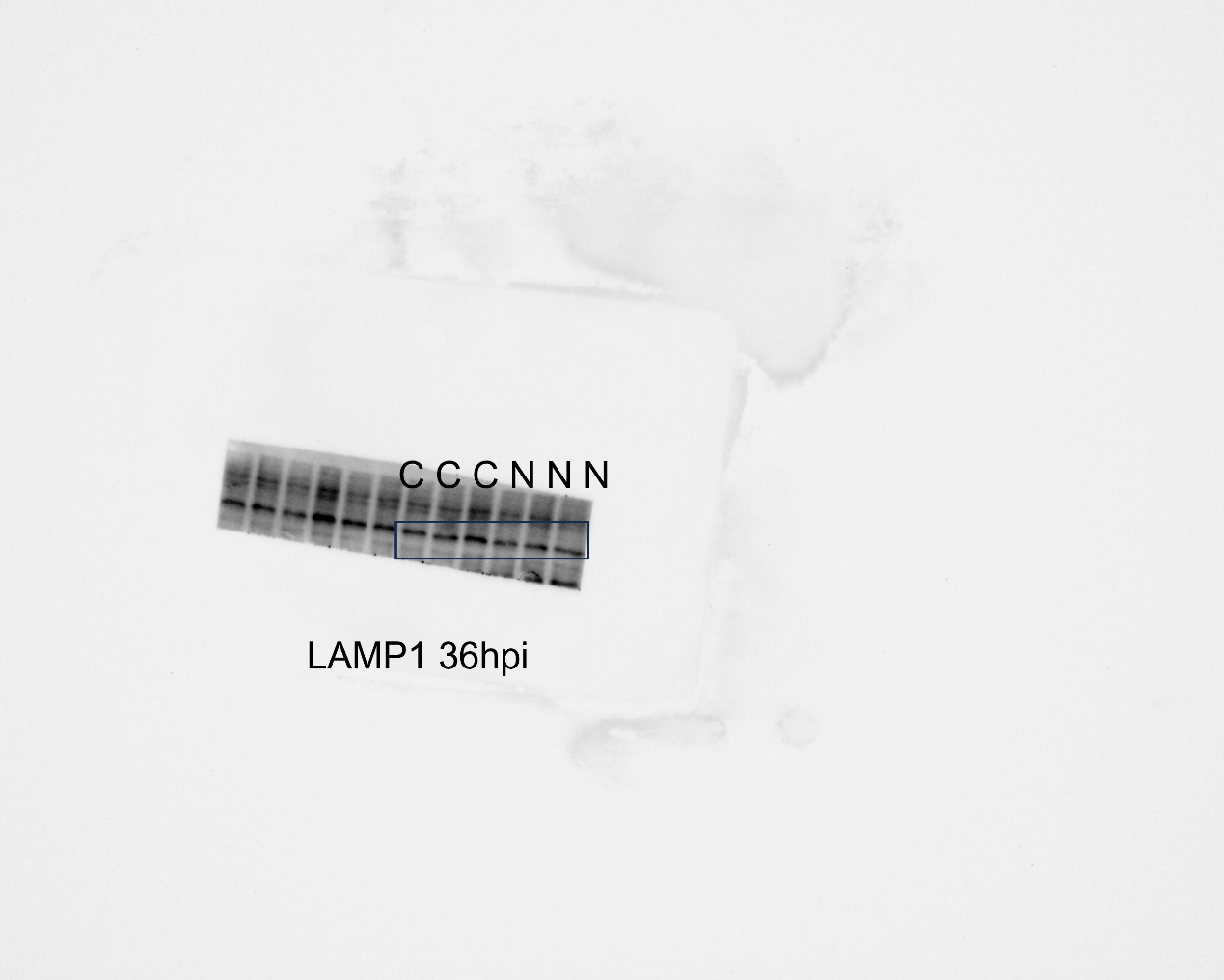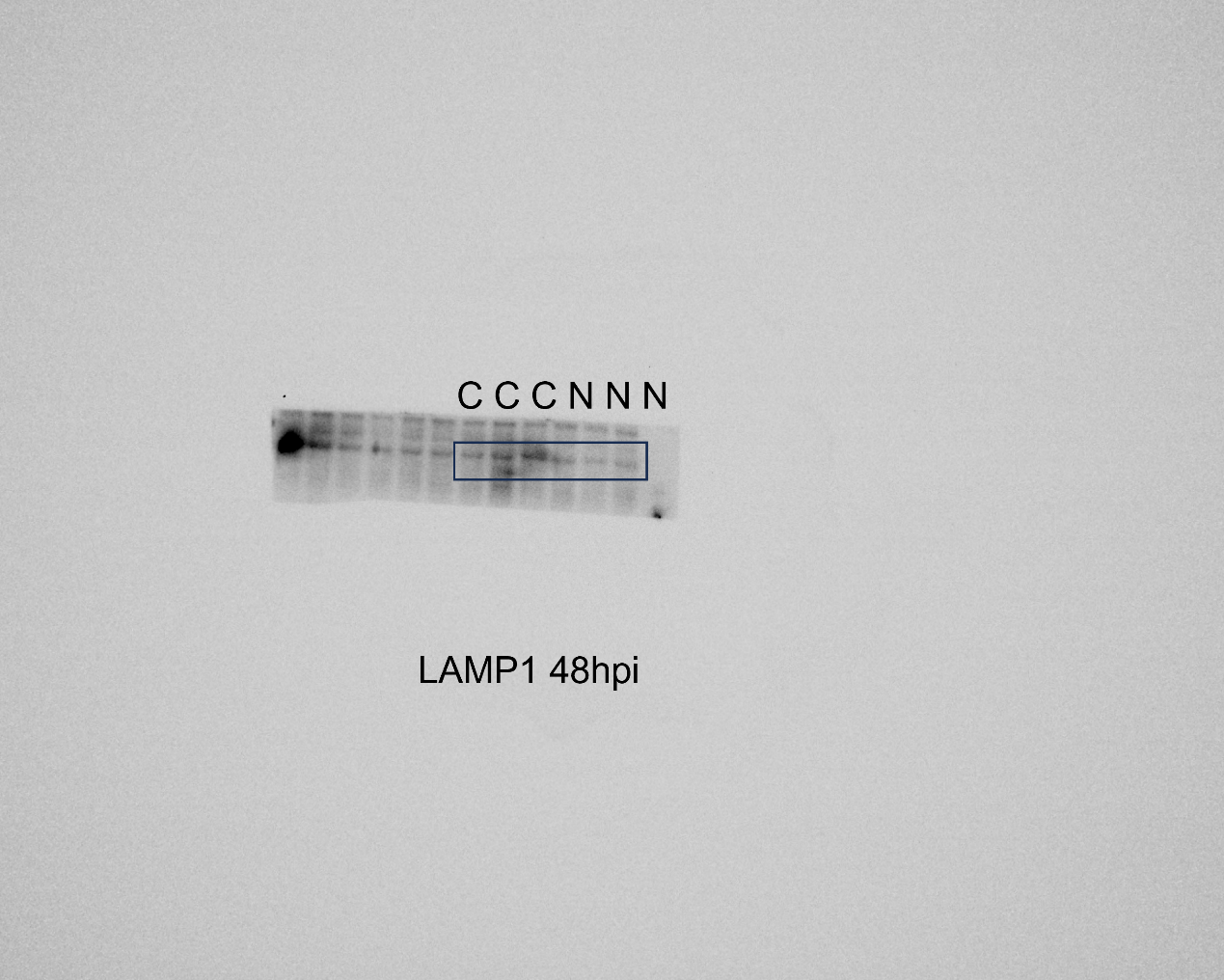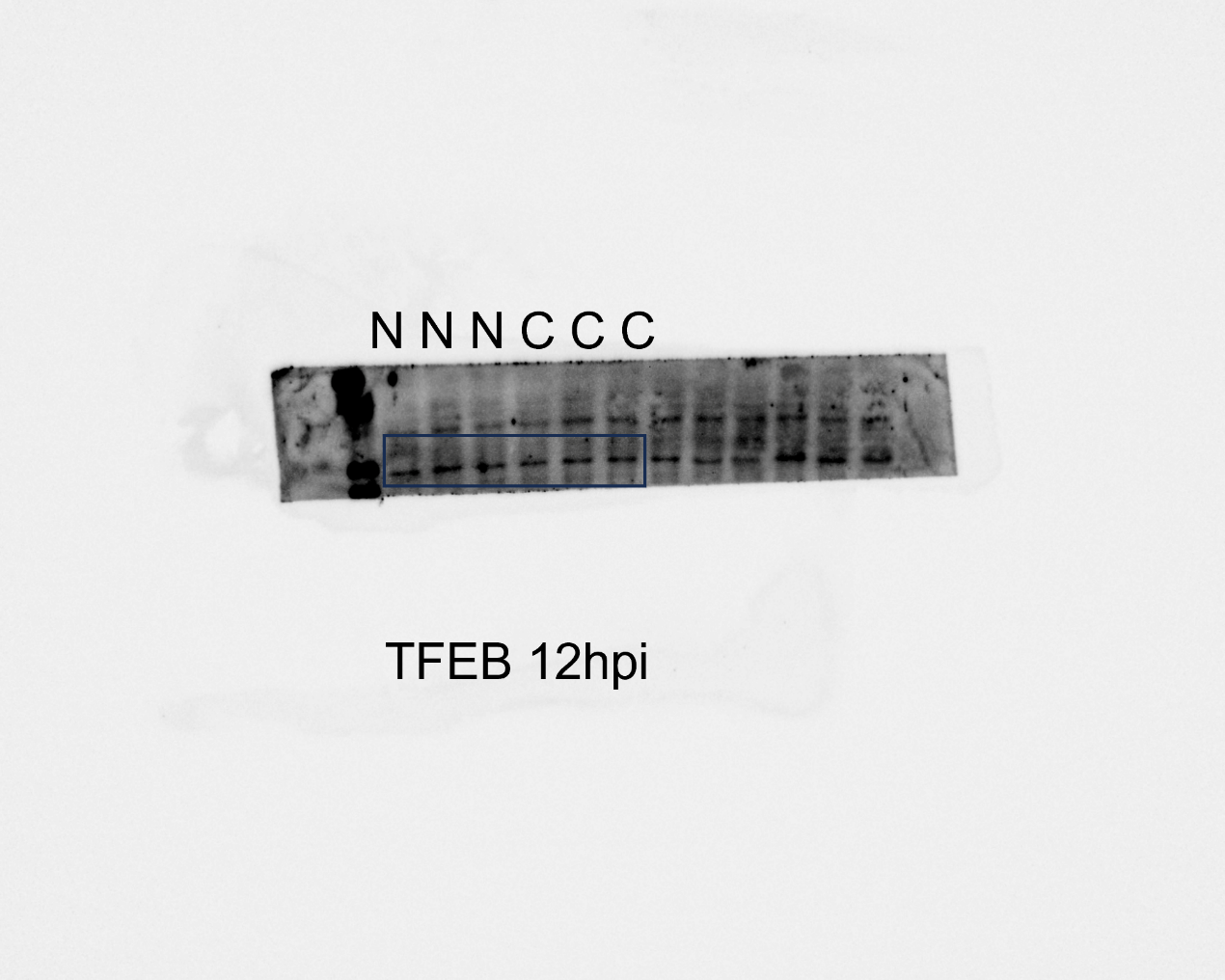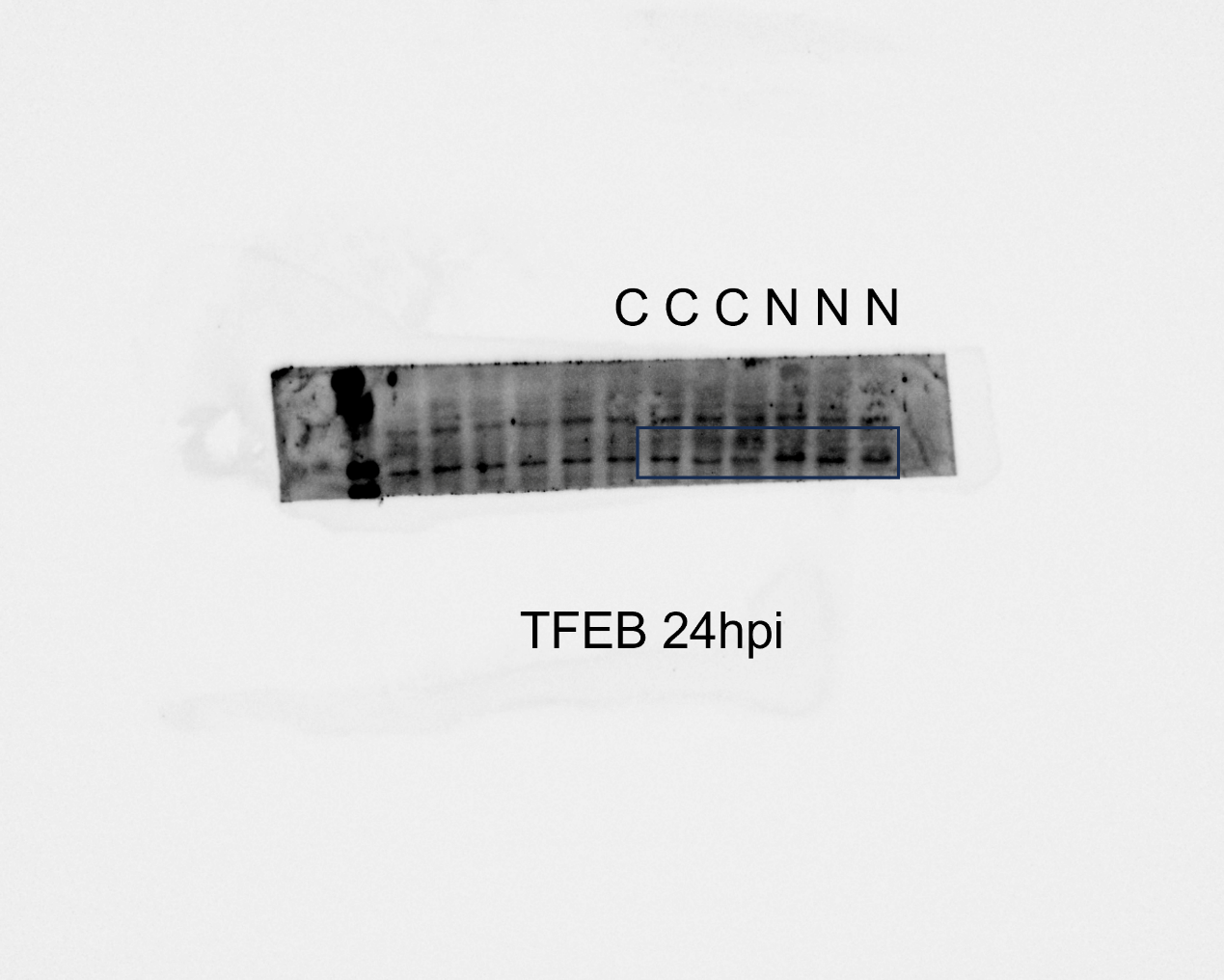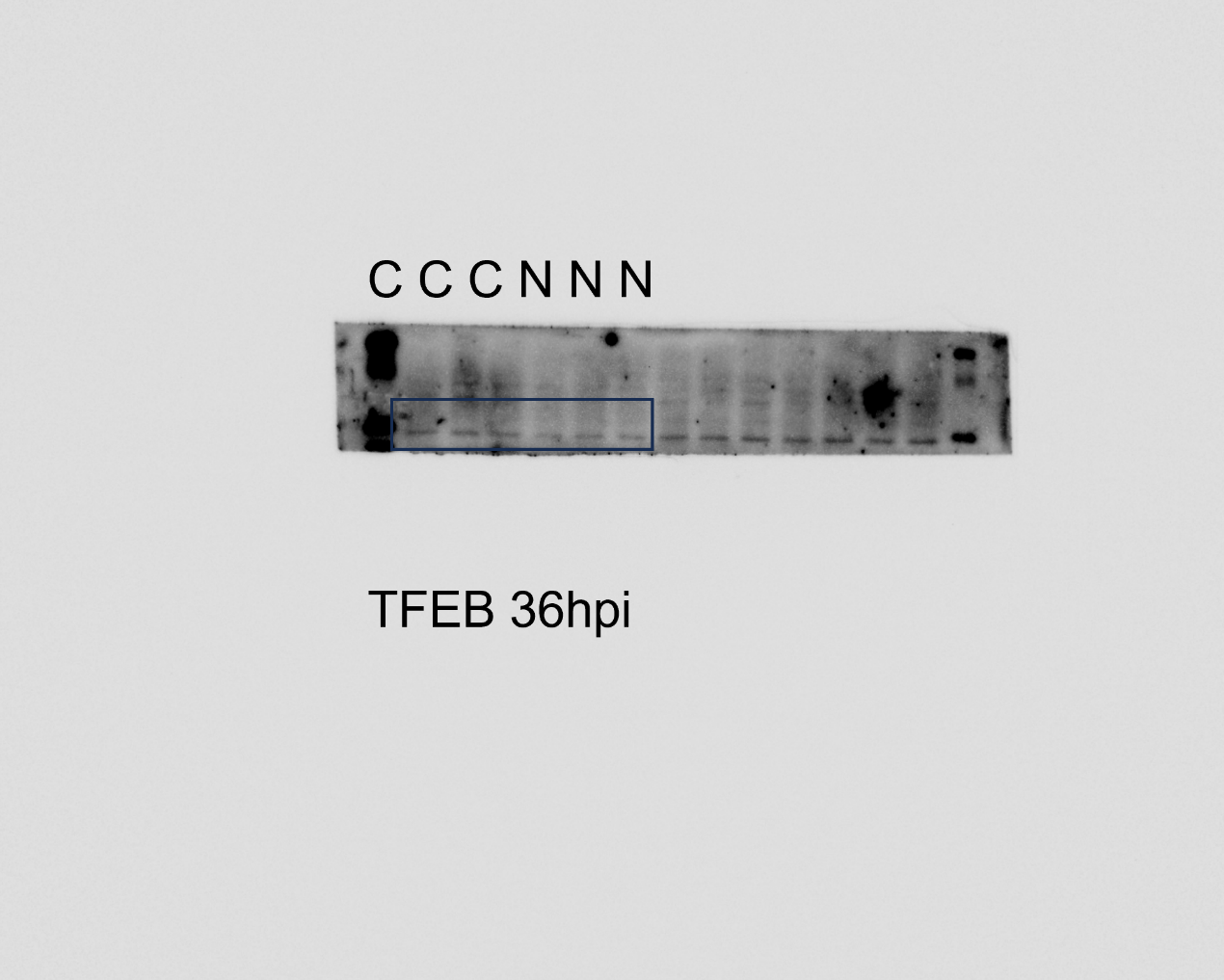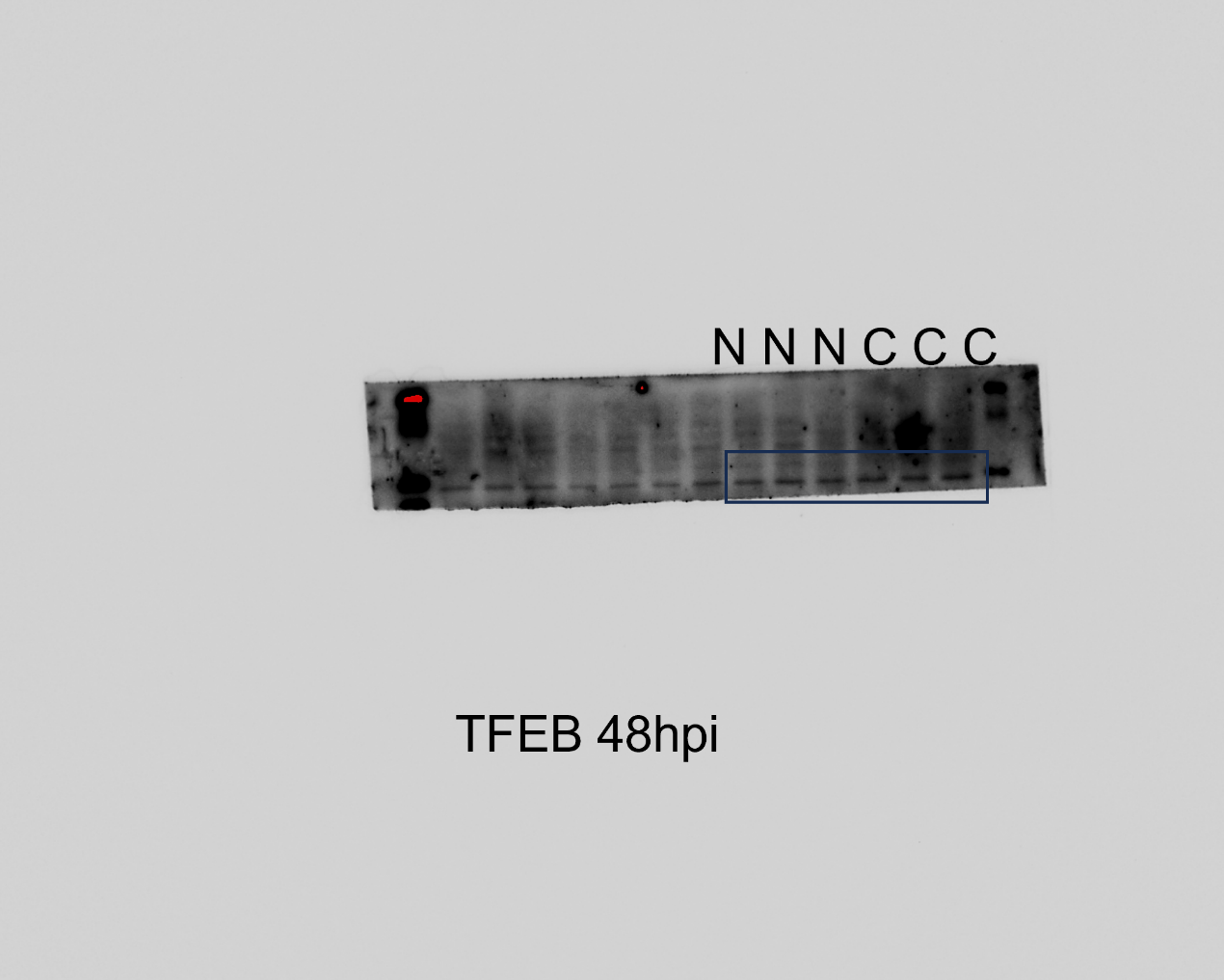** | | |
| **Figure.6 (D)** | | |
| **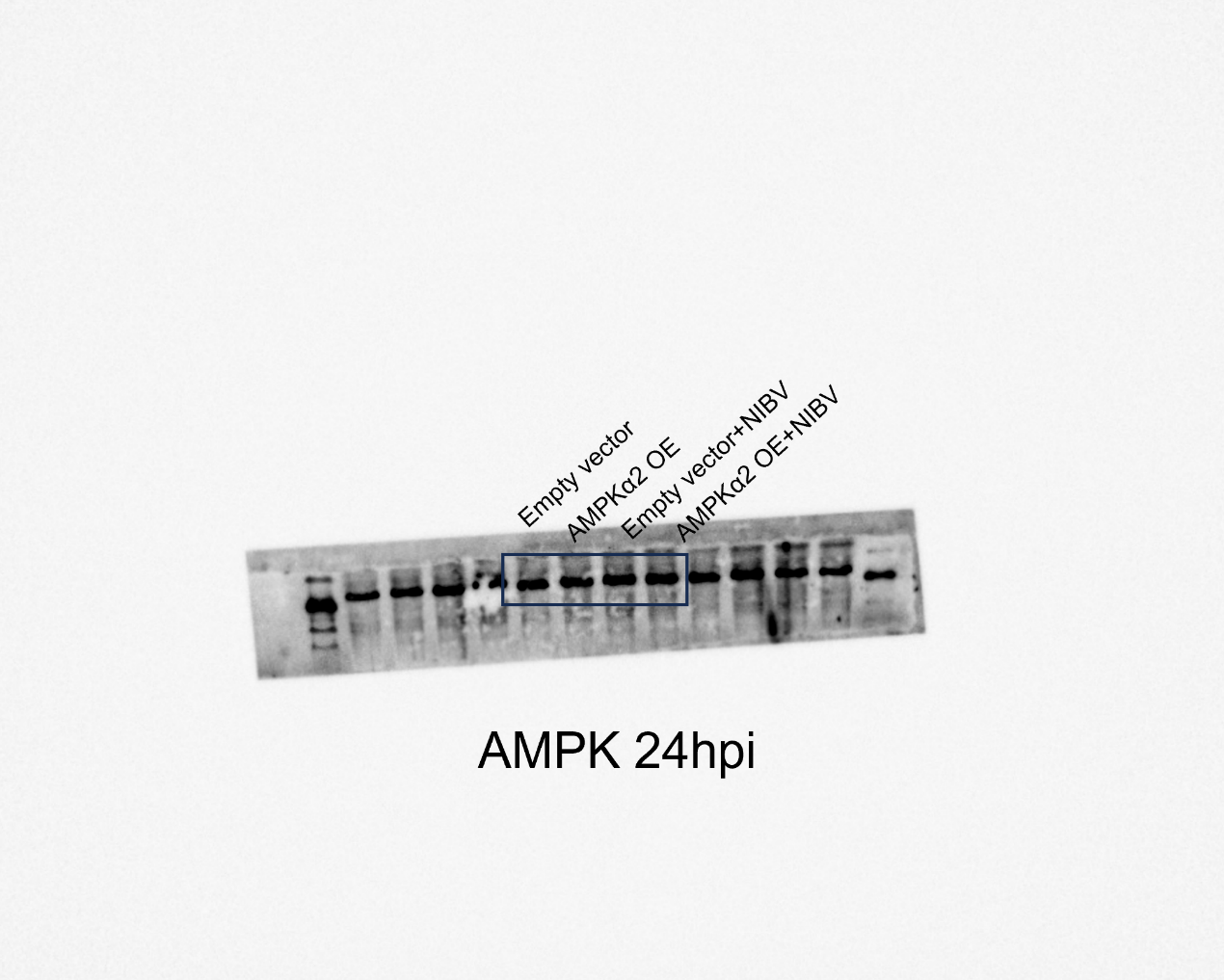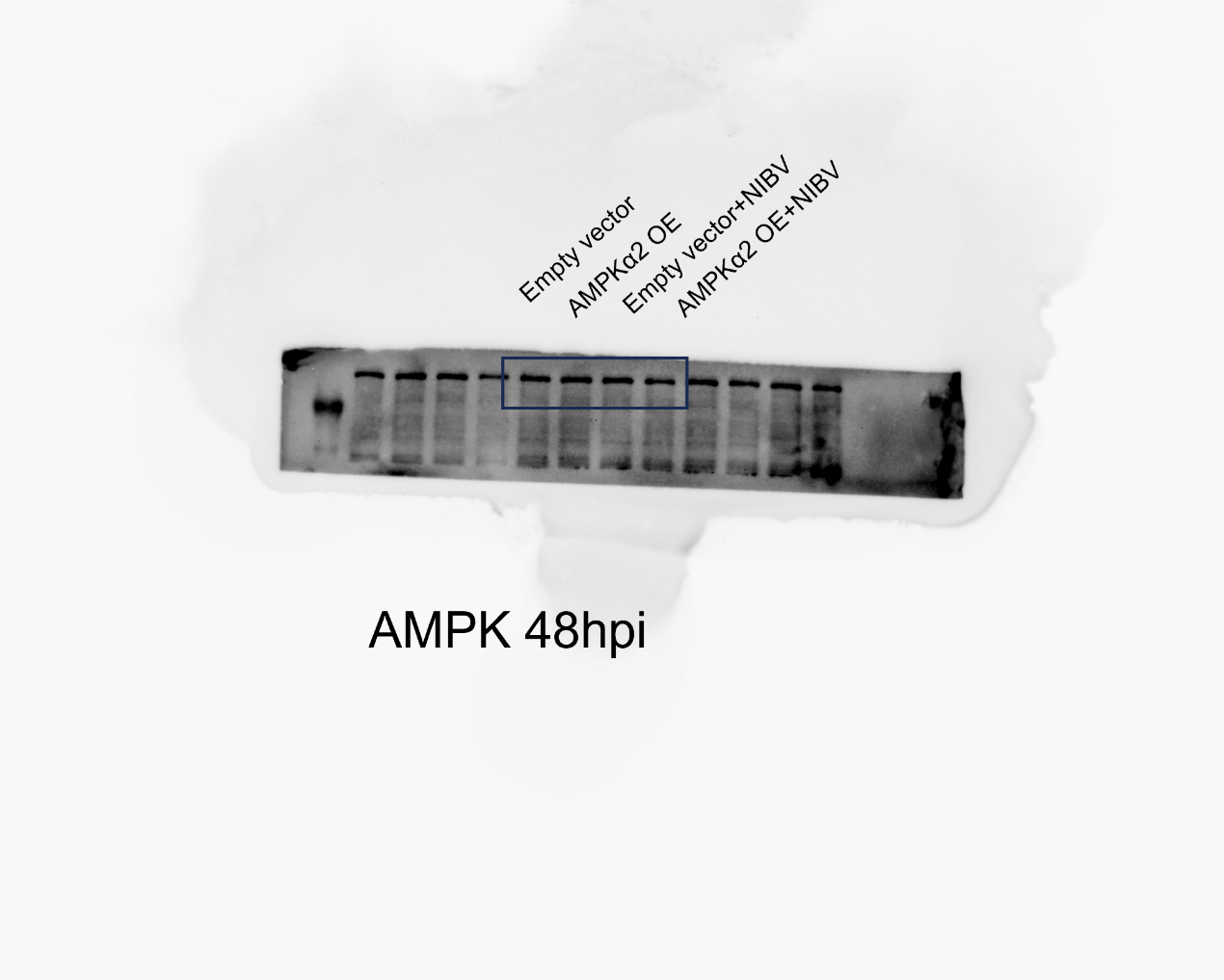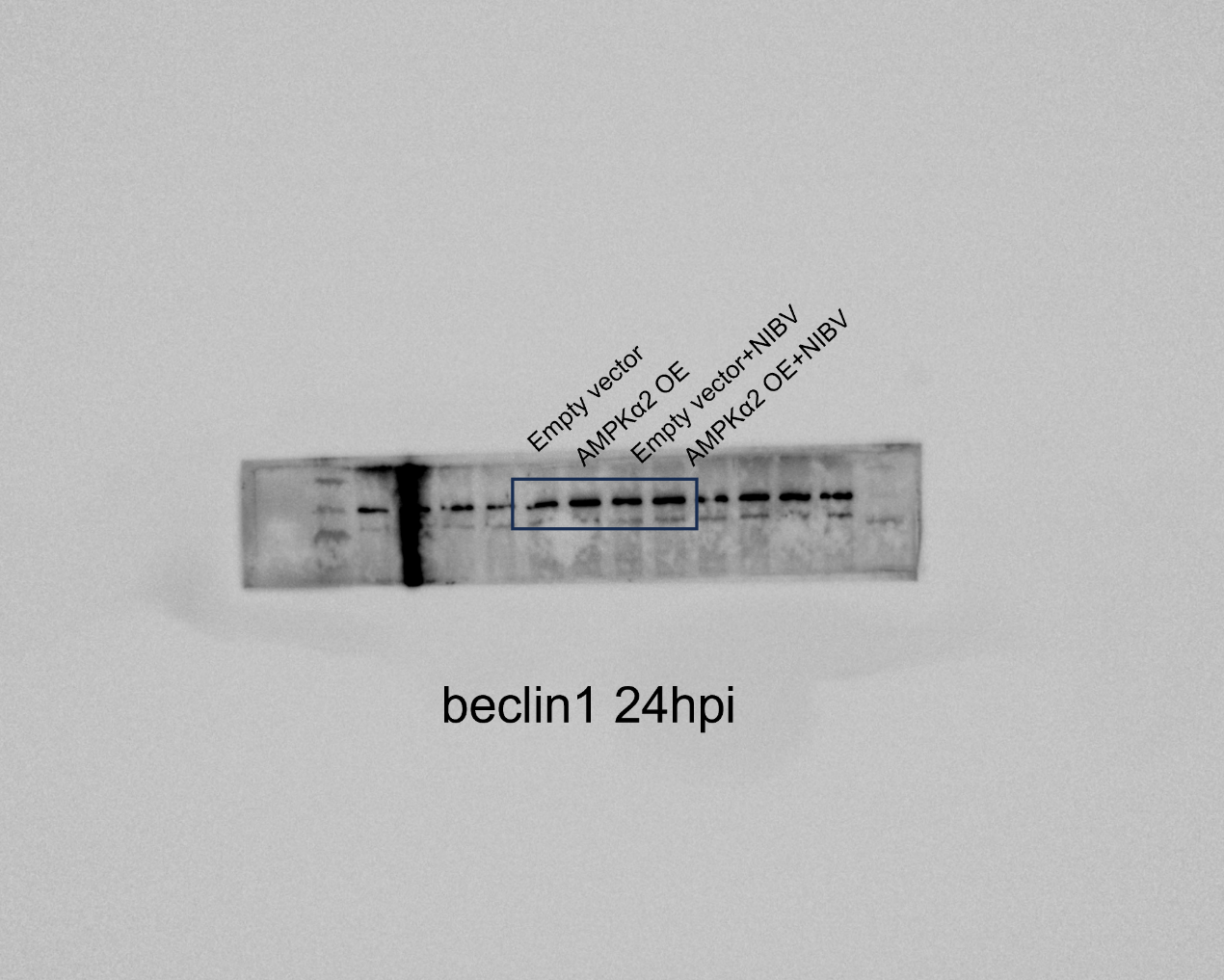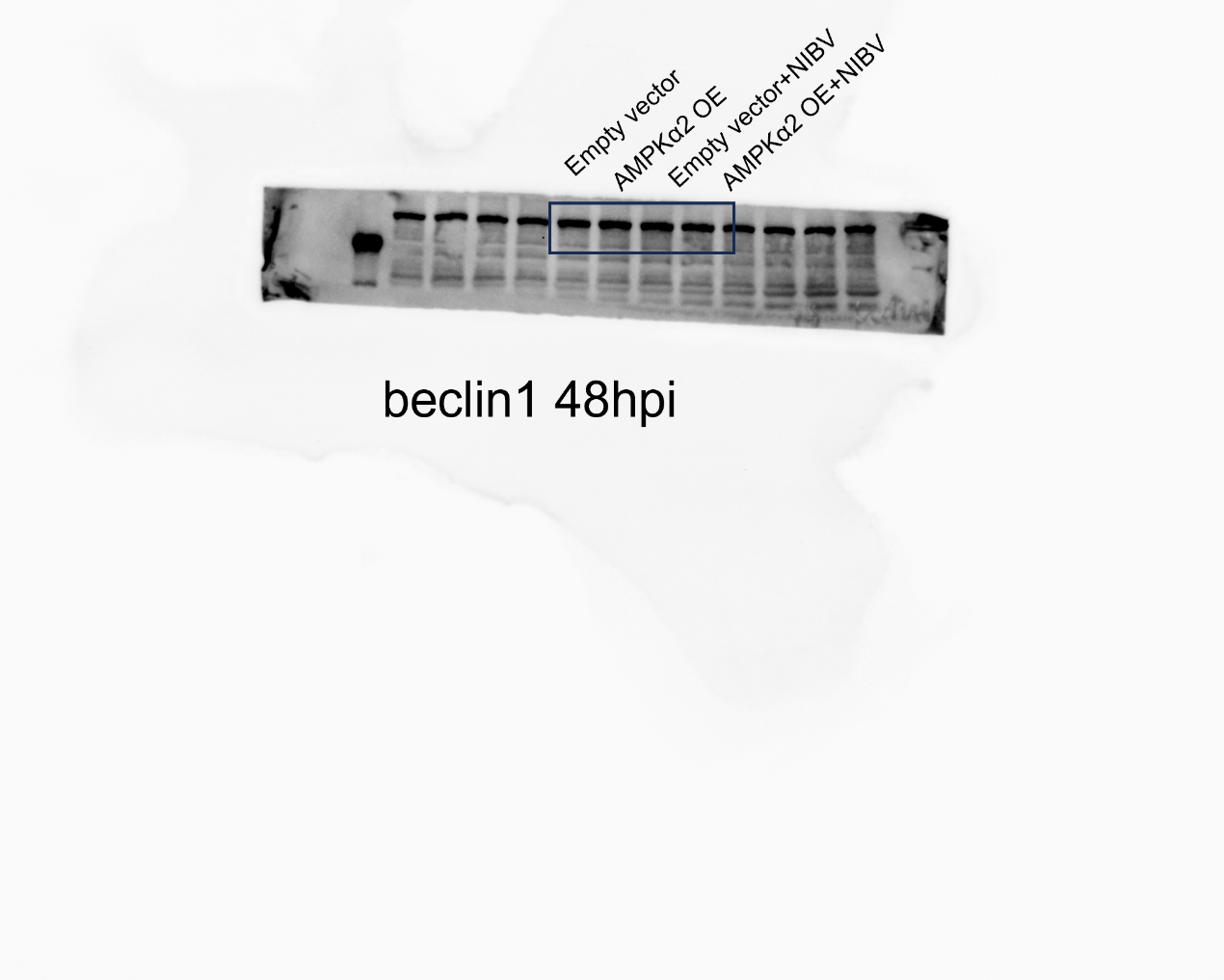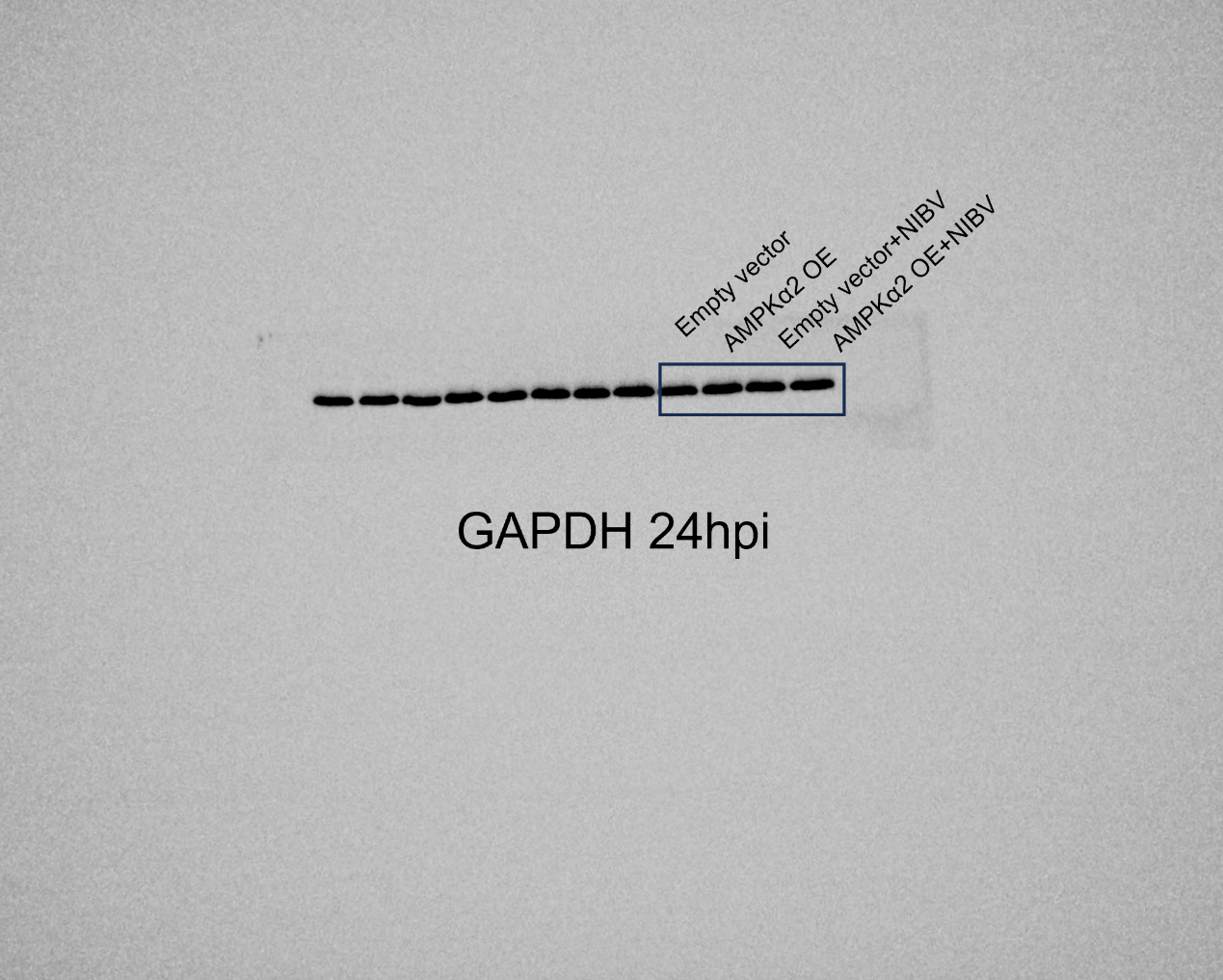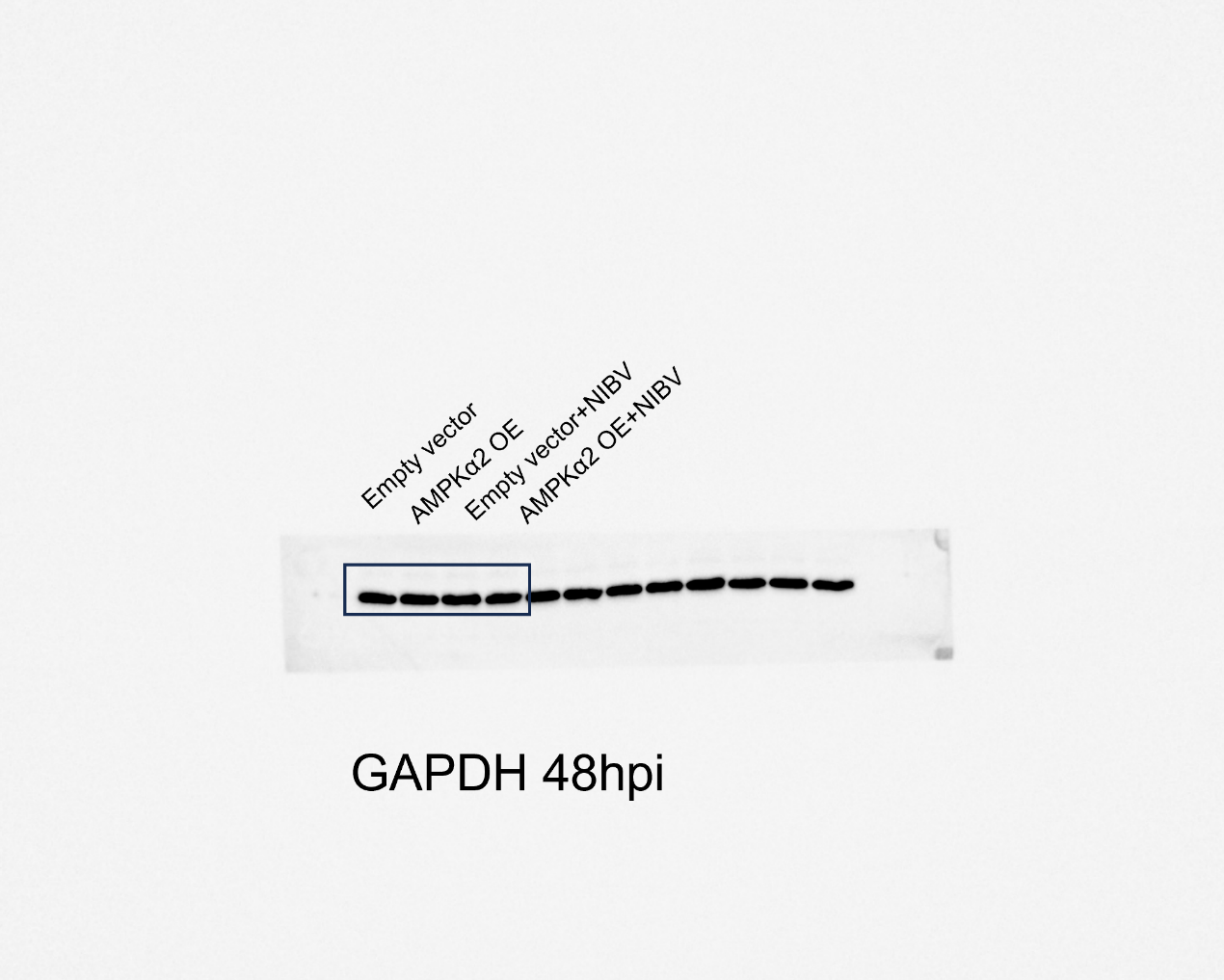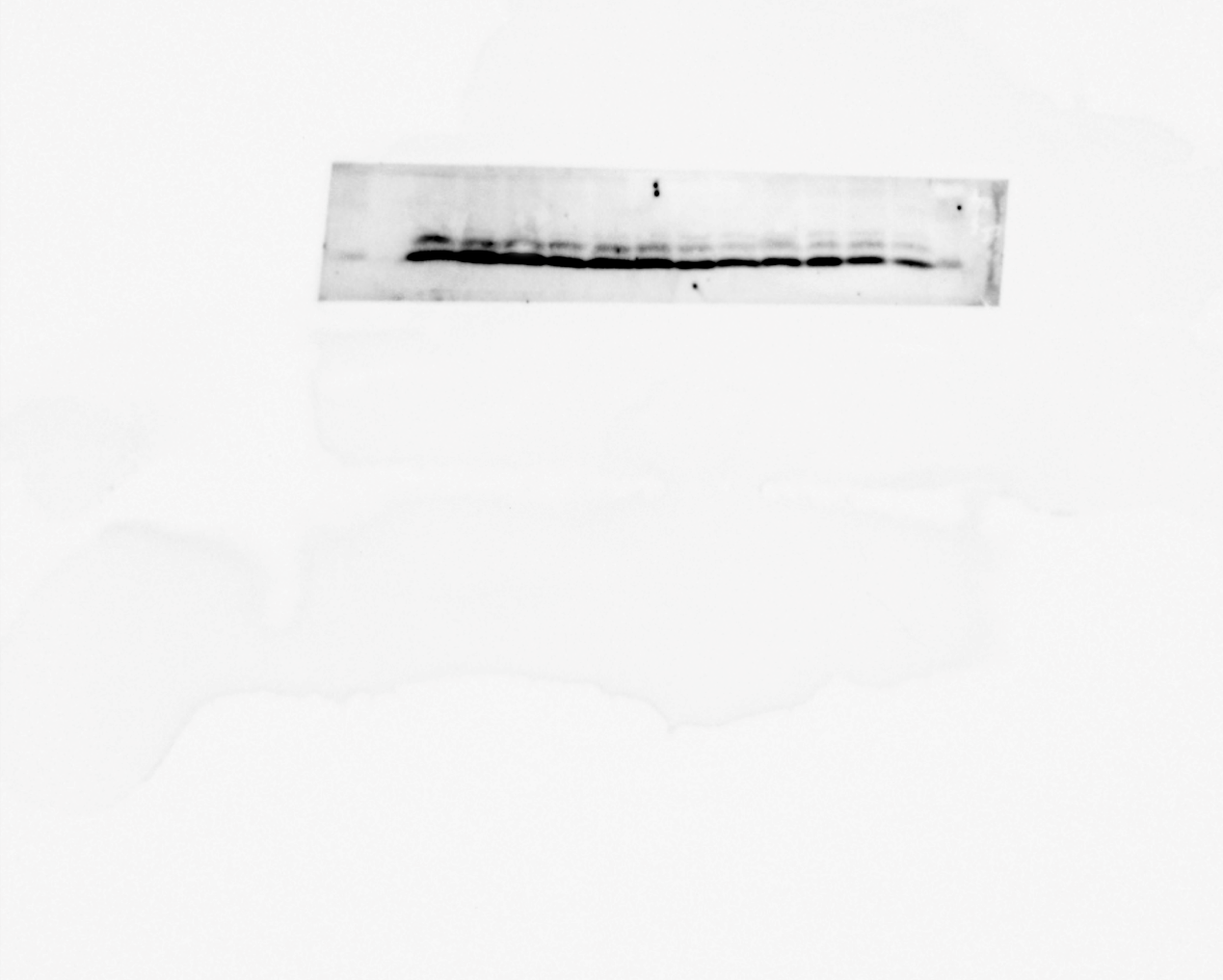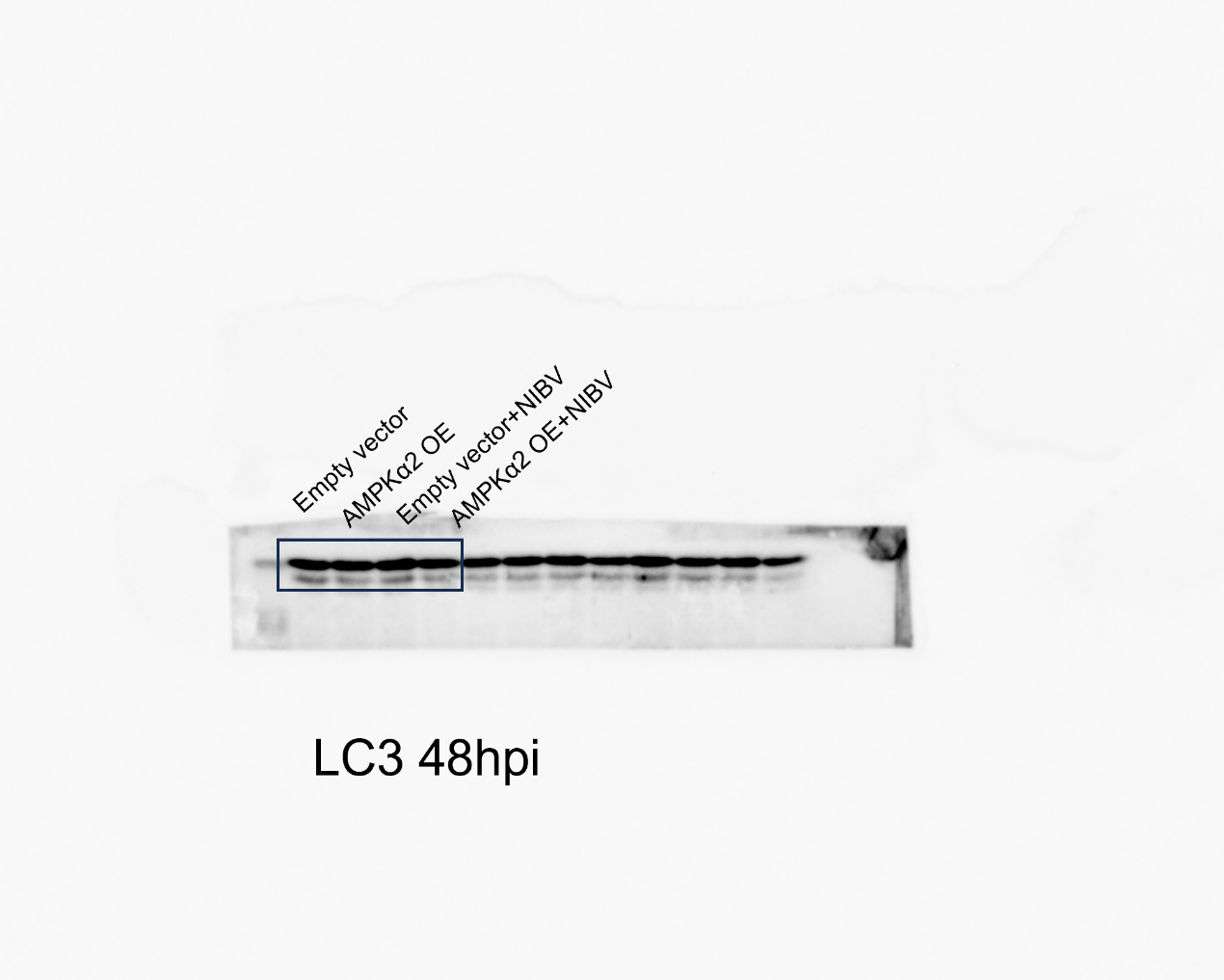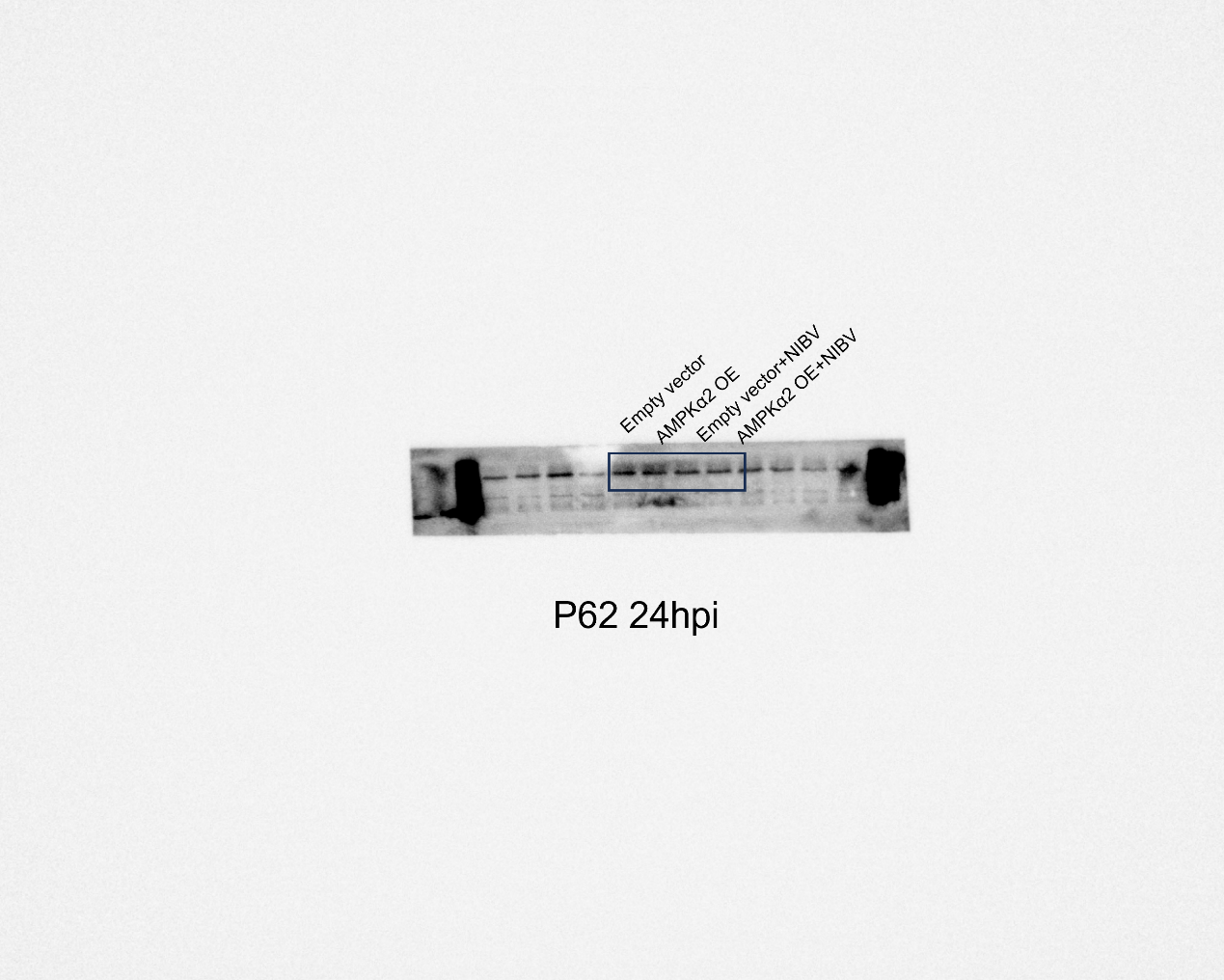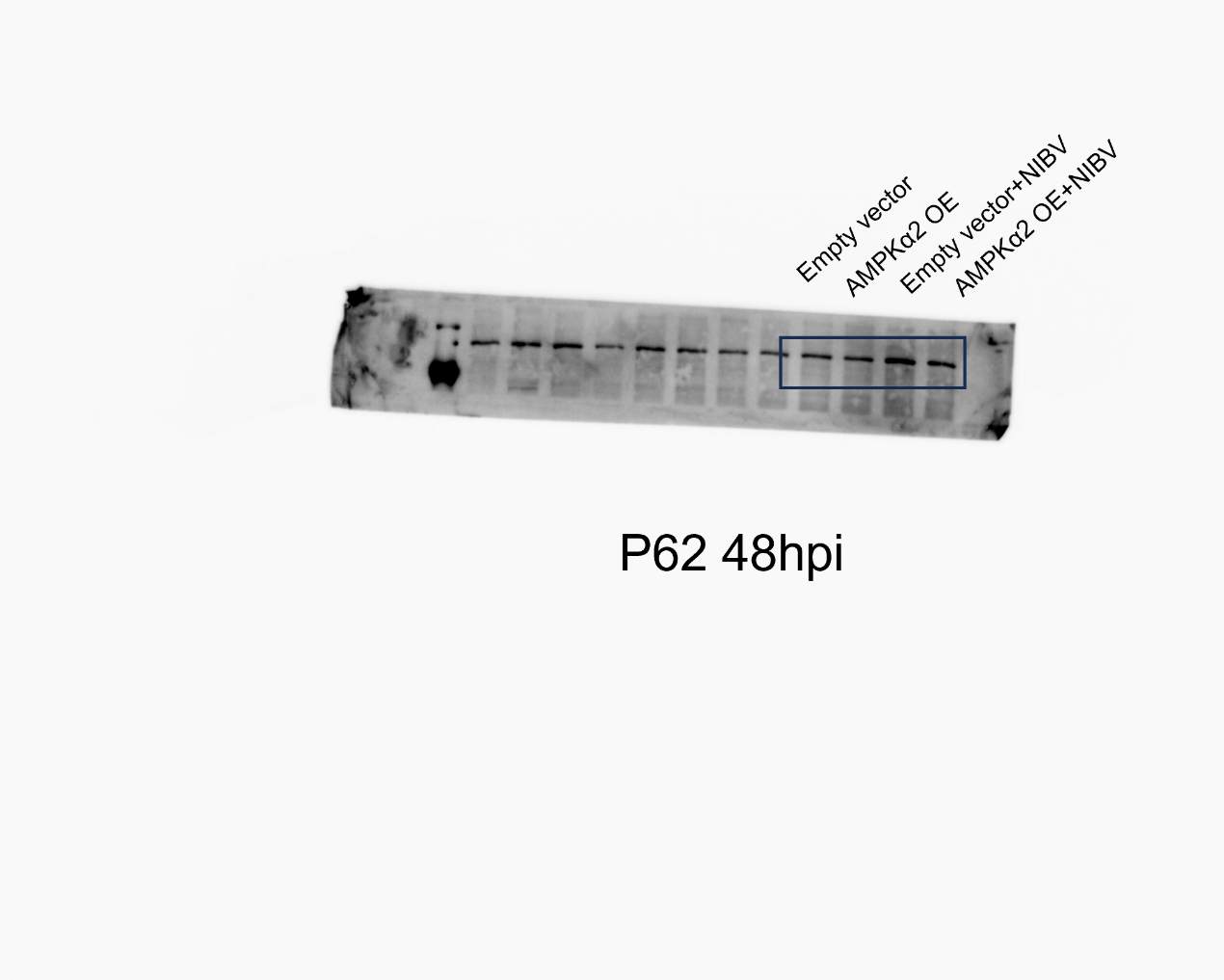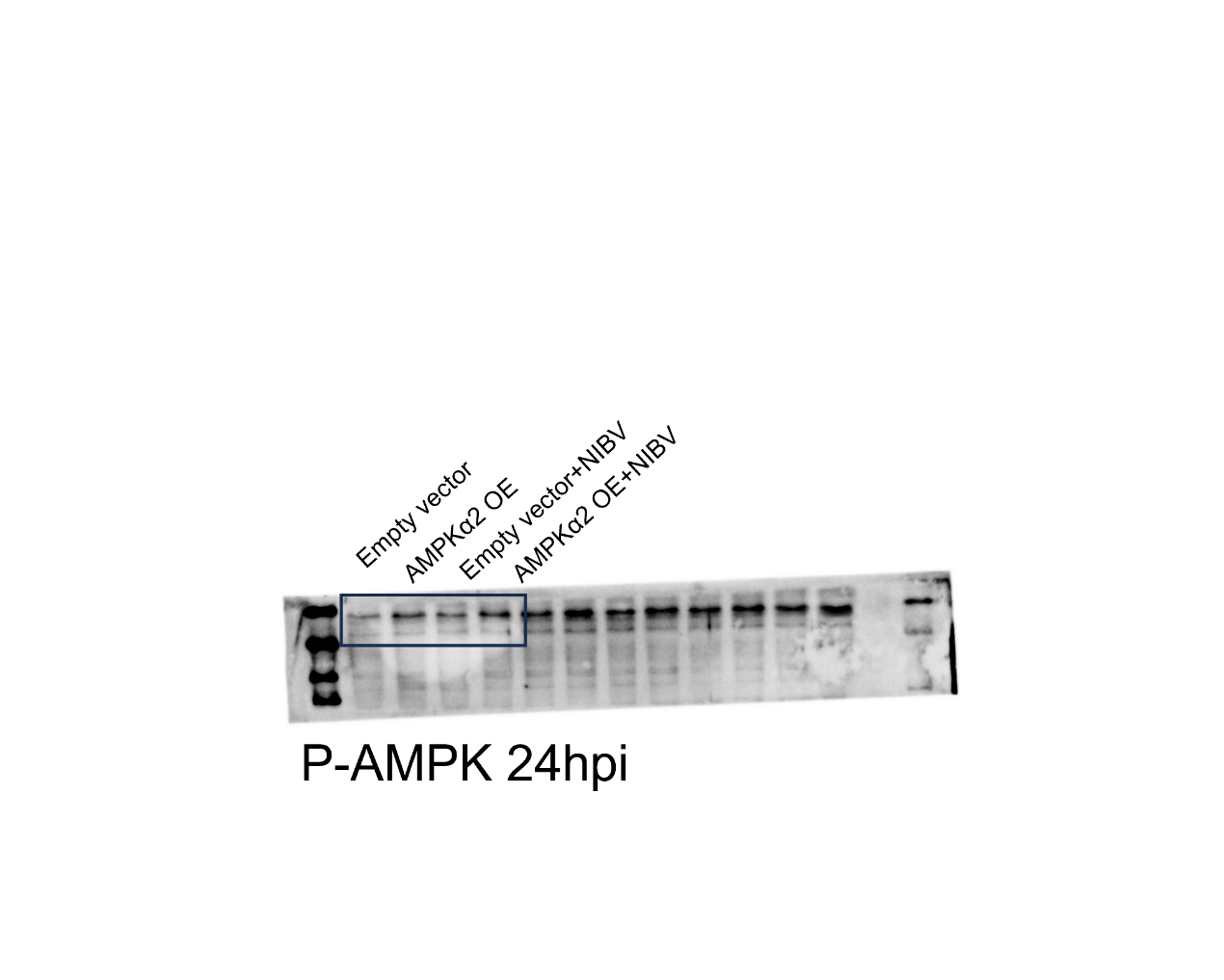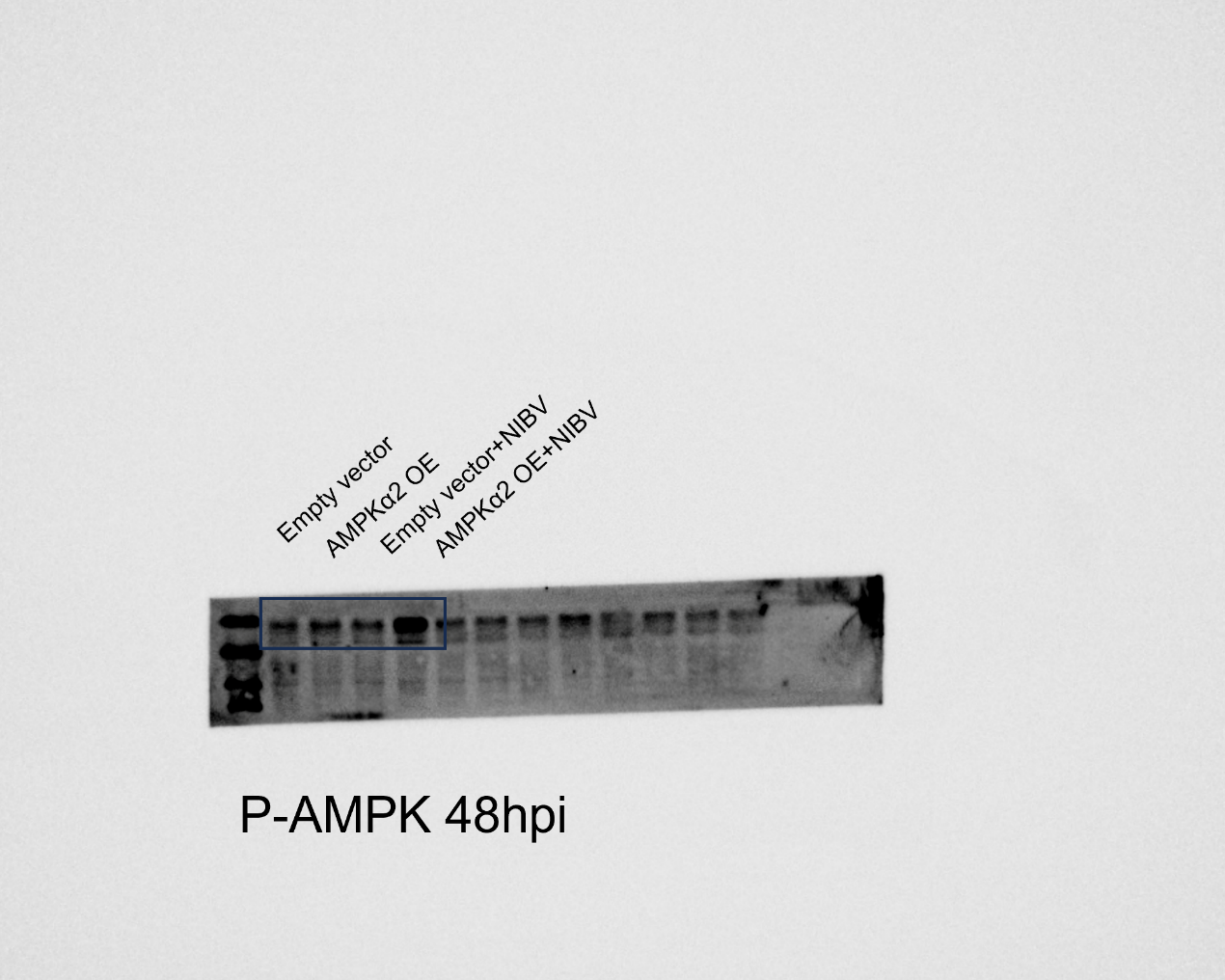** | | |
| **Figure.7 (B and C)** | | |
| **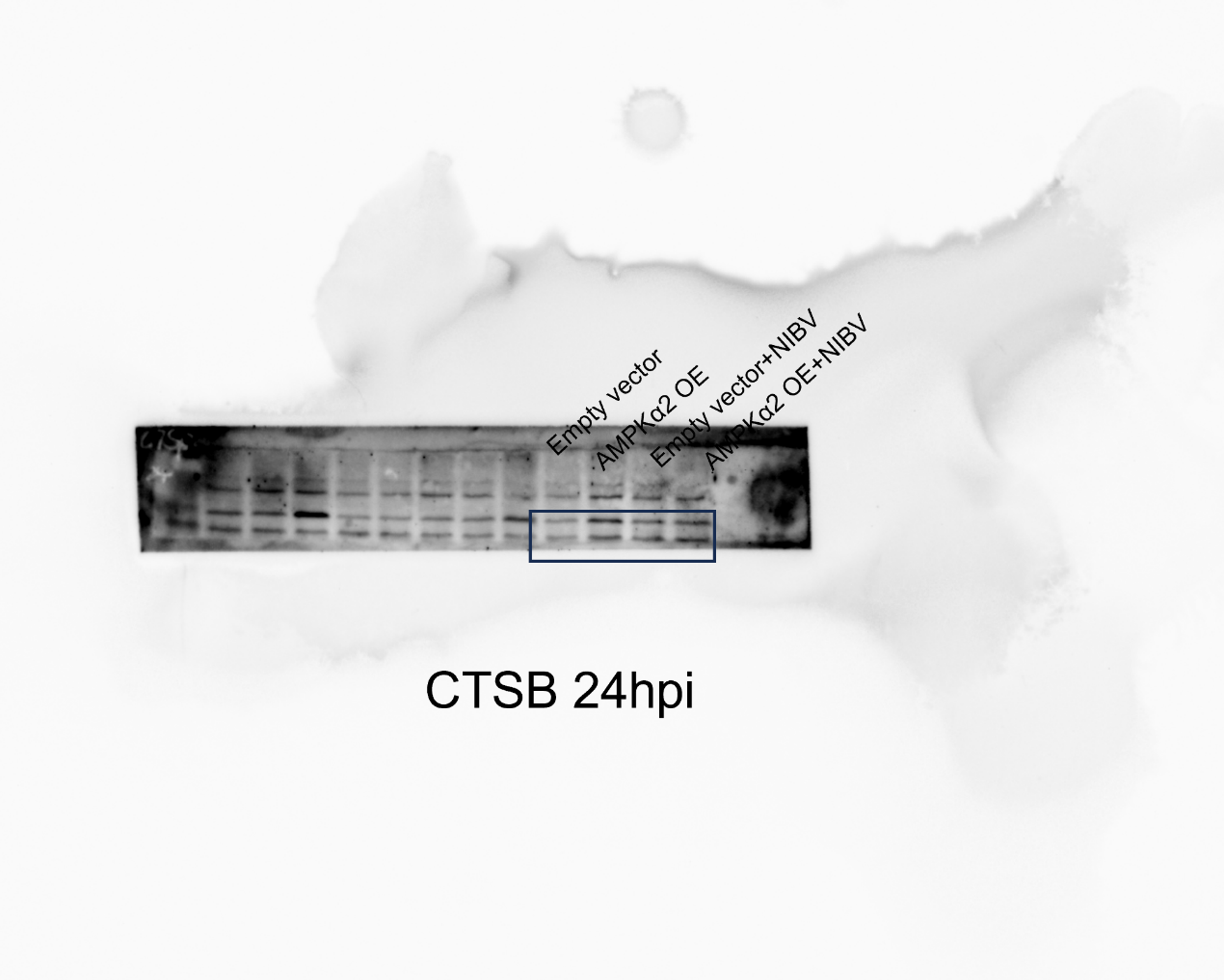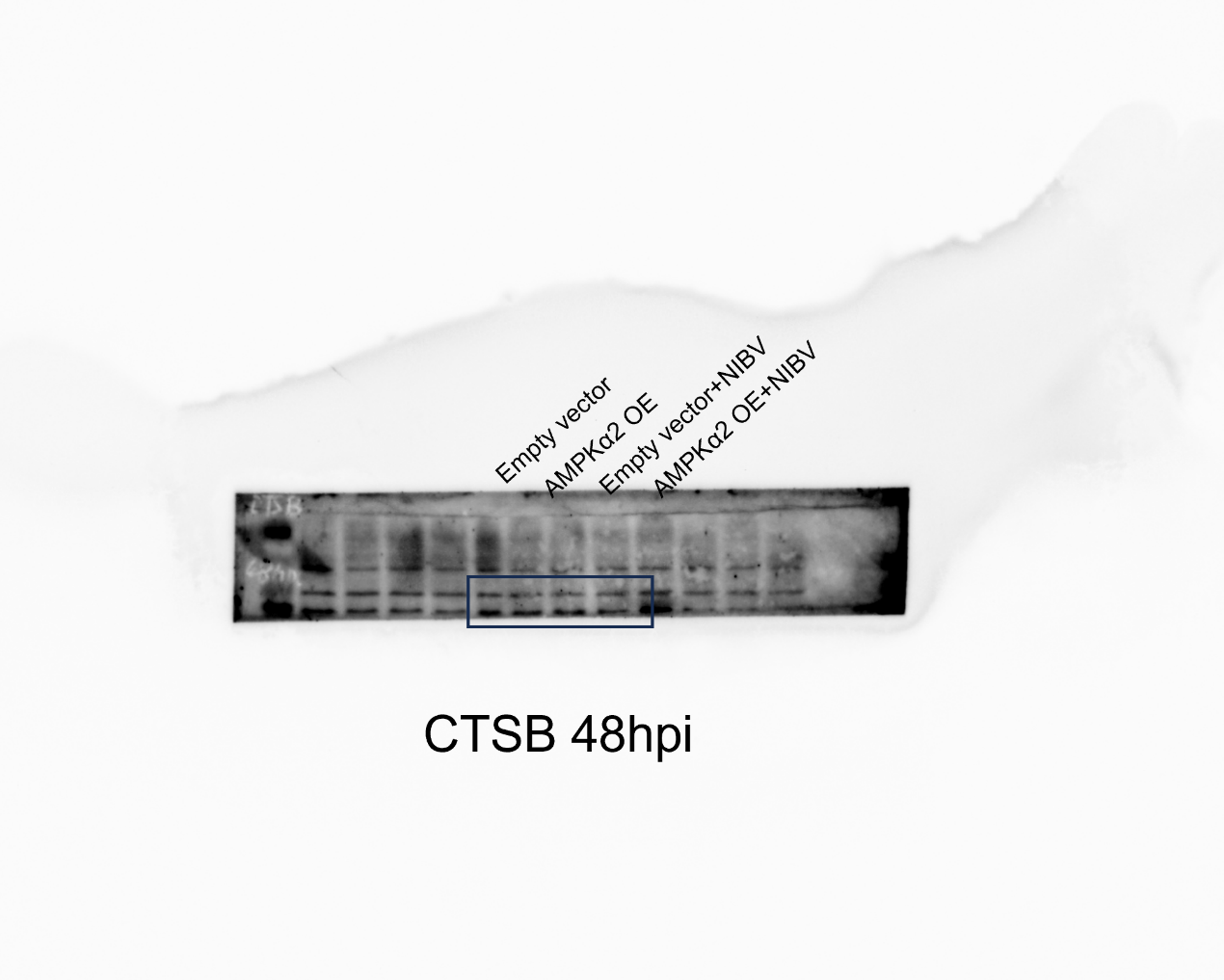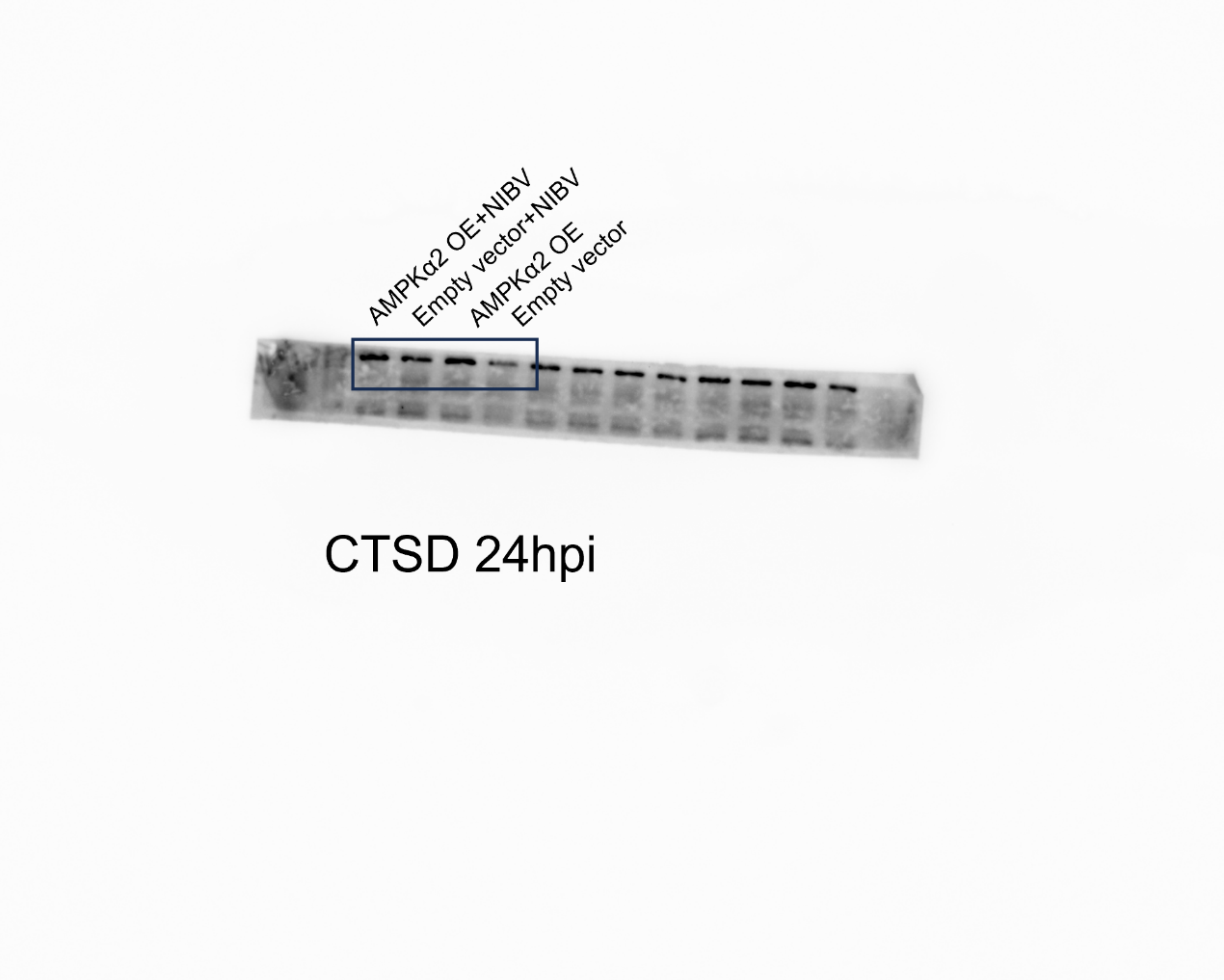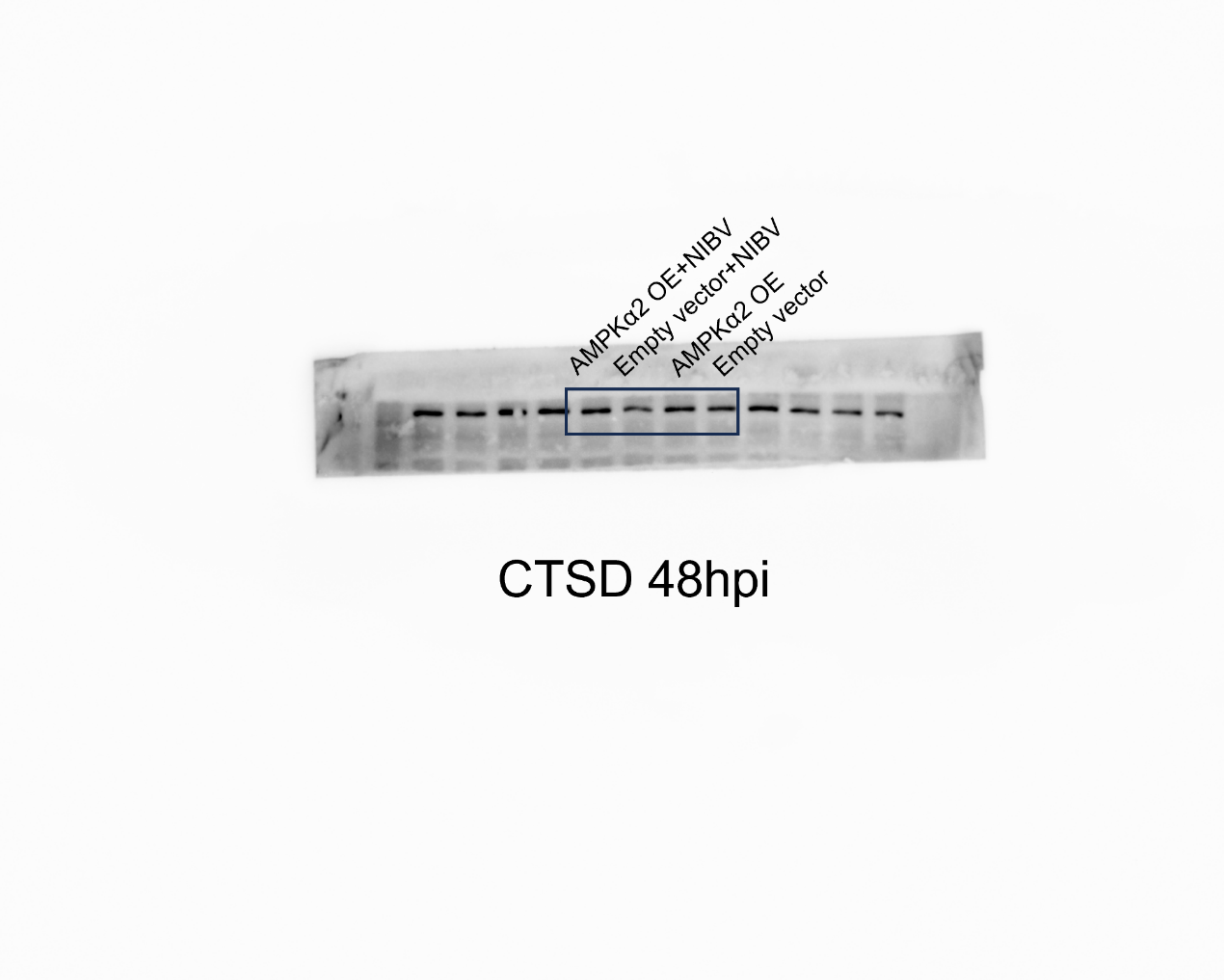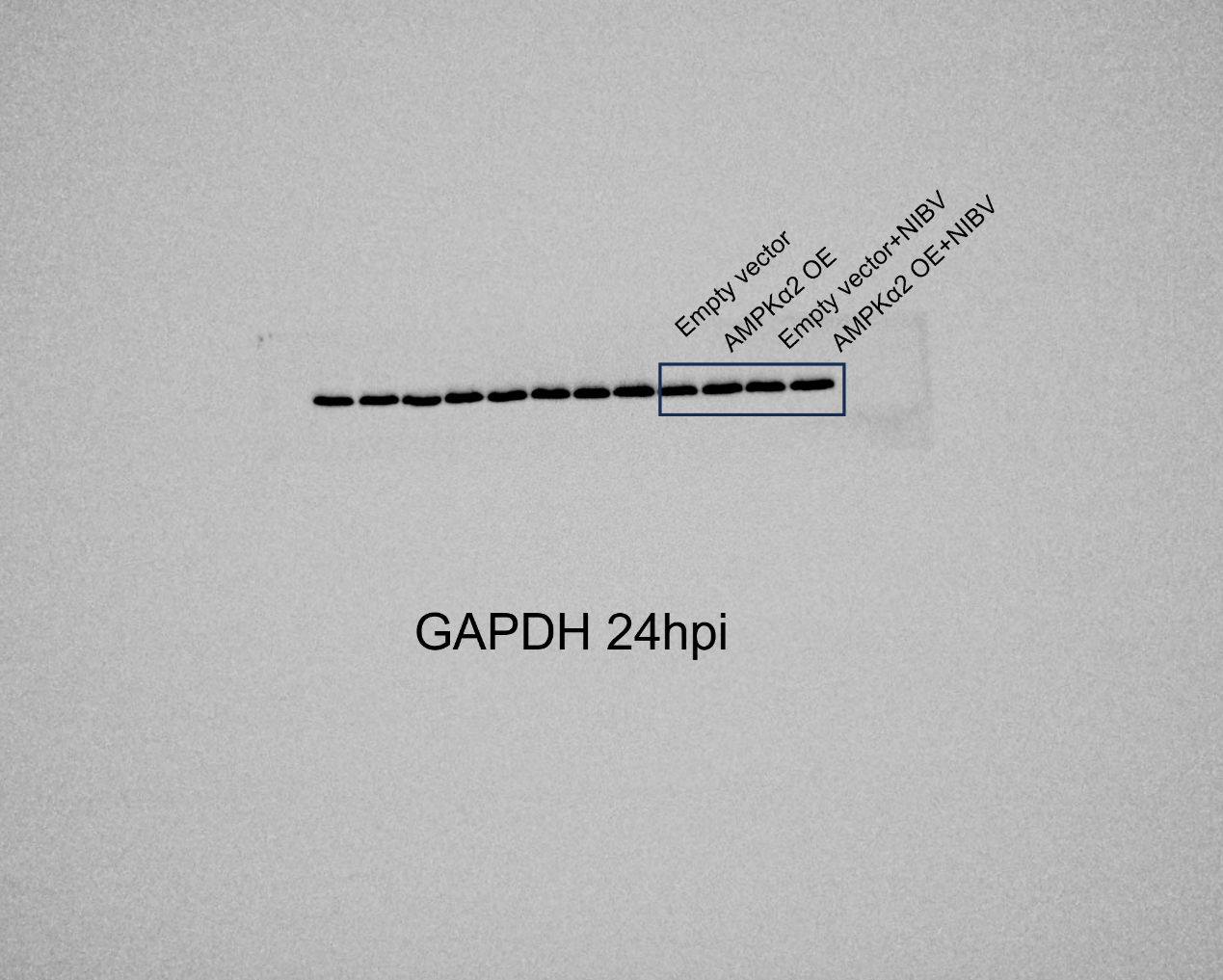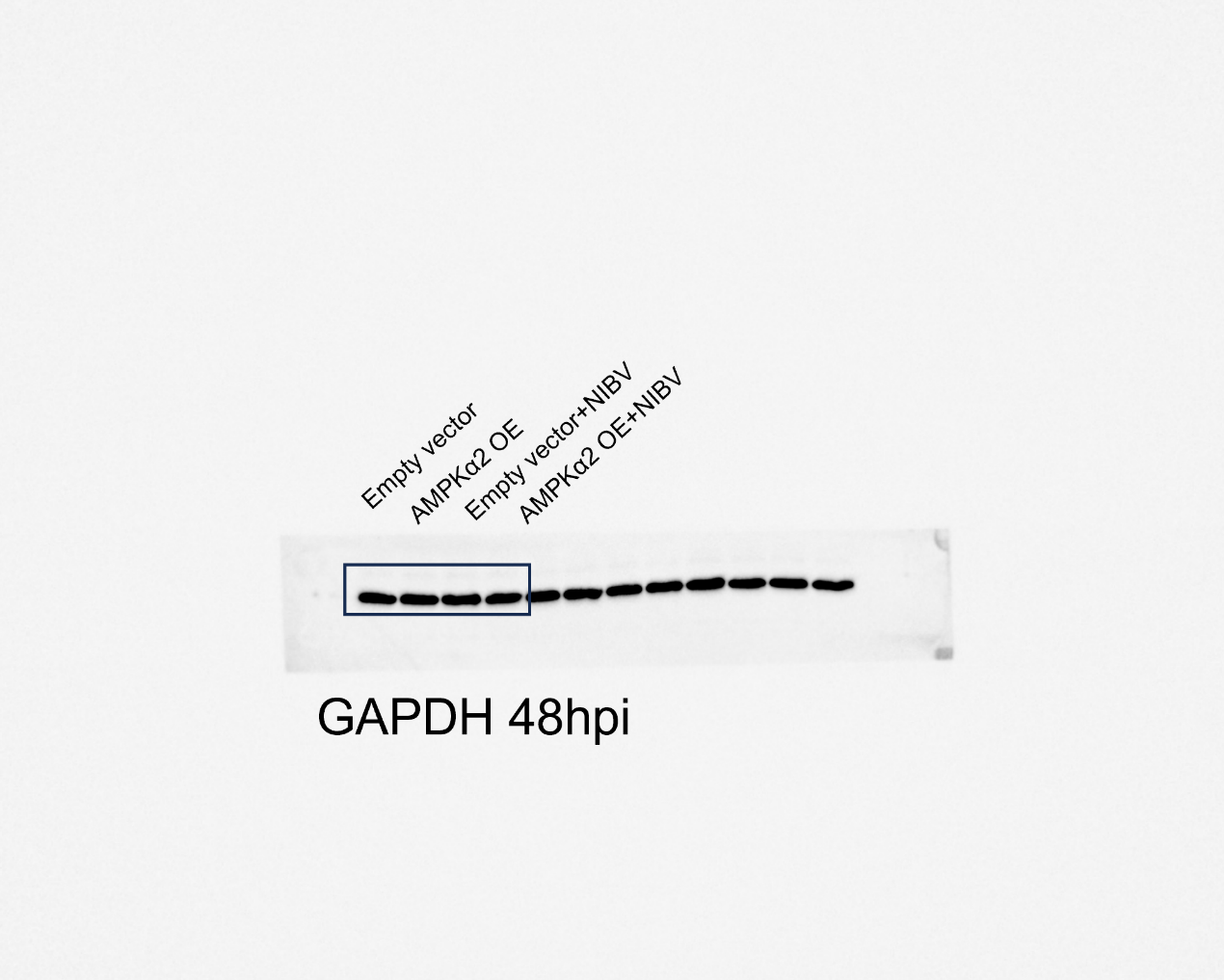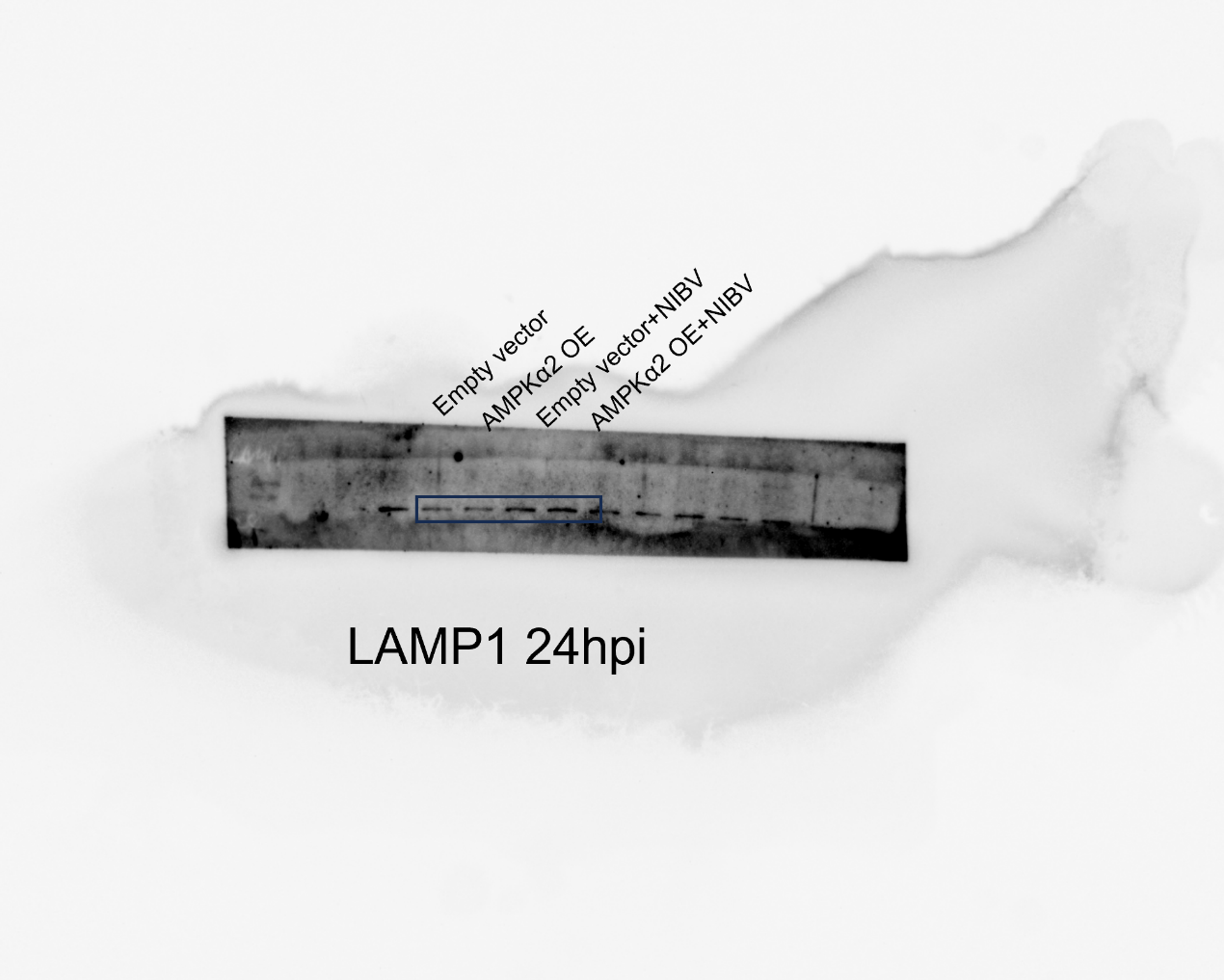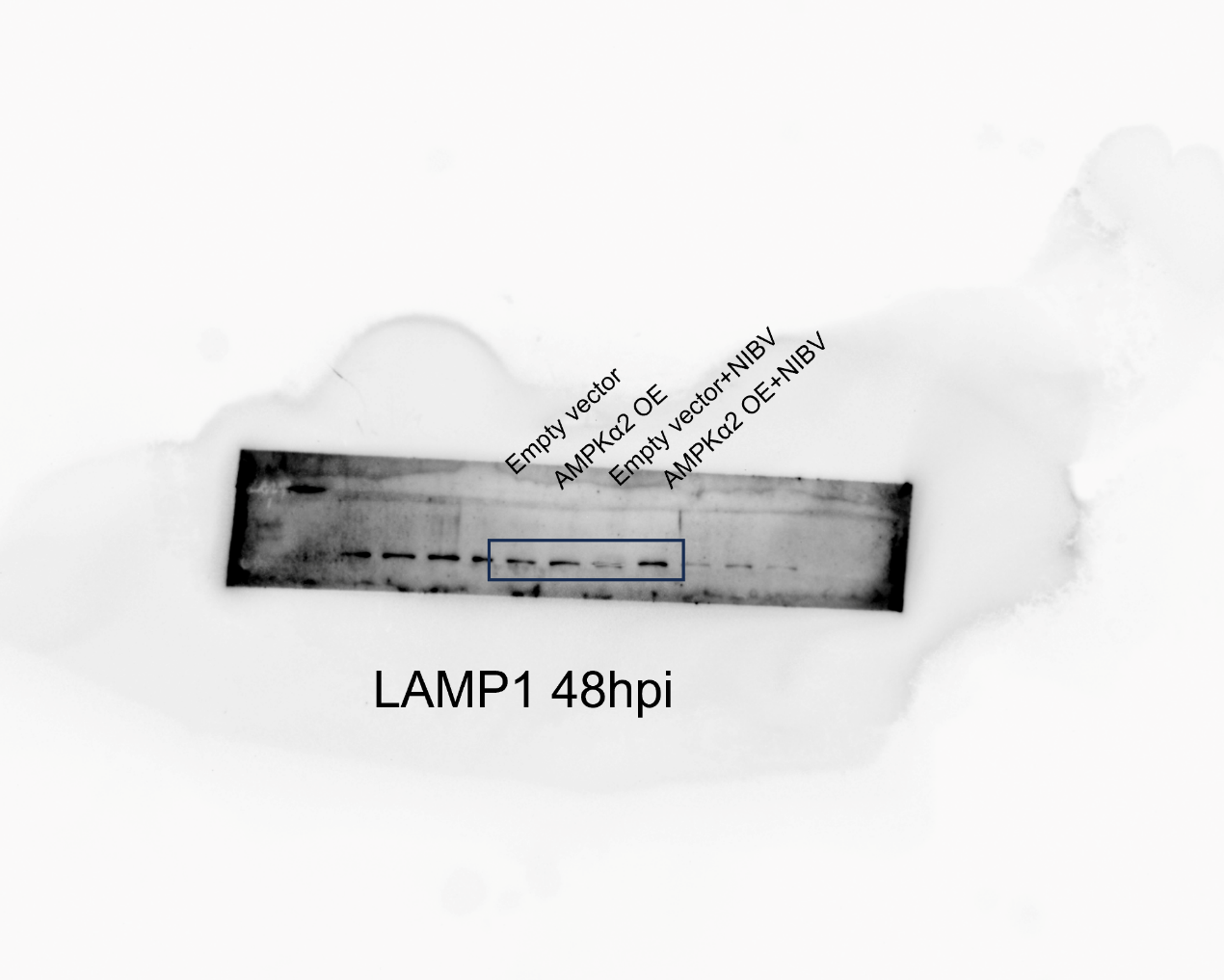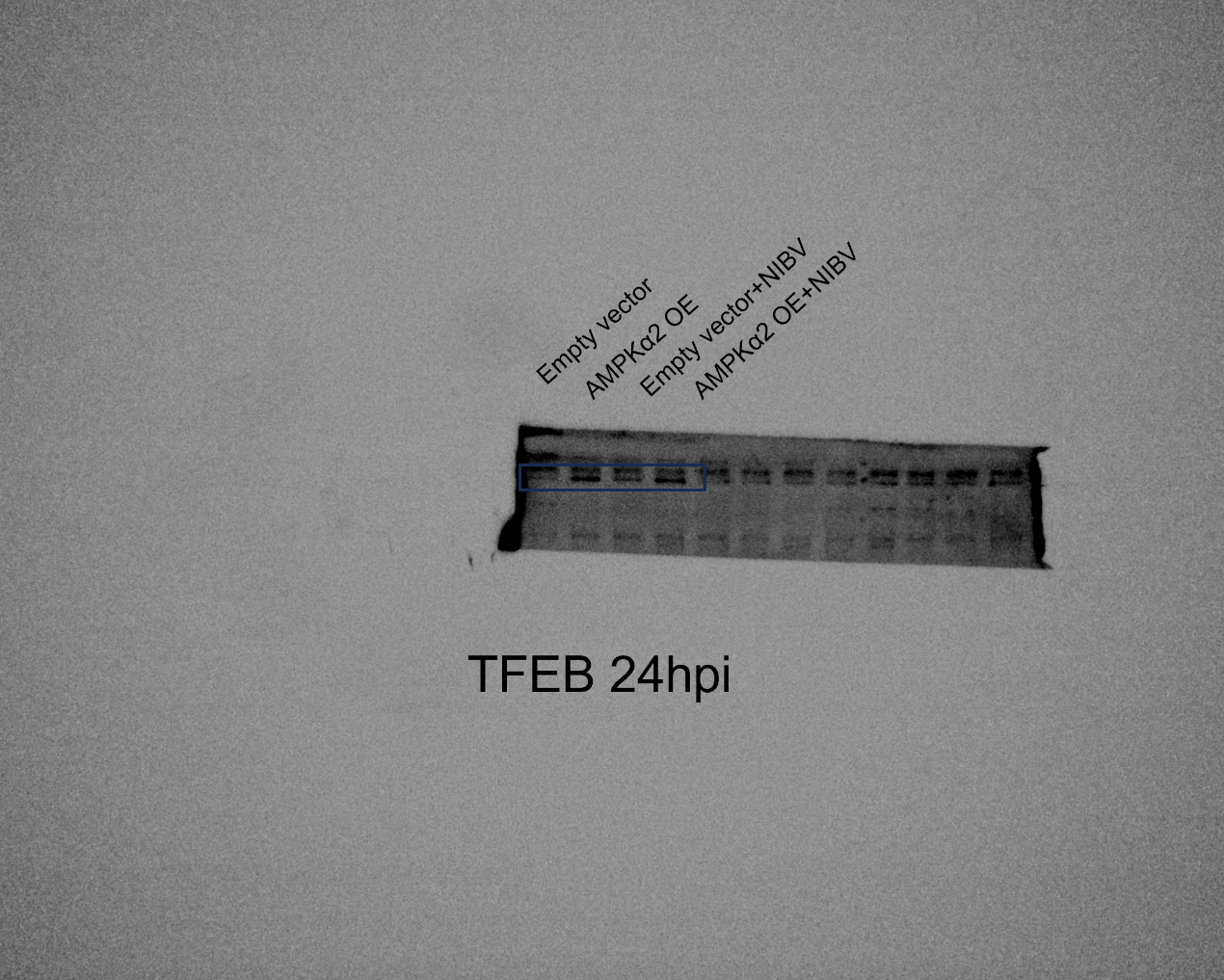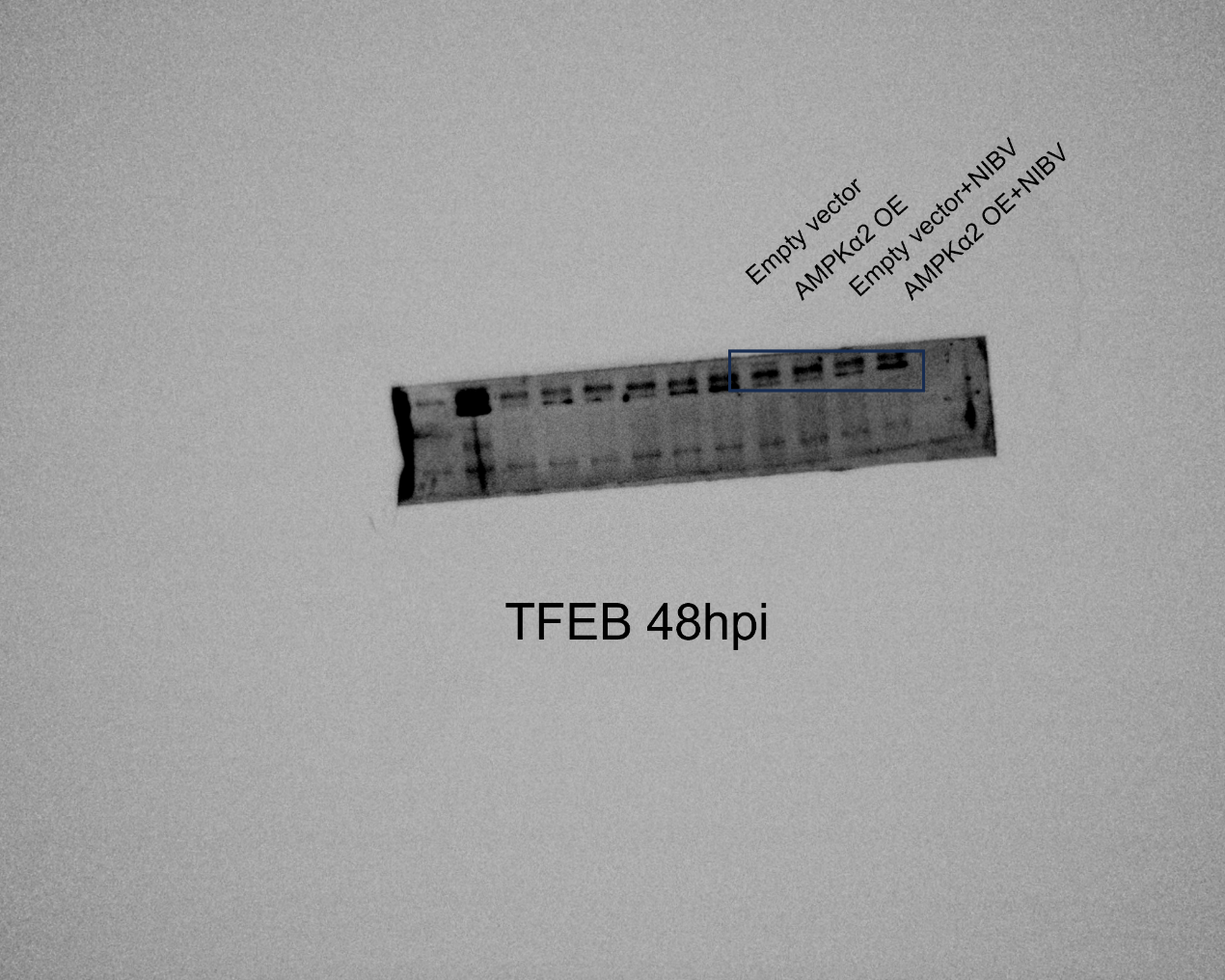** | | |
| **Figure.8 (C and D)** | | |
